# Mechanoresponsive Carbamoyloximes for the Activation of Secondary Amines in Polymers

**DOI:** 10.1002/anie.202207557

**Published:** 2022-08-23

**Authors:** Davide Campagna, Robert Göstl

**Affiliations:** ^1^ DWI—Leibniz Institute for Interactive Materials Forckenbeckstr. 50 52056 Aachen Germany; ^2^ Institute of Technical and Macromolecular Chemistry RWTH Aachen University Worringerweg 1 52074 Aachen Germany

**Keywords:** Amines, Mechanochemistry, Organocatalysis, Photochemistry, Polymers

## Abstract

Mechanophores are molecular moieties that are incorporated into polymers and respond to force with constitutional, configurational, or conformational bond rearrangements to enable functionality. Up to today, several chemically latent motifs have been activated by polymer mechanochemical methods, but the generation of secondary amines remains elusive. Here we report carbamoyloximes as mechanochemical protecting groups for secondary amines. We show that carbamoyloximes undergo force‐induced homolytic bond scission at the N−O oxime bond in polymers thus producing the free amine, as the reaction proceeds via the carbamoyloxyl and aminyl radicals, analogously to its photochemical counterpart. Eventually, we apply the carbamoyloxime motif in a force‐activated organocatalytic Knoevenagel reaction. We believe that this protecting strategy can be universally applied for many other secondary and primary amines in polymer materials.

## Graphical abstract

Carbamoylaldoximes undergo mechanochemical homolytic bond scission of the oxime N−O bond when embedded into polymers. The subsequent radical mechanism yields mainly the nitrile, upon hydrogen dissociation, and the free amine, upon decarboxylation and hydrogen abstraction. Force‐induced organocatalysis is demonstrated for the activation of a secondary amine.

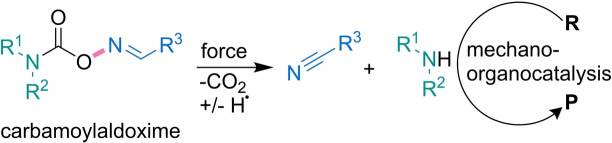

## Introduction

Force is a ubiquitous stimulus that acts on polymer materials in their respective area of application and is usually associated to degradation phenomena. Structural polymers bear loads, polymers in solution are exposed to shear force, and coatings are subjected to friction and wear. Using force to activate function from latent molecular motifs (mechanophores)[^1^, ^2^] is thus a promising method on the one hand to better understand the mechanical behavior of polymers and on the other hand to convert mechanical energy into useful chemical functionality. While the former is generally carried out by the incorporation of optical force probes,[^3^, ^4^] the latter entails examples, such as the release of small molecules,[^5^, ^6^] the activation of latent catalysts,^[7]^ or the initiation of secondary reactions.^[8]^ Therefore, reactive functional groups, such as transition metals,[^9^, ^10^, ^11^, ^12^, ^13^] carbenes,[^13^, ^14^, ^15^] organic acids,[^16^, ^17^, ^18^] latent nucleophiles,[^19^, ^20^] or persistent radicals,[^21^, ^22^] have been activated mechanochemically.

Amines are common functional groups in synthetic chemistry as organic bases, organocatalysts, or simply as nucleophiles for bond formation and polymerization reactions.[^23^, ^24^] Thus, it is highly desirable to activate amines with an external stimulus to gain control over their reactivity. While light, redox, or enzymes have been investigated for this purpose,[^25^, ^26^] a general method to activate latent amines by mechanical force has not yet been reported. Certainly, the release of primary amine‐bearing molecules from latent carbamates has been demonstrated both by Robb and co‐workers^[27]^ and us,^[28]^ and Jung and Yoon recently have shown the mechanochemical generation of imines and their subsequent hydrolysis to primary amines.^[29]^ However, a general method for the direct activation of chemically useful secondary amines is not available.

Several latent protecting groups for the light‐induced uncaging of amines have been reported, mostly so‐called photobase generators (PBGs).^[30]^ PBG motifs often are specific variants of carbamates which generate amines under hydrolytic conditions. However, for ordinary carbamates the nucleophilic attack of H_2_O to form the tetrahedral intermediate in the addition‐elimination hydrolysis mechanism is the rate‐determining step. This would also be true for a hypothetical carbamate mechanophore where the subsequent mechanochemical bond scission step would be severely rate‐limited therefore rendering carbamates unsuitable motifs for this purpose.^[31]^

**Scheme 1:**
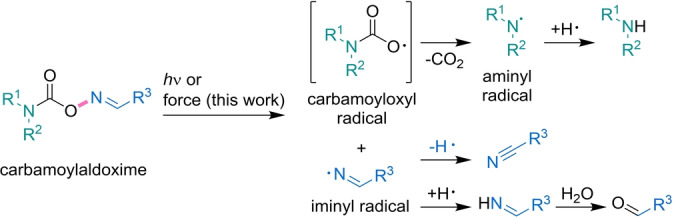
Reaction pathway of the light‐ (PBG, previous work) and force‐induced (MBG, this work) scission of carbamoyloximes. The initially cleaved bond is marked in bold pink.

Concurrently, oxime carbamates (or carbamoyloximes) have a relatively low bond dissociation energy (BDE) of approximately 222 kJ mol^−1^.^[32]^ When these motifs are used as PBGs,^[33]^ the light‐induced homolytic scission of the N−O bond is the key step for a subsequent radical mechanism culminating in the liberation of an amine (Scheme 1). Together, the low BDE, the initial homolytic bond scission step, and the report by Moore and co‐workers on oxime sulfonates as mechanoacid generators^[17]^ thus plausibly support the investigation of carbamoyloximes as mechanobase generators (MBGs).

Here we report the synthesis and mechanochemical activation of carbamoyloximes as a general strategy for the force‐induced generation of amines. Therefore, we rely on carbamoylaldoxime‐protected piperidine incorporated as mechanophore into linear polymer chains. We qualitatively verify the mechanochemical activity of the molecular motif by the constrained geometries simulate external forces (CoGEF) method.^[34]^ We activate the mechanophores in solution by ultrasonication using an immersion probe sonicator (20 kHz) and analyze the reaction pathway in detail compared to its photochemical counterpart. We find that in both cases the amine is generated from the aminyl radical, which is in turn produced from the carbamoyloxyl radical, while the iminyl radical intermediate affords the related nitrile or aldehyde (Scheme 1). Eventually, we exemplarily highlight the use of this motif for force‐activated organocatalysis.

## Results and Discussion

**Scheme 2:**
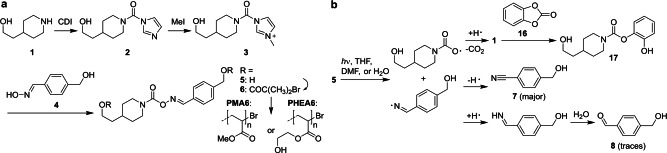
a) Synthesis of carbamoylaldoxime mechanophore diol **5** and subsequent esterification and polymerization reactions. b) Photochemical decomposition pathway of **5**.

The synthesis was carried out adapting the protocol of Batey and co‐workers using carbonyldiimidazole (CDI) as safe phosgene equivalent (Scheme 2a).^[35]^ Due to its high regioselectivity, this method did not require protecting the alkyl alcohols. First, piperidine **1** was carbamoylated to intermediate **2**, which after methylation to **3** reacted neatly with aldoxime **4** affording the carbamoylaldoxime mechanophore diol **5** (see Supporting Information for details). **5** was then esterified with α‐bromoisobutyryl bromide giving bifunctional initiator **6**. This was then used for Cu^0^‐mediated controlled radical polymerization of either methyl acrylate (MA) or hydroxyethyl acrylate (HEA) yielding hydrophobic **PMA6** (various *M*_n_ and *Đ*_M_, Table S1) and hydrophilic **PHEA6** (*M*_n_ *=*44 kDa, *Đ*_M_ *=*1.30). While the carbamoylaldoxime motif was stable at temperatures used for storage (2–8 °C) and mild chemical reactions (50 °C), thermal decomposition was observed above 70 °C (Figure S1–S5).

Hereafter, the photochemical decomposition pathway of mechanophore diol **5** upon irradiation at 254 nm was investigated (Scheme 2b, Table S2). Therefore, we used nonpolar aprotic THF, polar aprotic DMF, and polar protic H_2_O as solvents. The proceeding photochemical reaction was monitored by UV/Vis spectroscopy (Figure S6–S8). The aromatic products derived from the iminyl radical were identified by ^1^H NMR spectroscopy (Figure S9–S10) while ESI‐MS and ^1^H NMR, after derivatization with catechol carbonate **16**, were used to verify amine formation from the aminyl radical (Figure S11–S14). We found that after homolytic scission of the oxime bond, the iminyl radical was converted to nitrile **7**, while aldehyde **8** was only observed in traces. Concomitantly, the carbamoyloxyl radical rapidly decarboxylated yielding the aminyl radical and thereby amine **1**. The latter was detected by formation of carbamate **17** through ring‐opening of the cyclic carbonate **16**, which induced a diagnostic upfield shift of the aromatic protons. Thereby, the photochemical reaction mechanism was plausibly retraced in all three solvents. The photochemical reactivity of **PMA6** and **PHEA6** was investigated in complete analogy to diol **5** in both THF and H_2_O and gave similar results (Figure S15–S17).

**Figure 1:**
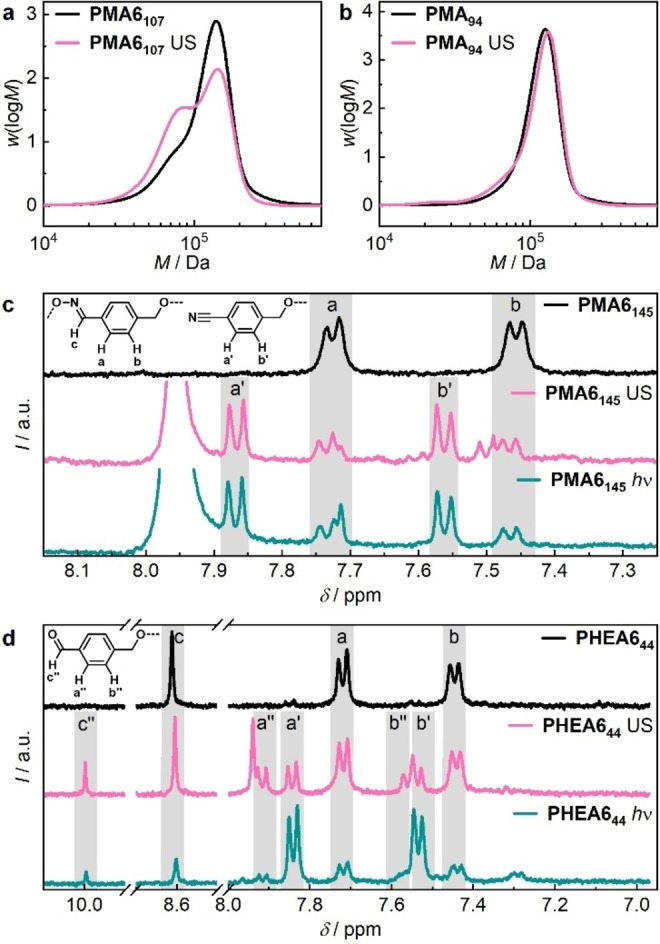
Mechanochemical activation of the carbamoyloxime mechanophore. Molar mass distributions obtained by GPC in THF via RI detector before and after 1 h sonication in THF of a) **PMA6_107_** and b) **PMA_94_** control polymer. Comparison of ^1^H NMR spectra in DMSO‐*d_6_*: c) **PMA6_145_** before (top), after sonication for 5 h in DMF (middle), and after irradiation for 1 h with light at 254 nm in DMF (bottom). d) **PHEA6_44_** before (top), after sonication for 1 h in H_2_O (middle), and after irradiation for 1 h with light at 254 nm in H_2_O (bottom). See Figure S30–S31 for a more detailed ^1^H NMR comparison.

Sonication of **PMA6** in THF and **PHEA6** in H_2_O then verified the mechanochemical activity of the carbamoyloxime (Table S3). Upon sonication of **PMA6** with *M*_n_=107 kDa (**PMA6_107_**) a new peak emerged in the gel permeation chromatography (GPC) elugram at ca. half the initial molar mass suggesting bond scission in the center of the polymer chain (Figure 1a). Sonication of a control PMA chain with *M*_n_ *=*94 kDa and *Đ*_M_=1.13 (**PMA_94_**) under identical conditions showed a considerably reduced mechanochemical conversion (Figure 1b). This underlined that the carbamoyloxime moiety was cleaved at a significantly increased rate compared to random scission of the PMA backbone. The sonication of **PHEA6** in H_2_O and subsequent UV/Vis spectroscopy confirmed this result (Figure S18) against PMA and PHEA control polymers as well as control **5** (Figure S32–S33).

Since accelerated mechanochemical bond scission is a necessary but not a sufficient criterion for the identification of a mechanophore, we subsequently investigated the mechanochemical reaction products by ^1^H NMR spectroscopy. Therefore, **PMA6_145_** was both cleaved photochemically and mechanochemically in DMF. The ^1^H NMR spectra revealed identical reaction products through the diagnostic signals at *δ*=7.86 and 7.57 ppm corresponding to the nitrile **PMA7** (Figure 1c). This was confirmed for a variety of reaction conditions under variable sonication times, molar masses, different solvents, and by dedicated synthesis of a nitrile control polymer (Figure S19–S21). A comparison between photochemical and mechanochemical reactivity was then also carried out in H_2_O using **PHEA6_44_** (Figure 1d). While irradiation with light yielded mostly nitrile **PHEA7**, sonication gave a considerably increased fraction of aldehyde **PHEA8** as product. This implies that under mechanochemical conditions in H_2_O the iminyl radical is likely to either abstract or lose an H‐atom while all other investigated conditions appear to favor solely the detachment of an H‐atom to form the nitrile (cf. Scheme 1 and 2).

Hereafter, we evaluated the mechanochemical selectivity. Therefore, we sonicated **PMA6** and **PHEA6** in different solvents and compared the desired mechanochemical conversion determined by ^1^H NMR to the overall degree of bond scission determined by GPC (Table S4, Figure S22–S29). Depending on the sonication conditions, we found that 42–66 % of the overall bond scission events were productive and occurred at the desired N−O bond of the carbamoylaldoxime. These values were moderate and we recognize that precise computations, in addition to kinetic analyses, will be necessary in the future to obtain a quantitative mechanochemical understanding regarding rate constant, *F*_max_, and *E*_max_. Nevertheless, we performed CoGEF simulations (Figures S36) for a qualitative indication that the N−O bond is the most prone one to undergo scission when the mechanophore is subjected to mechanical force. Furthermore, the mechanochemical origin of the observed reaction was unequivocally verified by the sonication of control polymer chains with terminally substituted mechanophores yielding no productive conversion (Figure S32–S34).

**Figure 2:**
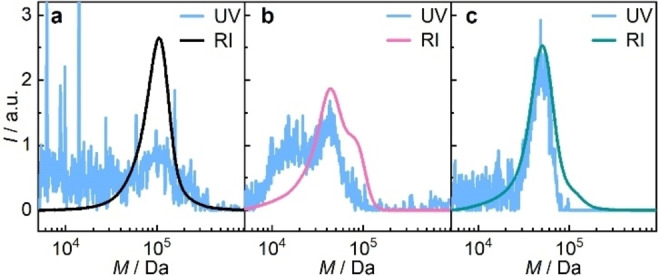
Derivatization of **PMA6_80_** before and after carbamoylaldoxime bond scission with **RhBITC**. GPC elugrams in THF containing both RI and UV (351 nm) detector traces of a) pristine **PMA6_80_**, b) **PMA6_80_** sonicated for 2 h in THF : H_2_O=7 : 3 (v:v), and c) **PMA6_80_** irradiated for 20 min with light at 254 nm in THF.

Though it appeared plausible to assume that the desired amine is formed under mechanochemical conditions in complete analogy to the photochemical pathway after the above results, we carried out a qualitative labelling experiment on sonicated **PMA6**. Therefore, we used Rhodamine B isothiocyanate **RhBITC** which formed a stable thiourea upon reaction with the amine allowing its observation by UV/Vis spectroscopy (Scheme S1). After optimization of the labelling conditions using piperidine as small molecule (Figure S37–S39), **PMA6_80_** was labelled using 10 equiv of **RhBITC**. GPC was then performed using a UV/Vis detector at 351 nm where only the dye absorbed. **PMA6_80_** before sonication did not react with the dye and showed only a very small residual absorbance in the UV channel coinciding with the RI channel of the polymer (Figure 2a). This signal did not significantly stand out from the overall background noise and might be caused by coelution of small amounts of unreacted dye that remained adsorbed non‐covalently to the polymer. On the contrary, **PMA6_80_** after sonication exhibited a clear peak in the UV channel that coincided with the peak of the RI channel at half the initial molar mass (Figure 2b). These results were comparable to dye‐labelled photochemically cleaved **PMA6_80_** (Figure 2c) thus confirming the successful mechanochemical generation of the amine.

**Scheme 3:**
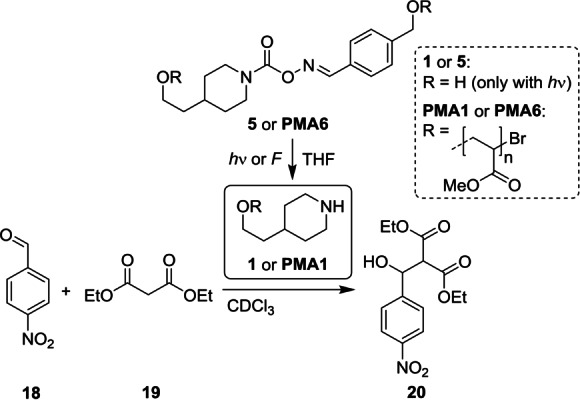
Photo‐ and mechanochemical reactions of **5** and **PMA6**, respectively, yielding amines **1** (by light) or **PMA1** (by ultrasound) to catalyze the Knoevenagel reaction of 4‐nitrobenzaldehyde **18** with diethyl malonate **19** to product **20**.

Hereafter, we aimed to apply this method in a proof‐of‐concept experiment where the mechanochemical bond scission of **PMA6** would yield a secondary amine **PMA1** that would then catalyze a Knoevenagel reaction (Scheme 3). Therefore, we carried out ex situ experiments where the catalyst generated by sonication or irradiation with light was mixed with the reagents in CDCl_3_ after its activation (Table S5 and S6). We used 4‐nitrobenzaldehyde **18** in combination with diethyl malonate **19** in 50‐fold excess to achieve a pseudo first‐order reaction and monitored the conversion to product **20** by ^1^H NMR (Table S7–S9, Figure S40–S45).

First, we verified the catalytic activity of the generated piperidine **1** by irradiation of carbamoyloxime **5** with light and then combining it with the reactants for 2 h. While a 0.49 mm solution of irradiated **5** gave a conversion of 23 % **20**, pristine 1.00 mm **1** yielded 60 % **20**, and pristine 5.50 mm **5** (as a negative control) only 7 % **20** (Table S7 and S9). Analogously, the catalysis experiments performed with photochemically generated **PMA1** from **PMA6** were also successful affording a conversion of 34 % (2 h reaction duration, 0.44 mm, Table S7). This suggested that the PMA chain was fully swollen with CDCl_3_ and reagents permeated the hydrodynamic coil efficiently to the reactive chain‐terminal amine moiety.

**Figure 3:**
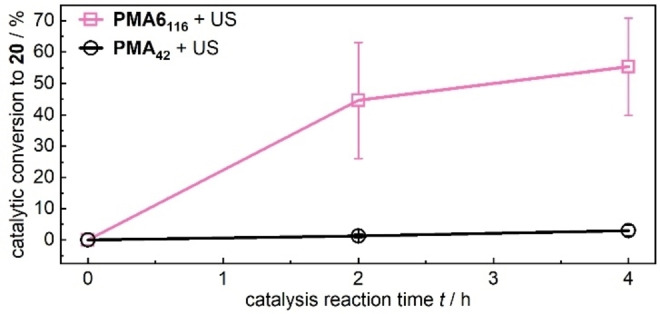
Knoevenagel reaction of 4‐nitrobenzaldehyde **18** with diethyl malonate **19** to product **20** over 2 and 4 h catalysis reaction time in the presence of sonicated **PMA6_116_** or control **PMA_42_**. Mean values±SD from the mean. *N*=3 independent sonication and subsequent catalysis runs of the same polymer sample. All data are reported in Table S8.

Subsequently, a **PMA6_116_** solution was sonicated to generate **PMA1** mechanochemically. Note that we found that the sonication conditions produced acids, electrophiles, and/or other species as minor byproducts that deactivated piperidine at low concentrations. Therefore, *n*‐butylamine (200 μL) was added to the sonication solution as scavenger for these byproducts. This primary amine, however, was removed by dialysis before the reactants were combined and in addition was verified to not catalyze the reaction of **18** with **19**. The sonication of **PMA6_116_** was performed in triplicate for 3 h giving mechanophore conversions between 52–58 % (Table S8). This corresponded to a final mechanophore concentration of 2.35–2.61 mm in the catalysis solution. Thereby, catalytic conversions from 26–63 % **20** were obtained after 2 h reaction, which is significantly higher than the conversions of 1–2 % with sonicated **PMA_42_** as negative control (Figure 3, Table S8). These data clearly underline that the amine **PMA1** was efficiently formed upon sonication and catalyzed the Knoevenagel reaction, therefore demonstrating that the carbamoyloxime motif is suitable for force‐induced organocatalysis applications.

## Conclusion

We here demonstrated a carbamoyloxime motif that acted as latent protecting group for the secondary amine piperidine. The synthesis of the mechanophore is straightforward (only three steps are required), efficient, and versatile, since the involved reactions are essentially not affected by the nature of the amine and the aromatic oxime precursors. We then transformed these carbamoyloximes into the free amine both photochemically by irradiation with light and mechanochemically by ultrasonication. With extensive control experiments, analyses, and qualitative computational investigations, we traced the reaction mechanism and identified all major intermediates and products of the reaction—both in the small molecular variants and when incorporated into polymer chains. Subsequently, we applied this motif for a force‐activated organocatalytic Knoevenagel reaction. Thus we have, for the first time, demonstrated a force‐responsive molecular protecting group for secondary amines—a mechanobase generator. Compared to previous examples of mechanochemical base activation (carbenes or primary amines), this system offers certain advantages. First, the stability of amines is significantly higher than carbenes, hence their applicability is less limited by degradation. Second, the reported activation of primary amines relies on the formation of imines which are then hydrolyzed to primary amines, thus the system requires a second input and the presence of water. The carbamoyloxime mechanophore here reported shows good efficiency for the direct formation of secondary amines with the sole input of mechanical force. Moreover, the mechanoresponsive oxime bond could in principle be exploited more broadly by varying the original structure of the mechanophore on different molecular moieties. Due to this potentially universal character of the latent carbamoyloxime strategy, we believe that this method will be applicable to other secondary and primary amines as well thus opening a new avenue for the mechanochemical control over functional groups in polymer systems.

## Conflict of interest

The authors declare no conflict of interest.

1

## Supporting information

As a service to our authors and readers, this journal provides supporting information supplied by the authors. Such materials are peer reviewed and may be re‐organized for online delivery, but are not copy‐edited or typeset. Technical support issues arising from supporting information (other than missing files) should be addressed to the authors.

Supporting Information

## Data Availability

The data that support the findings of this study are documented in the Supporting Information and are openly available in Zenodo at https://doi.org/10.5281/zenodo.6849938, reference number [36].
