# Supplementary material for: Mechanoresponsive Carbamoyloximes for the Activation of Secondary Amines in Polymers
Source: Angew Chem Int Ed Engl. 2022 Aug 23;61(39):e202207557. doi: 10.1002/anie.202207557 (PMC9805098; doi:10.1002/anie.202207557)
Supplement: Supplementary file 1 — Supporting Information [file ANIE-61-0-s001.pdf]

Angewandte Chemie
Eine Zeitschrift der Gesellschaft Deutscher Chemiker

Supporting Information

# Mechanoresponsive Carbamoyloximes for the Activation of Secondary Amines in Polymers

*D. Campagna, R. Göstl**

# Supporting Information

# Table of contents


1. Materials and methods ... 3
  1.1. Materials ... 3
  1.2. NMR spectroscopy ... 3
  1.3. Chromatography ... 3
  1.4. Photochemistry ... 3
  1.5. Ultrasonication ... 4
2. Experimental procedures ... 5
  2.1. Synthesis of small molecules ... 5
    2.1.1. Carbonylimidiazole **2** ... 5
    2.1.2. Carbamoylimidazolinium salt **3** ... 5
    2.1.3. Aldoxime **4** ... 5
    2.1.4. Carbamoylaldoxime diol **5** ... 6
    2.1.5. Carbamoylaldoxime mechanophore initiator **6** ... 6
    2.1.6. Nitrile control initiator **9** ... 6
    2.1.7. Aldehyde control initiator **10** ... 7
    2.1.8. Carbamoylimidazole **11** ... 7
    2.1.9. Carbamoylimidazolium salt **12** ... 7
    2.1.10. Carbamoylaldoxime control **13** ... 8
    2.1.11. Carbamoylaldoxime control initiator **14** ... 8
    2.1.12. Control initiator **15** ... 9
    2.1.13. Catechol carbonate **16** ... 9
  2.2. Polymer synthesis ... 10
    2.2.1. $Cu^0$-wire activation ... 10
    2.2.2. General procedure for $Cu^0$-mediated CRP with methyl acrylate (MA) ... 10

2.2.3. Purification of hydroxyethyl acrylate ........ 10
2.2.4. General procedure for $Cu^0$-mediated CRP with hydroxyethyl acrylate (HEA) ........ 10
2.2.5. Synthesis of **PMA6$_{107}$** ........ 11
2.2.6. Synthesis of **PMA6** with different molar masses ........ 11
2.2.7. Synthesis of **PHEA6$_{44}$** ........ 11
2.2.8. Chain-terminal control **PMA14** ........ 12
2.2.9. Chain-terminal control **PHEA14** ........ 12
2.2.10. Nitrile control **PMA7** ........ 12
2.2.11. Nitrile control **PHEA7** ........ 13
2.2.12. Aldehyde control **PMA8** ........ 13
2.2.13. Aldehyde control **PHEA8** ........ 13
2.2.14. PMA control polymers **PMA** ........ 13
2.3. Thermal stability experiments ........ 15
2.4. Photochemical experiments ........ 18
2.4.1. The mechanism ........ 18
2.4.2. General procedure and list of experiments ........ 19
2.4.3. Small molecules: the iminyl pathway ........ 19
2.4.4. Small molecules: amine detection ........ 23
2.4.5. Linear polymers ........ 28
2.5. Ultrasonication experiments ........ 31
2.5.1. General procedure and list of experiments ........ 31
2.5.2. Mechanochemical activity ........ 32
2.5.3. Influence of ultrasonication parameters ........ 32
2.5.4. Mechanochemical selectivity ........ 36
2.5.5. Complete NMR analysis after irradiation and sonication (Figure 1c,d) ........ 41
2.5.6. Control experiments ........ 43
2.6. Computational experiments ........ 47
2.7. Derivatization with **RhBITC** ........ 48
2.8. Organocatalytic experiments ........ 51
2.8.1. General procedure and list of experiments ........ 51
2.8.2. Photochemically induced organocatalysis ........ 54
2.8.3. Mechanochemically induced organocatalysis ........ 56
3. Supporting NMR, MS, and GPC spectra ........ 60
4. References ........ 94
5. Author contributions ........ 94

# 1. Materials and methods

## 1.1. Materials

Solvents and reagents were purchased from different suppliers and used without further purification, with the exception of hydroxyethyl acrylate monomer (*cf.* Section 2.2). TLC was performed on Merck TLC silica gel 60 F254 TLC plates with a fluorescent indicator. Purification by flash chromatography was performed manually on silica gel (40-63 µm), or on a Reveleris X2 Flash Chromatography System (Büchi), equipped with UV and ELSD detectors. Prior to the polymerization, methyl acrylate was filtered over neutral $Al_2O_3$, while hydroxyethyl acrylate was distilled *in vacuo*. $Cu^0$-wire was activated with concentrated HCl and stored under Ar atmosphere.

## 1.2. NMR spectroscopy

$^1$H- and $^{13}$C-NMR spectra were recorded on a 400 MHz Bruker Avance 400 spectrometer ($^{13}$C: 101 MHz) or a 300 MHz Bruker DPX 300 spectrometer ($^{13}$C: 75 MHz); chemical shifts are reported in δ units, using residual protonated solvent signals as internal standard (DMSO-$d_6$ at δ = 2.50 ppm; $CDCl_3$ at δ = 7.26 ppm). The multiplicity of the peaks were indicated as following: s = singlet, d = doublet, t = triplet, q = quartet, m = multiplet. When higher resolution was required, $^1$H-NMR was recorded applying 128 or 256 scans. High-resolution ESI mass spectrometry spectra were recorded on a microTOF-QII instrument from Bruker Daltonik.

## 1.3. Chromatography

Molar masses ($M_n$ and $M_w$) and molar mass distributions ($Đ_M$) were determined by gel permeation chromatography (GPC). Prior to analysis, every prepared sample was filtered through a CHROMAFIL PTFE membrane with 0.45 µm pore size. GPC was carried with two different solvents:

Tetrahydrofuran (THF) (≥99.7%, unstabilized, HiPerSolv CHROMANORM® HPLC grade, VWR) as the eluent. The machine was equipped with an HPLC pump (1260 Infinity II, Agilent), a refractive index detector (RI) (1290 Infinity II, Agilent), an UV-detector (UV-2075plus, Jasco) and a multi angle light scattering (MALS) (SLD 7100, Polymer Standards Service). The samples contained 250 mg·mL$^{-1}$ 3,5-di-*tert*-4-butylhydroxytoluene (BHT, ≥99%, Fluka) as the internal standard. One pre-column (8×50 mm) and four SDplus gel columns (8×300 mm, MZ Analysentechnik) were applied at a flow rate of 1.0 mL·min$^{-1}$ at 20 °C. The diameter of the gel particles measured 5 µm, the nominal pore widths were 50, $10^2$, $10^3$, and $10^4$ Å.

*N,N*-Dimethylformamide (DMF) (≥99.9%, HiPerSolv CHROMANORM® HPLC grade, VWR) as the eluent. The machine was equipped with an HPLC pump (1260 Infinity, Agilent), a dual RI-/Visco detector (ETA-2020, WGE), a UV-detector (VWD, 1290 Infinity II, Agilent). The eluent contained 1 g·L$^{-1}$ LiBr (≥99%, Sigma-Aldrich). The samples contained 2 µL·mL$^{-1}$ toluene (≥99%, Sigma-Aldrich) as internal standard. One pre-column (8×50 mm) and three GRAM gel columns (8×300 mm, Polymer Standards Service) were applied at a flow rate of 1.0 mL·min$^{-1}$ at 60 °C. The diameter of the gel particles measured 10 µm, the nominal pore widths were 30, $10^2$, and $10^3$ Å.

Calibration was performed using narrowly distributed poly(methyl methacrylate) standards (Polymer Standards Service). Results were evaluated using the PSS WinGPC UniChrom software (Version 8.3.2).

Ultra performance liquid chromatography-mass spectrometry (UPLC-MS) was performed on a Waters ACQUITY UPLC system assembled with multiple modules and equipped with a CSH C18 (1.7 µm 100×2.10 mm) column, operated at 40 °C with a gradient mixture of MeCN/$H_2O$. The system consisted of an ACQUITY UPLC I-Class Column Manager System, a Waters Fraction Manager Analytical System, an ACQUITY UPLC Photodiode Array Detector PDA, and an ACQUITY QDA detector for the mass analysis.

## 1.4. Photochemistry

Irradiation experiments were performed with UV light in two different ways depending on the type of light source and setup:

High intensity: the irradiation source was a Herloab UV-6 S/L hand lamp as 6 W blacklight source operating at two wavelengths, UV-A (long, 365 nm, 850 µW·cm$^{-2}$) and UV-C (short, 254 nm, 600 µW·cm$^{-2}$). This lamp was used for

all the photochemical experiments where the irradiation source was indicated as "high intensity". Every experiment was performed by irradiating a capped quartz cuvette (*d* = 1.00 cm) located below the lamp at a distance of approximately 5 cm (at this distance, the intensity was found be in a range of 0.6-0.8 $mW \cdot cm^{-2}$, measured with a radiometer). Every irradiation was performed at room temperature and covering the whole system with a proper box. The relative high intensity of this irradiation source is given by the total integral of the emission spectra, where its peak is at 254 nm. This setup was more appropriate when higher concentrations of the sample were required (*e.g.*, for NMR analysis, chemical derivatization, catalytic trials).

Low intensity: the irradiation source was a LOT-ORIEL high-pressure Hg lamp (500 W). The peak of the emission spectrum at 254.65 nm was selected through a monochromator, whose outlet was connected to an optical fiber, in turn connected to a cell holder located inside a spectrometer for in-line measurements. This setup was used for every experiment where the irradiation source was indicated as "low intensity". A capped quartz cuvette (*d* = 1.00 cm) was placed inside the thermostat holder and irradiated at 25 °C. The relative low intensity of this irradiation source is given by the narrow integral of the filtered light through the monochromator. This setup was more appropriate when low concentrations were required (*e.g.*, UV-vis absorption spectroscopy).

Single UV-vis absorption measurements were performed in a capped quartz cuvette (*d* = 1.00 cm) on a Thermo Evolution 300 spectrometer. UV-Vis absorption measurements during irradiation were performed in a capped quartz cuvette (*d* = 1.00 cm) on an Agilent Cary 60 spectrometer equipped with a Peltier thermostated cell holder.

## 1.5. Ultrasonication

Ultrasonication experiments were carried out with a Sonic VCX 500 ultrasonic processor purchased from Sonics & Materials, equipped with a 13 mm probe, at $f$ = 20 kHz and applying a pulse of 1 s "on", 1 s "off". The amplitude was set at 30% ($I_P$ = 7.4 $W \cdot cm^{-2}$) or 60% ($I_P$ = 17.8 $W \cdot cm^{-2}$). The power intensities $I_P$ were determined by a proper calibration using an established calorimetric method.[1] Samples were injected in a 50 mL Suslick vessel (from Sonics & Materials or from Zinsser Analytic), bubbled with $N_2$ for 10 min, sealed, and sonicated in a $H_2O$-ice bath (2-8 °C). Prior to every subsequent experiment, every solution was filtered to remove metallic particles released by the probe. In case of sonication in THF, DMF, THF/$H_2O$ mixture, the filtration was carried out with a CHROMAFIL PTFE membrane with 0.45 µm pore size. In case of pure $H_2O$, the filtration was carried out with a CHROMAFIL PA membrane with 0.45 µm pore size. Organic solvents were used at p.a. grade, while $H_2O$ was used at HPLC grade.

# 2. Experimental procedures

## 2.1. Synthesis of small molecules

### 2.1.1. Carbonylimidiazole 2

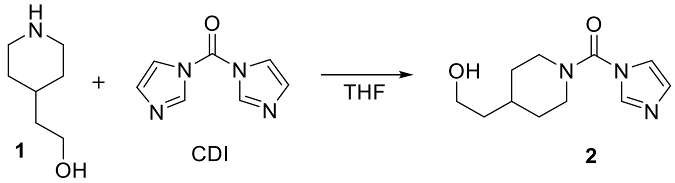


Carbonyldiimidazole (CDI, 874 mg, 5.39 mmol, 1.1 equiv.) was suspended in dry THF (10 mL). 4-piperidinethanol **1** (633 mg, 4.90 mmol,1.0 equiv.) was then added to the mixture; which was stirred and refluxed for 16 h under $N_2$ stream. Afterwards, all volatiles were removed *in vacuo*, the obtained oil was dissolved in $CH_2Cl_2$ (30 mL) and washed with $H_2O$ (2×10 mL) and brine (10 mL). The aqueous phase was extracted with more $CH_2Cl_2$ (20 mL) and the organic layers were collected and dried over $MgSO_4$. After filtration, the solvent was removed *in vacuo*. The final product was obtained without further purification (clear oil, 82%). **$^1$H-NMR (400 MHz, $CDCl_3$):** δ = 7.79 (s, 1H), 7.13 (s, 1H), 7.00 (s, 1H), 4.18 – 3.99 (m, 4H), 3.65 (t, 2H), 2.96 (t, 2H), 2.74 – 2.58 (s broad, OH), 1.82 – 1.71 (m, 2H), 1.68 – 1.56 (m, 3H), 1.55 – 1.42 (m, 2H), 1.24 – 1.19 (m, 1H). See Figure S46. **$^{13}$C-NMR (101 MHz, $CDCl_3$):** δ = 155.38, 150.73, 136.82, 129.42, 118.01, 62.74, 59.73, 46.74, 44.08, 39.17, 35.24, 33.08, 32.43, 31.92. See Figure S47. **ESI$^+$ HRMS:** *m/z* = 224.1478 (calcd. 224.1394 for $MH^+$). See Figure S48.

### 2.1.2. Carbamoylimidazolinium salt 3

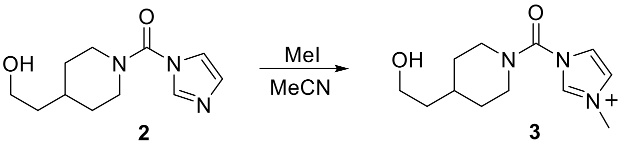


Carbamoylimidazole **2** (669 mg, 3.00 mmol, 1 equiv.) was dissolved in dry MeCN (6 mL) in a Schlenk flask under $N_2$ atmosphere. MeI (747 µL, 12.00 mmol, 4 equiv.) was slowly injected and the mixture was then stirred at r.t. for 24 h, observing the coloration of the solution slowly turning to light yellow. All the volatiles were removed *in vacuo* to yield the final product without further workup (yellow solid, quant.). **$^1$H-NMR (400 MHz, DMSO-$d_6$):** δ = 9.54 (s, 1H), 8.01 (s, 1H), 7.86 (s, 1H), 4.08 – 3.98 (m, 2H), 3.92 (s, 3H), 3.45 (t, 2H), 3.17 – 3.02 (m, 2H), 2.72 (s, OH), 1.77 – 1.48 (m, 4H), 1.44 – 1.20 (m, 2H), 1.05 – 0.90 (m, 1H). See Figure S49. **$^{13}$C-NMR (101 MHz, DMSO-$d_6$):** δ = 155.03, 146.97, 124.15, 121.43, 62.86, 58.54, 44.13, 36.84, 35.17, 32.37, 31.42. See Figure S50. **ESI$^+$ HRMS:** *m/z* = 238.1650 (calcd. 238.1550 for $M^+$). See Figure S51.

### 2.1.3. Aldoxime 4

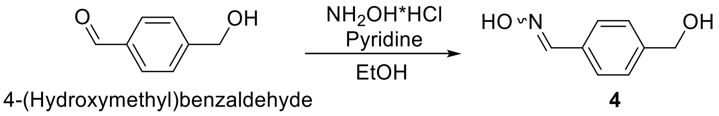


4-(Hydroxymethyl)benzaldehyde **8** (1.09 g, 8.00 mmol, 1 equiv.) and hydroxylamine hydrochloride ($NH_2OH{\cdot}HCl$, 611 mg, 8.80 mmol, 1.1 equiv.) were dissolved in abs. EtOH (80 mL). Pyridine (16 mL) was added and the mixture was stirred and refluxed for 24 h. Afterwards, all volatiles were removed *in vacuo* and $H_2O$ (20 mL) was added to the oily residue, affording a white precipitate which was filtered off, washed with more $H_2O$ (2×20 mL), dried, and collected. The remaining aqueous layers were also collected and extracted with $Et_2O$ (2×20 mL). The combined organic layers were dried over $MgSO_4$, filtered, and the solvent was removed *in vacuo.* The final product was obtained without further purification (white solid, 87%). **$^1$H-NMR (300 MHz, DMSO-$d_6$):** δ = 11.16 (s, OH), 8.13 (s, 1H), 7.60 – 7.51 (m, 2H), 7.43 – 7.30 (m, 2H), 4.52 (s, 2H). See Figure S52. **$^{13}$C-NMR (101 MHz, DMSO-$d_6$):** δ =

148.50, 144.29, 131.96, 127.13, 126.67, 63.08. See Figure S53. **ESI⁺ HRMS**: *m/z* = 152.0789 (calcd. 152.0706 for $MH^+$). See Figure S54.

## 2.1.4. Carbamoylaldoxime diol 5

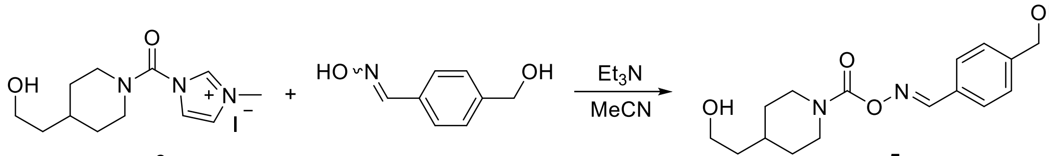


**3** **4** **5**

Aldoxime **4** (453 mg, 3.00 mmol, 1 equiv.) and carbamoylimidazolinium salt **3** (2.73 g, 7.50 mmol, 2.5 equiv.) were dissolved in dry MeCN (15 mL) in a Schlenk flask under $N_2$ atmosphere. Subsequently, dry $Et_3N$ (1.08 mL, 7.50 mmol, 2.5 equiv.) was slowly injected, whereupon the color of the solution turned from light yellow to transparent. The mixture was then stirred at r.t. overnight. All the volatiles were removed *in vacuo*, the oily residue was dissolved in $CH_2Cl_2$ (60 mL), washed with $H_2O$ (15 mL), and brine (15 mL). The organic layer was dried over $MgSO_4$ and filtered. The solvent was removed *in vacuo* and subjected to column chromatography (silica, $CH_2Cl_2$:MeOH = 99:1, gradient to 95:5) to afford the final product (clear oil, 76%). **$^1$H-NMR (400 MHz, $CDCl_3$):** δ = 8.24 (s, 1H), 7.67 – 7.60 (m, 2H), 7.37 – 7.30 (m, 2H), 4.66 (s, 2H), 4.17 – 4.06 (m, 2H), 3.64 (t, 2H), 2.83 – 2.67 (m, 2H), 2.35 – 1.99 (s broad, OH), 1.72 – 1.54 (m, 4H), 1.47 (q, 2H), 1.18 – 1.12 (m, 1H). See Figure S55. **$^{13}$C-NMR (101 MHz, $CDCl_3$):** δ = 154.95, 154.18, 144.50, 129.65, 128.34, 127.06, 64.65, 60.13, 44.56, 39.12, 35.50, 32.42. See Figure S56. **ESI⁺ HRMS:** *m/z* = 307.1788 (calcd. 307.1652 for $MH^+$). See Figure S57.

## 2.1.5. Carbamoylaldoxime mechanophore initiator 6

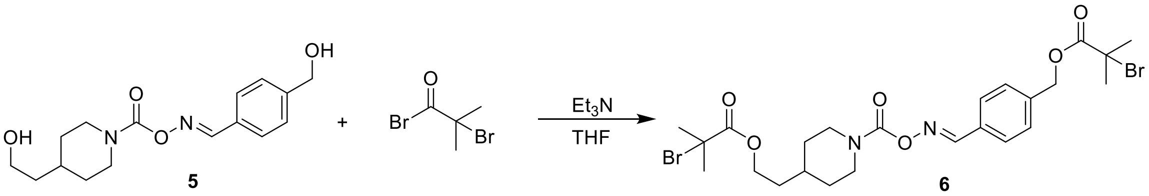


**5** **6**

Carbamoylaldoxime mechanophore **5** (245 mg, 0.80 mmol, 1 equiv.) and dry $Et_3N$ (450 µL, 3.20 mmol, 4 equiv.) were dissolved in dry THF (8 mL) in a Schlenk flask under $N_2$ atmosphere. Subsequently, a solution of α-bromoisobutyryl bromide (395 µL, 3.20 mmol, 4 equiv.) in dry THF (2 mL) was added dropwise over 30 min at 0 °C. Then, the mixture was stirred at r.t. overnight. Afterwards, all the volatiles were removed *in vacuo*, the residue was dissolved in EtOAc (30 mL), washed with $H_2O$ (5 mL), sat. aq. $NaHCO_3$ sol. (5 mL), and brine (5 mL). The organic layer was dried over $MgSO_4$ and the salt was filtered off. The solvent was removed *in vacuo* and a further purification by means column chromatography (silica, EtOAc:heptane = 1:9, gradient to 2:1) afforded the final product (white ductile solid, 37%). **$^1$H-NMR (400 MHz, $CDCl_3$):** δ = 8.25 (s, 1H), 7.71 – 7.64 (m, 2H), 7.39 – 7.32 (m, 2H), 5.16 (s, 2H), 4.17 (t, 2H), 4.07 (t, 2H), 2.88 – 2.76 (m, 2H), 2.69 (t, 2H), 1.89 (s, 6H), 1.86 (s, 6H), 1.75 – 1.62 (m, 2H), 1.59 – 1.52 (m, 2H), 1.21 – 1.17 (m, 1H). See Figure S58. **$^{13}$C-NMR (101 MHz, $CDCl_3$):** δ = 155.45, 153.86, 153.56, 138.60, 130.58, 128.43, 128.05, 66.91, 63.66, 44.00, 34.80, 32.95, 31.87, 30.75. See Figure S59. **ESI⁺ HRMS:** m/z = found 603.0731 (calcd. 603.0700 for $MH^+$). See Figure S60.

## 2.1.6. Nitrile control initiator 9

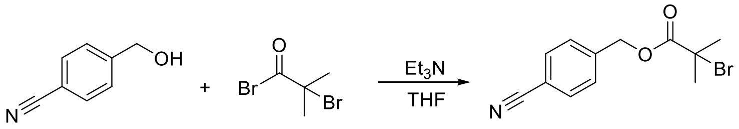


**9**

4-cyanobenzylalcohol **7** (266 mg, 2.0 mmol, 1 equiv.) and dry $Et_3N$ (843 µL, 6.0 mmol, 3 equiv.) were dissolved in dry THF (10 mL) in a Schlenk flask under $N_2$ atmosphere. Subsequently, a solution of α-bromoisobutyryl bromide (742 µL, 6.0 mmol, 3 equiv.) in dry THF (10 mL) was added dropwise over 30 min at 0 °C. Then, the mixture was stirred at r.t. overnight. Afterwards, all the volatiles were removed *in vacuo*, the residue was dissolved in EtOAc (30 mL), washed with $H_2O$ (10 mL), sat. aq. $NaHCO_3$ sol. (10 mL), and brine (10 mL). The organic layer was dried over $MgSO_4$ and the salt was filtered off. The solvent was removed *in vacuo* and a further purification by means column chromatography (silica, EtOAc:petroleum ether = 1:4) afforded the final product (clear oil, 84%). **$^1$H-NMR (400 MHz, $CDCl_3$):** δ = 7.72 – 7.64 (m, 2H), 7.53 – 7.45 (m, 2H), 5.25 (s, 2H), 1.96 (s, 6H). See Figure S61. **$^{13}$C-NMR (101 MHz, $CDCl_3$):** δ = 171.25, 140.68, 132.47, 128.03, 118.51, 112.17, 77.38, 77.06, 76.74, 66.27, 55.23, 30.69. See Figure S62. **ESI$^+$ HRMS:** *m/z* = 282.0253 (calcd. 282.0124 for $MH^+$). See Figure S63.

### 2.1.7. Aldehyde control initiator 10

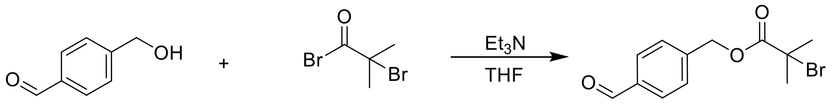


**10**

4-(Hydroxymethyl)benzaldehyde **8** (1.22 g, 8.0 mmol, 1 equiv.) and dry $Et_3N$ (3.37 mL, 24.0 mmol, 3 equiv.) were dissolved in dry THF (30 mL) in a Schlenk flask under $N_2$ atmosphere. Subsequently, a solution of α-bromoisobutyryl bromide (2.96 mL, 24.0 mmol, 3 equiv.) in dry THF (10 mL) was added dropwise over 30 min at 0 °C. Then, the mixture was stirred at r.t. overnight. Afterwards, all the volatiles were removed *in vacuo*, the residue was dissolved in EtOAc (100 mL), washed with $H_2O$ (30 mL), at. aq. $NaHCO_3$ sol. (30 mL), and brine (30 mL). The organic layer was dried over $MgSO_4$ and the salt was filtered off. The solvent was removed *in vacuo* and a further purification by means column chromatography (silica, EtOAc:Heptane = 1:4) afforded the final product (clear oil, 48%). **$^1$H-NMR (400 MHz, $CDCl_3$):** δ = 9.95 (s, 1H), 7.86 – 7.79 (m, 2H), 7.51 – 7.44 (m, 2H), 5.21 (s, 2H), 1.90 (s, 6H). See Figure S64. **$^{13}$C-NMR (101 MHz, $CDCl_3$):** δ = 191.74, 171.31, 142.11, 136.20, 130.03, 127.96, 77.38, 77.06, 76.74, 66.62, 55.36, 30.73. See Figure S65. **ESI$^+$ HRMS:** *m/z* = 285.0256 (calcd. 285.0121 for $MH^+$). See Figure S66.

### 2.1.8. Carbamoylimidazole 11

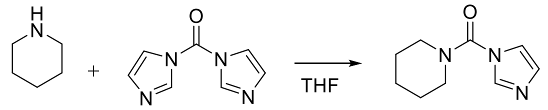


**11**

Carbonyldiimidazole (CDI, 1.42 g, 8.80 mmol, 1.1 equiv.) was suspended in THF dry (15 mL), piperidine (790 µL, 8.00 mmol, 1 equiv.) was then added to the mixture; this latter was stirred and refluxed for 16 h under $N_2$ stream. Afterwards, all volatiles were removed *in vacuo*, the obtained oil was dissolved in $CH_2Cl_2$ (30 mL) and washed with $H_2O$ (2×20 mL) and brine (20 mL). The aqueous phase was extracted with more $CH_2Cl_2$ (50 mL), the organic layers were collected and dried over $MgSO_4$. The salt was filtered off, the solvent was removed *in vacuo*, and the product was obtained without further purification (clear oil, 85%). **$^1$H-NMR (400 MHz, $CDCl_3$):** δ = 7.79 (s, 1H), 7.13 (s, 1H), 7.02 (s, 1H), 3.51 – 3.44 (m, 4H), 1.71 – 1.56 (m, 6H). See Figure S67. **$^{13}$C.NMR (101 MHz, $CDCl_3$):** δ = 129.55, 117.99, 47.54, 25.78, 24.18. See Figure S68. **ESI$^+$ HRMS:** *m/z* = 180.1149 (calcd. 180.1131 for $MH^+$). See Figure S69.

### 2.1.9. Carbamoylimidazolium salt 12

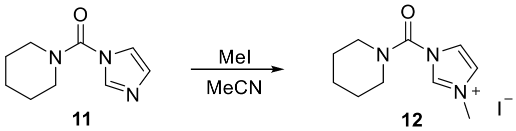


**11** **12**

Carbamoylimidazole **11** (1.23 g, 6.85 mmol, 1 equiv.) was dissolved in dry MeCN (11 mL) in a Schlenk flask under $N_2$ atmosphere. MeI (1.70 mL, 27.40 mmol, 4 equiv.) was slowly injected; the mixture was then stirred at r.t. for 24 h, observing the coloration of the solution slowly turning to light yellow. All the volatiles were removed *in vacuo* to yield the final product without further workup (yellow solid, quantitative). **$^1$H-NMR (400 MHz, DMSO-*d*$_6$):** δ = 9.56 (s, 1H), 8.01 (s, 1H), 7.86 (s, 1H), 3.94 – 3.90 (m, 2H), 3.37 – 3.32 (m, 2H), 1.66 – 1.56 (m, 6H). See Figure S70. **$^{13}$C-NMR (101 MHz, DMSO-*d*$_6$):** δ = 144.73, 135.62, 121.88, 119.14, 37.19, 34.62, 34.62, 23.16, 21.43. See Figure S71. **ESI$^+$ HRMS**: *m/z* = 194.1314 (calcd. 194.1288 for M$^+$). See Figure S72.

## 2.1.10. Carbamoylaldoxime control 13

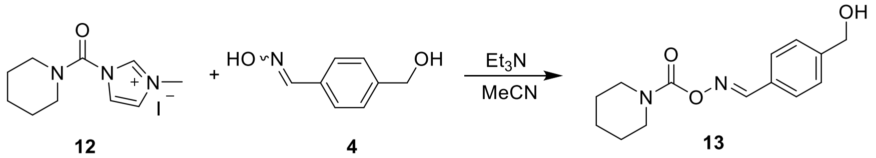


12 4 13

Aldoxime **4** (211 mg, 1.40 mmol, 1 equiv.) and carbamoylimidazolium salt **12** (900 mg, 2.80 mmol, 2.0 equiv.) were dissolved in dry MeCN (10 mL) in a Schlenk flask under $N_2$ atmosphere. Subsequently, dry $Et_3N$ (404 µL, 2.80 mmol, 2.0 equiv.) was slowly injected, observing the coloration of the solution immediately turning from light yellow to transparent. The mixture was then stirred at r.t. overnight. All the volatiles were removed *in vacuo*, the oily residue was dissolved in $CH_2Cl_2$ (30 mL), washed with $H_2O$ (10 mL), sat. aq. $NaHCO_3$ sol. (10 mL), and brine (10 mL). The organic layer was dried over $MgSO_4$ and the salt was filtered off. The solvent was removed *in vacuo* and a further purification by means column chromatography (silica, $CH_2Cl_2$:MeOH = 99:1, gradient to 97:3) afforded the final product (tough clear oil, 74%). **$^1$H-NMR (400 MHz, $CDCl_3$):** δ = 8.23 (s, 1H), 7.66 – 7.59 (m, 2H), 7.36 – 7.29 (m, 2H), 4.66 (s, 2H), 3.50 – 3.41 (m, 4H), 2.33 (s broad, OH), 1.65 – 1.41 (m, 6H). See Figure S73. **$^{13}$C-NMR (101 MHz, $CDCl_3$):** δ = 154.10, 153.78, 144.47, 129.67, 128.33, 127.03, 64.66, 45.24, 25.70, 24.31. See Figure S74. **ESI$^+$ HRMS**: *m/z* = 263.1506 (calcd. 263.1390 for MH$^+$). See Figure S75.

## 2.1.11. Carbamoylaldoxime control initiator 14

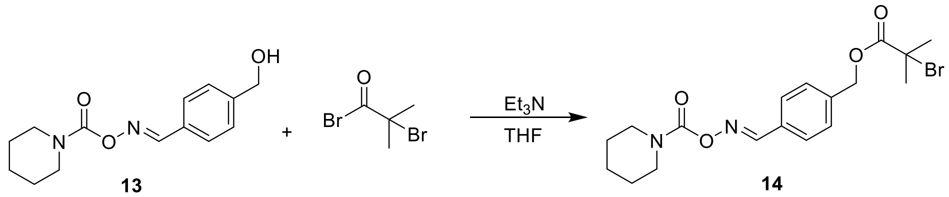


13 14

Carbamoylaldoxime control **13** (139 mg, 0.53 mmol, 1 equiv.) and dry $Et_3N$ (223 µL, 1.59 mmol, 3 equiv.) were dissolved in dry THF (5 mL) in a Schlenk flask under $N_2$ atmosphere. Subsequently, a solution of α-bromoisobutyryl bromide (196 µL, 1.59 mmol, 3 equiv.) in dry THF (1 mL) was added dropwise over 30 min at 0 °C. Then, the mixture was stirred at r.t. overnight. Afterwards, all the volatiles were removed *in vacuo*, the residue was dissolved in EtOAc (30 mL), washed with $H_2O$ (10 mL), sat. aq. $NaHCO_3$ sol. (10 mL), and brine (10 mL). The organic layer was dried over $MgSO_4$ and the salt was filtered off. The solvent was removed *in vacuo* and a further purification by column chromatography (silica, EtOAc:heptane = 1:1) afforded the final product (white ductile solid, 87%). **$^1$H-NMR (400 MHz, $CDCl_3$):** δ = 8.25 (s, 1H), 7.72 – 7.64 (m, 2H), 7.39 – 7.32 (m, 2H), 5.16 (s, 2H), 3.49 – 3.42 (m, 4H), 1.89 (s, 6H), 1.58 – 1.50 (m, 6H). See Figure S76. **$^{13}$C-NMR (101 MHz, $CDCl_3$):** δ = 153.73, 153.62, 138.53, 130.66, 128.41, 128.04, 66.92, 45.26, 30.76, 25.65, 24.31. See Figure S77. **ESI$^+$ HRMS**: *m/z* = 413.1047 (calcd. 413.0896 for MH$^+$ of $^{81}$Br). See Figure S78.

## 2.1.12. Control initiator **15**

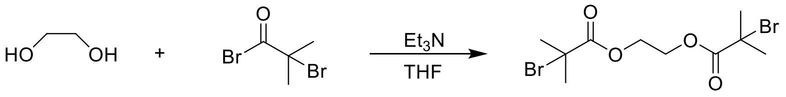


**15**

Ethylene glycol (900 µL, 16.11 mmol, 1 equiv.) and dry $Et_3N$ (4.65 mL, 32.22 mmol, 2 equiv.) were dissolved in dry THF (80 mL) in a Schlenk flask under $N_2$ atmosphere. Subsequently, a solution of α-bromoisobutyryl bromide (3.89 mL, 32.22 mmol, 2 equiv.) in dry THF (20 mL) was added dropwise over 30 min at 0 °C. Then, the mixture was stirred at r.t. overnight. Afterwards, all the volatiles were removed *in vacuo*, the residue was dissolved in EtOAc (100 mL), washed with $H_2O$ (30 mL), sat. aq. $NaHCO_3$ sol. (30 mL), and brine (30 mL). The organic layer was dried over $MgSO_4$ and the salt was filtered off. The solvent was removed *in vacuo* and a further purification by column chromatography (silica, EtOAc:heptane = 1:1) afforded the final product (white ductile solid, 38%). **$^{1}$H-NMR (300 MHz, $CDCl_3$):** δ = 4.37 (s, 4H), 1.87 (s, 12H). $^{13}$C NMR (75 MHz, $CDCl_3$) δ 171.44, 63.20, 30.71. See Figure S79. **$^{13}$C-NMR (75 MHz, $CDCl_3$):** δ = 171.44, 63.20, 30.71. See Figure S80.

## 2.1.13. Catechol carbonate **16**

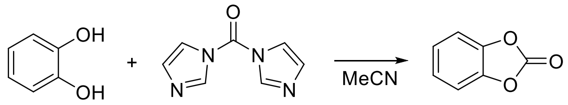


**16**

Pyrocatechol (880 mg, 8.0 mmol, 1 equiv.) and carbonyildiimidazole (1.29 g, 8.0 mmol, 1 equiv.) were dissolved in dry MeCN (15 mL) in a Schlenk flask under $N_2$ atmosphere. The mixture was stirred and refluxed for 24 h. Afterwards, all the volatiles were removed *in vacuo*, the residue was dissolved in $CH_2Cl_2$ (40 mL) and washed with $H_2O$ (20 mL) and brine (20 mL). The parent aqueous layers were collected and extracted with more $CH_2Cl_2$ (30 mL). The organic layers were collected, dried over $MgSO_4$ and the salt was filtered off. The solvent was removed *in vacuo* and the final product was obtained without further purification (white solid, 78%). **$^{1}$H-NMR (400 MHz, $CDCl_3$):** δ = 7.23 – 7.12 (m, 4H). See Figure S81. **$^{13}$C-NMR (101 MHz, $CDCl_3$):** δ = 151.25, 143.25, 124.91, 110.48. See Figure S82.

## 2.2. Polymer synthesis

### 2.2.1. $Cu^0$-wire activation

A large amount of $Cu^0$-wire was completely immersed in conc. HCl and left for approximately 20 min. Then, the wire was extensively washed with acetone and carefully dried *in vacuo*. The activated $Cu^0$-wire was stored in a sealed Schlenk flask under Argon atmosphere.

### 2.2.2. General procedure for $Cu^0$-mediated CRP with methyl acrylate (MA)

The selected initiator was dissolved in a small amount of DMSO and different stock solutions of $CuBr_2$ and $Me_6TREN$ in DMSO, previously prepared, were collected; in parallel, the inhibitor was removed from the monomer by means a short column of neutral $Al_2O_3$. Then, in a Schlenk tube with proper size, the initiator solution, two aliquots of respectively $CuBr_2$ stock solution and $Me_6TREN$ stock solution, and the monomer, were mixed, eventually adding an amount of DMSO necessary to reach a total volume of this latter equal to the volume of the monomer. The mixture was degassed by three freeze-pump-thaw cycles, after which the stir bar, having the activated $Cu^0$-wire (approximately 5 cm) wrapped around it, was added to the Schlenk tube and kept anchored to the glass wall through an external magnet; subsequently, three more freeze-pump-thaw cycles were performed. Then, the stir bar with the $Cu^0$-wire was dropped into the degassed mixture, which was left stirring at r.t. for 6 h, observing the solution getting more viscous. Afterwards, the mixture was diluted in THF and filtered over neutral $Al_2O_3$. The solvent was removed *in vacuo* and the viscous residue was precipitated in cold MeOH (2×) to yield the final polymer. Eventually, the polymer was dialyzed against MeCN overnight.

### 2.2.3. Purification of hydroxyethyl acrylate

The monomer HEA was purified prior to use, to remove residues of diacrylate side product, according to literature.[2] A monomer solution was diluted in $H_2O$ (20% v/v), extracted with heptane (10×50 mL); the aqueous layer was salted with NaCl, to reach approximately 200 $g \cdot L^{-1}$, and extracted with $Et_2O$ (4×200 mL). Afterwards, Hydroquinone (0.05 wt%) was added to the organic layer, which was dried over $MgSO_4$ and the salt was filtered off. The solvent was removed *in vacuo* to yield the final pure liquid HEA, which was safely stored at 2-8 °C in the dark.

### 2.2.4. General procedure for $Cu^0$-mediated CRP with hydroxyethyl acrylate (HEA)

The procedure of the CRP with HEA to synthesize polymers with high molar masses was reproduced according to the literature.[2] The selected initiator was dissolved in a small amount of DMSO and a stock solution of $Me_6TREN$ in DMSO, previously prepared, was used. The inhibitor was removed from the purified monomer by means a distillation *in vacuo* (max. 65 °C at 0.001-0.010 mbar). Then, in a Schlenk tube with proper size, the initiator solution, an aliquot of $Me_6TREN$ stock solution, and the monomer, were mixed, eventually adding an amount of DMSO necessary to reach a total volume of twice the volume of the monomer. The mixture was degassed by three freeze-pump-thaw cycles, after which the stir bar, having the activated $Cu^0$-wire (approximately 5 cm) wrapped around it, was added to the Schlenk tube and kept anchored to the glass wall through an external magnet. Subsequently, three more freeze-pump-thaw cycles were performed. Then, the stir bar with the $Cu^0$-wire was dropped into the degassed mixture, which was left stirring at r.t. for 6 h, observing the solution getting more viscous. Afterwards, the mixture was precipitated in cold $Et_2O$, the residue was dissolved in $H_2O$, dialyzed overnight against $H_2O$, and freeze-dried to yield the final polymer.

## 2.2.5. Synthesis of PMA6$_{107}$

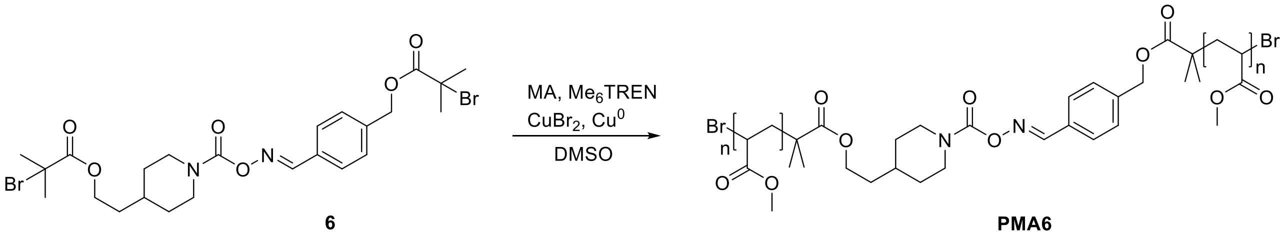


The synthesis was performed according to the general procedure (*cf.* Section 2.2.2). A degassed solution containing the carbamoylaldoxime mechanophore initiator **6** (15.1 mg, 0.025 mmol, 1.00 equiv.), MA monomer (2.26 mL, 25.00 mmol, 1000 equiv.), $CuBr_2$ (1.25 µmol, 0.05 equiv.), $Me_6TREN$ (3.75 µmol, 0.15 equiv.), and $Cu^0$-wire, in DMSO (2.26 mL) was stirred at r.t. for 6 h. After workup, the polymer was obtained. $M_n$ = 107.0 kDa and $Đ_M$ = 1.20 (Figure S84). NMR spectra (Figure S83) are reported with the zoomed aromatic region and it shows a low appearance of new aromatic peaks which are attributed to a small degree of thermal conversion to the nitrile product **PMA7** caused by local high temperature in the polymerization mixture due to the intrinsic exothermicity of the reaction (*cf.* section 2.3 for more information). For the same reason, the GPC spectra in Figure S84 presents a small shoulder at lower molar mass, attributed to the half-length polymer chain having the nitrile functionality as end group.

## 2.2.6. Synthesis of PMA6 with different molar masses

Following the same procedure as for **PMA6$_{107}$**, but varying the equivalents of the monomer and keeping the Schlenk flask immerged in a $H_2O$ bath to maintain thermostatic conditions, multiple polymers with different molar masses were obtained (**PMA6$_{80}$**, **PMA6$_{90}$**, **PMA6$_{145}$**, **PMA6$_{134}$**, **PMA6$_{116}$**, **PMA6$_{50}$**), observing a generally lower conversion compared to **PMA6$_{107}$**.Table S1 summarizes the results, with reference to the GPC and NMR analysis Figures of every polymer. Every NMR spectrum is reported with the zoomed aromatic region to evidence the structural integrity of the mechanophore initiator. For all the polymers prepared in thermostatic conditions, no appearance of new aromatic peaks was observed.

## 2.2.7. Synthesis of PHEA6$_{44}$

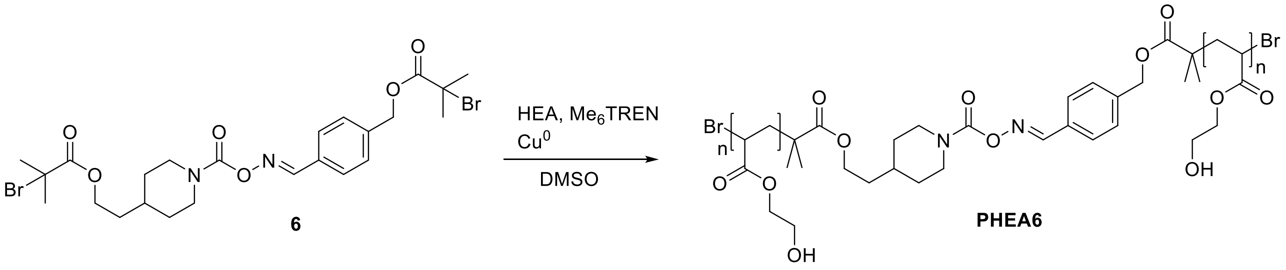


The synthesis was performed according to the general procedure (*cf.* Section 2.2.4). A degassed solution containing the carbamoylaldoxime mechanophore initiator **6** (9.7 mg, 0.016 mmol, 1.00 equiv.), HEA monomer (504 µL, 4.80 mmol, 300 equiv.), $Me_6TREN$ (8.0 µmol, 0.5 equiv.), and $Cu^0$-wire, in DMSO (1 mL) was stirred at r.t. for 6 h. After workup, the polymer was obtained. $M_n$ = 43.9 kDa and $Đ_M$ = 1.30 (Figure S96). NMR spectrum (Figure S95) is reported with the zoomed aromatic region to show the integrity of the mechanophore initiator.

## 2.2.8. Chain-terminal control PMA14

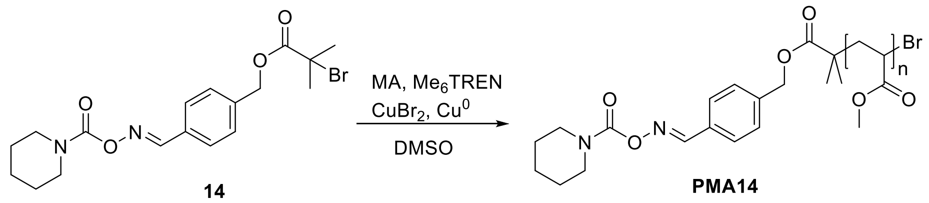


The synthesis was performed according to the general procedure (*cf.* Section 2.2.2). A degassed solution containing the carbamoylaldoxime control initiator **14** (5.1 mg, 0.0125 mmol, 1.00 equiv.), MA monomer (1.13 mL, 12.50 mmol, 1000 equiv.), $CuBr_2$ (0.63 µmol, 0.05 equiv.), $Me_6TREN$ (1.88 µmol, 0.15 equiv.), and $Cu^0$-wire in DMSO (1.13 mL) was stirred at r.t. for 6 h. After workup, the polymer was obtained. $M_n$ = 58.7 kDa and $Đ_M$ = 1.14 (Figure S98). NMR spectrum (Figure S97) is reported with the zoomed aromatic region to show the integrity of the control initiator. A low appearance of new aromatic peaks which are attributed to a small degree of thermal conversion to the nitrile product, most likely caused by local elevated temperature in the polymerization mixture due to the intrinsic exothermicity of the reaction.

## 2.2.9. Chain-terminal control PHEA14

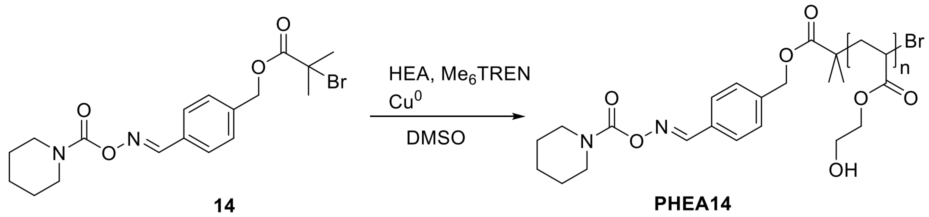


The synthesis was performed according to the general procedure (*cf.* Section 2.2.4). A degassed solution containing the carbamoylaldoxime control initiator **14** (10.3 mg, 0.025 mmol, 1.00 equiv.), HEA monomer (2.10 mL, 20 mmol, 800 equiv.), $Me_6TREN$ (0.0125 mmol, 0.5 equiv.), and $Cu^0$-wire in DMSO (4 mL), was stirred at r.t. for 6 h. After workup, the polymer was obtained. $M_n$ = 95.1 kDa and $Đ_M$ = 1.56 (Figure S100). NMR spectrum (Figure S99) is reported with the zoomed aromatic region to show the integrity of the control initiator.

## 2.2.10. Nitrile control PMA7

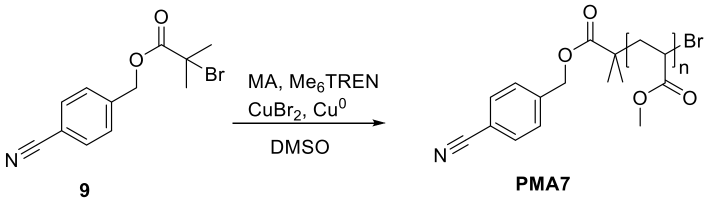


The synthesis was performed according to the general procedure (*cf.* Section 2.2.2). A degassed solution containing the nitrile control Initiator **9** (7.1 mg, 0.0250 mmol, 1.00 equiv.), MA monomer (1.81 mL, 20.00 mmol, 800 equiv.), $CuBr_2$ (1.25 µmol, 0.05 equiv.), $Me_6TREN$ (3.75 µmol, 0.15 equiv.), and $Cu^0$-wire in DMSO (1.81 mL) was stirred at r.t. for 6 h. After workup, the polymer was obtained. $M_n$ = 59.9 kDa and $Đ_M$ = 1.09 (Figure S102). NMR spectrum (Figure S101) is reported with the zoomed aromatic region to show the integrity of the control initiator.

## 2.2.11. Nitrile control **PHEA7**

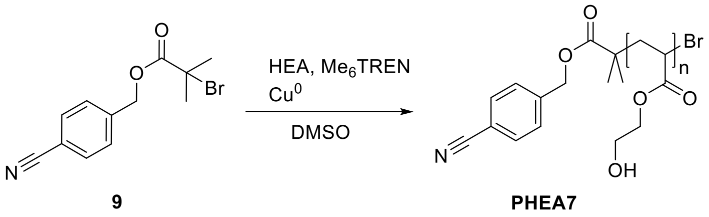


The synthesis was performed according to the general procedure (*cf.* Section 2.2.4). A degassed solution containing the nitrile control initiator **9** (14.1 mg, 0.050 mmol, 1.00 equiv.), HEA monomer (2.10 mL, 20 mmol, 400 equiv.), $Me_6TREN$ (0.025 mmol, 0.5 equiv.) and $Cu^0$-wire in DMSO (4 mL), was stirred at r.t. for 6 h. After workup, the polymer was obtained. $M_n$ = 67.1 kDa and $Đ_M$ = 1.37 (Figure S104). NMR spectrum (Figure S103) is reported with the zoomed aromatic region to show the integrity of the reference initiator.

## 2.2.12. Aldehyde control **PMA8**

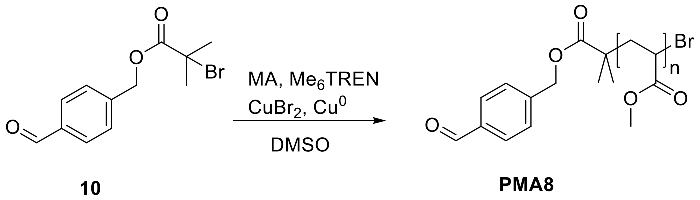


The synthesis was performed according to the general procedure (*cf.* Section 2.2.2). A degassed solution containing the aldehyde control initiator **10** (7.1 mg, 0.0250 mmol, 1.00 equiv.), MA monomer (1.81 mL, 20.00 mmol, 800 equiv.), $CuBr_2$ (1.25 µmol, 0.05 equiv.), $Me_6TREN$ (3.75 µmol, 0.15 equiv.), and $Cu^0$-wire in DMSO (1.81 mL) was stirred at r.t. for 6 h. After workup, the polymer was obtained. $M_n$ = 73.2 kDa and $Đ_M$ = 1.19 (Figure S106). NMR spectrum (Figure S105) is reported with the zoomed aromatic region to show the integrity of the control initiator.

## 2.2.13. Aldehyde control **PHEA8**

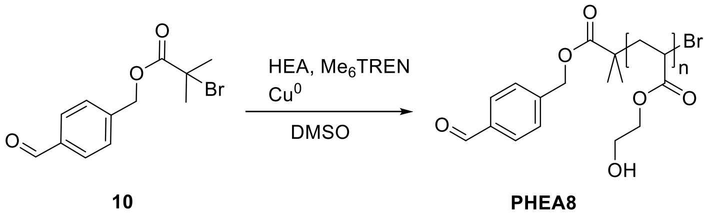


The synthesis was performed according to the general procedure (*cf.* Section 2.2.4). A degassed solution containing the aldehyde control initiator **10** (14.3 mg, 0.050 mmol, 1.00 equiv.), HEA monomer (2.10 mL, 20 mmol, 400 equiv.), $Me_6TREN$ (0.025 mmol, 0.5 equiv.), and $Cu^0$-wire in DMSO (4 mL), was stirred at r.t. for 6 h. After workup, the polymer was obtained. $M_n$ = 70.3 kDa and $Đ_M$ = 1.51 (Figure S108). NMR spectrum (Figure S107) is reported with the zoomed aromatic region to show the integrity of the reference initiator.

## 2.2.14. PMA control polymers **PMA**

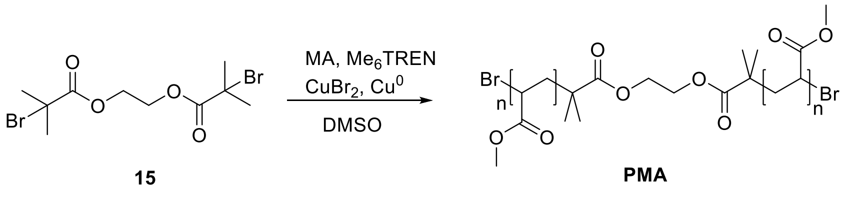

The synthesis was performed according to the general procedure (*cf.* Section 2.2.2). Referring to the final polymer named **PMA$_{94}$**, a degassed solution containing the control initiator **15** (9.0 mg, 0.025 mmol, 1.00 equiv.), MA monomer (2.83 mL, 12.50 mmol, 1000 equiv.), $CuBr_2$ (1.25 µmol, 0.05 equiv.), $Me_6$TREN (3.75 µmol, 0.15 equiv.), and $Cu^0$-wire in DMSO (2.83 mL) was stirred at r.t. for 6 h. After workup, the polymer was obtained. $M_n$ = 94.1 kDa and $Đ_M$ = 1.13 (Figure S109). The procedure was repeated multiple times and different additional control polymers were obtained (**PMA$_{139}$**, **PMA$_{119}$**, **PMA$_{42}$**). The results are summarized in Table S1.

**Table S1.** Summary of the prepared polymers with references to the related GPC and NMR spectra.

| Polymer | MA/HEA equiv. used | $M_n$ | $Đ_M$ | GPC | $^1$H-NMR |
|---|---|---|---|---|---|
| **PMA6$_{107}$** | 1000 | 107.0 | 1.20 | Figure S84 | Figure S83 |
| **PMA6$_{80}$** | 1200 | 80.4 | 1.20 | Figure S86 | Figure S85 |
| **PMA6$_{90}$** | 1200 | 90.9 | 1.20 | Figure S88 | Figure S87 |
| **PMA6$_{145}$** | 1800 | 145.0 | 1.21 | Figure S90 | Figure S89 |
| **PMA6$_{134}$** | 1500 | 134.3 | 1.17 | Figure S92 | Figure S91 |
| **PMA6$_{116}$** | 1200 | 115.6 | 1.12 | Figure S94 | Figure S93 |
| **PHEA6$_{44}$** | 300 | 43.9 | 1.30 | Figure S96 | Figure S95 |
| **PMA14** | 1000 | 58.7 | 1.14 | Figure S98 | Figure S97 |
| **PHEA14** | 800 | 95.1 | 1.56 | Figure S100 | Figure S99 |
| **PMA7** | 800 | 59.9 | 1.09 | Figure S102 | Figure S101 |
| **PHEA7** | 400 | 67.1 | 1.37 | Figure S104 | Figure S103 |
| **PMA8** | 800 | 73.2 | 1.19 | Figure S106 | Figure S105 |
| **PHEA8** | 400 | 70.3 | 1.51 | Figure S108 | Figure S107 |
| **PMA$_{94}$** | 1000 | 94.1 | 1.13 | Figure S109 | - |
| **PMA$_{139}$** | 2000 | 139.1 | 1.20 | Figure S110 | - |
| **PMA$_{119}$** | 1800 | 119.0 | 1.16 | Figure S111 | - |
| **PMA$_{42}$** | 500 | 42.0 | 1.08 | Figure S112 | - |

## 2.3. Thermal stability experiments

According to the literature,[3,4] different oxime derivatives show also a partial thermal responsiveness, whose kinetics is normally much slower than for the photochemical pathway, and very high temperature are necessary to achieve high conversions. Therefore, the thermal sensitivity at elevated temperature and stability over long-term storage was investigated for the carbamoylaldoxime motif in question.

The initiator **6**, after approximately 8 months of storage at 2-8 °C, was analyzed by $^1$H-NMR (Figure S1), which does not show any conversion or degradation of the aromatic region, confirming its stability in these storage conditions over long term. Similarly, the integrity of the structure was also evaluated incorporated in the middle of linear polymer chains. Therefore, both **PMA6** and **PHEA6** were analyzed by $^1$H-NMR spectroscopy (Figure S2 and Figure S3), showing that they are both stable after 4 months of storage at 2-8 °C, and also after 4 d in DMSO-$d_6$ at r.t. exposed to visible light. In Figure S2, residues of solvents are present, DMF at δ = 7.95 ppm, and $CHCl_3$ at δ = 8.32 ppm.

Then, the thermal responsiveness at higher temperatures was evaluated for the molecule **5**. An NMR sample of this latter in DMSO-$d_6$ was left at 70 °C for 6 h. Thereafter, the $^1$H-NMR spectrum was recorded (Figure S4) showing a low appearance of the peaks related to the nitrile **7**. However, their intensity suggests that this thermal process occurs with very slow kinetics, and very high temperature are required. Furthermore, concerning the synthesis of **5** and its photochemical experiments reported in the related Section 2.4, the removal of the solvents *in vacuo* (THF and DMF), always done at a maximum of 50 °C for maximum 1 h, was never characterized by some conversion of the molecule to the related nitrile. Generally, complete stability was confirmed by consulting the corresponding $^1$H-NMR spectra after staying in the hot $H_2O$ bath during the evaporation process. However, as already mentioned in Section 2.2, different batches of this latter were made keeping the mixture in a $H_2O$ bath to maintain thermostatic conditions to avoid possible local exothermic heating, which realistically affected the purity of the isolated **PMA6$_{107}$**.

An extreme study of the thermal responsiveness of **5** was performed by simply submitting it to a GC-MS analysis, whose conditions of vaporization are reported to reach the temperature of approximately 250 °C. As shown in Figure S5, the final chromatogram does not show any residual **5**, but only the products amine **1** and nitrile **7**, demonstrating that the thermal pathway at extreme conditions affords the same products as the photochemical one.

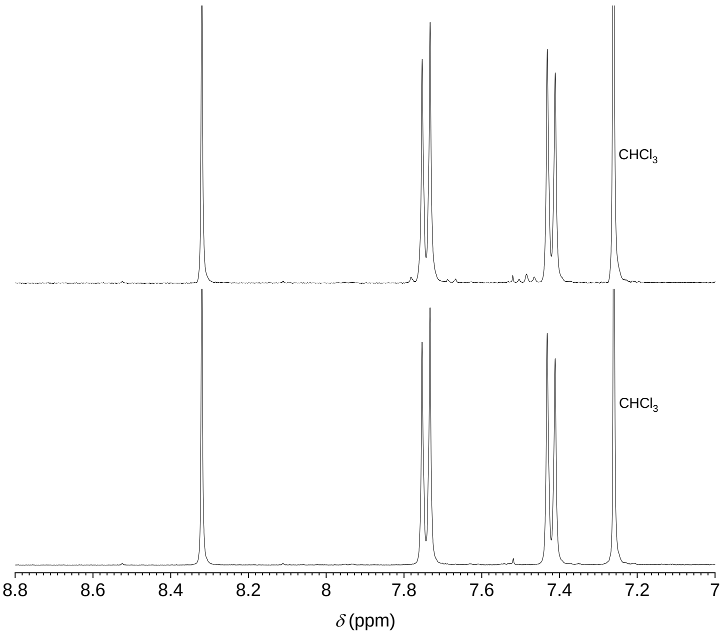


**Figure S1.** $^1$H-NMR spectra comparison of **6**, in $CDCl_3$, zoomed in between δ = 8.8 and 7.0 ppm. From top to bottom: freshly prepared, after 8 months of storage at 2-8°C.

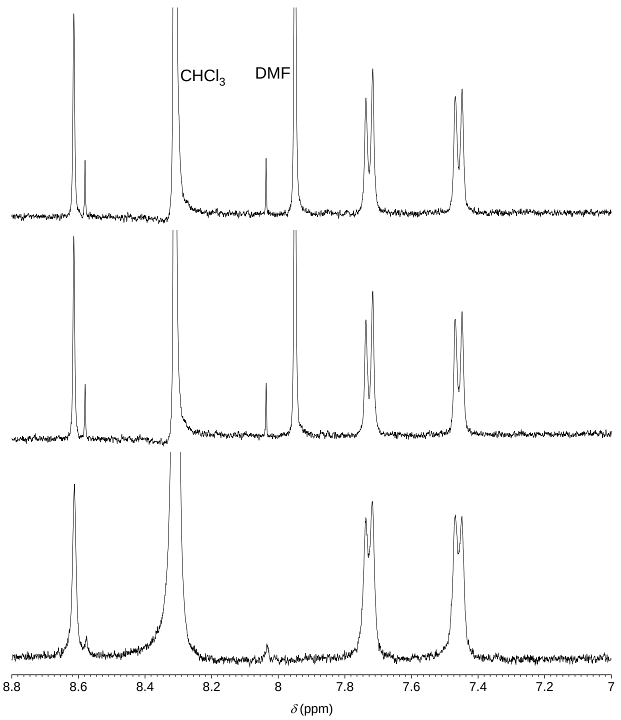


**Figure S2.** $^{1}$H-NMR spectra comparison of **PMA6$_{80}$** in DMSO-$d_6$ zoomed in between δ = 8.8 and 7.0 ppm. From top to bottom: freshly prepared, after 4 months of storage at 2-8 °C, after 4 d in DMSO-$d_6$ at r.t.

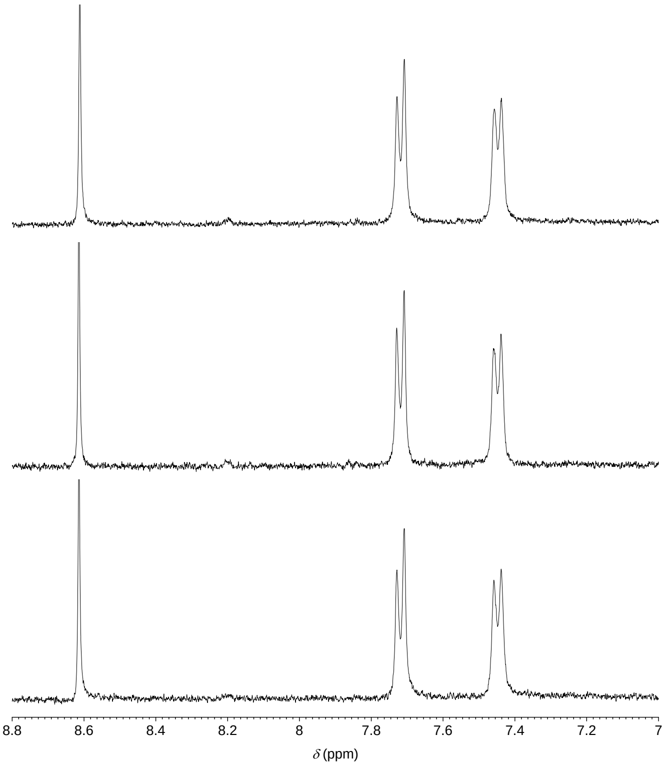


**Figure S3.** $^{1}$H-NMR spectra comparison of **PHEA6$_{44}$** in DMSO-$d_6$ zoomed in between δ = 8.8 and 7.0 ppm. From top to bottom: freshly prepared, after 4 months of storage at 2-8 °C, after 4 d in DMSO-$d_6$ at r.t.

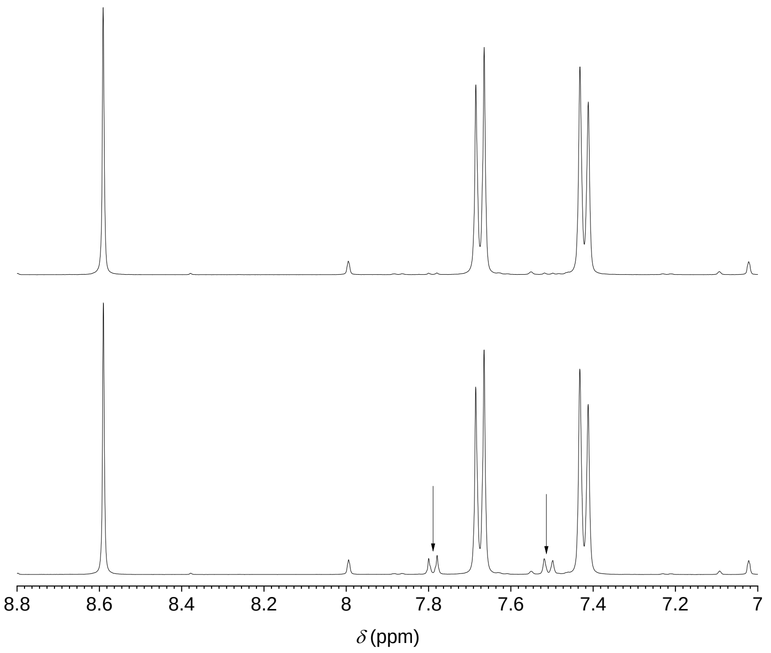

**Figure S4.** $^{1}$H-NMR spectra comparison of **5**, heated at 70 °C for 6 h in DMSO-$d_6$ zoomed in between δ = 8.8 and 7.0 ppm. From top to bottom: starting sample, after heating. The arrows indicated the aromatic peaks of nitrile **7**, which are emerging at low intensity.

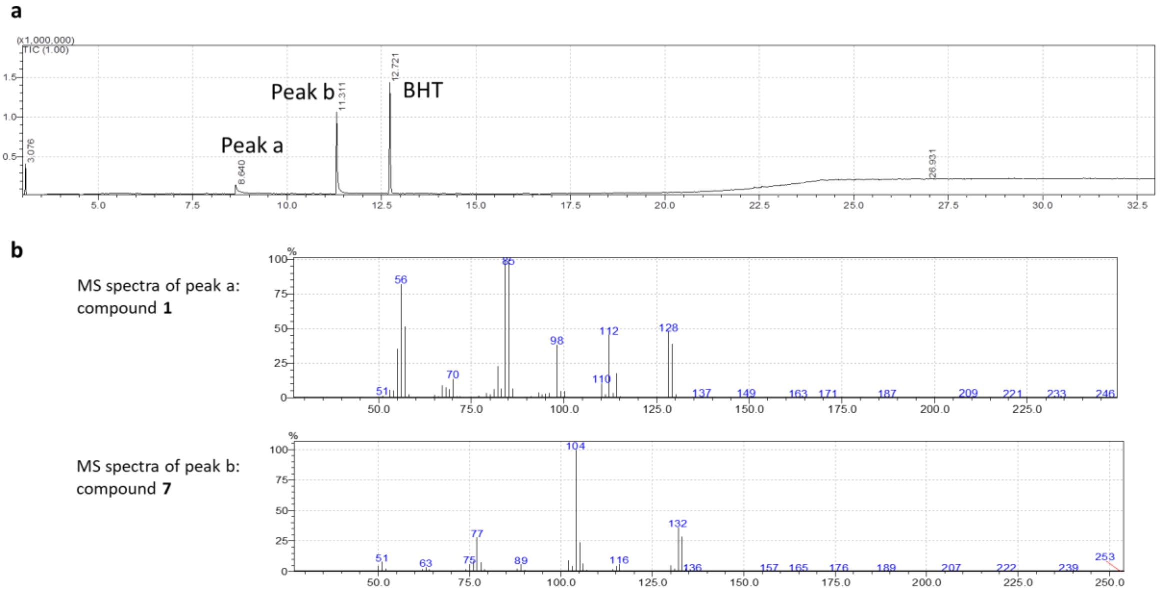


**Figure S5.** (a) GC chromatogram of **5**. (b) EI MS spectra of the fractions of peaks a and b, assigned to respectively **1** and **7**.

## 2.4. Photochemical experiments

### 2.4.1. The mechanism

The excitation of the oxime derivatives triggers a photoinduced homolytic scission of the oxime N-O bond, generating an iminyl radical on one side, and another radical, which depends on the compound subclass, on the other side. The iminyl radical can undergo three different pathways, depending on the structure and on the external conditions:

i. the hydrogen radical abstraction, to give the imine functional group,
ii. the cyclization through 5-*exo* or 6-*endo*, if a double bond is present in the structure, and
iii. the dissociation to give the nitrile functional group, which mostly happens when the starting oxime presents an aldehyde-like structure (aldoxime),[5] therefore reducing the lifetime of the iminyl radical, that becomes less prone to undergo cyclization or H-abstraction, which are instead more favored when the oxime presents a ketone-like structure (ketoxime).

The nature of the other radical will determine the final product on the other side, depending on the compound subclass. Among different ones accurately summarized in literature,[3] the most significant ones which have been studies are the oxime esters, oxime ethers, and oxime carbamates (or carbamoyloximes), which is the one that is subject of this work. The detailed mechanism of the photochemical decomposition of these carbamoyloximes concerns the formation of the iminyl radical and of the carbamoyloxyl radical. This latter is known to be extremely unstable and prone to undergo an immediate decarboxylation,[6] as shown, to give the aminyl radical. This, in turn, exhibits longer lifetimes, but as soon as it can abstract an hydrogen radical from its surrounding, it affords the final amine.[7]

**Table S2.** List of photochemical experiments, with related parameters, conditions, and analytic techniques performed.

| entry | compound no. | irradiation method | irradiation time / min | *ρ* / mg·mL$^{-1}$ or *c* / mM | *V* / mL | solvent | analyses and Figures |
|---|---|---|---|---|---|---|---|
| 1 | **5** | low intensity | 60 | 0.05 mM | 3×4 | THF | NMR (Figure S9) and UV-vis (Figure S6) |
| 2 | **5** | low intensity | 60 | 0.05 mM | 3×4 | $H_2O$ | NMR (Figure S9) and UV-vis (Figure S7) |
| 3 | **5** | low intensity | 240 | 1.0 mg·mL$^{-1}$ (3.26 mM) | 1.5 | THF | ESI-MS (Figure S11) |
| 4 | **5** | high intensity | 30 | 1.0 mg·mL$^{-1}$ | 3 | THF | NMR after derivatization with **16** (Figure S13) |
| 5 | **5** | low intensity | 60 | 0.05 mM | 3 | DMF | UV-vis (Figure S8) |
| 6 | **5** | high intensity | 20 | 1.0 mg·mL$^{-1}$ | 1 | DMF | NMR (Figure S10) |
| 7 | **14** | high intensity | 60 | 1.0 mg·mL$^{-1}$ | 3 | DMF | NMR after derivatization with **16** (Figure S14) |
| 8 | **PMA6$_{107}$** | low intensity | 60 | 5.0 mg·mL$^{-1}$ | 3×4 | THF | NMR (Figure S17) and UV-vis (Figure S15) |
| 9 | **PMA6$_{80}$** | high intensity | 20 | 1.0 mg·mL$^{-1}$ | 5 | THF | GPC after reaction with **RhBITC** (Figure 2c) |
| 10 | **PMA6$_{145}$** | high intensity | 60 | 45 mg·mL$^{-1}$ | 2 | DMF | NMR (Figure 1c and Figure S30) |
| 11 | **PHEA6$_{44}$** | low intensity | 60 | 2.5 mg·mL$^{-1}$ | 3×4 | $H_2O$ | NMR (Figure 1d and Figure S31) and UV-vis (Figure S16) |

### 2.4.2. General procedure and list of experiments

The experiments were carried out on different structures synthetized and in different conditions, moreover, different analytic techniques were used depending on the purpose of the specific photochemical experiment. Therefore, multiple entries were created, and these are summarized in Table S2. Two different light sources (*cf.* Section 1.1 for details) were used for different entries, depending on the concentration of the sample and on the intrinsic kinetics of the compound for the photochemical reaction. When high concentration was required and/or the kinetics were specifically slow, the high intensity UV source was used. On the other hand, the low intensity UV source was always used when the concentration was in UV-vis spectroscopy range. All experiments were performed in a Quartz cuvette. In case of the low intensity source (coupled with a spectrometer), the cuvette was kept in thermostatic conditions at 25 °C and equipped with a stir bar, so that the solution was constantly stirred during the whole photochemical experiments. In case of the high intensity source, the cuvette was placed below the beam source (at a distance of 5 cm), the system was properly isolated in the dark, and the irradiation was performed without magnetic stirring and without thermostatic conditions. To aid the reaction, the mixture in the cuvette was manually mixed at regular intervals and the temperature of the solution was measured and verified to never exceed a maximum of 31 °C after 60 min of irradiation. This temperature effect could be neglected in terms of ability to compete for the photochemical conversion of the molecule in question.

### 2.4.3. Small molecules: the iminyl pathway

The UV-vis absorption spectra of **5** were recorded during irradiation of the sample in: THF (Entry 1 of Table S2; UV-vis absorption spectra in Figure S6), $H_2O$ (Entry 2 of Table S2; UV-vis absorption spectra in Figure S7), and DMF (Entry 5 of Table S2; UV-vis absorption spectra in Figure S8). During the irradiation, the general trend is in agreement with previous studied reported in the literature with similar structures.[8] Either in THF or $H_2O$, the main peak at 262 nm is decreasing, while the shoulder on the left, at around 230 nm, is increasing in both solvents. The shoulder at higher wavelengths (around 310 nm) is significantly increasing in $H_2O$, while only slightly in THF. Furthermore, looking at the ratio between the main band and the shoulders, the kinetics in $H_2O$ appear to be faster than in THF, considering the starting identical conditions. To conclude, the irradiation was also performed in DMF, but since DMF also absorbs UV light at around 254 nm, no significant change of the spectra (the region of the shoulder at higher wavelengths was monitored) was observed. This may well be due to the lack of absorbed photons by the molecule in question, because of the low intensity light source, thus low density of irradiation, and low concentration of the sample.

The exact photochemical outcomes of **5** for the pathway of the iminyl radical intermediate was semi-quantitatively investigated through a detailed $^1$H-NMR analysis of the aromatic region. First, the study was performed in THF and $H_2O$, reproducing the same irradiation conditions used for the UV-vis absorption monitoring (respectively Entry 1 and Entry 2 of Table S2). In this case, for each entry three batches of samples were collected for a total of approximately 9 mL. The solvent was then removed *in vacuo* and the residue was dissolved in DMSO-$d_6$ for $^1$H-NMR analysis, which was ran over 128 scans given the low amount of the sample and the necessity to have an acceptable resolution for the semi-quantitative survey. The results are summarized in Figure S9. The starting molecule **5** has its set of aromatic peaks at δ = 8.59, 7.66, and 7.43 ppm (i, g, and h in Figure S9b), but after irradiation in THF we recognize only one new set of aromatic peaks at δ = 7.78 and 7.52 ppm (g' and h' in Figure S9b), which are attributed to the nitrile compound **7**. The result is very similar when the irradiation is carried out in $H_2O$, where the nitrile **7** is the main product obtained. The only difference is that there is another set of new aromatic peaks at δ = 7.58, 7.49 and 7.28 ppm (indicated as black dots in Figure S9b), whose structural elucidation remains unknown. A possibility is that they are related to a product derived from a diradical coupling between two intermediates, for instance the aminyl and iminyl radicals, as already reported in literature for similar classes of oxime derivatives.[3,8] Concerning the intensity of the peaks, we can observe that in case of irradiation in $H_2O$ more starting molecule **5** has been converted, which is in agreement with the observation done for the UV-vis absorption results.

Regarding the irradiation of **5** in DMF, no response was observed at low concentration as commented above (Entry 5 of Table S2; UV-vis absorption spectra in in Figure S8). Therefore, the photochemical activity in this solvent was tested in different conditions using a high intensity light source and increasing the concentration of the sample (Entry 6 of Table S2). After the irradiation, the sample was collected and the solvent was removed *in vacuo*. The residue was dissolved in $CDCl_3$ for $^1$H-NMR analysis, the results are summarized in Figure S10. The starting molecule **5** has its aromatic peaks at δ = 8.32, 7.72, and 7.42 ppm (i, g, and h in Figure S10b), but after irradiation the partial appearance of new aromatic peaks at δ = 7.64 and 7.49 ppm (g' and h' in Figure S10b) is observed, which are assigned to the nitrile **7**. Residual $CHCl_3$ is present at δ = 7.26 ppm, while residues of DMF can also be identified at δ = 8.02 ppm. To conclude, we were able to verify that, when the intensity of irradiation and the concentration of the sample are high enough to permit the molecules of **5** to absorb photons, these can undergo photochemical decomposition in DMF in the same way as they do in THF and $H_2O$. Here, the nitrile **7** is also the only aromatic product observed.

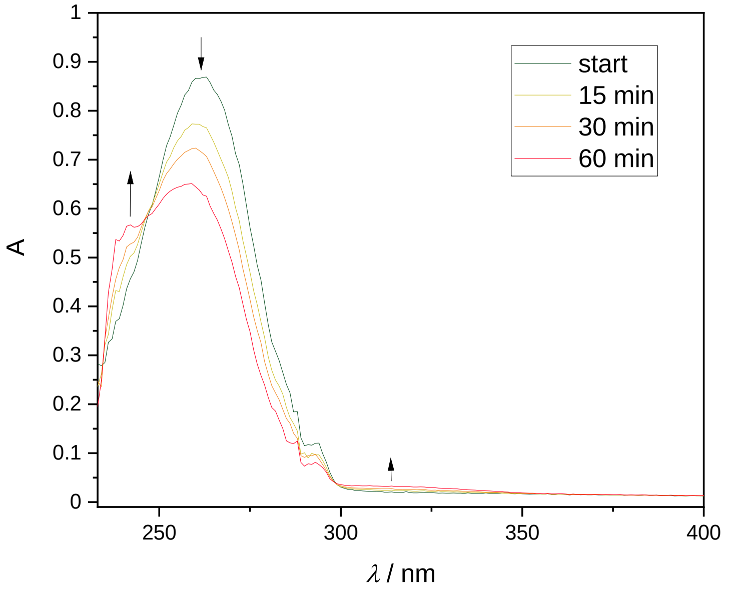


**Figure S6.** UV-vis absorption spectra of **5** in THF (Entry 1 of Table S2), at different irradiation times.

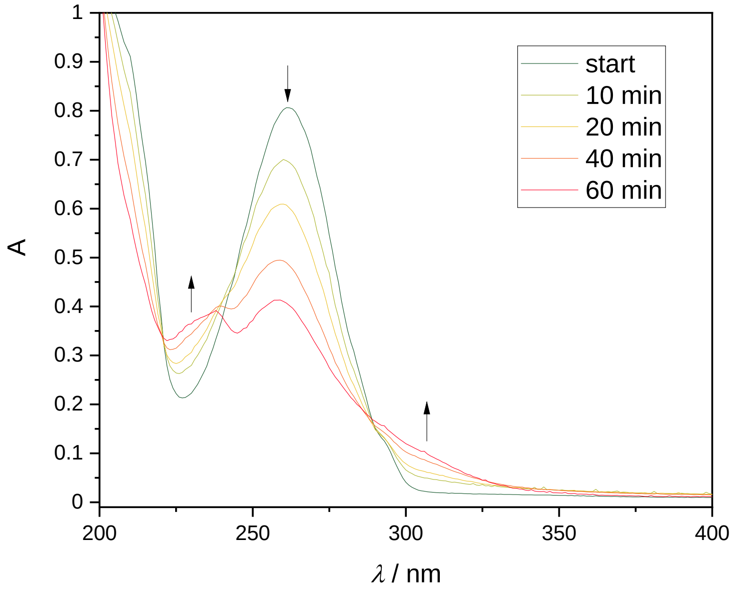


**Figure S7.** UV-vis absorption spectra of **5** in $H_2O$ (Entry 2 of Table S2), at different irradiation times.

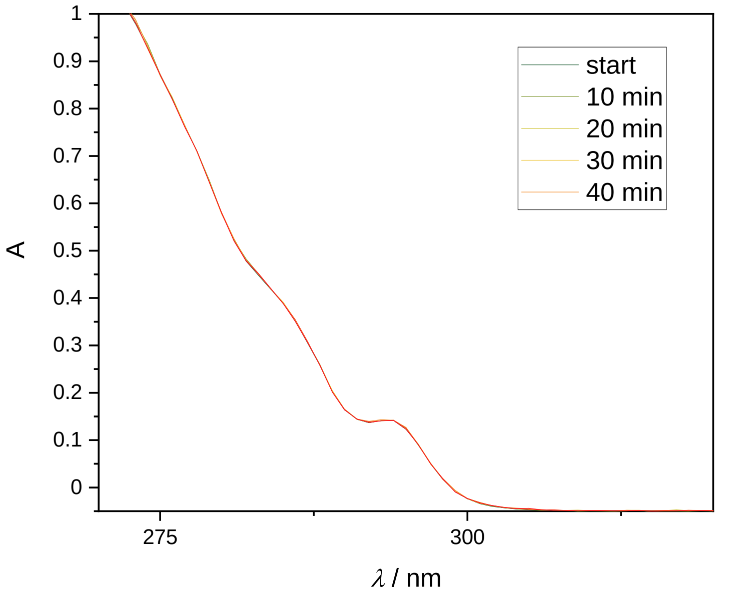


**Figure S8.** UV-vis absorption spectra of **5** in DMF (Entry 5 of Table S2), at different irradiation times.

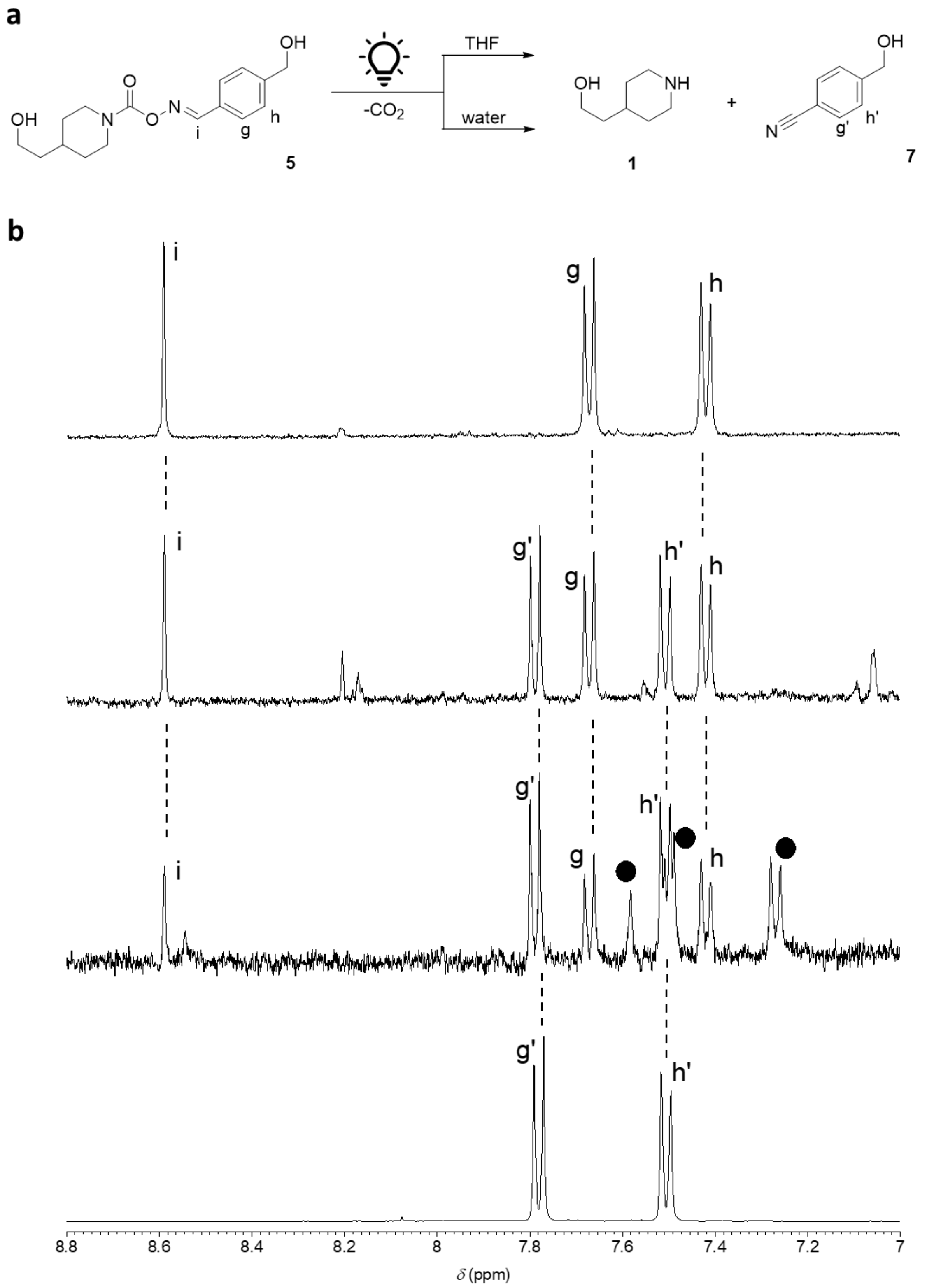


**Figure S9.** (a) Summary of the irradiation outcomes of **5**. Either in THF (Entry 1 of Table S2) and $H_2O$ (Entry 2 of Table S2) the same nitrile product **7** is obtained. The amine **1** is derived from the aminyl radical intermediate and it is not subject of this NMR analysis (identified by ESI-MS and chemical derivatization, *cf.* Section 2.4.4). (b) Comparison of $^1H$-NMR spectra in DMSO-$d_6$ zoomed between 8.8 and 7.0 ppm. From top to bottom: starting molecule **5**, irradiated **5** in THF (Entry 1 of Table S2), irradiated **5** in $H_2O$ (Entry 2 of Table S2), control molecule **7**.

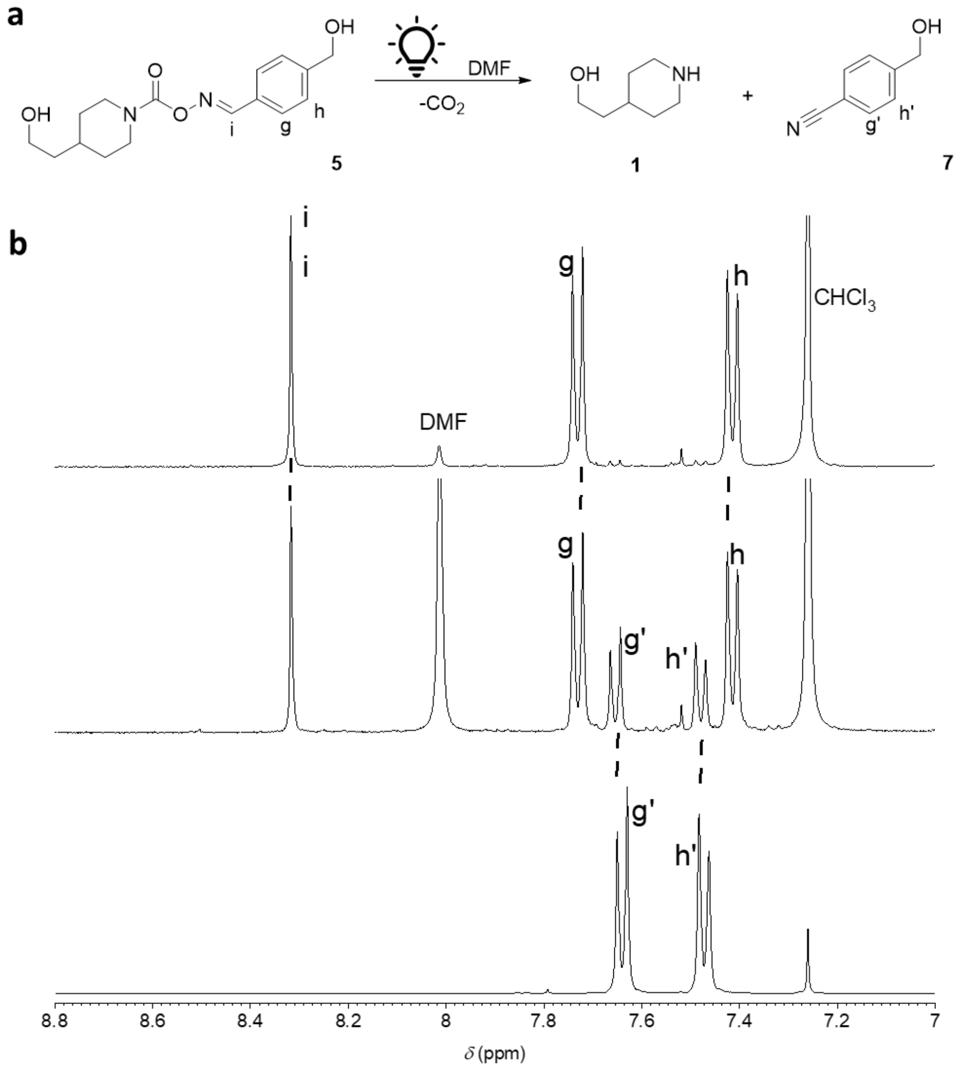


**Figure S10.** (a) Summary of the irradiation outcomes of **5** in DMF (Entry 6 of Table S2), giving the nitrile **7** as only product. The amine **1** is derived from the aminyl radical intermediate and it is not subject of this NMR analysis (identified by chemical derivatization, *cf.* Section 2.4.4). (b) Comparison of $^1$H-NMR spectra in $CDCl_3$ zoomed between δ = 8.8 and 7.0 ppm. From top to bottom: starting molecule **5**, irradiated **5** in DMF (Entry 6 of Table S2), control molecule **7**.

## 2.4.4. Small molecules: amine detection

### 2.4.4.1. ESI$^+$ HRMS analysis

First, the amine was detected by ESI$^+$ HRMS analysis. The irradiated sample (Entry 3 of Table S2) was collected and the solvent (THF) was removed *in vacuo*. The residue was re-dissolved in MeCN for the final preparation required for the MS analysis. ESI$^+$ HRMS was performed, and results are reported in Figure S11. This analysis can only be used for a qualitative and semi-quantitative evaluation of the amine formation, but no exact quantitative information can be extracted. Figure S11a shows the spectrum of only **1** as control, while Figure S11b shows the spectrum of pristine **5**. Figure S11c is the spectrum of the irradiated sample, where we can clearly see a significant increasing of the peak at $m/z$ = 130.12, which is attributed to the MH$^+$ of the compound **1**. The intensity of the latter, after irradiation, is approximately 6 times lower than the peak of the reference (considering the same initial concentration in mg·mL$^{-1}$). However, the information is only semi-quantitative. Moreover, it must be taken into account that the photochemical conversion is only partial, besides that the starting equal concentration in mg·mL$^{-1}$ means that the final molarity of the amine is lower, due to the lower molar mass.

### 2.4.4.2. $^1$H-NMR after derivatization with cyclic carbonate 16

This approach was chosen given the difficult interpretation of direct $^1$H- and $^{15}$N-NMR of the produced amine in low concentrations. The $^1$H-NMR result of a first test reaction carried out directly in $CDCl_3$ (0.5 mL), between piperidine (1 mM) and the carbonate **16** (10 mM), is reported in Figure S12. The NMR spectra clearly show the diagnostic upfield shift: before the addition of the piperidine, the merged peaks of the four protons of **16** are only present at δ = 7.30-7.20 ppm. After the addition of the amine, the pattern of the final adduct piperdine-**16** can be clearly recognized at δ = 7.09, 7.02, and 6.91 ppm (o, o', o'', and o''' in Figure S12b). The spectra also present side peaks attributed to degradation products of **16** deriving from hydrolysis/oxidation processes (black dots in Figure S12b). Therefore, it must be considered that this molecule **16** requires storage at -20 °C and under dry and inert atmosphere. Its high reactivity is reflected in high tendency to hydrolyze to the corresponding catechol, which is then prone to oxidize/polymerize in presence of oxygen and at higher temperatures, giving rise to a mixture of broad aromatic signals. These can be already partially observed in the spectra in Figure S12b.

The chemical derivatization with **16** was then applied to the irradiated **5** in THF (Entry 4 of Table S2). In this case, the solvent was first removed *in vacuo*, the residue was re-dissolved in a freshly prepared solution of **16** in excess (3 mg) in $CDCl_3$ (0.6 mL). This was directly submitted to $^1$H-NMR analysis, the results are shown in Figure S13. A control solution, prepared from pristine **5** (3 mg) and **16** (3 mg) in $CDCl_3$ (0.5 mL), was also submitted to $^1$H-NMR analysis. Observing the comparison of the NMR spectra (Figure S13b) it can be seen that before irradiation the only peaks present are related to **16** in the range δ = 7.30-7.20 ppm and to **5** at δ = 7.70 and 7.42 ppm (g and h in Figure S13b). After irradiation, the peaks of nitrile **7** at δ = 7.63 and 7.49 ppm (g' and h' in Figure S13b) in parallel with the aromatic pattern related to the final adduct **17** at δ = 7.08, 7.02 and 6.90 ppm (o, o', o'', and o''' in Figure S13b) emerge. This is significant proof of the formation of amine **1** in concomitance with the conversion to the nitrile **7**. Furthermore, the integrals of the pattern of the adduct **17** and of the signals of **7** are comparable, indicating an high efficiency of the amine formation.

The detection of the amine through derivatization with **16** was also performed after the irradiation of **14** in DMF. The removal of DMF *in vacuo* requires higher vacuum, thus either the amine **1** or the piperidine would be eventually removed together with the solvent. Therefore the derivatization with **16** had to be carried out directly in DMF. Since the subsequent evaporation of the solvent in a hot $H_2O$ bath would partially trigger the reaction between **16** and the OH groups of **5**, the control compound **14** was chosen for this experiment. To the irradiated solution of **14** in DMF (Entry 7 of Table S2), the carbonate **16** (4 mg) was added. Then, the solvent was removed *in vacuo*, the final residue was re-dissolved in $CDCl_3$ (0.5 mL), and submitted to $^1$H-NMR analysis. Again, a control solution of **14** (3 mg) and **16** (4 mg) was prepared in DMF, the solvent was removed *in vacuo*, the residue re-dissolved in $CDCl_3$, and submitted to $^1$H-NMR analysis. The results are shown in Figure S14. The NMR spectra in Figure S14b present a significant decrease of the peak of **16** in the range of δ = 7.20-7.30 ppm, while peaks deriving from its thermal decomposition during the solvent evaporation emerge (black dots in Figure S14b). Before irradiation, only the starting aromatic peaks of **14** are visible at δ = 7.72 and 7.42 (g and h in Figure S14b). However, after irradiation a partial appearance of the aromatic peaks of the nitrile **9** at δ = 7.66 and 7.50 (g' and h' in Figure S14b) together with the adduct piperidine-**16** at δ = 7.08, 7.03 and 6.90 (o, o', o'', and o''' in Figure S14b) can be observed. Moreover, one can clearly see the comparable integration of emerging peaks of **9** and the pattern of piperidine-**16**, as indication of the high efficiency of the amine formation.

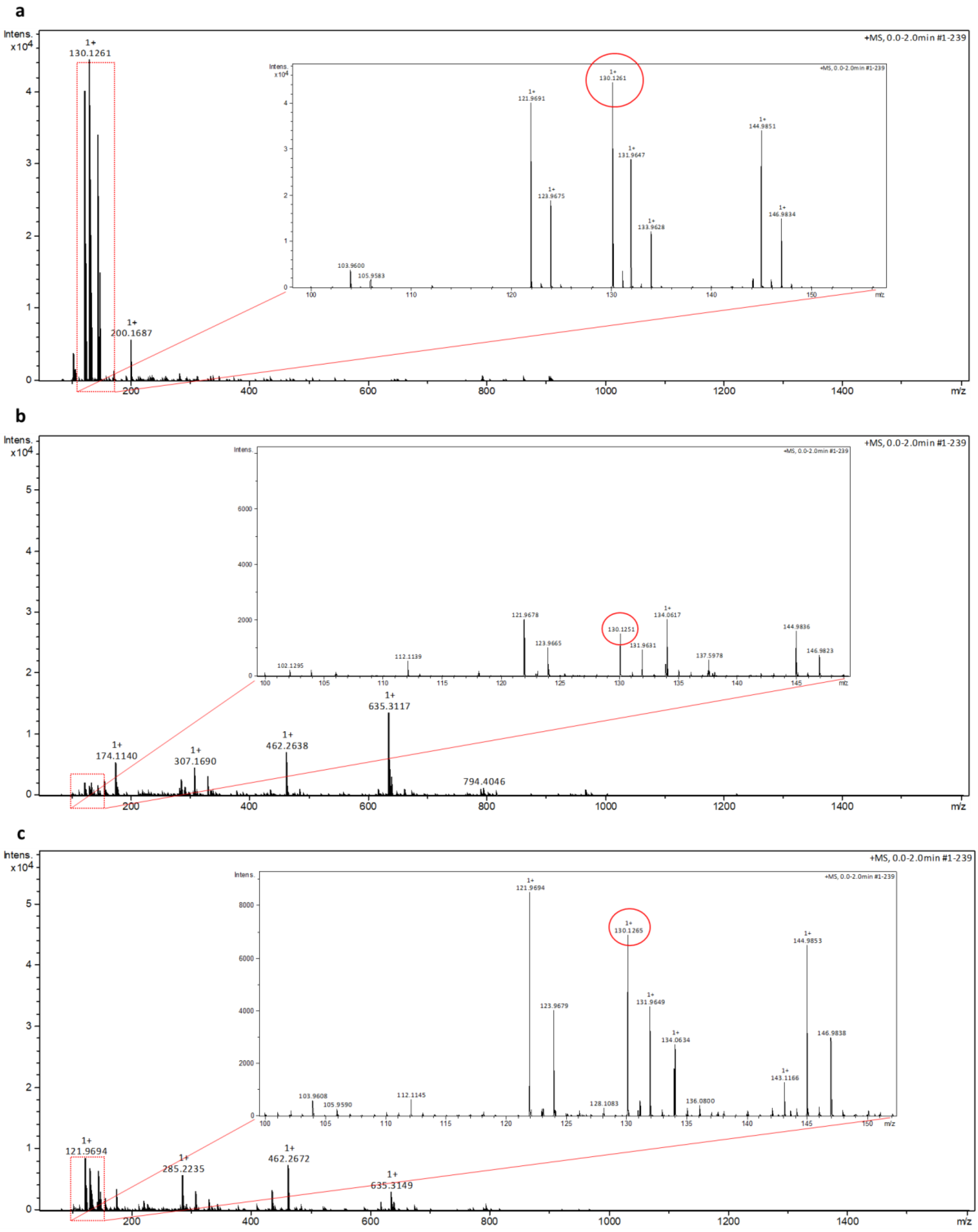


**Figure S11.** ESI$^{+}$ HRMS spectra with zoomed in region between $m/z$ = 100 and $m/z$ = 1600. (a) Spectrum of control **1**. (b) Spectrum of pristine **5**. (c) Spectrum of irradiated **5** (Entry 3 of Table S2).

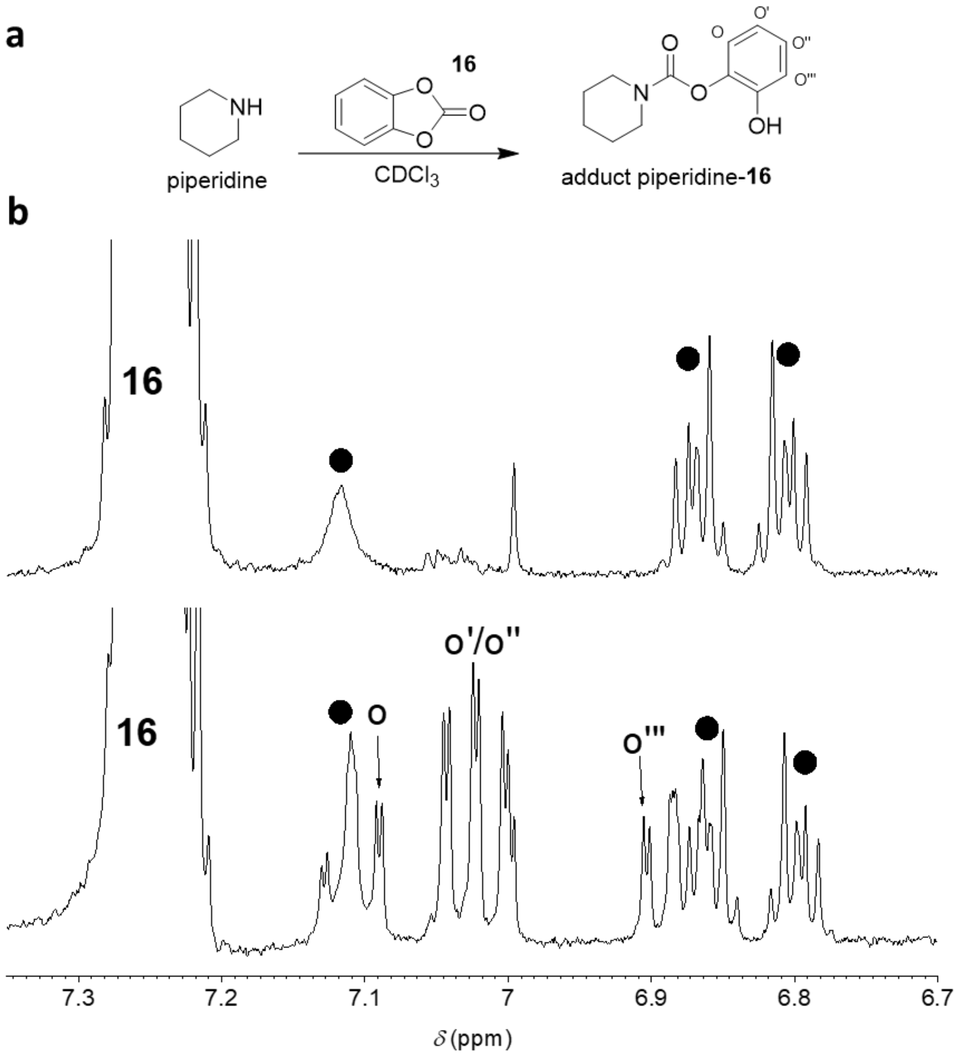


**Figure S12.** (a) Reaction scheme for the derivatization of the piperidine with carbonate **16**. (b) Comparison of $^1$H-NMR spectra in $CDCl_3$ zoomed in between δ = 7.4 and 6.7 ppm. From top to bottom: pristine **16**, final adduct **16** + piperidine.

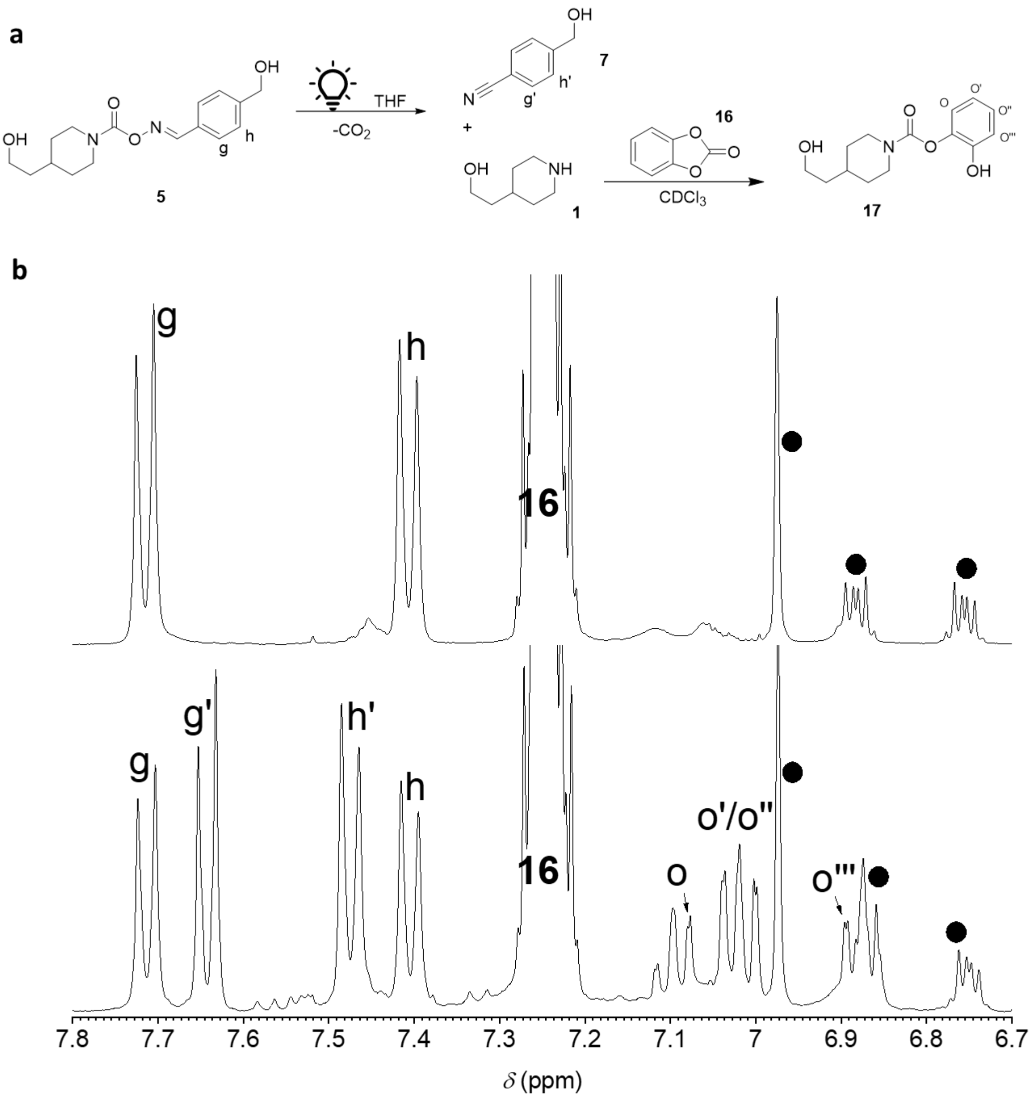


**Figure S13.** (a) Reaction scheme for the irradiation of **5** in THF (Entry 4 of Table S2) with subsequent derivatization of **1** with **16**. (b) Comparison of $^1$H-NMR spectra in $CDCl_3$ zoomed in between δ = 7.8 and 6.7 ppm. From top to bottom: **5** + **16** before irradiation, **5** after irradiation + **16** yielding **17**.

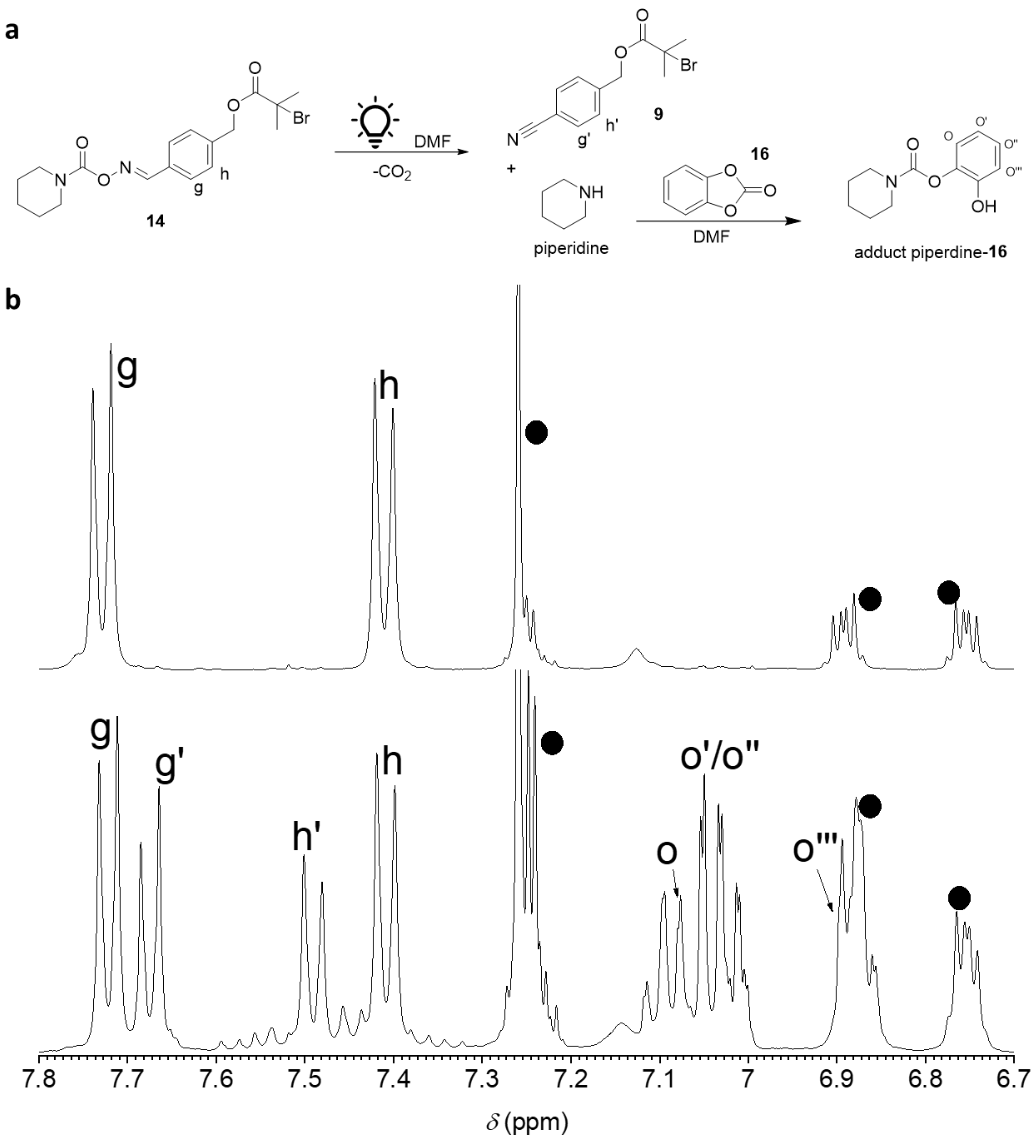


**Figure S14.** (a) Reaction scheme for the irradiation of **14** in DMF (Entry 7 of Table S2) with subsequent derivatization of piperidine with **16**. (b) Comparison of $^1$H-NMR spectra in $CDCl_3$ zoomed in between δ = 7.8 and 6.7 ppm. From top to bottom: **14** + **16** before irradiation, **14** after irradiation + **16**.

## 2.4.5. Linear polymers

Analogously to small molecule **5**, the photochemical activity of **PMA6** and **PHEA6** was verified by UV-vis absorption spectroscopy over the course of irradiation with light in both THF and $H_2O$ (Figure S15 and Figure S16).

After confirming the photochemical activity, the pathway of the iminyl radical intermediate was studied by a detailed $^1$H-NMR analysis of the aromatic outcomes for the irradiated polymers **PMA6** and **PHEA6** (applying 128 scans for a good resolution of the low intensity peaks of the initiator). The experiments were conducted again in the three solvents THF, DMF, and $H_2O$. We focus here on the experiments performed in THF (Entry 8 of Table S2). Therefore, three irradiated batches of 3 mL each were collected together, the solvent was removed *in vacuo*, the residue was dissolved in DMSO-$d_6$, and the sample was submitted to $^1$H-NMR analysis. As shown in Figure S17, the irradiation of **PMA6** in THF also yields **PMA7** as sole product. Exactly the same procedure was repeated for the irradiated **PHEA6** in $H_2O$ (Entry 11 of Table S2) and is discussed in the manuscript. The irradiation of **PMA6** in DMF (Entry 10 of Table S2) was performed with a high intensity light source. After the irradiation, the DMF was removed

*in vacuo*, the residue was dissolved in DMSO-$d_6$, and the sample was submitted to $^1$H-NMR analysis, as discussed in the manuscript.

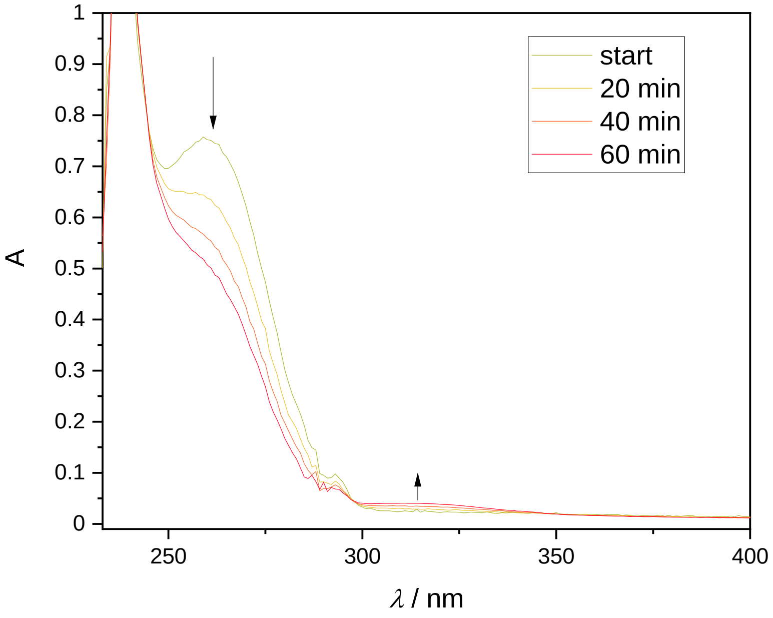


**Figure S15.** UV-vis absorption spectra of **PMA6** in THF (Entry 8 of Table S2) at different irradiation times.

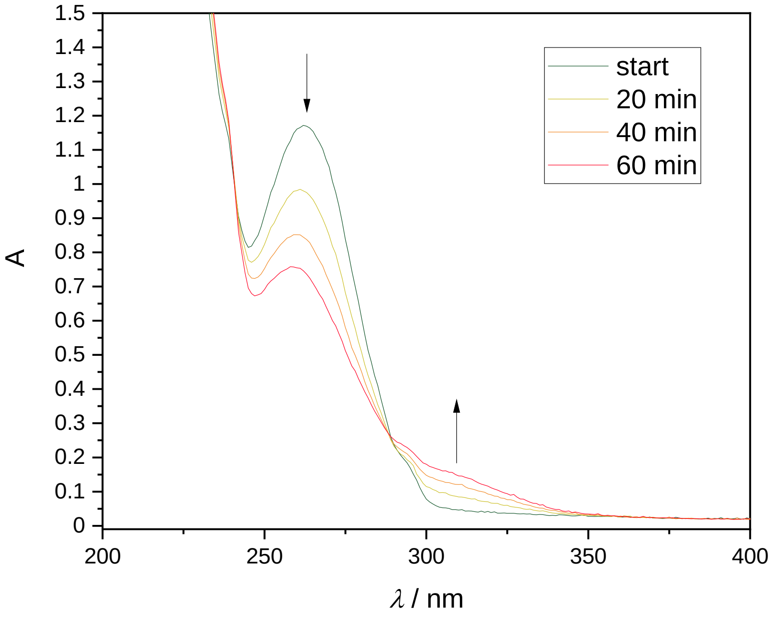


**Figure S16.** UV-vis absorption spectra of **PHEA6** in $H_2O$ (Entry 11 of Table S2) at different irradiation times.

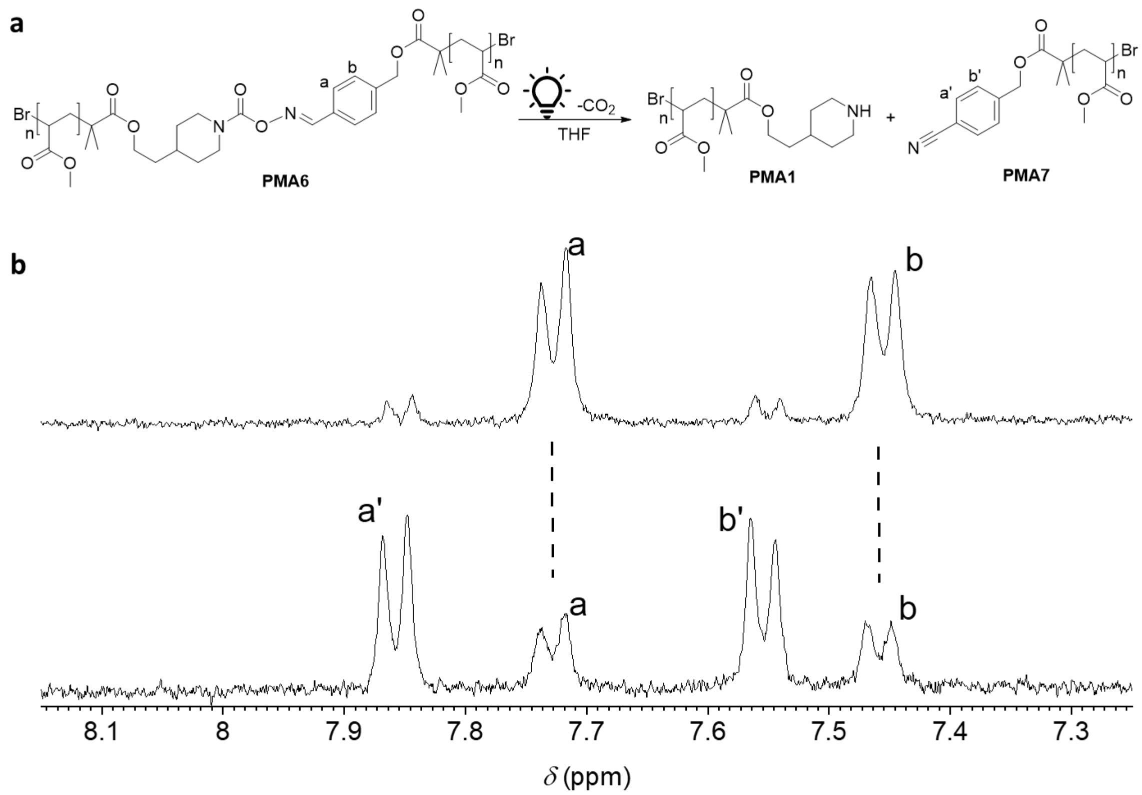


**Figure S17.** (a) Summary of the irradiation outcomes of **PMA6** in THF (Entry 8 of Table S2) giving the nitrile **PMA7** as only product. The amine **PMA1** is derived from the aminyl radical intermediate and it is not subject of this NMR analysis (identified by chemical derivatization, *cf.* Section 2.7). (b) Comparison of $^1$H-NMR spectra in DMSO-$d_6$ zoomed in between δ = 8.15 and 7.25 ppm. From top to bottom: pristine **PMA6**, irradiated **PMA6** in THF (Entry 8 of Table S2).

## 2.5. Ultrasonication experiments

### 2.5.1. General procedure and list of experiments

The investigation of the mechanochemical scission process was conducted by a series of ultrasonication experiments under varying conditions (Table S3). A specific set of sonication parameters and experimental steps was maintained fixed for the entries 1-14: $f$ = of 20 kHz, amplitude of 30%, and pulse sequence 1 s "on" and 1 s "off", while the volume of the sonicated solution was always 10 mL. For entry 15, which represents an important control experiment, the conditions of sonication were kept similar to the ones used for the sonication of polymer **PMA6** in question, subsequently used for the catalysis trials (see experiments in Table S6, in section 2.8.1). These conditions were the following: pulse sequence was kept at 1 s "on" and 1 s "off", amplitude was increased to 60%, and volume was increased to 30 mL.

**Table S3.** List of the sonication experiments, with related parameters, conditions, and analytic techniques performed.

| entry | compound no. | solvent | sonication time / h | $\rho$ / mg·mL$^{-1}$ | analyses and Figures |
|---|---|---|---|---|---|
| 1 | **PMA6$_{107}$** | THF | 1 | 1.0 | GPC (Figure 1a) |
| 2 | **PMA6$_{145}$** | DMF | 5 | 5.0 | NMR (Figure 1c, Figure S30, Figure S22), GPC (Figure S23) |
| 3 | **PMA6$_{80}$** | THF | 2 | 2.5 | NMR (Figure S19) |
| 4 | **PMA6$_{145}$** | THF | 3 | 2.5 | NMR (Figure S19) |
| 5 | **PMA6$_{145}$** | THF | 10 | 2.5 | NMR (Figure S19 and Figure S24), GPC (Figure S25) |
| 6 | **PMA6$_{80}$** | THF/$H_2O$ 8:2 (v/v) | 2 | 2.5 | NMR (Figure S20) |
| 7 | **PMA6$_{80}$** | THF/$H_2O$ 7:3 (v/v) | 2 | 2.5 | NMR (Figure S20 and Figure S26), GPC before (Figure S27) and after labelling with RhBITC (Figure 2b) |
| 8 | **PMA6$_{90}$** | THF/$H_2O$ 7:3 (v/v) | 3 | 2.5 | NMR (Figure S21) |
| 9 | **PMA6$_{90}$** | DMF | 3 | 2.5 | NMR (Figure S21) |
| 10 | **PHEA6$_{44}$** | $H_2O$ | 1 | 2.5 | NMR (Figure 1d, Figure S31 and Figure S28), GPC (Figure S29), UV-vis (Figure S18) |
| 11 | **PMA14** | THF | 2 | 5.0 | NMR (Figure S32) |
| 12 | **PHEA14** | $H_2O$ | 2 | 2.5 | NMR (Figure S33), UV-vis (Figure S34) |
| 13 | **PMA$_{94}$** | THF | 1 | 1.0 | GPC (Figure 1b) |
| 14 | **PMA$_{139}$** | THF | 2 | 5.0 | NMR (Figure S35) |
| 15 | **5** | THF | 3 | 0.033 | NMR (Figure S32) |

All samples in Table S3 that were sonicated in THF, DMF, or THF/$H_2O$ mixtures were filtered through a PTFE membrane filter after sonication to remove metallic residues from the sonicator probe. Afterwards, a small volume (to reach 5 mg of polymer) was collected for GPC analysis. Therefore, the solvent was removed *in vacuo*, the final residue was re-dissolved in THF (for GPC, 1 mL), and submitted to the analysis. The rest of the sample, containing most of the polymer, was dialyzed against MeCN. From the final solution, the solvent was removed *in vacuo* and the residue was re-dissolved in DMSO-$d_6$ for $^1$H-NMR analysis with 128 scans for the visualization of the initiator in the aromatic region.

For the samples sonicated in pure $H_2O$ (Entry 10 and 12 of Table S3), the final solution after sonication was filtered through a PA membrane filter. A small volume (to reach 5 mg) was collected for GPC analysis, the solvent was removed by freeze-drying, the final residue was re-dissolved in DMF (for GPC, 1 mL), and submitted to the analysis.

The rest of the sample, containing most of the polymer, was dialyzed against $H_2O$. From the final solution, the solvent was removed by freeze-drying and the final residue was re-dissolved in DMSO-$d_6$ for $^1$H-NMR analysis, again applying 128 scans. For the UV-vis absorption analysis, a second batch of the samples was sonicated. Again, the solution was then filtered and dialyzed as described above. Afterwards, the final solution was adjusted to the initial volume (10 mL) to maintain the initial concentration. Then volume fraction of this solution was used for the optical analysis in comparison with the pristine, non-sonicated polymer.

### 2.5.2. Mechanochemical activity

$^1$H-NMR and GPC samples were prepared according to the general procedure. The evidence of the mechanochemical activity of the mechanophores **PMA6** and **PHEA6** was shown by NMR and GPC, discussed in the main manuscript. Concerning the sonication of **PHEA6** in $H_2O$ (Entry 10 of Table S3), the additional UV-vis absorption spectra are depicted in Figure S18. In this case, GPC was not the ideal method because any reference polymer sonicated in $H_2O$ would show a lot of unselective cleavage due to the high tensile force applied to the chains. Therefore, it would be difficult to extract information concerning the possible cleavage of the mechanophore. Hence, UV-vis absorption spectra were recorded before and after sonication, from which a decrease of the peak at 262 nm can be discerned. In contrast, the sonication of the chain-terminal control **PHEA14** does not give any significant change in the UV-vis absorption spectrum (Figure S34).

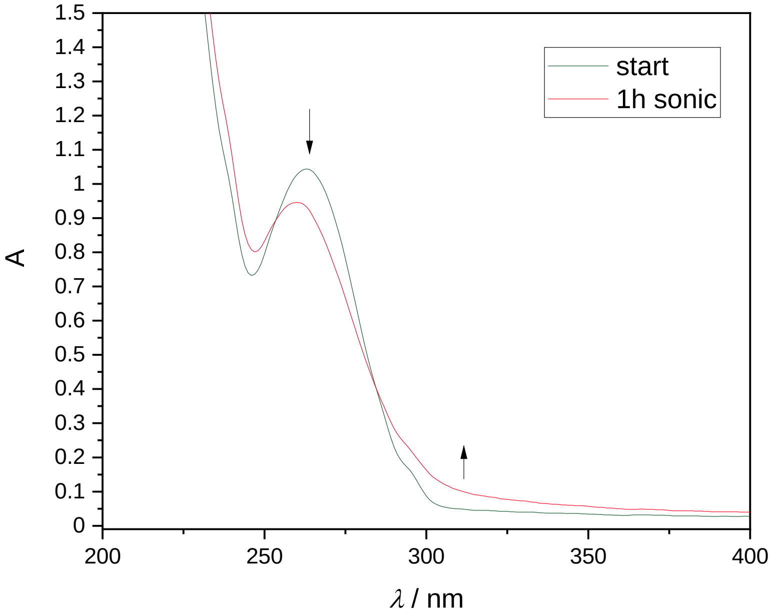


**Figure S18.** UV-vis absorption spectra of **PHEA6$_{44}$** (2.5 mg·mL$^{-1}$) before and after sonication (Entry 10 of Table S3).

### 2.5.3. Influence of ultrasonication parameters

The mechanochemical scission kinetics of the carbamoylaldoxime mechanophore were investigated by sonicating **PMA6** in different conditions. $^1$H-NMR samples were prepared according to the general procedures (*cf.* Section 2.5.1). Comparison of the spectra for different entries are reported. Figure S19 (comparison between Entry 3, 4, and 5 of Table S3) concerns the sonication in THF for different durations and with different $M_n$ of the polymer chain. Both Figure S20 (comparison between Entry 6 and Entry 7 of Table S3) and Figure S21 concern the sonication with different solvents THF/$H_2O$ at different ratios and DMF.

Even though the sonication performed in THF gives lower kinetics than DMF, the nitrile **PMA7** is the aromatic product in both solvents. The mechanochemistry of **PMA6** was additionally studied in the mixture THF/ $H_2O$; the

addition of $H_2O$ was reflected by additional peaks emerging at δ = 7.93 and 7.59 ppm (a'' and b'' in Figure S21b), which are assigned to aldehyde **PMA8** (see Figure S21a).

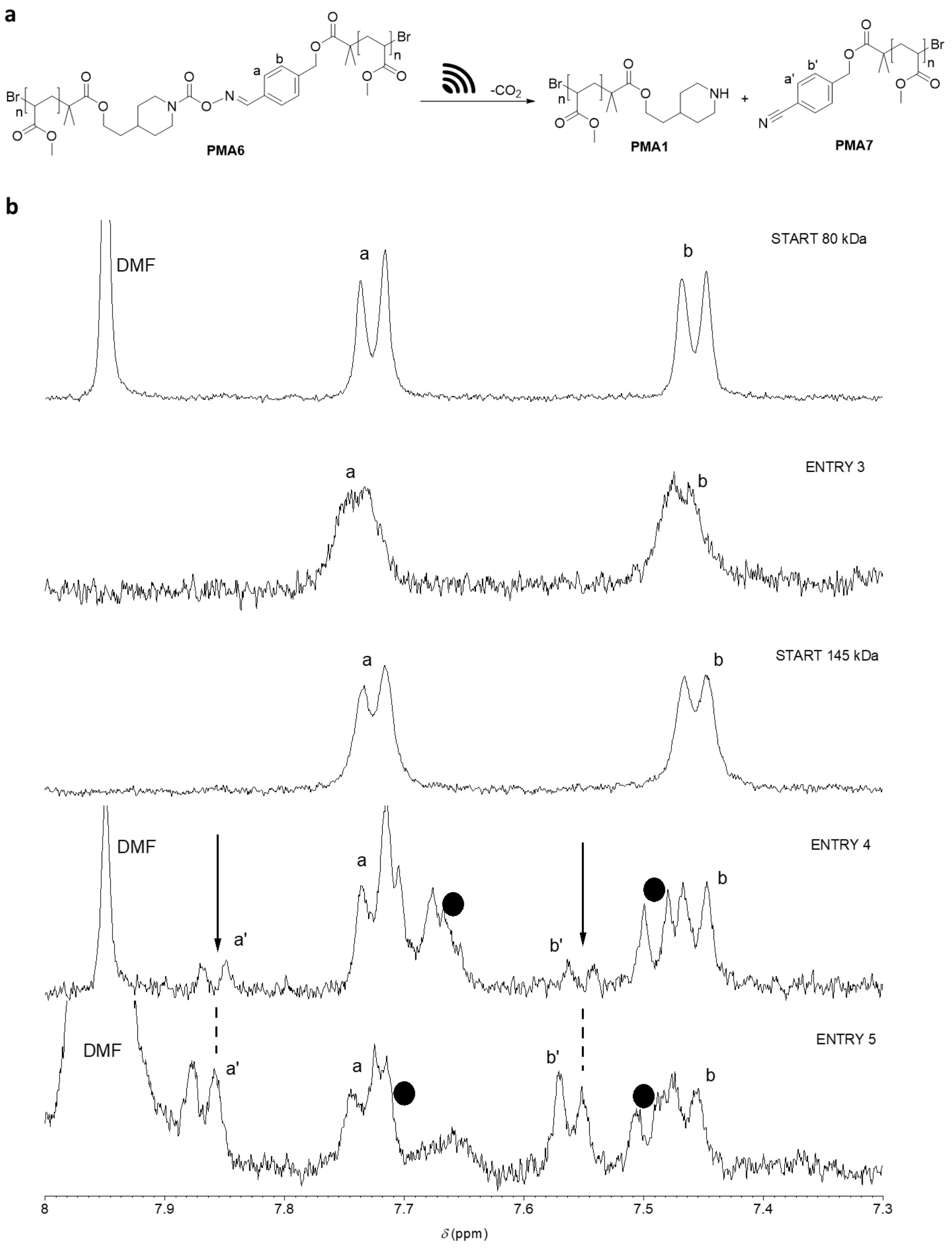


**Figure S19.** (a) Reaction scheme of the sonication of **PMA6** in THF. **PMA7** is the only aromatic outcome. **PMA1** derived from the aminyl radical pathway is not subject of this NMR study (identified by chemical derivatization, *cf.* Section 2.7). (b) Comparison of $^1$H-NMR spectra in DMSO-$d_6$ zoomed in between 8.0 and 7.3 ppm of different sonicated **PMA6** samples. Black dots are undesired aromatic peaks visible also from sonicating the control polymer **PMA$_{139}$** (Entry 14 of Table S3, and Figure S35). From top to bottom: pristine **PMA6$_{80}$**, **PMA6$_{80}$** sonicated in THF for 2 h (Entry 3 of Table S3),

pristine **PMA6₁₄₅**, **PMA6₁₄₅** sonicated in THF for 3 h (Entry 4 of Table S3), **PMA6₁₄₅** sonicated in THF for 10 h (Entry 5 of Table S3).

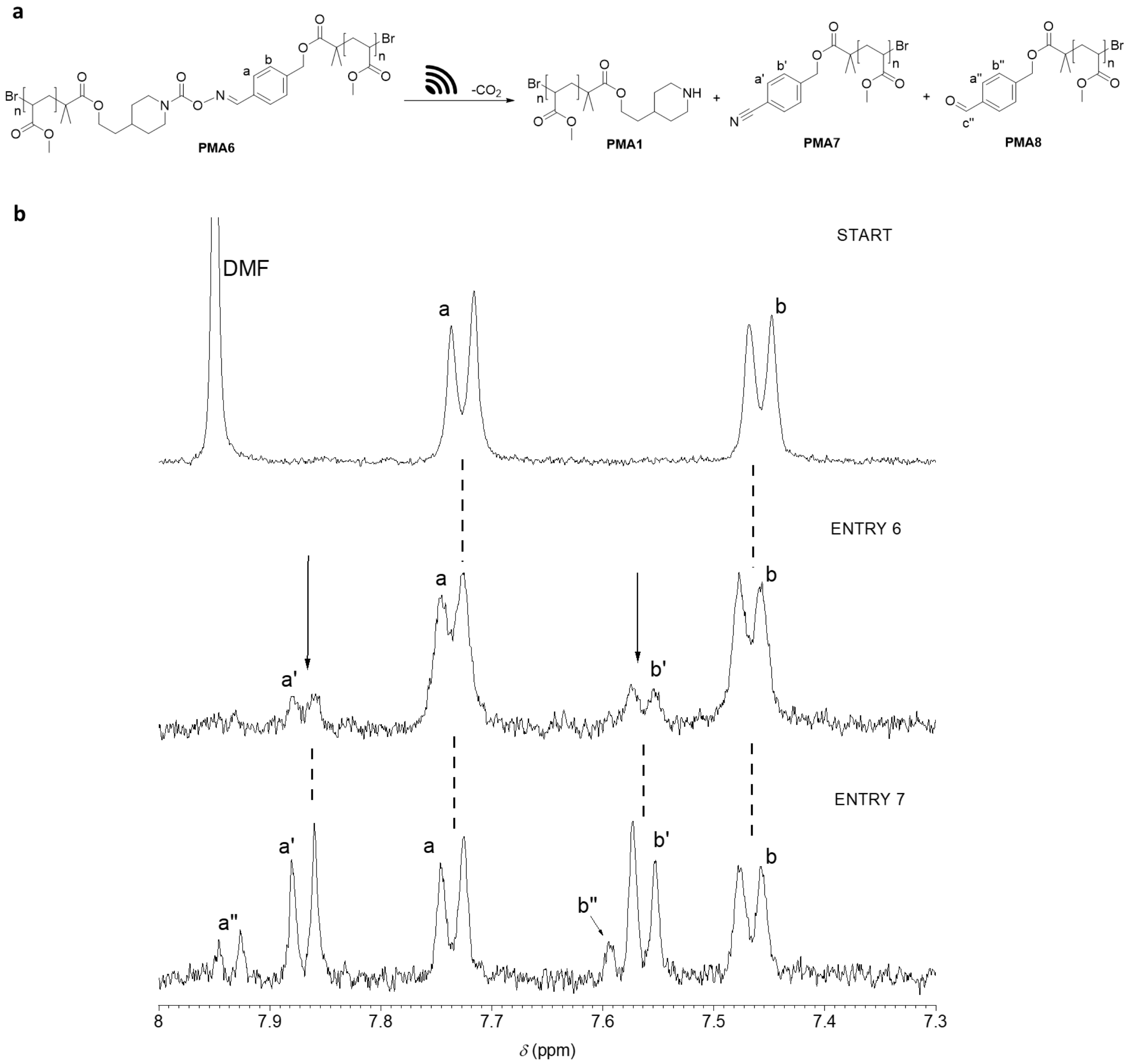


**Figure S20.** (a) Reaction scheme of the sonication of **PMA6** in THF/$H_2O$. **PMA7** and **PMA8** are the aromatic outcomes. **PMA1** derived from the aminyl radical pathway is not subject of this NMR study (identified by chemical derivatization, *cf.* Section 2.7). (b) Comparison of $^1$H-NMR spectra in DMSO-$d_6$ zoomed in between 8.0 and 7.3 ppm of different sonicated **PMA6** samples. From top to bottom: pristine **PMA6$_{80}$**, **PMA6$_{80}$** sonicated in THF/$H_2O$ 8:2 (v/v) for 2 h (Entry 6 of Table S3), **PMA6$_{80}$** sonicated in THF/$H_2O$ 7:3 (v/v) for 2 h (Entry 7 of Table S3).

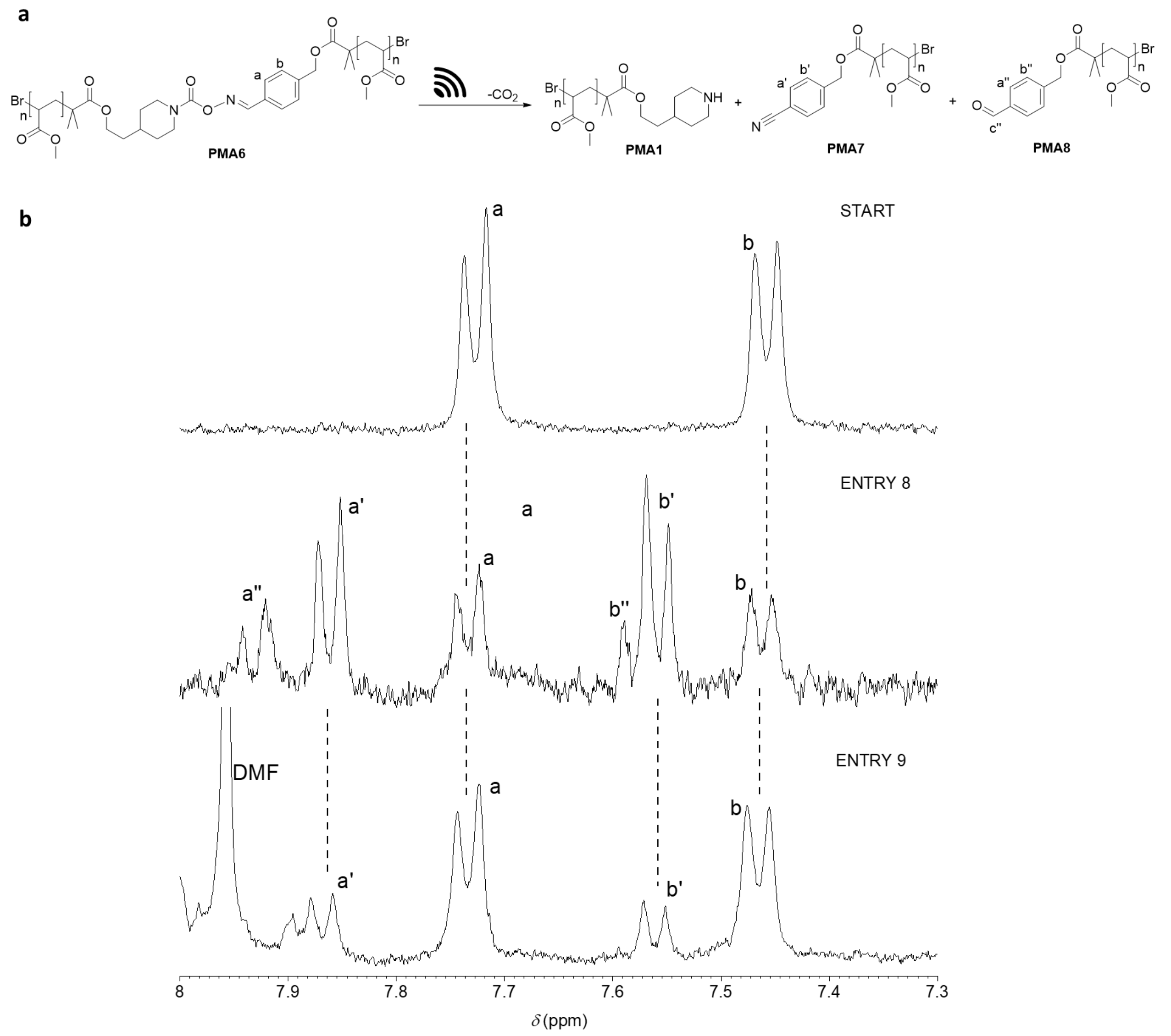


**Figure S21.** (a) Reaction scheme of the sonication of **PMA6** in THF/$H_2O$ and DMF. **PMA7** and **PMA8** are the aromatic outcomes. **PMA1** comes from the aminyl radical pathway is not subject of this NMR study (identified by chemical derivatization, *cf.* Section 2.7). (b) Comparison of $^1$H-NMR spectra in DMSO-$d_6$ zoomed in between 8.0 and 7.3 ppm of different sonicated **PMA6** samples. From top to bottom: pristine **PMA6$_{90}$**, **PMA6$_{90}$** sonicated in THF/$H_2O$ 7:3 (v/v) for 3 h (Entry 8 of Table S3), **PMA6$_{90}$** sonicated in DMF for 3 h (Entry 9 of Table S3).

### 2.5.4. Mechanochemical selectivity

The summary of the calculated data concerning the selectivity study is reported in Table S4. $^1$H-NMR and GPC samples were prepared and analyzed according to the general procedure (*cf.* Section 2.5.1). The peak integrals of the aromatic products in the $^1$H-NMR spectra were used to determine the fraction of cleaved mechanophore (**PMA6** or **PHEA6**), further divided in the fraction of the nitrile (**PMA7** and **PHEA7**) and fraction of the aldehyde (**PMA8** and **PHEA8**). The fraction of total scission was calculated from GPC by the starting $M_n$ ($M_{n,\text{start}}$) and the $M_n$ after sonication ($M_{n,\text{end}}$) of the polymer according to the following equation:[9]

$$\text{fraction of total scission} = \frac{M_{n,\text{end}}^{-1} - M_{n,\text{start}}^{-1}}{M_{n,\text{start}}^{-1}}$$

$^1$H-NMR integrals and GPC elugrams of Entry 2, 5, 7, and 10 of Table S3 and Table S4 are depicted from Figure S22 to Figure S29. From these data, the final selective scission represents the percentage of the selectively cleaved mechanophore in a sample of *n* polymer chains at the desired bond (oxime bond) out of the total number of scission events along the polymer chain in the same sample of *n* polymers. This scission selectivity (in %) was calculated as follows:

$$\text{selective scission} = \frac{\text{fraction of cleaved mechanophore}}{\text{fraction of total scission}} \cdot 100$$

**Table S4.** Summary of the calculated data for the evaluation of the scission selectivity.

| entry of Table S3 | fraction of total scission (GPC) | fraction of nitrile after scission | fraction of aldehyde after scission | fraction of cleaved mechanophore | scission selectivity / % |
|---|---|---|---|---|---|
| 2 | 1.23 | 0.66 | - | 0.66 | 54 |
| 5 | 0.86 | 0.52 | - | 0.52 | 60 |
| 7 | 0.83 | 0.45 | 0.10 | 0.55 | 66 |
| 10 | 0.95 | 0.18 | 0.22 | 0.40 | 42 |

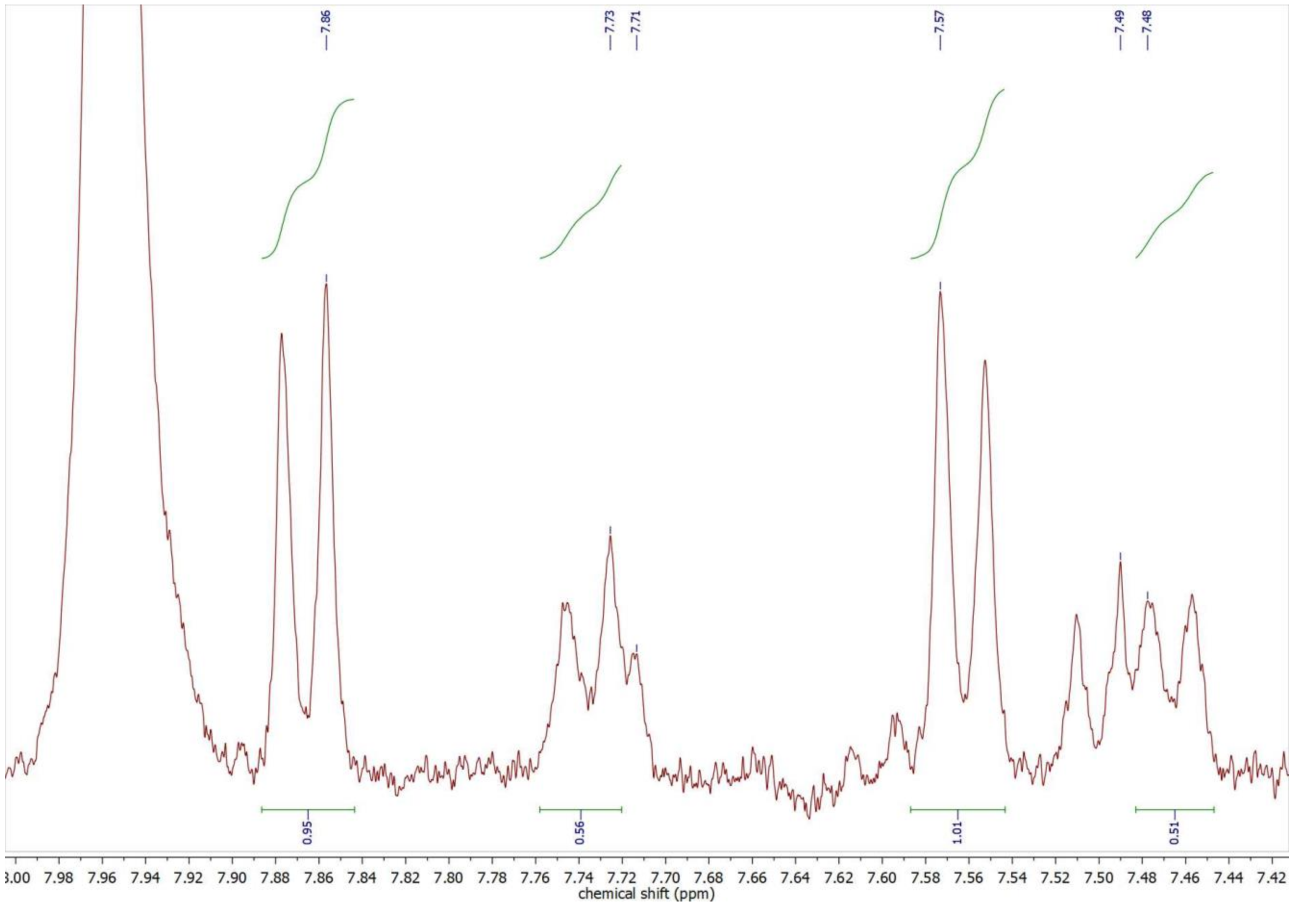


**Figure S22.** $^{1}$H-NMR spectrum in DMSO-$d_6$ with integral analysis of Entry 2 in **Table S3** and **Table S4**. Starting **PMA6$_{145}$** at δ = 7.71 and 7.48 ppm; nitrile **PMA7** at δ = 7.86 and 7.57 ppm. The integrals of the peaks at δ = 7.57 and 7.48 ppm were used for the calculations.

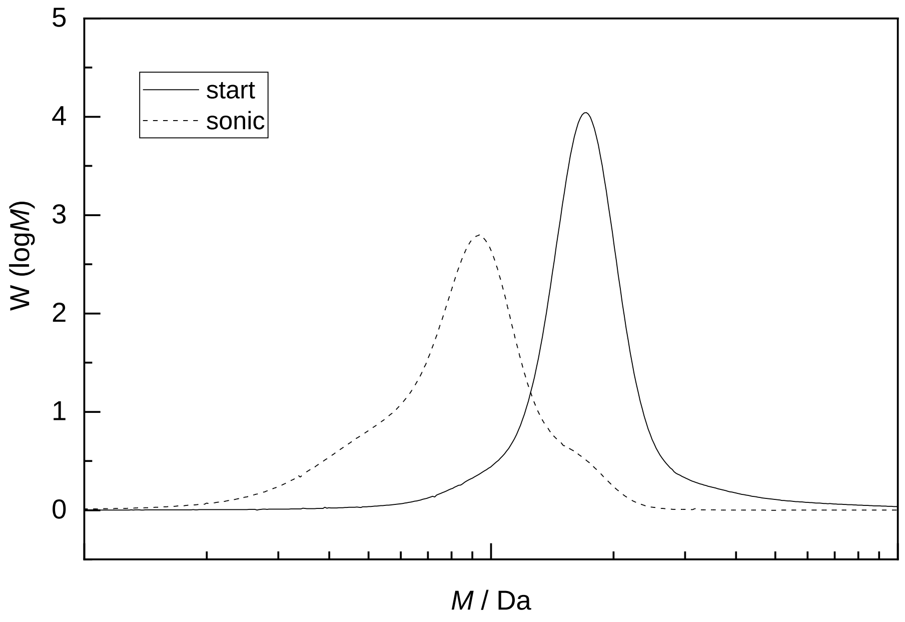


**Figure S23.** GPC elugrams in THF of pristine **PMA6$_{145}$** (solid line) versus the sonicated sample (Entry 2 of **Table S3** and **Table S4**, dashed line).

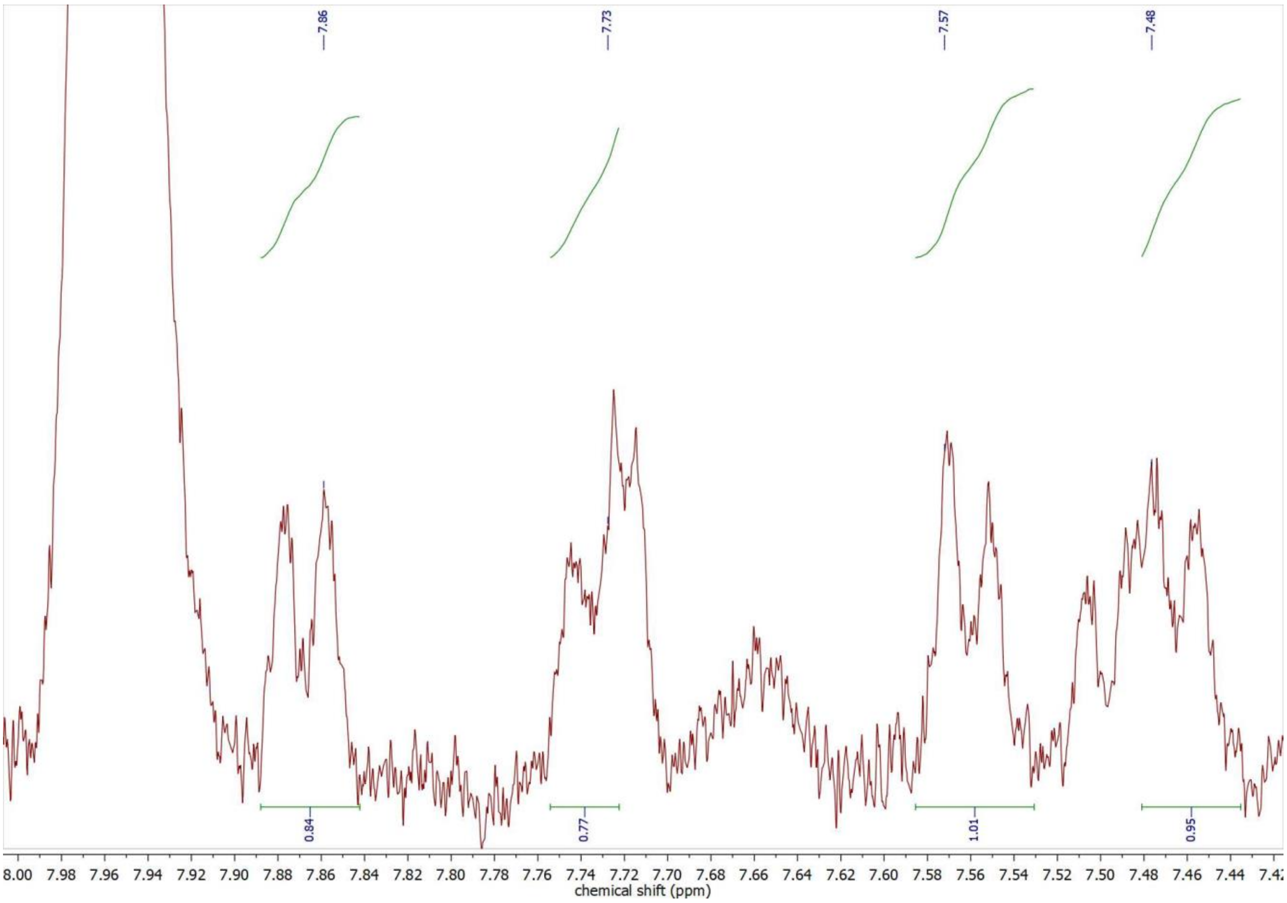


**Figure S24.** $^1$H-NMR spectrum in DMSO-$d_6$ with integral analysis of Entry 5 in **Table S3** and **Table S4**. Starting **PMA6$_{145}$** at δ = 7.73 and 7.48 ppm; nitrile **PMA7** at δ = 7.86 and 7.57 ppm. The integrals of the peaks at δ = 7.57 and 7.48 ppm were used for the calculations.

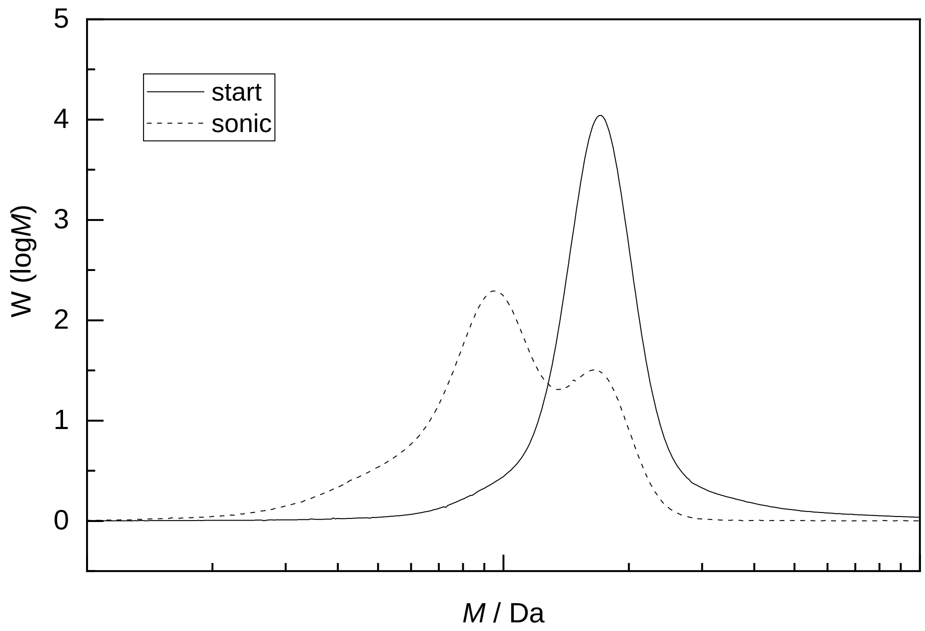


**Figure S25.** GPC elugrams in THF of pristine **PMA6$_{145}$** kDa (solid line) versus the sonicated sample (Entry 5 of **Table S3** and **Table S4**, dashed line).

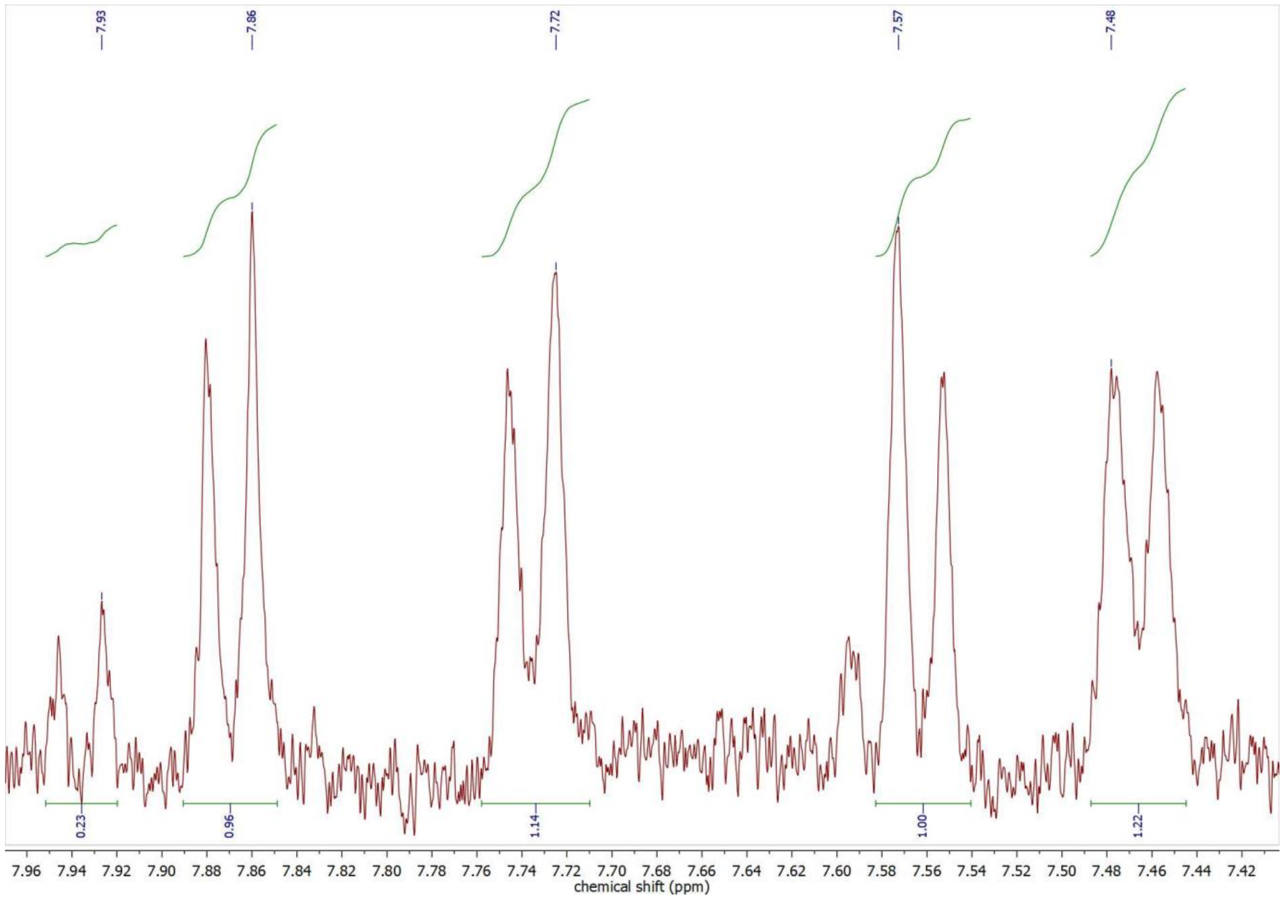


**Figure S26.** $^{1}$H-NMR spectrum in DMSO-$d_6$ with integral analysis of Entry 7 in **Table S3** and **Table S4**. Starting **PMA6**$_{80}$ at δ = 7.72 and 7.48 ppm; nitrile **PMA7** at δ = 7.86 and 7.57 ppm; aldehyde **PMA8** at δ = 7.93 and 7.59 ppm. The integrals of the peaks at δ = 7.93, 7.57, and 7.48 ppm were used for the calculations.

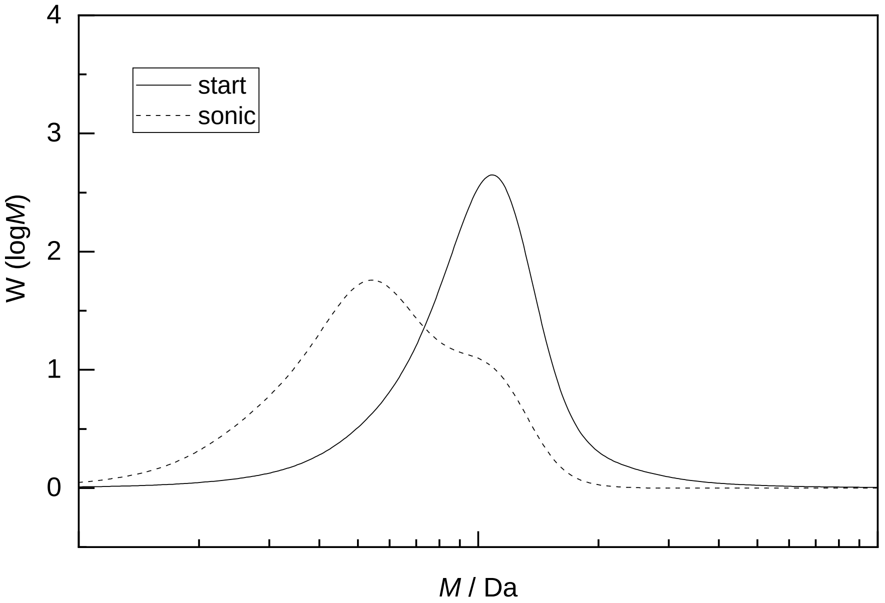


**Figure S27.** GPC elugrams in THF of pristine **PMA**$_{80}$ (solid line) versus the sonicated sample (Entry 7 of **Table S3** and **Table S4**, dashed line).

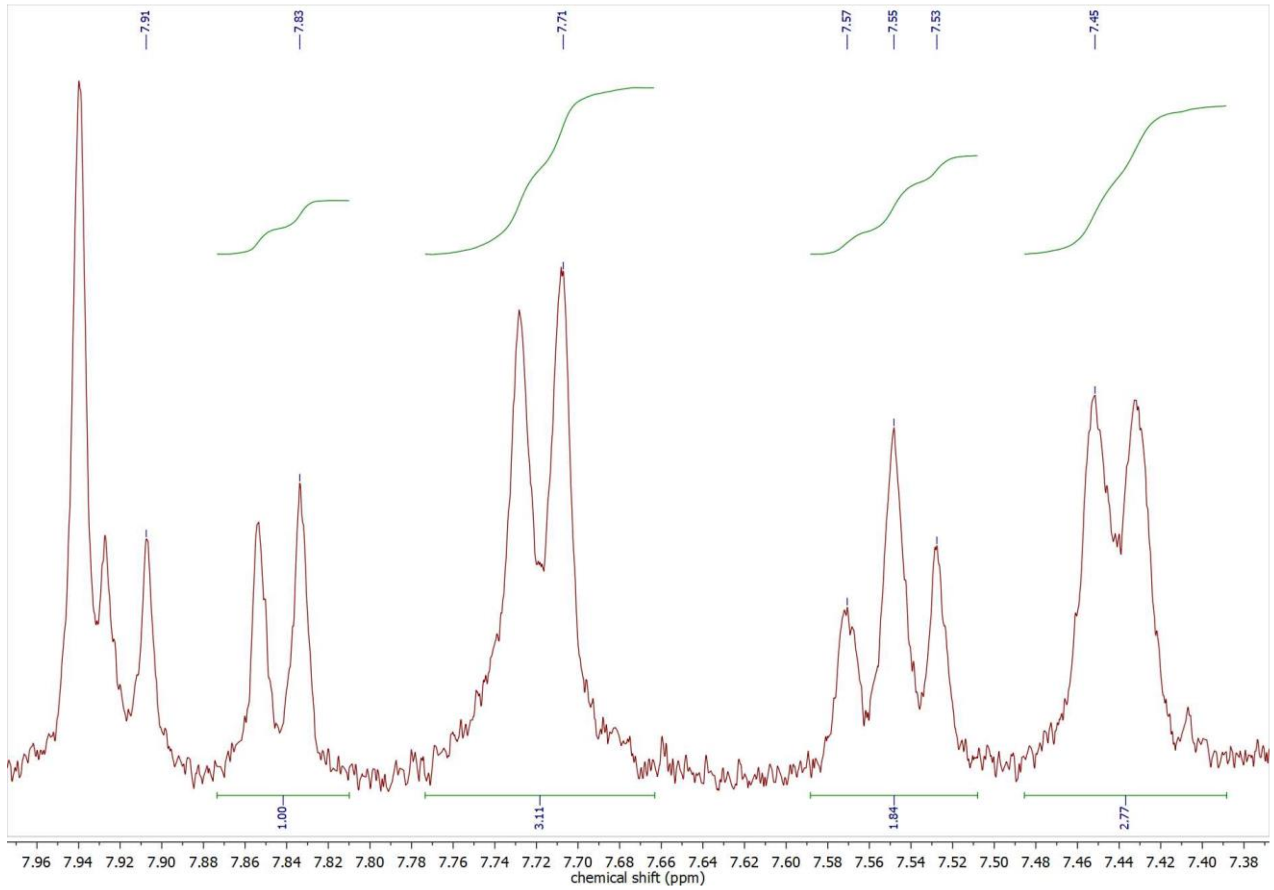


**Figure S28.** $^{1}$H-NMR spectrum in DMSO-$d_6$ with integral analysis of Entry 10 in **Table S3** and **Table S4**). Starting **PHEA6$_{44}$** at δ = 7.71 and 7.45 ppm; nitrile **PHEA7** at δ = 7.83 and 7.55 ppm; aldehyde **PHEA8** at δ = 7.91 and 7.57 ppm. The integrals of the peaks at δ = 7.91, 7.57+7.55 (partially overlapped), and 7.48 ppm were used for the calculations.

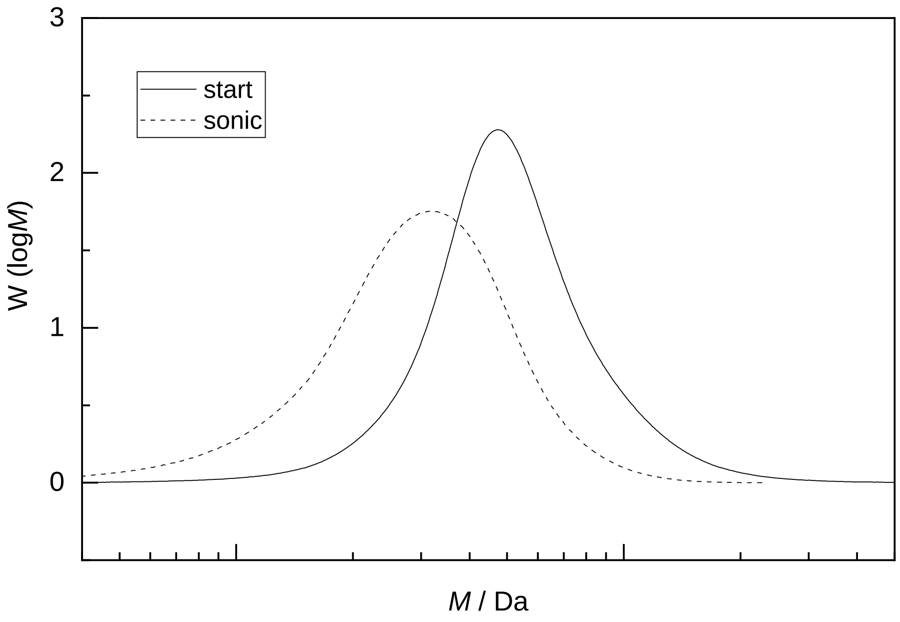


**Figure S29**. GPC elugrams in DMF of pristine **PHEA6$_{44}$** (solid line) versus the sonicated sample (Entry 10 of **Table S3** and **Table S4**, dashed line).

## 2.5.5. Complete NMR analysis after irradiation and sonication (Figure 1c,d)

Here (Figure S30 and Figure S31) is reported a more complete NMR analysis of the Figure 1c and 1d (main manuscript), by stacking and comparing the synthetized reference compound **PMA7, PMA8, PHEA7, PHEA8.**

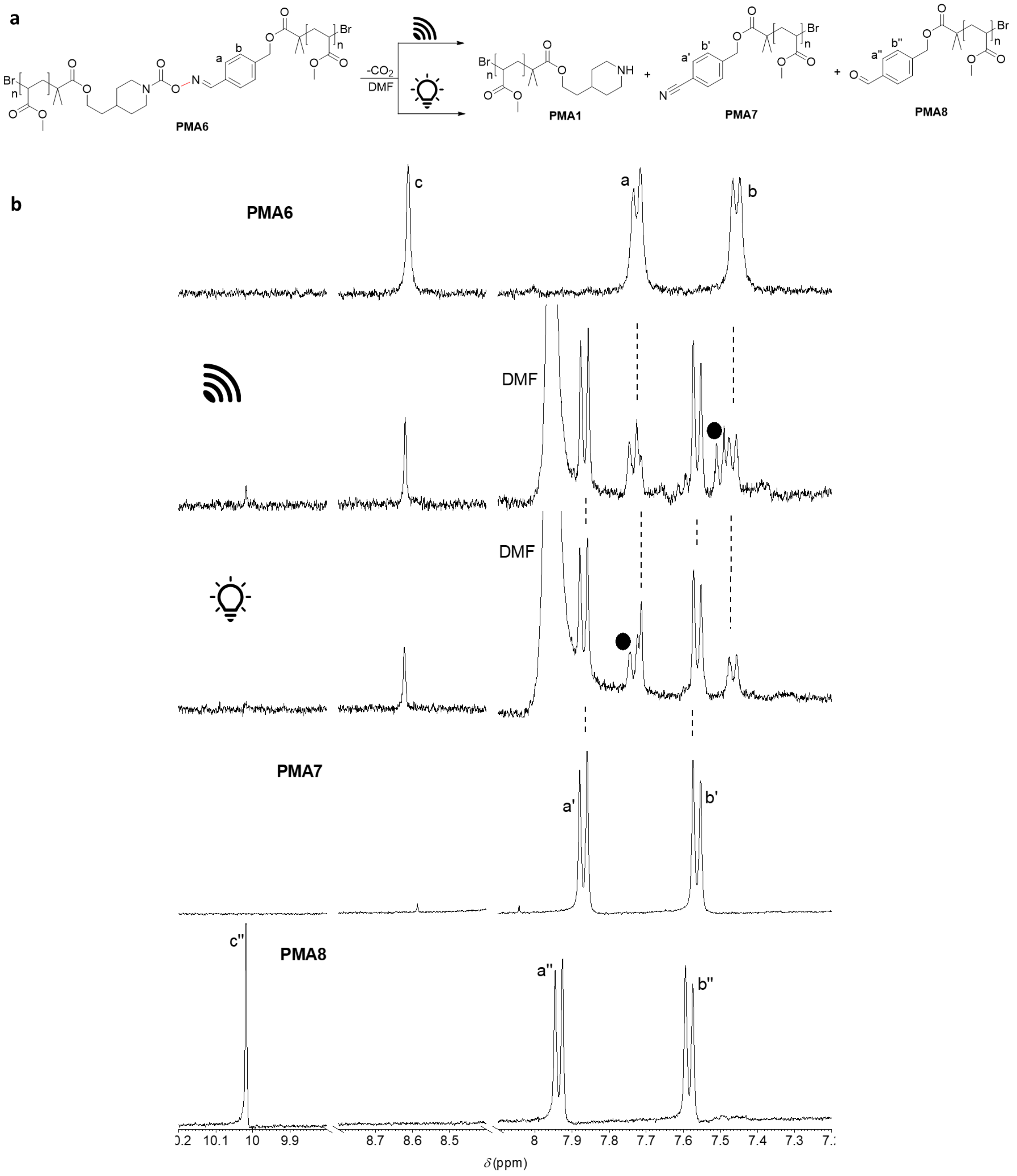


**Figure S30.** (a) Reaction scheme of the sonication and irradiation of **PMA6** in DMF. (b) Comparison of $^{1}$H-NMR spectra in DMSO-$d_6$ zoomed in between 10.2 and 7.2 ppm. Black dots are undesired aromatic peaks, which are visible also from sonicating the control polymer **PMA$_{139}$** (*cf.* Entry 14 of Table S3 and Figure S35). From top to bottom: pristine **PMA6$_{145}$**, **PMA6$_{145}$** sonicated in DMF for 5 h (Entry 2 of Table S3), PMA6$_{145}$ irradiated in DMF for 1 h (Entry 10 of Table S2), pristine **PMA7**, pristine **PMA8**.

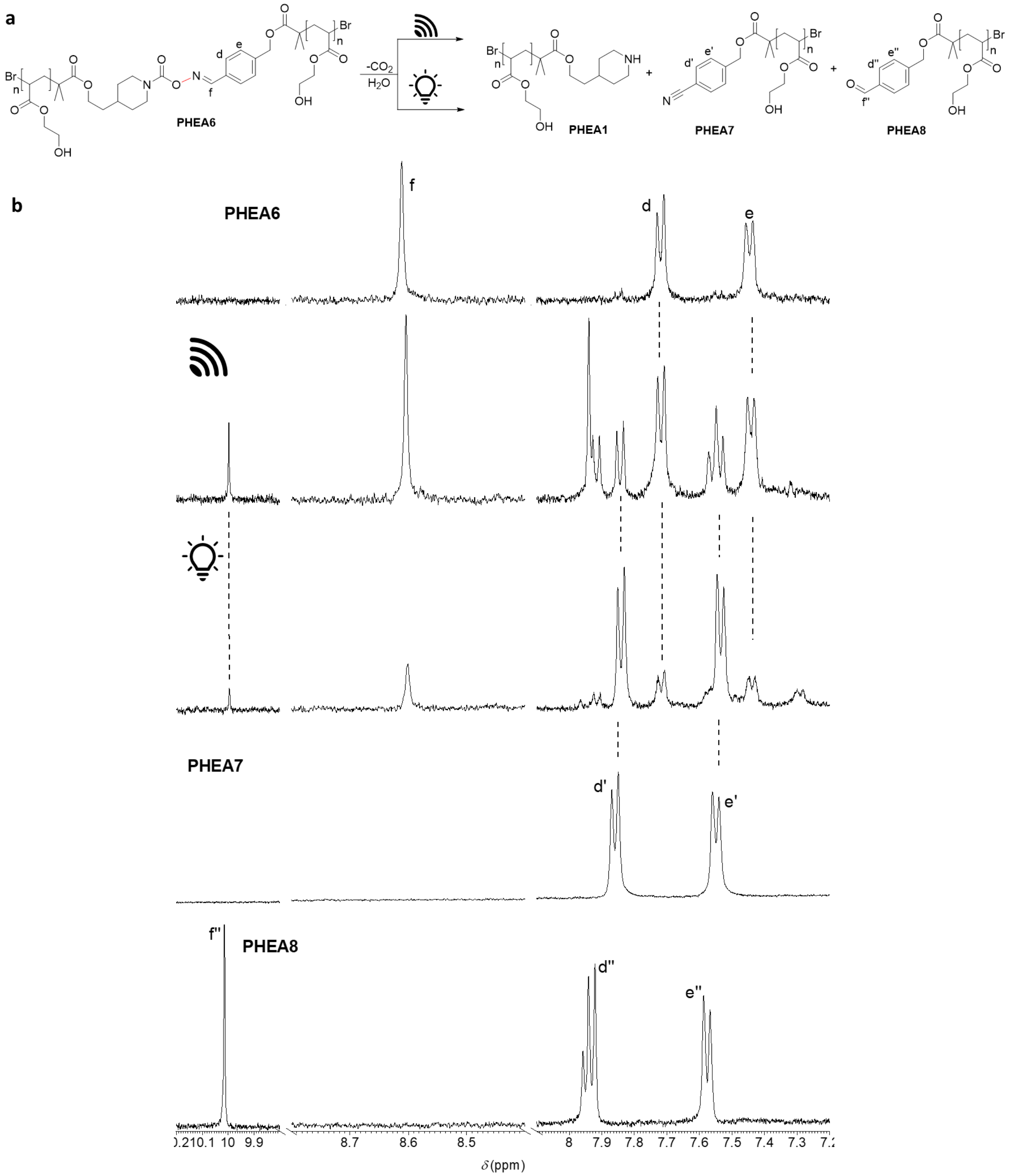


**Figure S31.** (a) Reaction scheme of the sonication and irradiation of **PHEA6** in DMF. (b) Comparison of $^1$H-NMR spectra in DMSO-$d_6$ zoomed in between 10.2 and 7.2 ppm. From top to bottom: pristine **PHEA6$_{44}$**, **PHEA$_{44}$** sonicated in $H_2O$ for 1 h (Entry 10 of Table S3), **PHEA$_{44}$** irradiated in $H_2O$ for 1 h (Entry 11 of Table S2), pristine **PHEA7**, pristine **PHEA8**.

### 2.5.6. Control experiments

$^1$H-NMR and UV-vis samples were prepared and analyzed according to the general procedure (*cf.* Section 2.5.1). Control experiments are required to prove that the scission of the mechanophore by sonication is a mechanochemical process caused by the tensile force applied to the polymer chains. Therefore, polymers bearing the mechanophore as end group in a chain-terminal position were synthesized (**PMA14** and **PHEA14**, *cf.* Section 2.2). $^1$H-NMR measurements over the course of the sonication of these control polymers (Entry 11 and 12 of Table S3) are reported in Figure S32 and Figure S33. The final spectra after sonication resemble those before sonication strongly suggesting that the mechanophore was not activated. Furthermore, for the sonicated **PHEA14** the UV-vis absorption analysis was also performed (Figure S34), from which we can deduce that there is no significant variation. Additionally, as further control experiment, compound **5** was sonicated under the same conditions used in Table S6, where the polymers were subsequently used for the organocatalysis experiments. These conditions are the strongest used in this work, mostly due to the high amplitude (60%), hence, an inactivity of **5** under these conditions would unequivocally reflect the mechanochemical nature of the process related to the oxime bond cleavage. As result (Figure S33d), the $^1$H-NMR spectra clearly show no significant cleavage of the oxime bond, but only a neglectable presence at very low intensity of probable peaks associated with the nitrile **7**, that is formed probably in very low amount during some residual thermal processes in the proximity of the cavitation bubbles, given the higher diffusion ability of the small molecule **5** compared to the mechanophore incorporated in the middle of the polymer chain.

Further proof that the cleavage of the mechanophore by sonication is a mechanochemical process is provided by the result of sonicated **PMA6$_{80}$** versus **PMA6$_{145}$** in THF (Entry 3 and Entry 4 of Table S3). The $^1$H-NMR analysis of these two latter experiments were reported in Figure S20, where it is visible that a small amount of nitrile **PMA7** has only been produced by sonicating **PMA6$_{145}$** while no new aromatic peaks appeared for the sonicated **PMA6$_{80}$**. The fact that the partial cleavage of the mechanophore is only observed for the polymer with higher starting $M_n$ represents a clear proof that the cleavage is caused by the tensile forces applied to the polymer.

An additional control experiment was performed by sonicating the polymer **PMA$_{139}$** (Entry 14 of Table S3) for which a detailed $^1$H-NMR analysis of the aromatic region was carried out with the purpose of elucidating eventual presence of side peaks that are unrelated to the mechanophore but are present in the NMR spectra of other sonicated samples of **PMA6**. It was clarified that residual peaks observed in the $^1$H-NMR of Entry 2, 4, and 5 of Table S3 at $\delta$ = 7.71 and 7.50 ppm (black dots in Figure 2b and Figure S19) are also present in the $^1$H-NMR spectrum of sonicated **PMA$_{139}$** (black dots in Figure S35). This suggests that these peaks stem from impurities from the immersion probe, but are not related to the mechanophore structure and mechanochemical products.

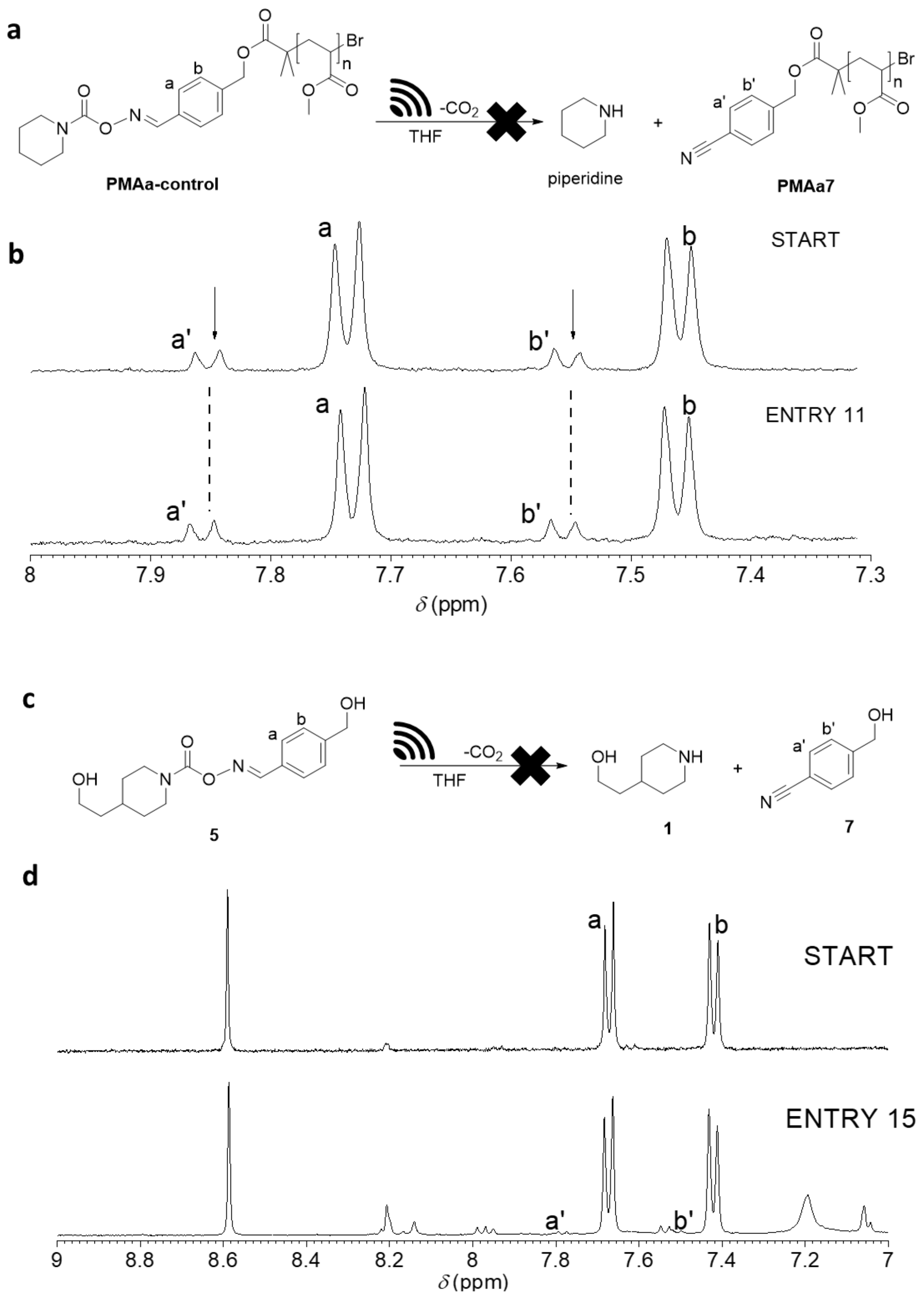


**Figure S32.** (a) Mechanochemically inaccessible reaction scheme for the sonication of **PMA14**. (b) $^{1}$H-NMR spectra comparison in DMSO-$d_6$ zoomed in between 7.3 and 8 ppm. From top to bottom: pristine **PMA14**, **PMA14** sonicated in THF for 2 h (Entry 11 of Table S3). (c) Mechanochemically inaccessible reaction scheme for the sonication of **5**. (d) $^{1}$H-NMR spectra comparison in DMSO-$d_6$ zoomed in between 7 and 9 ppm. From top to bottom: pristine **5**, **5** sonicated in THF for 3 h at 60% amplitude (Entry 15 of Table S3).

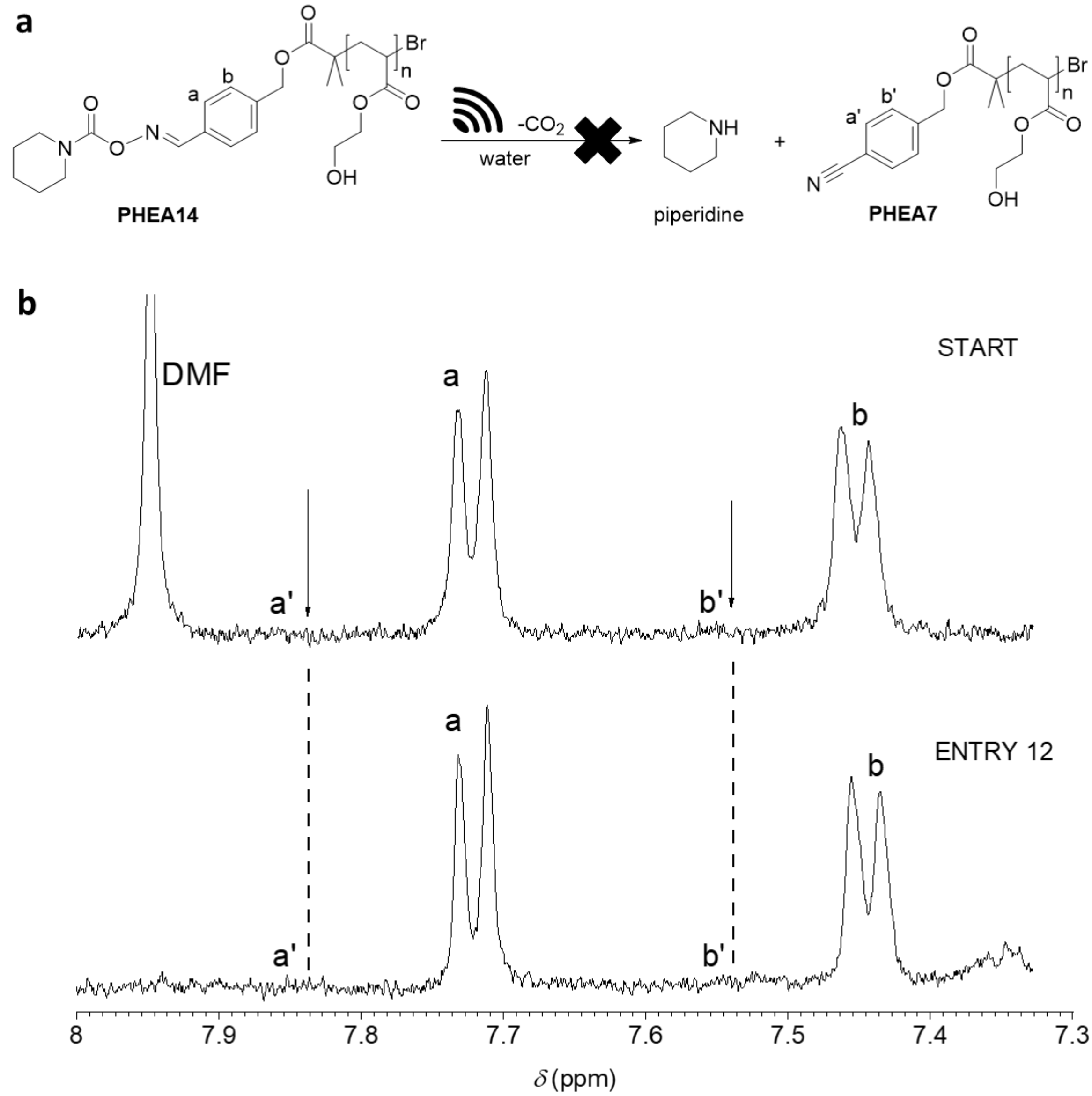


**Figure S33**. (a) Mechanochemically inaccessible reaction scheme for the sonication of **PHEA14**. (b) $^1$H-NMR spectra comparison in DMSO-$d_6$ zoomed in between 7.3 and 8 ppm. From top to bottom: pristine **PHEA14**, **PHEA14** sonicated in $H_2O$ for 2 h (Entry 12 of Table S3).

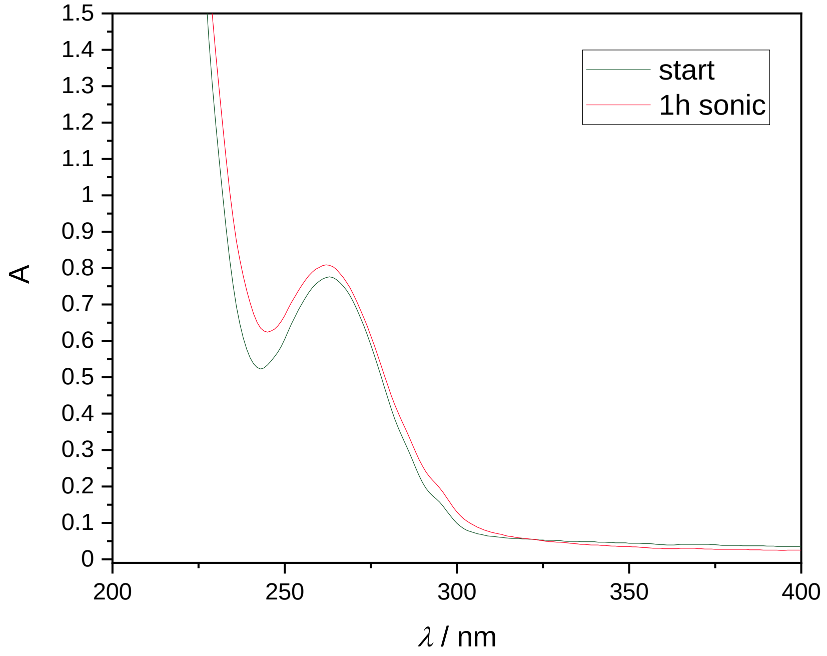


**Figure S34.** UV-vis absorption spectra of **PHEA14** (2.5 mg·mL$^{-1}$) before and after sonication (Entry 12 of Table S3).

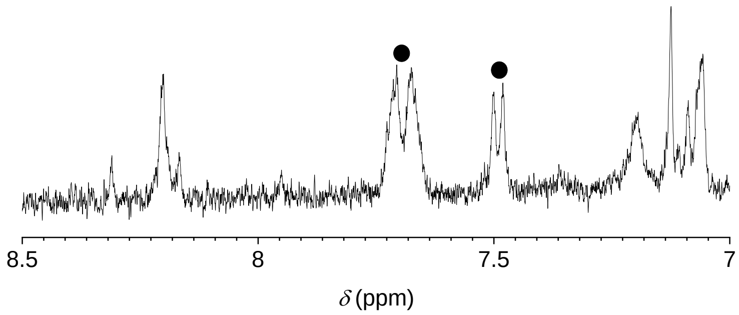


**Figure S35.** $^{1}$H-NMR spectrum in DMSO-$d_6$ zoomed in between 7 and 8.5 ppm of the sonicated **PMA$_{139}$** (Entry 14 of Table S3)

## 2.6. Computational experiments

The qualitative simulation was carried out by CoGEF method, applied according to literature.[10] Gaussian 09 software package with GaussView implementation were used to perform the study. DFT (density functional theory) was the quantum mechanical modelling method selected, and it was used at the B3LYP/6-31G* level of theory (Figure S36a). First, the drawn model was geometrically optimized without any constraints, obtaining the most stable local conformer. Afterwards, as starting step of the CoGEF procedure, two anchor atoms were selected, in this case the $sp^3$ carbon atoms of the methyl ester functional groups (blue atoms in Figure S36a). The distance between these two atoms was increased by a certain $\Delta d$, then frozen, and the geometry was optimized again, obtaining a structure slightly more constrained and with higher energy. Therefore, the distance increase-freeze-optimization cycle was repeated multiple times, varying the $\Delta d$ from a maximum of 1 Å in the flat region of the energetic profile (Figure S36b) to a minimum of 0.1 Å in the most steep region. This iteration was repeated until the breakage of the oxime bond was observed (model image in Figure S36b, O in red and N in blue), that also corresponded to a sudden drop of the energy value. Small energy drops along the energy profile were attributed to conformational variations.

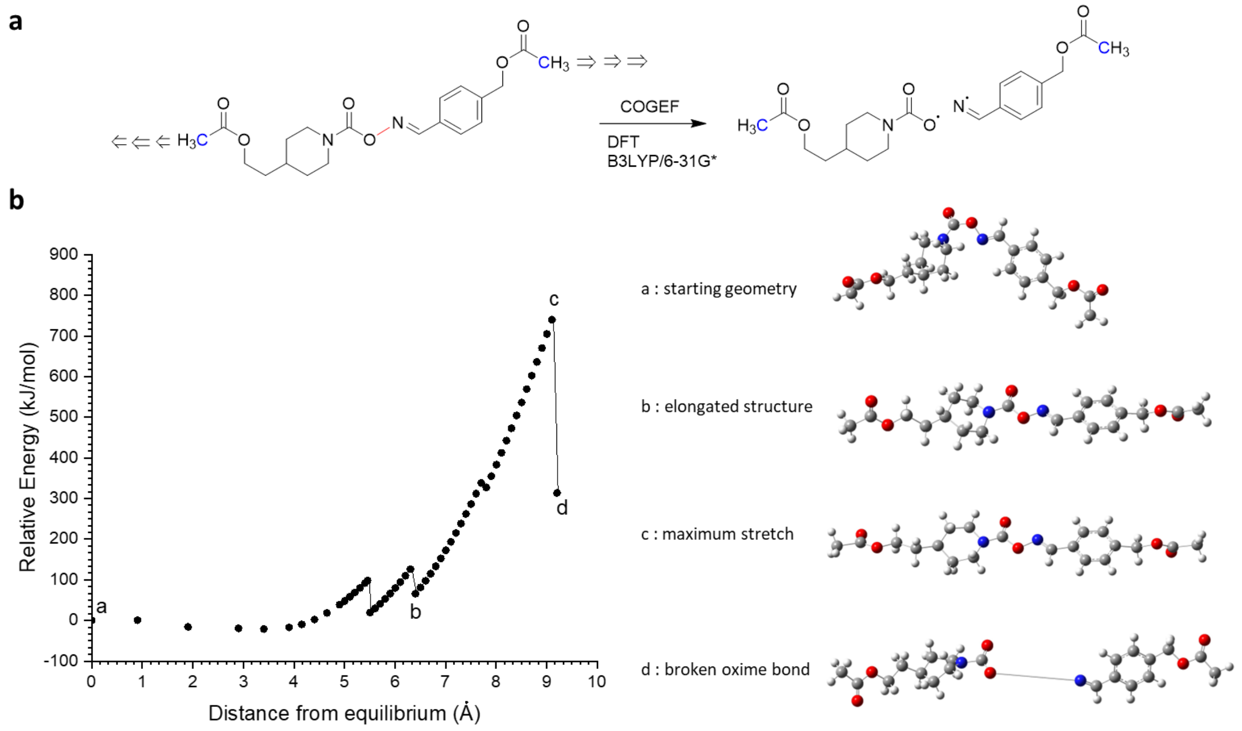


**Figure S36.** (a) Simulation scheme (anchor atoms in blue, bond broken in red). (b) Energy profile of the CoGEF simulation and summary of the data obtained (left), ball and stick models (N in blue, O in red) at various stages of the curve (right).

## 2.7. Derivatization with RhBITC

The labelling reaction between the allegedly formed amine **PMA1** after irradiation and/or sonication of **PMA6** and Rhodamine B isothiocyanate (**RhBITC**) was first optimized with small molecules using piperidine (Scheme S1).

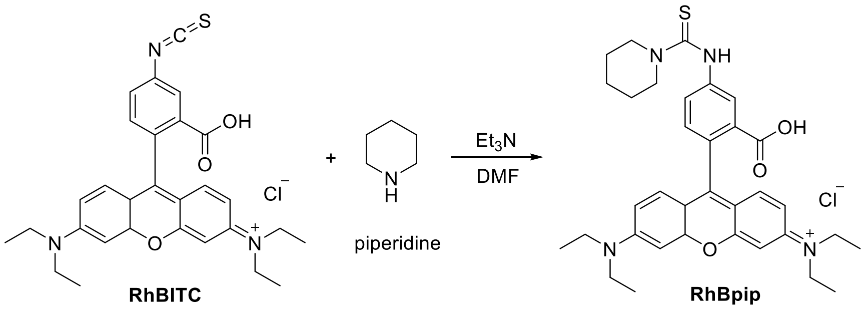


**Scheme S1**. **RhBITC** forms a stable thiourea **RhBpip** with piperidine.

Piperidine (0.58 µL, 5.87 µmol, 1 equiv.), **RhBITC** (3.16 mg, 1 equiv.), and $Et_3N$ (0.82 µL, 1 equiv.) were dissolved in dry DMF (5 mL). The mixture was stirred at r.t. for 5 h, then all the volatiles were removed *in vacuo*, and the final residue was re-dissolved in MeCN (1 mL). To directly evaluate the formation of **RhBpip**, samples were withdrawn and subjected to RP-UPLC (Figure S37), $ESI^+$ MS (Figure S38), and UV-vis absorption spectroscopy (Figure S39). All analyses were performed in comparison with **RhBITC**. The results prove the successful formation of the product, verifying the reaction conditions. Notably, when the isothiocyanate group has reacted with the amine to form the thiourea, a significant decrease of the molar absorptivity at 558 nm can be observed.

Hereafter, these conditions were applied for labeling **PMA1** after irradiation and sonication of **PMA6**. For the former, irradiation in THF was performed (Entry 9 of Table S2), the solvent removed *in vacuo*, and the final polymer residue (5 mg) re-dissolved in dry DMF (1 mL). In the latter case, 2 mL were taken from the sonicated sample in THF/$H_2O$ 7:3 (v/v) (Entry 7 of Table S3), the solvent removed *in vacuo*, and the polymer (5 mg) re-dissolved in dry DMF (1 mL). Afterwards, **RhBITC** (0.34 mg, 0.62 µmol, 10 equiv. per end group) and $Et_3N$ (0.087 µL, 10 equiv. per end group) were added and left stirring overnight at r.t. Then, the solvents were removed *in vacuo*, the residues re-dissolved in THF (for GPC, 1 mL), and submitted to GPC analysis monitoring the UV absorption channel at 351 nm. The results are discussed in the main manuscript (*cf.* Figure 2). The high noise of the UV-vis signals reflect the intrinsic low sensitivity of the UV-vis detector of the GPC and the likely low yield of the labeling reaction due to the poor end group reactivity. Therefore, the detection of the amine **PMA1** could only be verified qualitatively.

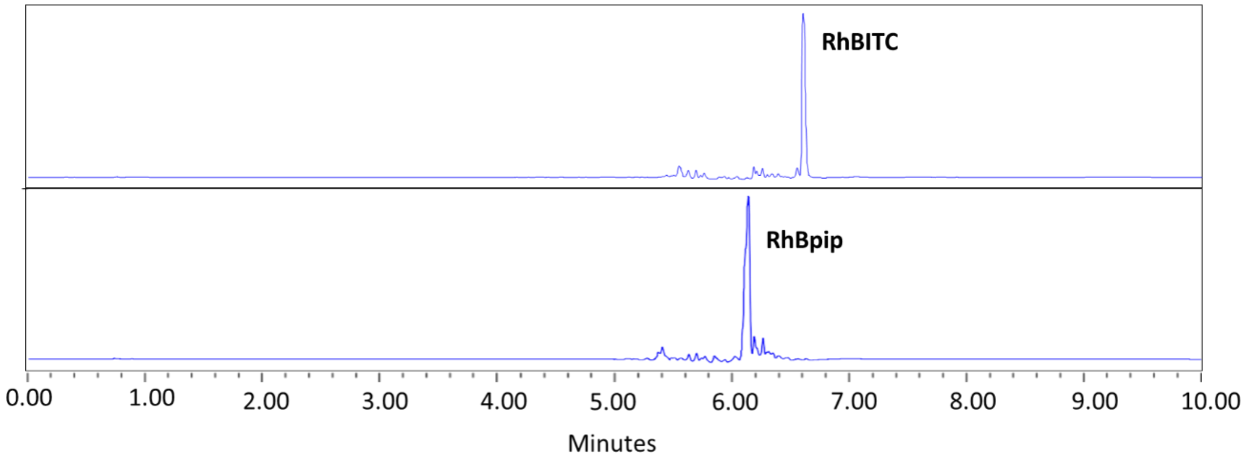


**Figure S37.** RP-UPLC chromatogram with eluent gradient $H_2O$/MeCN (95:5) to $H_2O$/MeCN (5:95). From top to bottom: only **RhBITC**, product **RhBpip**.

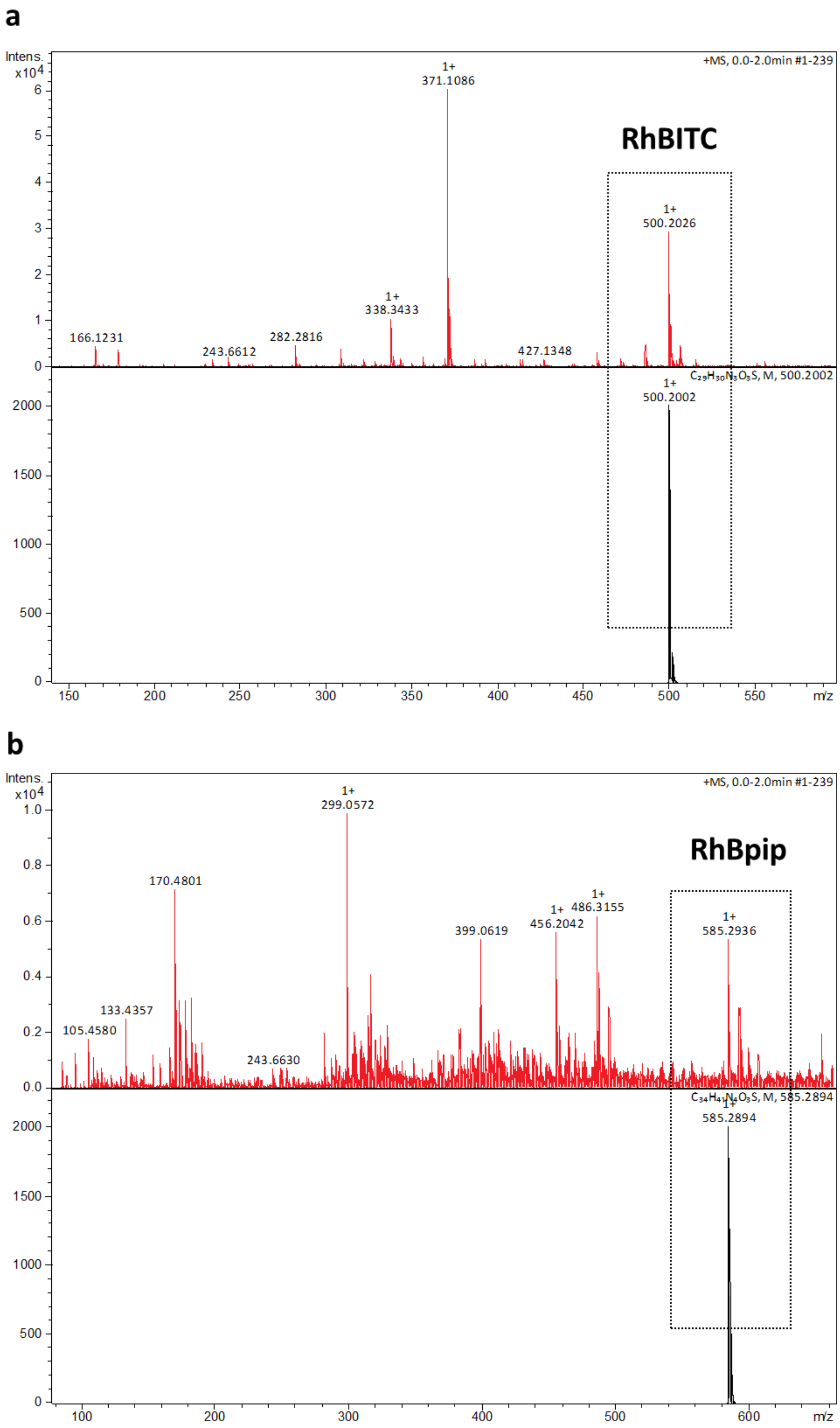


**Figure S38.** ESI$^+$ MS spectra of (a) **RhBITC** and (b) **RhBpip**. Real spectra (red) and simulated (black).

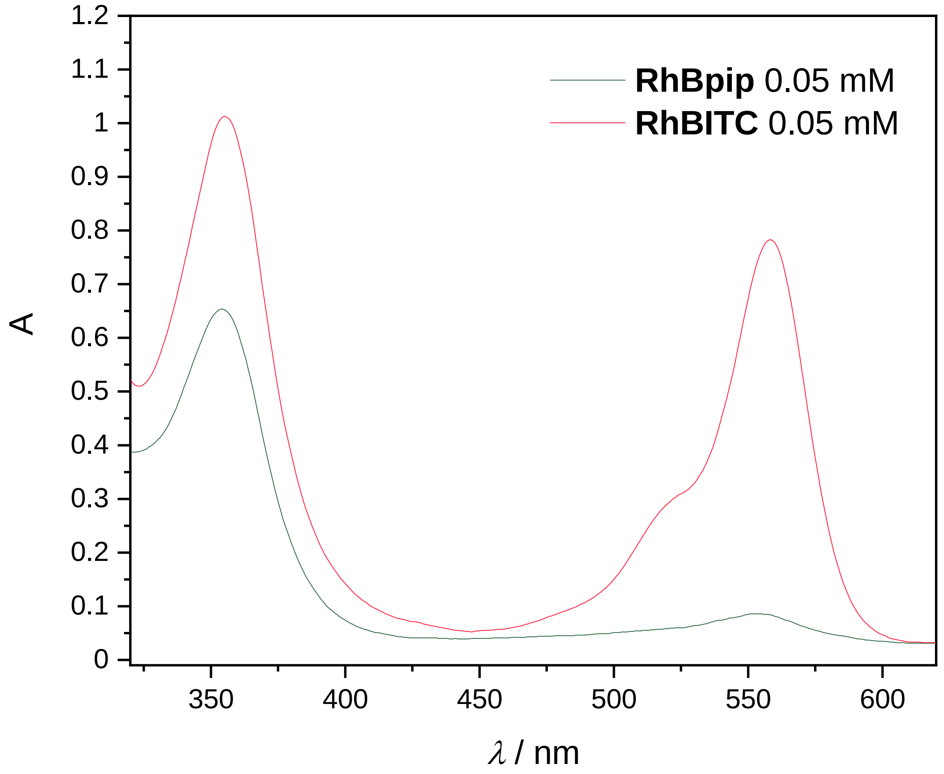


**Figure S39.** UV-vis absorption spectra of **RhBITC** (red line) and **RhBpip** (green line) in MeCN.

## 2.8. Organocatalytic experiments

### 2.8.1. General procedure and list of experiments

Prior to conducting the Knoevenagel reaction with the mechanophore it was activated by irradiation with light or ultrasonication under certain conditions.

The irradiation trials were conducted through a standard procedure similar to the previous photochemical experiments (*cf.* Section 2.4.2): a certain amount of sample was placed in a quartz cuvette and irradiated with the high or low intensity light source (*cf.* Section 1.4). The multiple experiments are summarized in Table S5. Every sample was dissolved in THF and after the irradiation period an aliquot was taken and the solvent was removed *in vacuo*. The residue was stored and used for the subsequent catalytic experiments.

The sonication experiments were also performed similarly to the previous mechanochemical experiments (*cf.* Section 2.5.1), but with some important variations. The trials are summarized in Table S6. Every sample was dissolved in approximately 40 mL THF and the sonication was run for 3 h ("on" time) at 60% amplitude to significantly increase the kinetics of the scission and therefore activating larger amounts of polymer. For some trials, additional 200 µL of *n*-butylamine were added to the solution prior sonication. The motivations behind this experimental setup are discussed in detail in the Section 2.8.3. After the sonication, every sample was filtered through a PTFE membrane (0.45 µm) and the solution was dialyzed with an RC (regenerated cellulose) membrane (1 kDa) overnight against ca. 0.5 L MeCN to remove most of the n-butylamine excess. In the end, the solvent and most of the leftover n-butylamine were removed *in vacuo* and the residue was stored and used for the subsequent catalytic experiment. For the calculation of the sonicated sample concentration in the catalytic mixture, it was assumed the all polymer was retained inside the membrane. Therefore, the possible amount of polymer that leaked out of the membrane was neglected.

**Table S5.** List of the light irradiation experiments. The products were used for the catalysis experiments reported in Table S7.

| entry | compound no. | Irradiation source and time / min | *c* / mM | *ρ* / mg·mL$^{-1}$ | *V* / mL | *V* used for catalysis / mL |
|---|---|---|---|---|---|---|
| 1 | THF | high intensity 30 | - | - | 3.00 | 3.00 |
| 2 | **5** | low intensity 420 | 0.33 | 0.10 | 2.00 | 2.00 |
| 3 | **5** | high intensity, 20 | 3.26 | 1.00 | 1.00 | 1.00 |
| 4 | **5** | high intensity 5 | 1.08 | 0.33 | 1.00 | 0.60 |
| 5 | **5** | high intensity 5 | 0.33 | 0.10 | 2.00 | 1.00 |
| 6 | **PMA$_{94}$** | low intensity 420 | 0.31 | 29.14 | 2.00 | 2.00 |
| 7 | **PMA$_{119}$** | high intensity 10 | 0.85 | 101.15 | 3.00 | 3.00 |
| 8 | **PMA6$_{134}$** | low intensity 420 | 0.35 | 46.90 | 2.00 | 2.00 |
| 9 | **PMA6$_{116}$** | high intensity 10 | 0.87 | 100.92 | 3.00 | 3.00 |

**Table S6.** List of the ultrasonication experiments. The products were used for the catalysis experiments reported in Table S8.

| entry | compound no. | solvent | sonicated *m* / mg |
|---|---|---|---|
| 1 | **$PMA6_{145}$** | THF | 300 |
| 2 | 4-piperidinethanol **1** | THF | 0.23 |
| 3 | **$PMA6_{116}$** | THF + *n*-butylamine | 300 |
| 4 | **$PMA6_{116}$** | THF + *n*-butylamine | 300 |
| 5 | **$PMA6_{116}$** | THF + *n*-butylamine | 300 |
| 6 | **$PMA_{42}$** | THF + *n*-butylamine | 300 |
| 7 | **$PMA_{42}$** | THF + *n*-butylamine | 300 |
| 8 | **$PMA_{42}$** | THF + *n*-butylamine | 300 |

Regarding the subsequent Knoevenagel reaction, the reaction conditions and experiment setup were reproduced similarly to a reported study.[11] $CDCl_3$ was selected to monitor the catalytic conversion directly by submitting the reaction mixture to NMR analysis without any additional purification steps. Every experiment was carried out with the same procedure: the stored irradiated or sonicated isolated sample was dissolved in 0.6 mL of $CDCl_3$ and the solution was then mixed with 3 mg of 4-nitrobenzaldehyde **18** giving a final concentration of 33 mM. Then, 50 equiv. of diethyl malonate **19** were added (152 µL) to obtain pseudo first-order kinetics. The mixture was stirred at room temperature and after 2 h (and 4h) and aliquot of 200 µL was removed, diluted with 0.4 mL of $CDCl_3$, and submitted to NMR analysis with 128 scans when a more zoomed view of the cleaved mechanophore was required. A summary of the photochemically and mechanochemically triggered catalytic experiments is shown in Table S7 and Table S8. The entry numbers of the catalysis samples are the same as the related treated sample in Table S5 and Table S6. Additionally, Table S9 reports important catalytic control experiments performed. In this case, the 4-nitrobenzaldehyde **18** was first dissolved in $CDCl_3$ and then the respective compound was added.

$^1$H-NMR spectroscopy was used to calculate conversions after light irradiation and ultrasonication to the active species and also to calculate catalytic conversion of the Knoevenagel reaction (Table S7 and Table S8, Figure S40 to Figure S45). A full exemplary spectrum of the Knoevenagel reaction is shown in Figure S40 (Entry 1 of Table S9) where the conversion can be calculated by comparing the integrals of the aromatic peaks of 4-nitrobenzaldehyde **18** and the coupling product **20** (signals c-b vs h-g in Figure S40). Therefore, all other spectra are zoomed in the diagnostic aromatic region. The calculation of mechanophore conversion in in Table S7 and Table S8 was carried out by comparing the integrals of the visible diagnostic aromatic signals of **5** (δ = 7.63 ppm) with the produced nitrile **7** (δ = 7.43 ppm) visible in the zoomed in spots in Figure S41c-f. In case of irradiation or sonication of **PMA6**, the signals taken into account were for **PMA6** at δ = 7.67 ppm while for the produced end group nitrile **PMA7** at δ = 7.63 ppm.

**Table S7.** List of the catalytic experiments after irradiation with light. The concentration $c_1$ refers to the total concentration of the mechanophore (and initiator) considering the non-cleaved and the cleaved residue. The concentration $c_2$ refers to the concentration of the cleaved mechanophore calculated through the irradiation conversion.

| entry in Table S5 | compound no. | $c_1$ / mM | irradiation conversion determined by NMR / % | $c_2$ / mM | catalytic conversion after 2 h by NMR / % |
|---|---|---|---|---|---|
| 1 | THF only | - | - | \ | 5 (Figure S41a) |
| 2 | **5** | 1.08 | 46 | 0.49 | 23 (Figure S41c) |
| 3 | **5** | 5.50 | 75 | 4.11 | 52 (Figure S41d) |
| 4 | **5** | 1.08 | 78 | 0.84 | 26 (Figure S41e) |
| 5 | **5** | 0.55 | 100 | 0.55 | 8 (Figure S41f) |
| 6 | **$PMA_{94}$** | 1.04 | - | \ | 7 (Figure S42a) |
| 7 | **$PMA_{119}$** | 4.20 | - | \ | 5 (Figure S42b) |
| 8 | **$PMA6_{134}$** | 1.15 | 38 | 0.44 | 34 (Figure S42c) |
| 9 | **$PMA6_{116}$** | 4.30 | 43 | 1.85 | 46 (Figure S42d) |

**Table S8.** List of the catalytic experiments after ultrasonication. The concentration $c_1$ refers to the total concentration of the mechanophore (and initiator) considering the non-cleaved and the cleaved residue. The concentration $c_2$ refers to the concentration of the cleaved mechanophore calculated through the sonication conversion.

| entry in Table S6 | compound no. | $c_1$ / mM | sonication conversion determined by NMR / % | $c_2$ / mM | catalytic conversion after 2 h by NMR / % | catalytic conversion after 4 h by NMR / % |
|---|---|---|---|---|---|---|
| 1 | **$PMA6_{145}$** | 3.60 | not detectable | - | 11 (Figure S43a) | - |
| 2 | 4-piperi-dinethanol **1** | 3.00 | - | - | 16 (Figure S43b) | - |
| 3 | **$PMA6_{116}$** | 4.50 | 52 | 2.35 | 26 (Figure S44a) | 38 (Figure S44b) |
| 4 | **$PMA6_{116}$** | 4.50 | 58 | 2.61 | 45 (Figure S44c) | 60 (Figure S44d) |
| 5 | **$PMA6_{116}$** | 4.50 | 56 | 2.54 | 63 (Figure S44e) | 68 (Figure S44f) |
| 6 | **$PMA_{42}$** | 12.00 | - | - | 2 (Figure S45a) | 2 (Figure S45b) |
| 7 | **$PMA_{42}$** | 12.00 | - | - | 1 (Figure S45c) | 4 (Figure S45d) |
| 8 | **$PMA_{42}$** | 12.00 | - | - | 1 (Figure S45e) | 1 (Figure S45f) |

**Table S9.** List of the catalytic control experiments

| entry | compound no. | c / mM | catalytic conversion after 2 h by NMR / % |
|---|---|---|---|
| 1 | 4-piperidinethanol **1** | 1.00 | 60 (Figure S40) |
| 2 | *n*-butylamine | 30.00 | 15 (Figure S43c) |
| 3 | **5** | 5.50 | 7 (Figure S41b) |

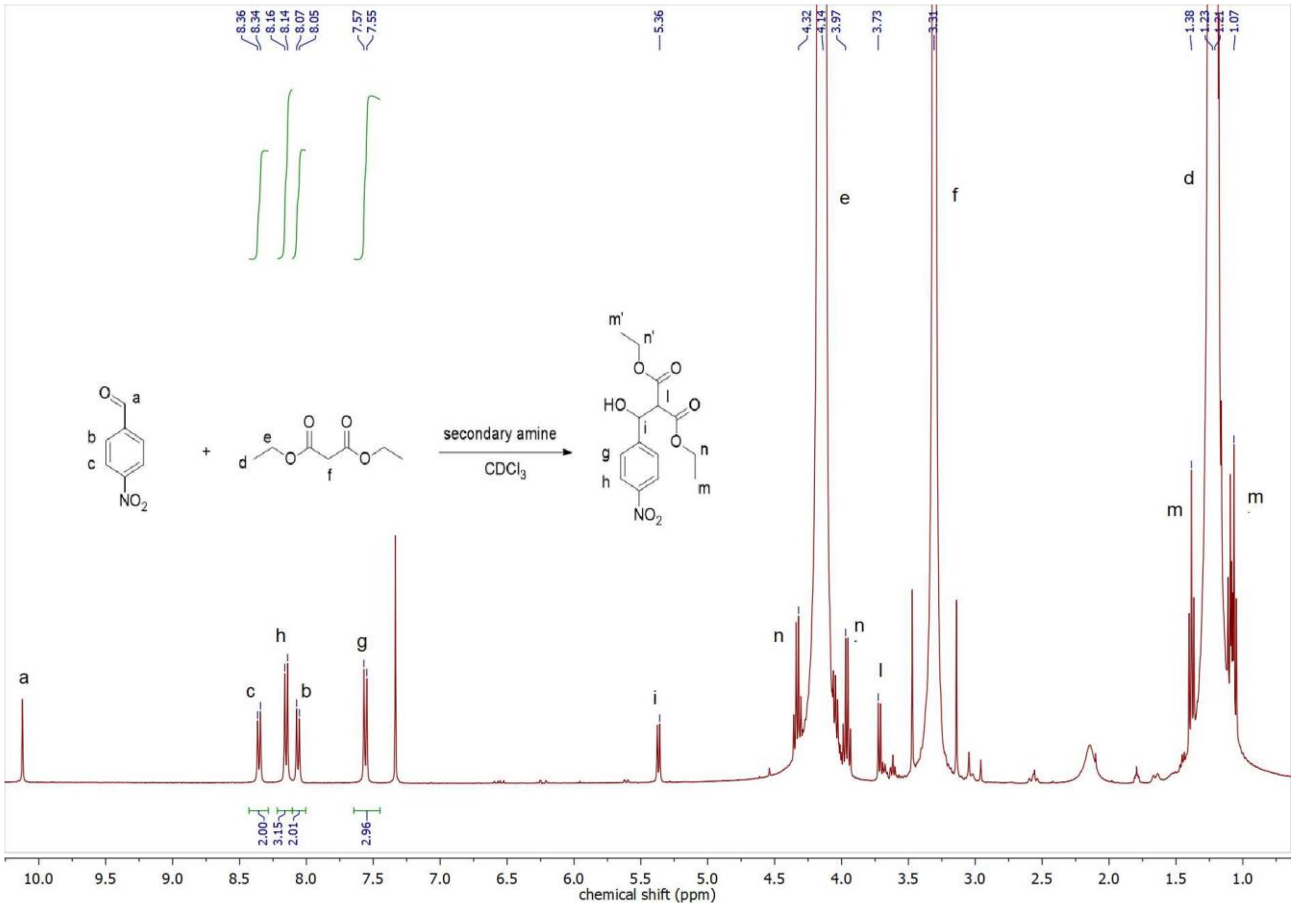


**Figure S40.** $^1$H-NMR spectrum of Entry 1 in Table S9. Exemplary analysis showing the complete assignments of the mixture containing a partial conversion of the 4-nitrobenzaldehyde **18** to the coupling product **20** with excess of the diethyl malonate **19**.

## 2.8.2. Photochemically induced organocatalysis

The $^1$H-NMR spectra of the catalytic experiments involving the photochemical activation of **5**, in comparison to negative controls, were recorded according to the general procedure (*cf.* Section 2.8.1) and are shown in Figure S41. By comparing the results in different conditions, it was observed that the high intensity light source could trigger more light-induced side reactions which reduce it final amine yield. This could be partially avoided either by increasing the irradiation concentration or by using the low intensity light source. Similar experiments were then carried out with the mechanophore-centered **PMA6**. The related NMR spectra, against negative controls, were recorded according to the general procedure (*cf.* Section 2.8.1) and are summarized in Figure S42.

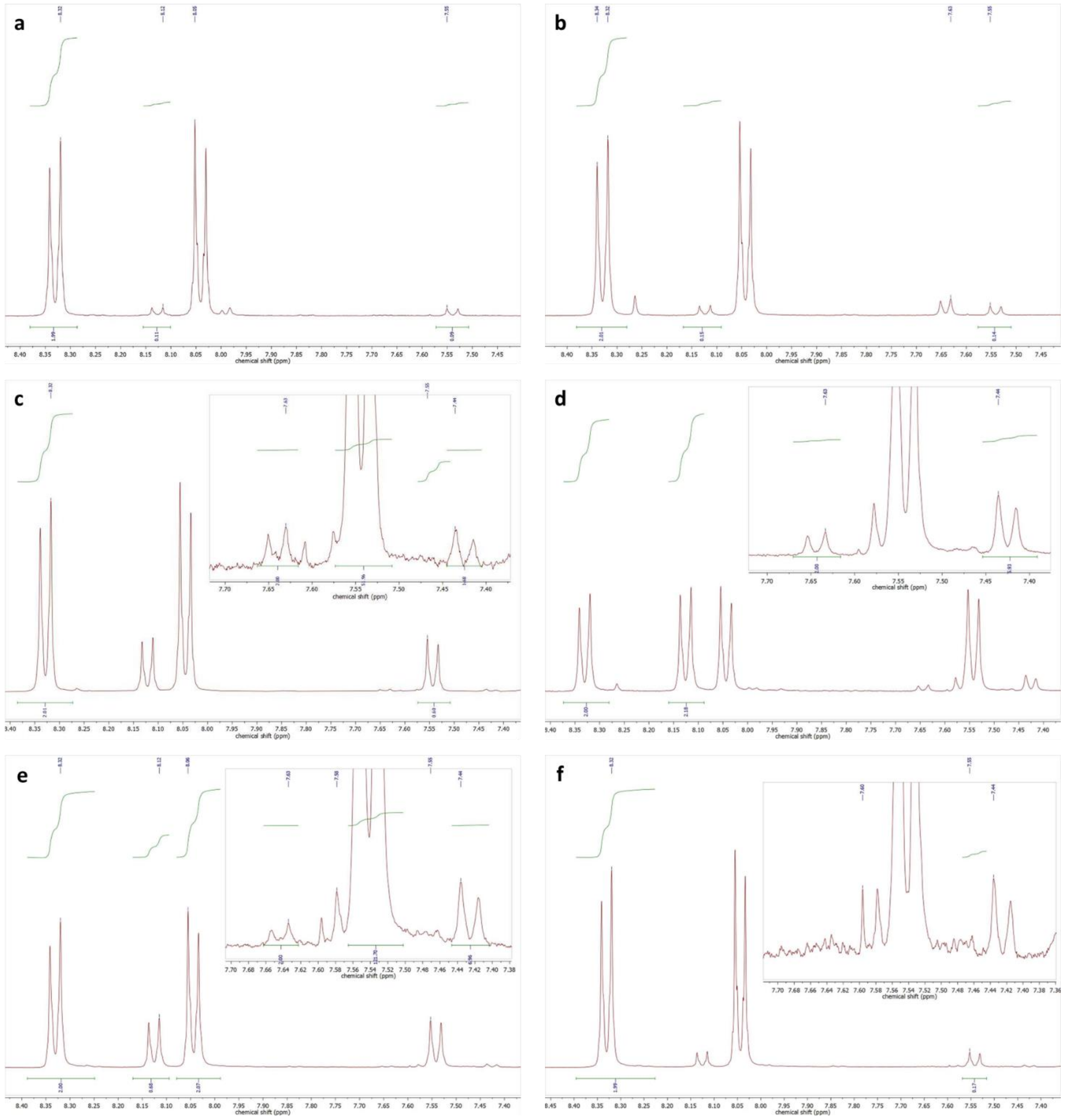

**Figure S41.** $^{1}$H-NMR spectra of different catalytic experiments involving the photochemically activated small mechanophore precursor **5**. The zoomed in regions evidence the irradiation conversion of **5**. (a) Negative control pure THF (Entry 1 of Table S7). (b) Negative control pristine **5** not irradiated (Entry 3 of Table S9). (c-d) Entry 2 and 5 of Table S7. Cleaved mechanophore **5** at different irradiation and catalytic concentrations and with different light sources.

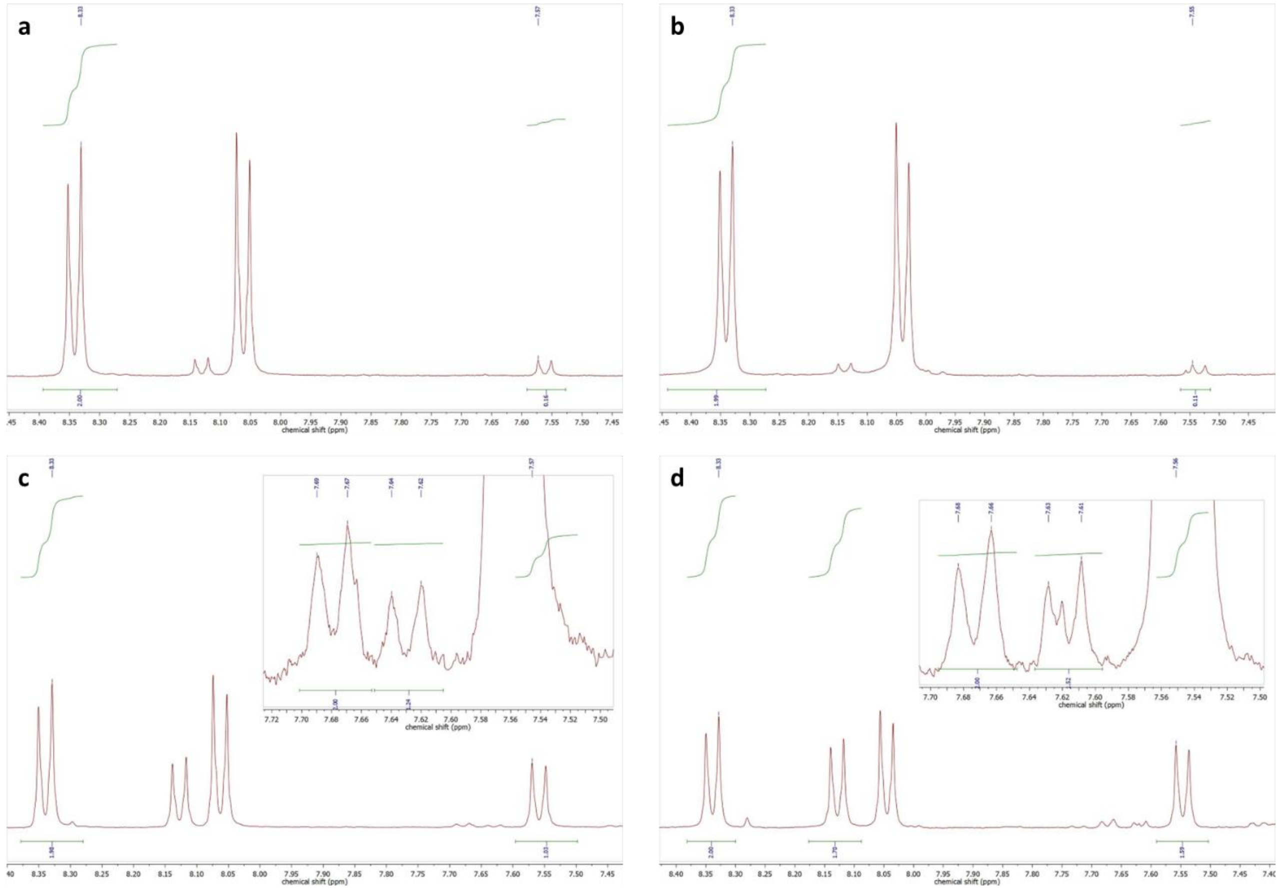


**Figure S42.** $^{1}$H-NMR spectra of different catalytic experiments involving the photochemically activated mechanophore-centered **PMA6**. The zoomed in regions evidence the irradiation conversion of the mechanophore scaffold of **PMA6**. (a) negative control irradiated **PMA**$_{94}$ with low intensity source (Entry 6 of Table S7). (b) Negative control irradiated **PMA**$_{119}$ with high intensity source (Entry 7 of Table S7). (c) Mechanophore irradiated **PMA6**$_{134}$ with low intensity source (Entry 8 of Table S7). (d) Mechanophore irradiated **PMA6**$_{116}$ with high intensity source (Entry 9 of Table S7).

### 2.8.3. Mechanochemically induced organocatalysis

A small amount of *n*-butylamine was used as scavenger of acids/electrophiles residues produced during sonication. Figure S43 reports the $^{1}$H-NMR spectra of some key control experiments recorded according to the general procedure (*cf.* Section 2.8.1). The catalytic experiment carried out with the sonicated pristine 4-piperidinethanol **1** in pure THF (Entry 2 of Table S6 and Table S8) shows low conversion (16%) suggesting that some residues of acids or electrophiles have been produced during the sonication and partially quenched the amine reducing its final catalytic concentration. One possible theory is that the sonication causes a very small decomposition of the glass surface resulting in the dissolution of some silica particles bearing slightly acidic silanol groups. To investigate more, solvent was removed from a pure THF sonicated solution and the residue was redissolved in water for pH measurements, as comparison, a similar water sample was prepared with a non-sonicated THF solution. However, given a non-excellent sensitivity of the instrument, no significant difference in the average pH values was obtained. Assuming that the issue is related to acidic impurities rather than electrophiles, we believe that amount of these impurities would be enough to affect the activity of very lowly concentrated amines, but without manifesting a significant pH changes when these impurities are in aqueous environment. In the future, more precise measures could be done, for instance using spectroscopic analysis of very sensitive pH indicators.

In fact, the catalytic experiments performed with sonicated **PMA6** in pure THF (Entry 1 of Table S8) also showed a low conversion (11%). Therefore, the solution that was found was to use *n*-butylamine to maintain slightly basic conditions preventing the possible quenching of the secondary amine produced during the cleavage of the

sonication. As primary amine, *n*-butylamine cannot catalyze the Knoevenagel reaction thus not affecting the reliability of the eventual results. Even in very high concentrations, its presence in the mixture containing the Knoevenagel reagents (Entry 2 of Table S9) yields the related imine upon reaction with 4-nitrobenzaldehyde **18**, while the presence of the coupling product **20** is only observed in low amount (15% conversion).

The subsequent $^1$H-NMR spectra of the catalytic experiments performed with sonicated **PMA6$_{116}$** and **PMA$_{42}$** in THF containing *n*-butylamine were recorded according to the general procedure (*cf.* Section 2.8.1) and are reported in Figure S44 and Figure S45.

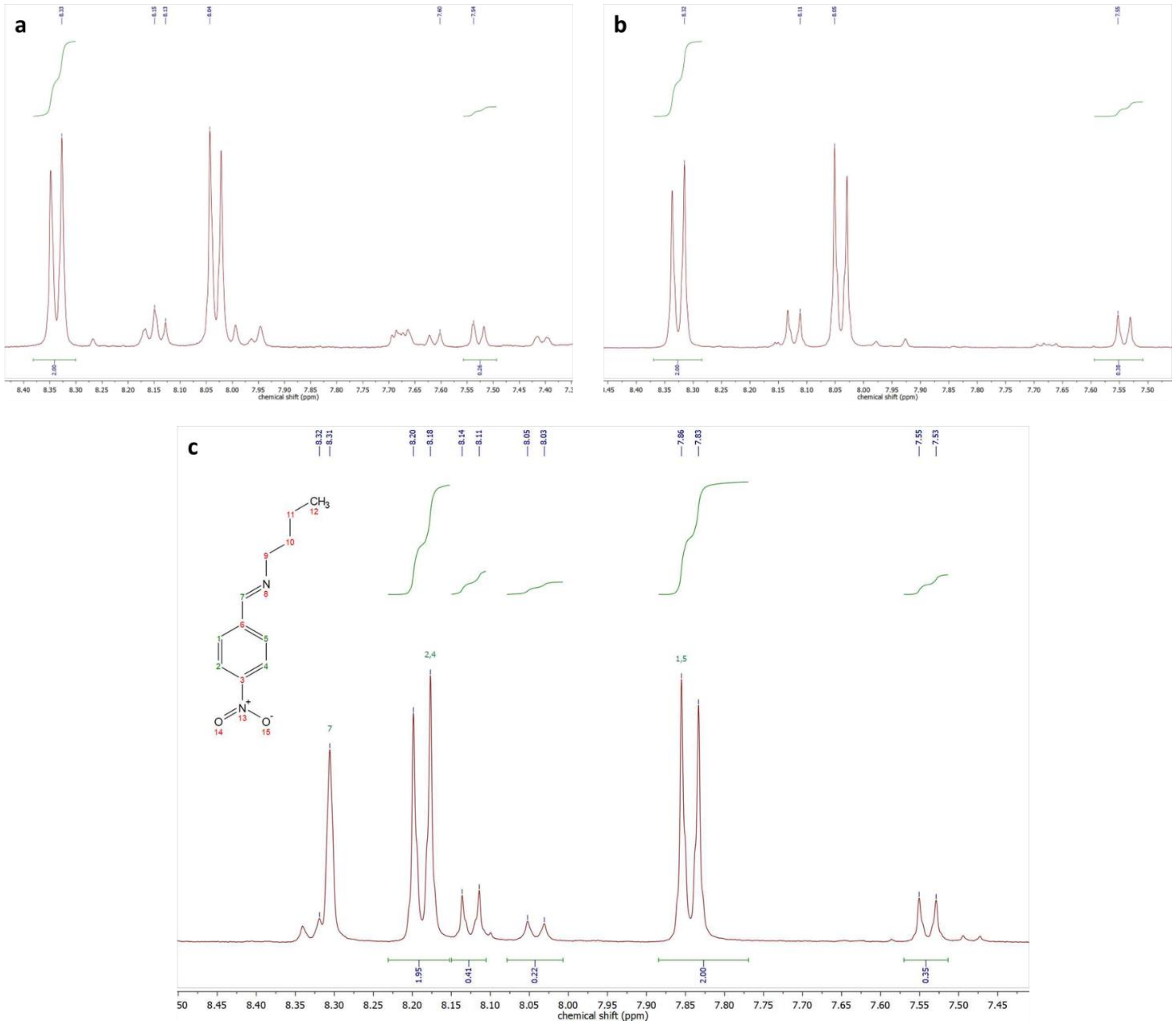


**Figure S43.** $^1$H-NMR spectra of different catalytic experiments involving key sonication control experiments. (a) Sonicated mechanophore **PMA6$_{145}$** in pure THF (Entry 1 of Table S8). (b) Sonicated pristine 4-piperidinethanol **1** in pure THF (Entry 2 of Table S8). (c) Pristine *n*-butylamine (Entry 2 of Table S9).

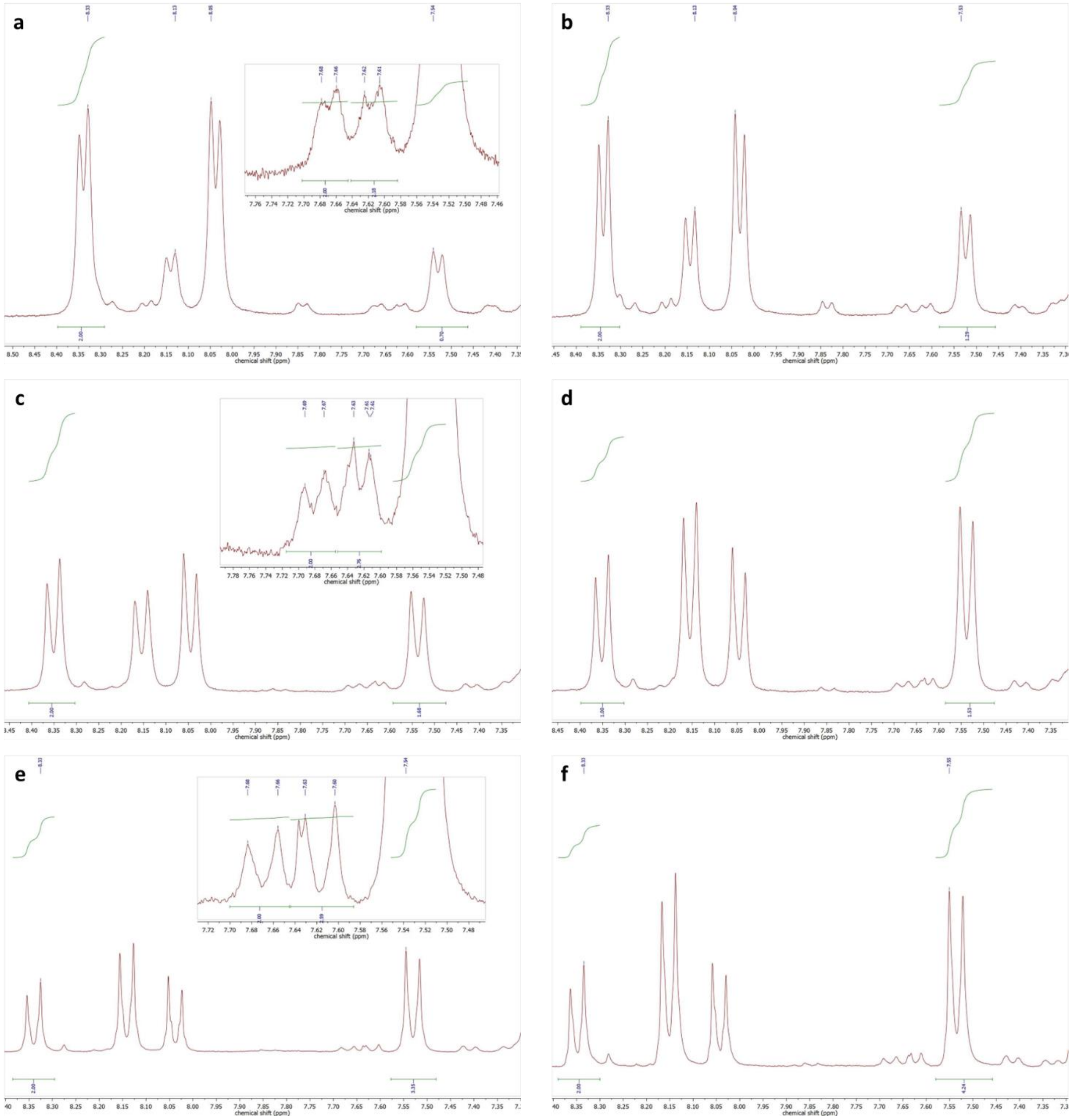


**Figure S44.** $^{1}$H-NMR spectra of different catalytic experiments involving mechanochemically activated mechanophore-centered **PMA6$_{116}$**. The zoomed in regions evidence the sonication conversion of the mechanophore scaffold of **PMA6$_{116}$**. (a-b) First iteration of sonicated **PMA6$_{116}$** after 2 h and 4 h (Entry 3 of Table S8). (c-d) Second iteration of sonicated **PMA6$_{116}$** after 2 h and 4 h (Entry 5 of Table S8). (e-f) Third iteration of sonicated **PMA6$_{116}$** after 2 h and 4 h (Entry 5 of Table S8).

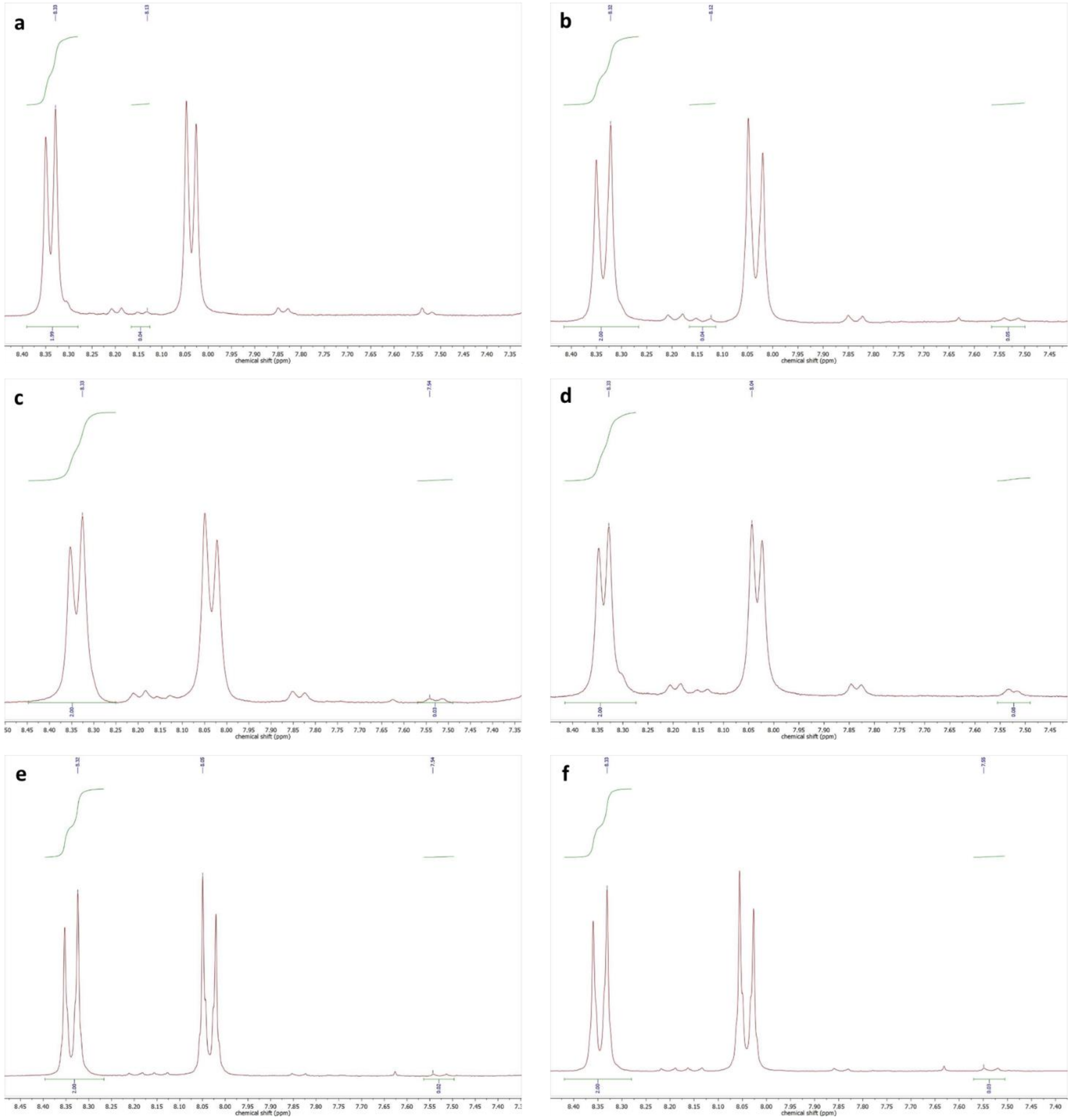


**Figure S45**. $^1$H-NMR spectra of different catalytic experiments involving sonicated **PMA$_{42}$** as negative control. (a-b) First iteration of sonicated **PMA$_{42}$** after 2 h and 4 h (Entry 6 of Table S8). (c-d) Second Iteration of sonicated **PMA$_{42}$** after 2 h and 4 h (Entry 7 of Table S8). (e-f) Third iteration of sonicated **PMA$_{42}$** after 2 h and 4 h (Entry 8 of Table S8).

# 3. Supporting NMR, MS, and GPC spectra

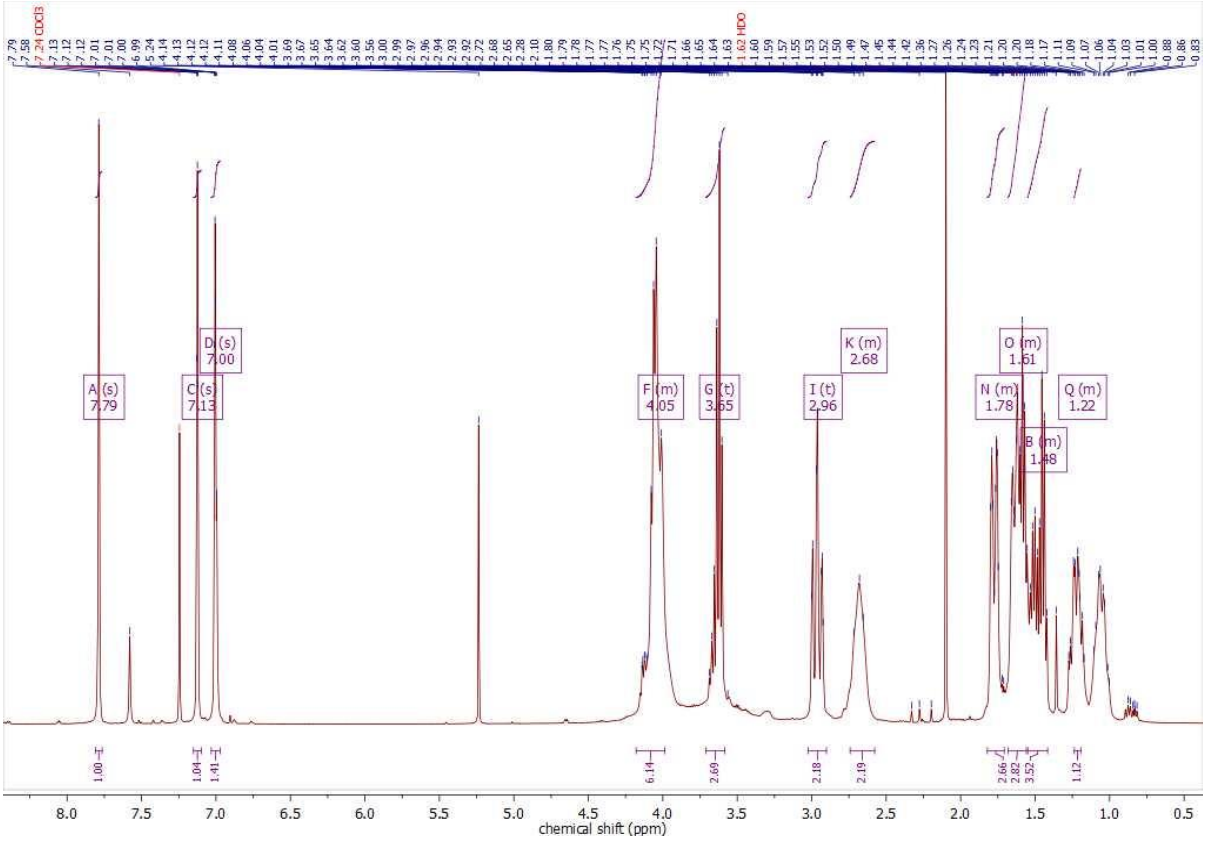


**Figure S46.** $^{1}$H-NMR spectrum of carbamoylimidazole **2**.

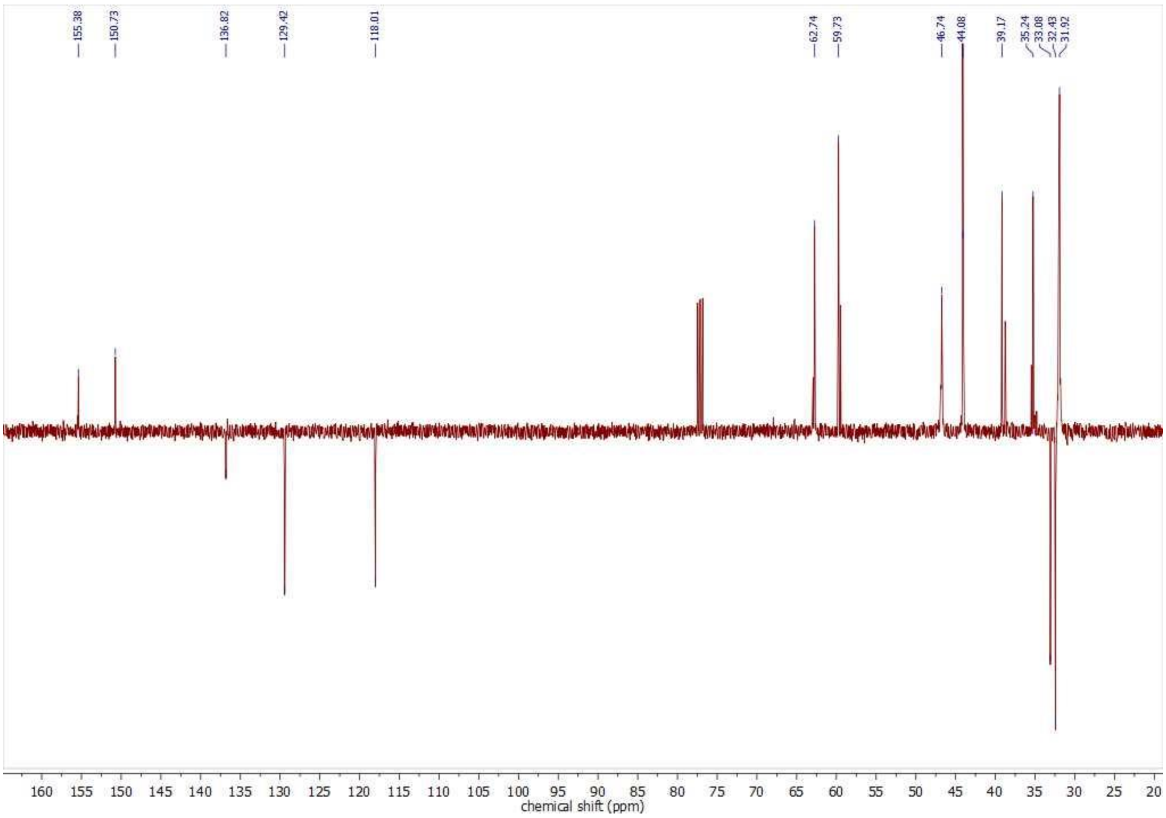


**Figure S47.** $^{13}$C-NMR spectrum of carbamoylimidazole **2**.

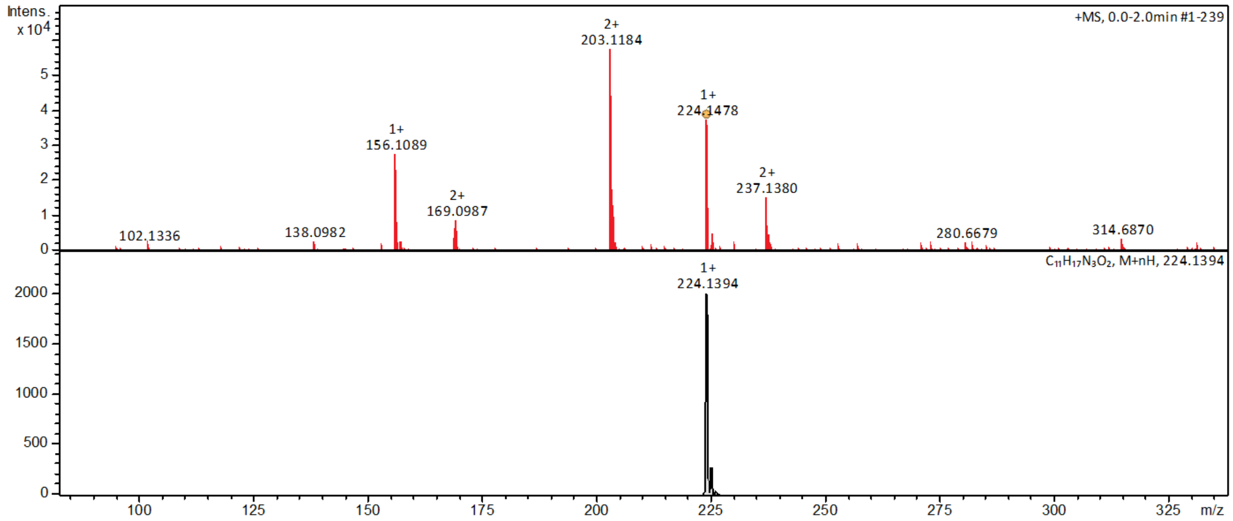


**Figure S48.** Deconvoluted ESI$^+$ HRMS (top) and calculated mass (bottom) spectra of carbamoylimidazole **2**.

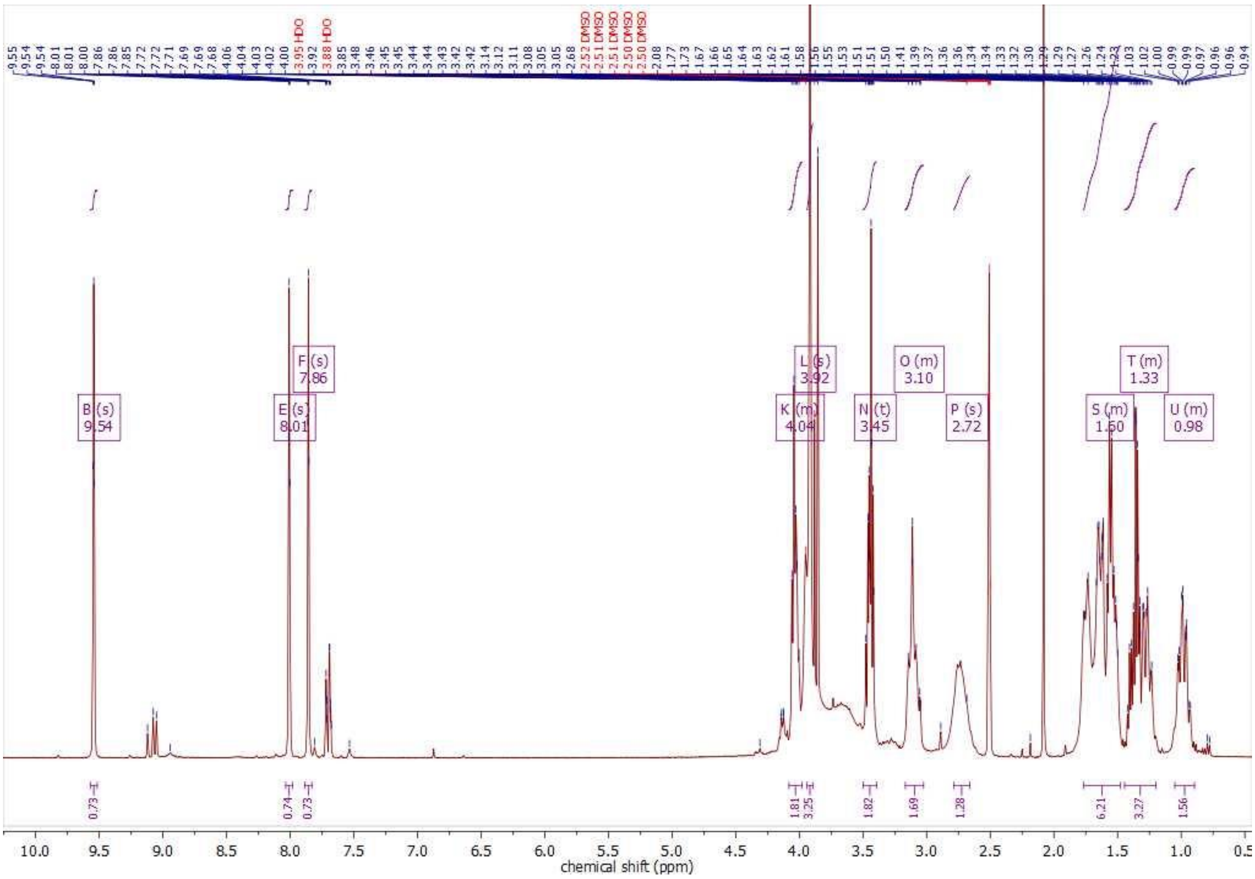


**Figure S49.** $^1$H-NMR spectrum of carbamoylimidazolium salt **3**.

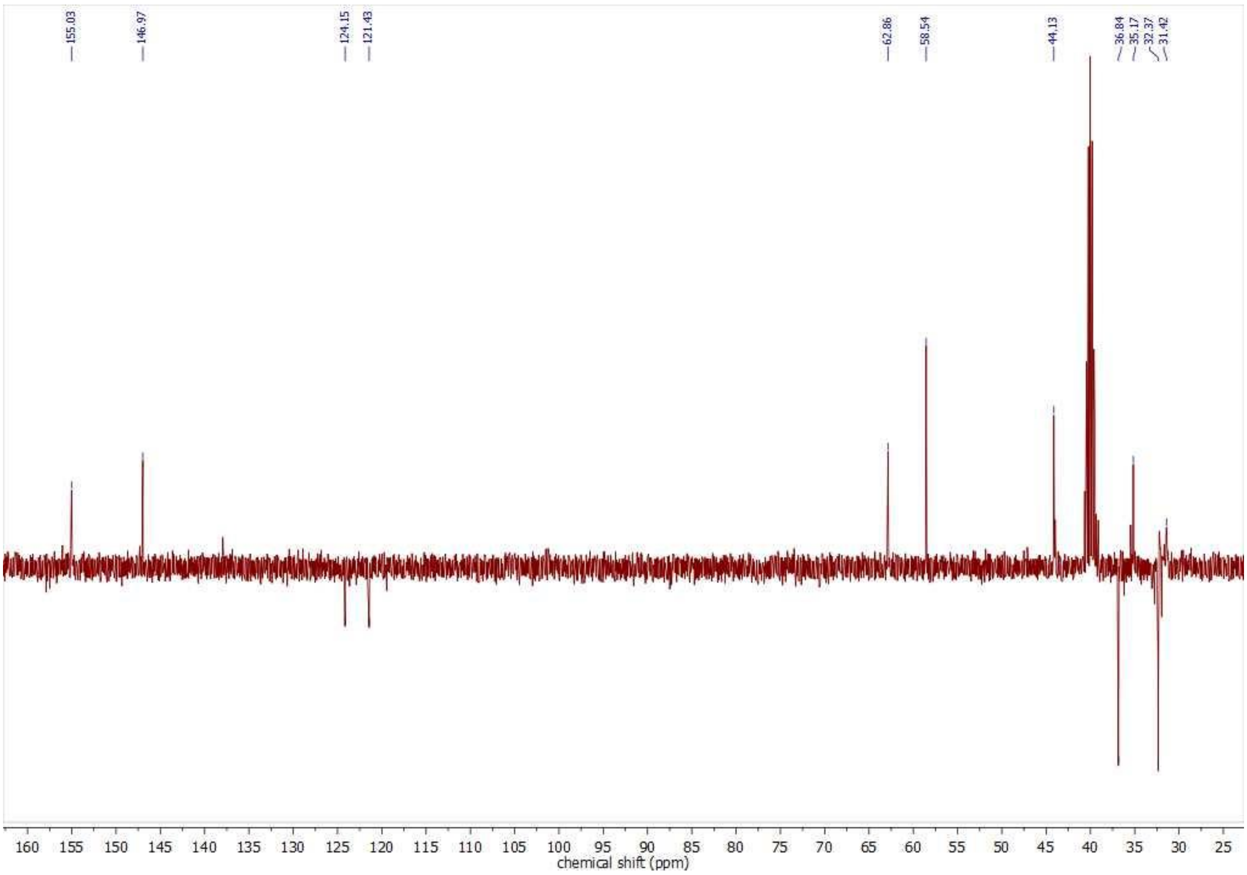


**Figure S50.** $^{13}C$-NMR spectrum of carbamoylimidazolium salt **3**.

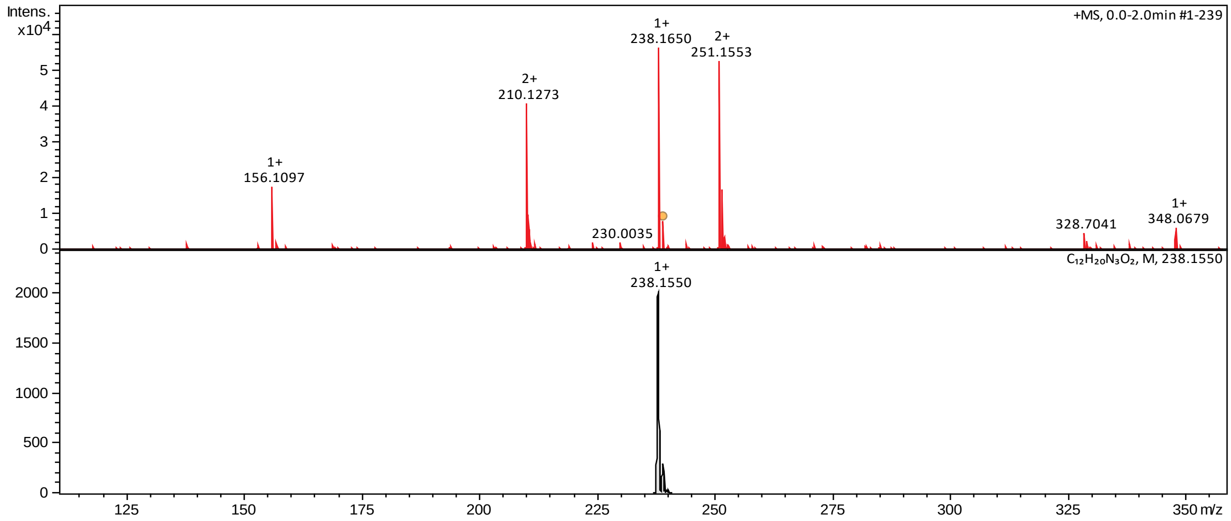


**Figure S51.** Deconvoluted ESI$^+$ HRMS (top) and calculated mass (bottom) spectra of carbamoylimidazolium salt **3**.

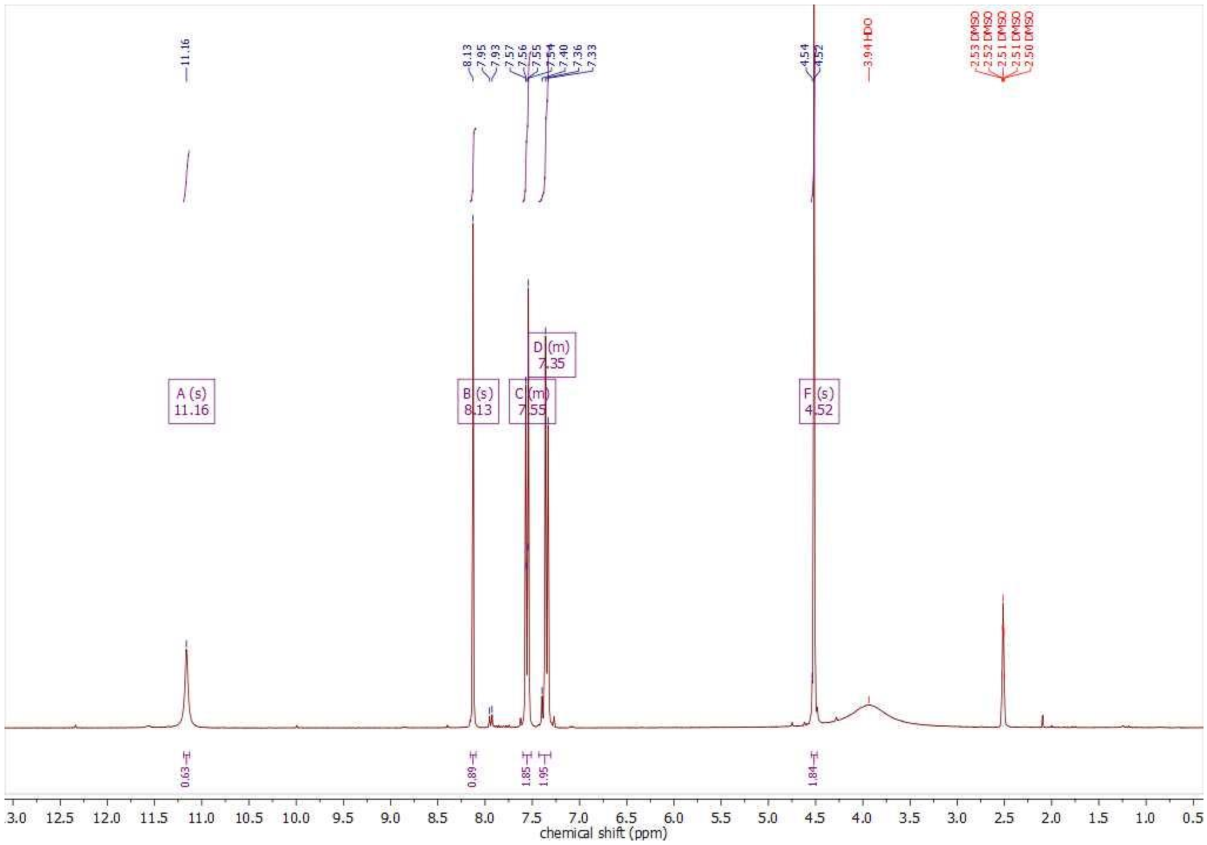


**Figure S52.** $^{1}$H-NMR spectrum of aldoxime **4**.

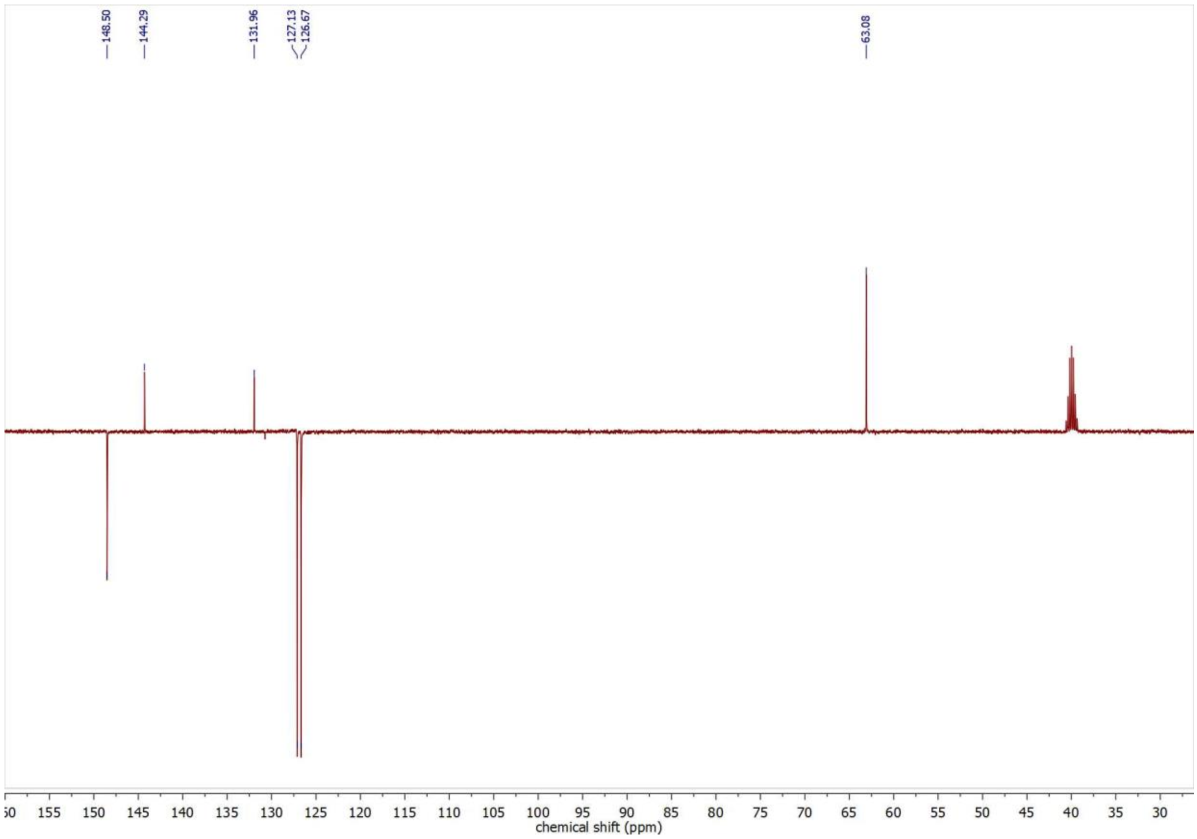


**Figure S53.** $^{13}$C-NMR spectrum of aldoxime **4**.

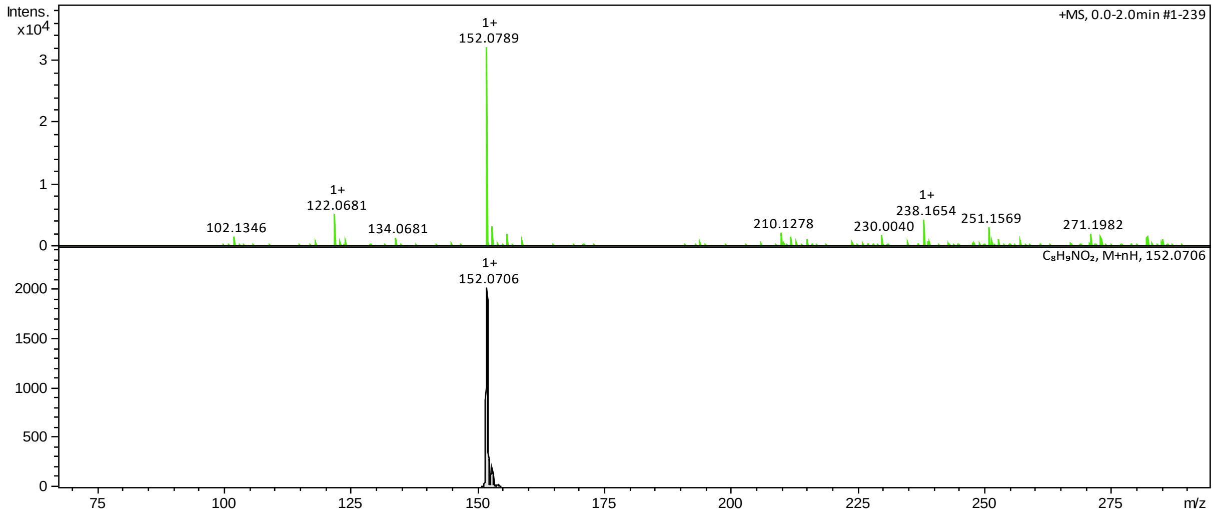


**Figure S54.** Deconvoluted ESI$^+$ HRMS (top) and calculated mass (bottom) spectra of aldoxime **4**.

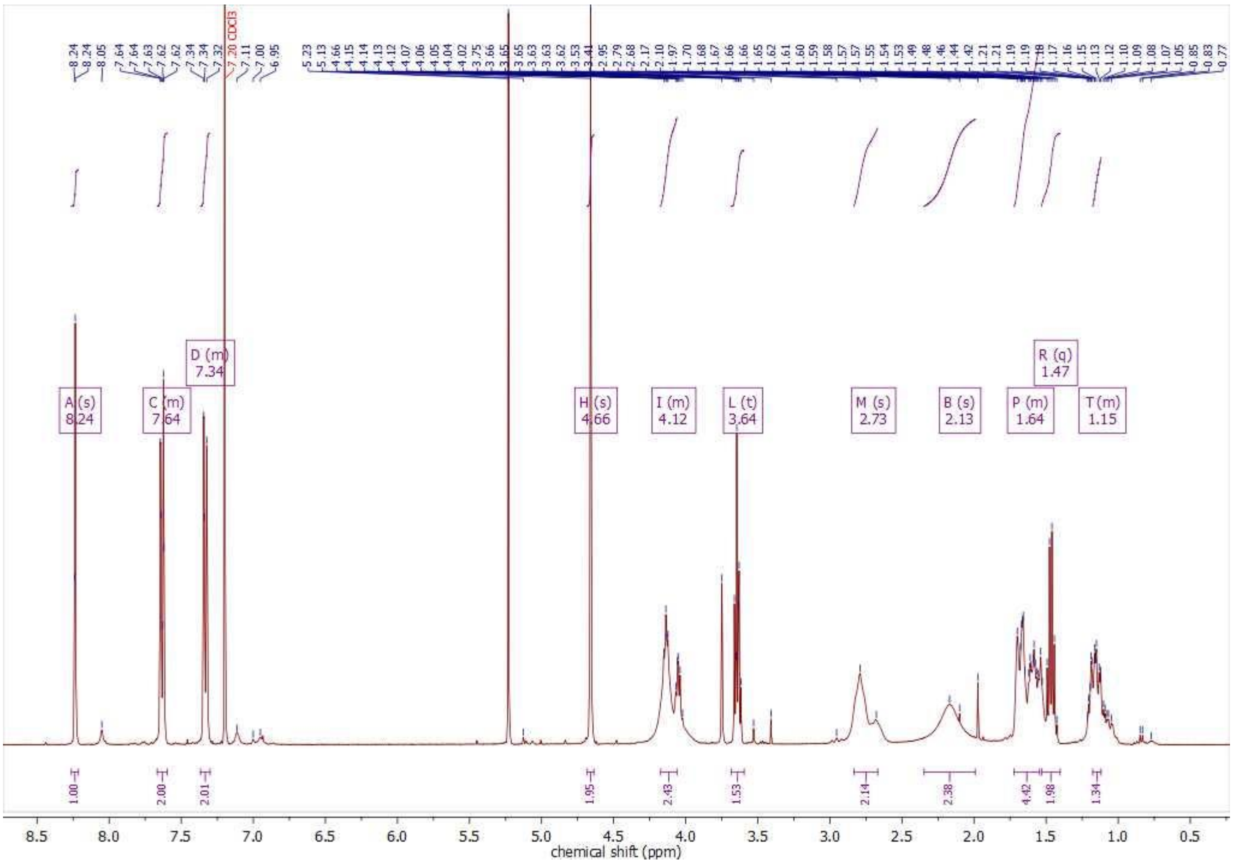


**Figure S55.** $^1$H-NMR spectrum of carbamoylaldoxime mechanophore **5**.

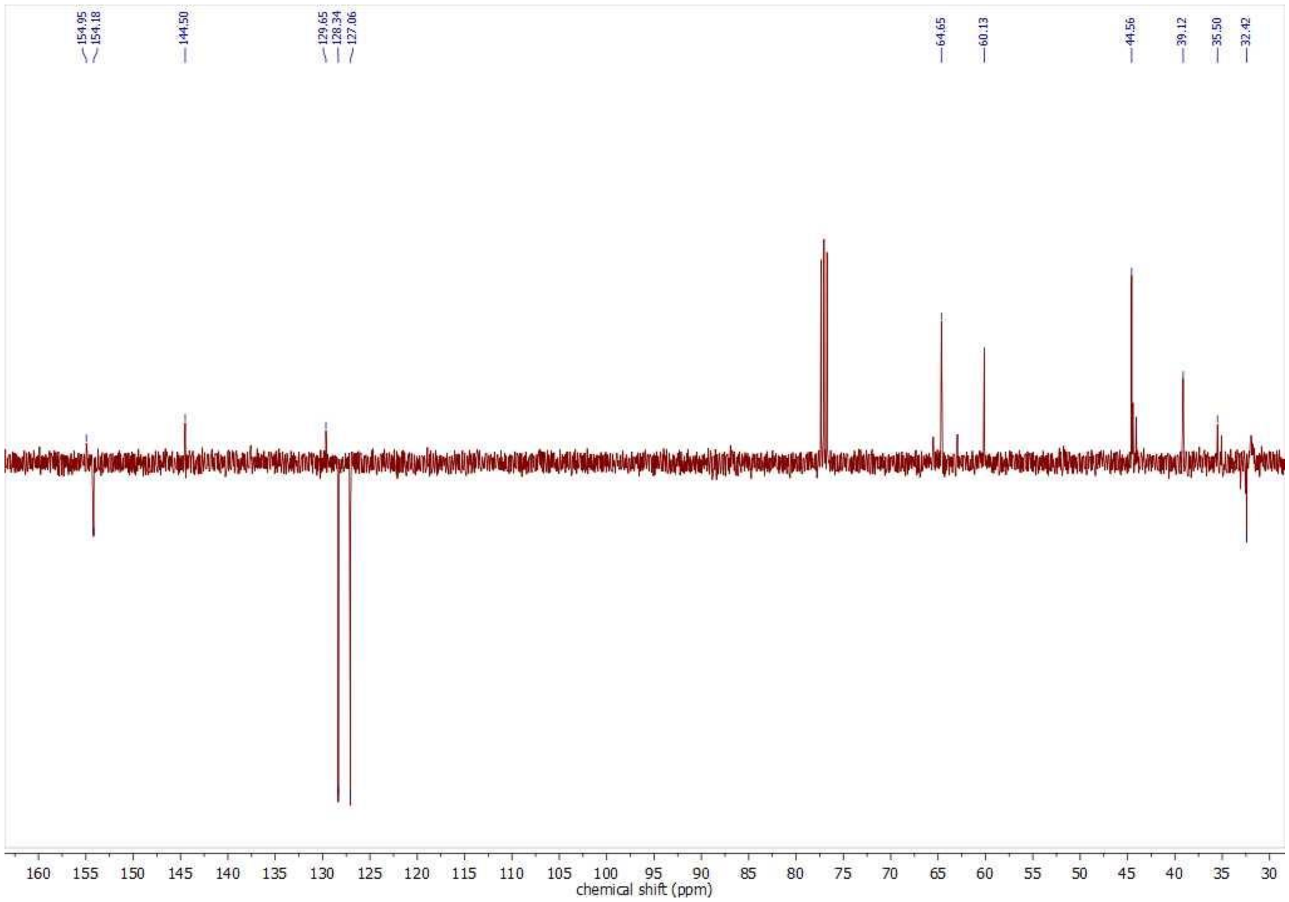


**Figure S56.** $^{13}$C-NMR spectrum of carbamoylaldoxime **5**.

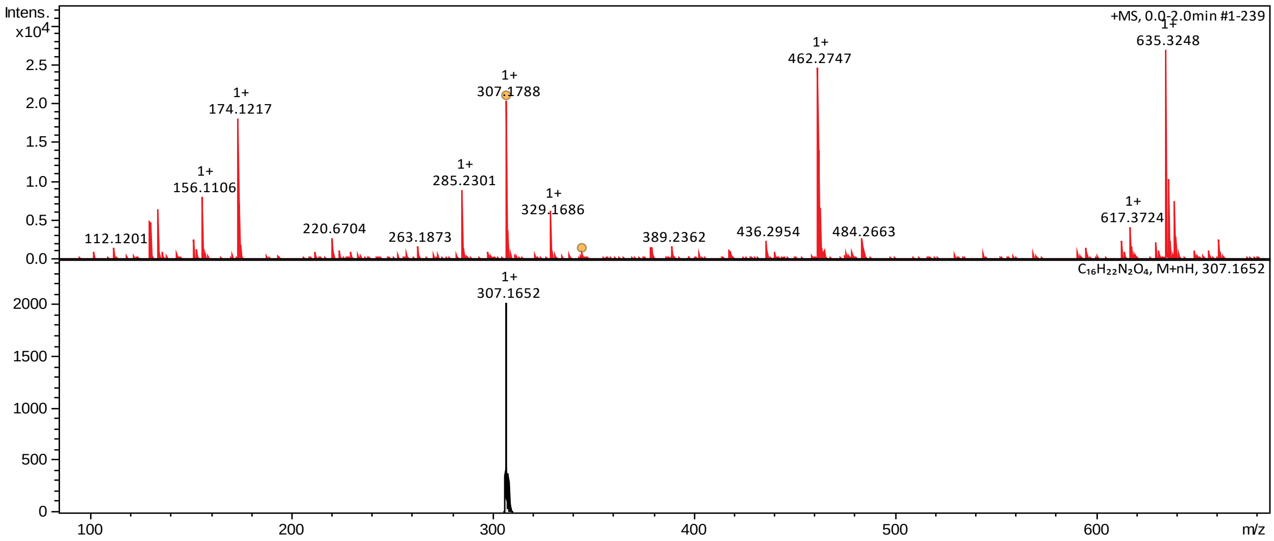


**Figure S57.** Deconvoluted ESI$^+$ HRMS (top) and calculated mass (bottom) spectra of carbamoylaldoxime mechanophore **5**.

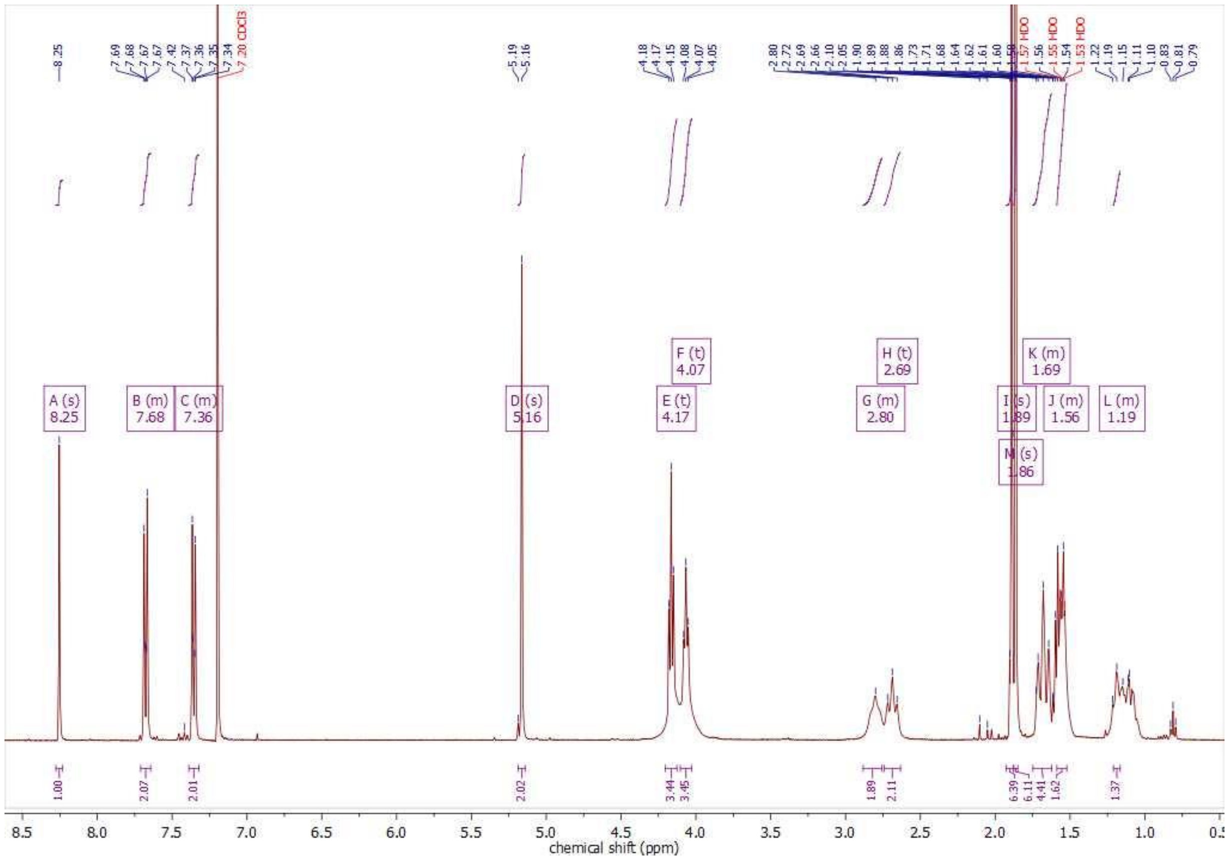


**Figure S58.** $^{1}$H-NMR spectrum of carbamoylaldoxime mechanophore initiator **6**.

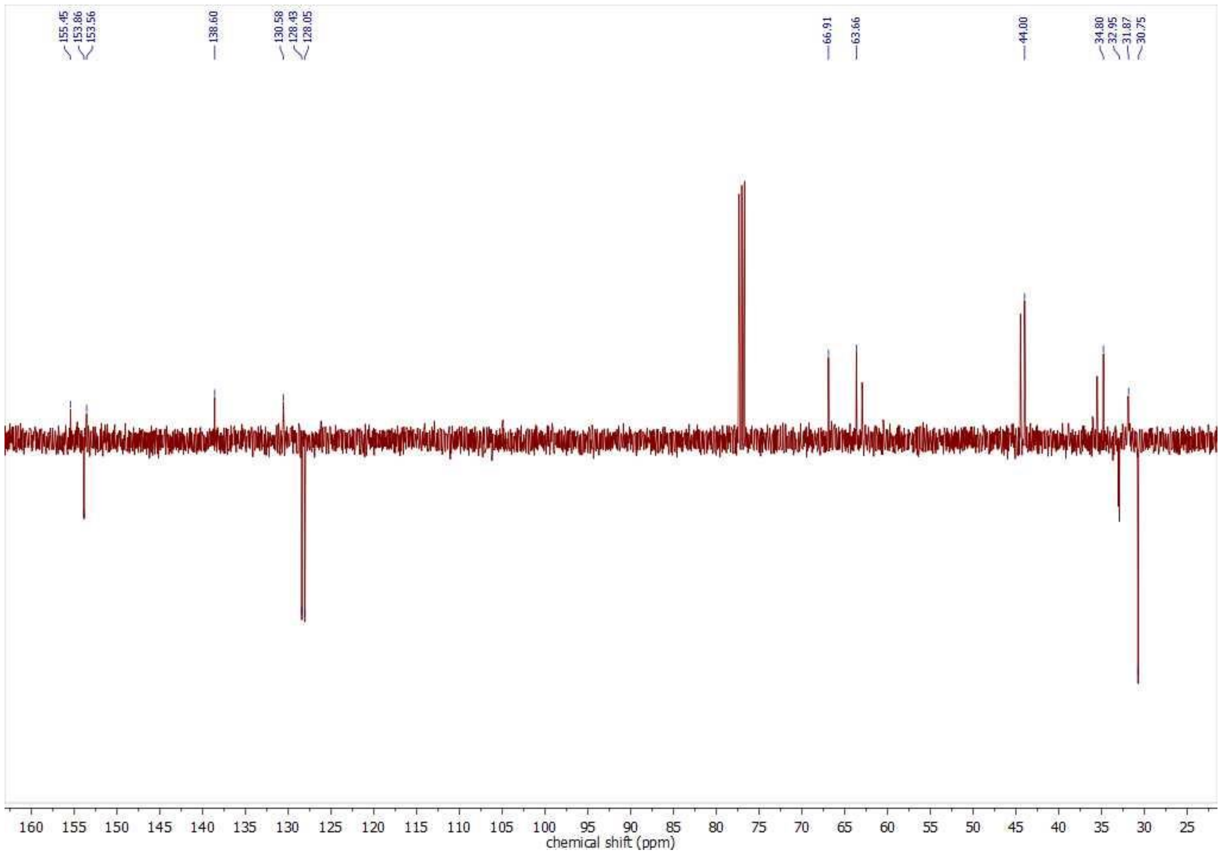


**Figure S59.** $^{13}$C-NMR spectrum of carbamoylaldoxime mechanophore initiator **6**.

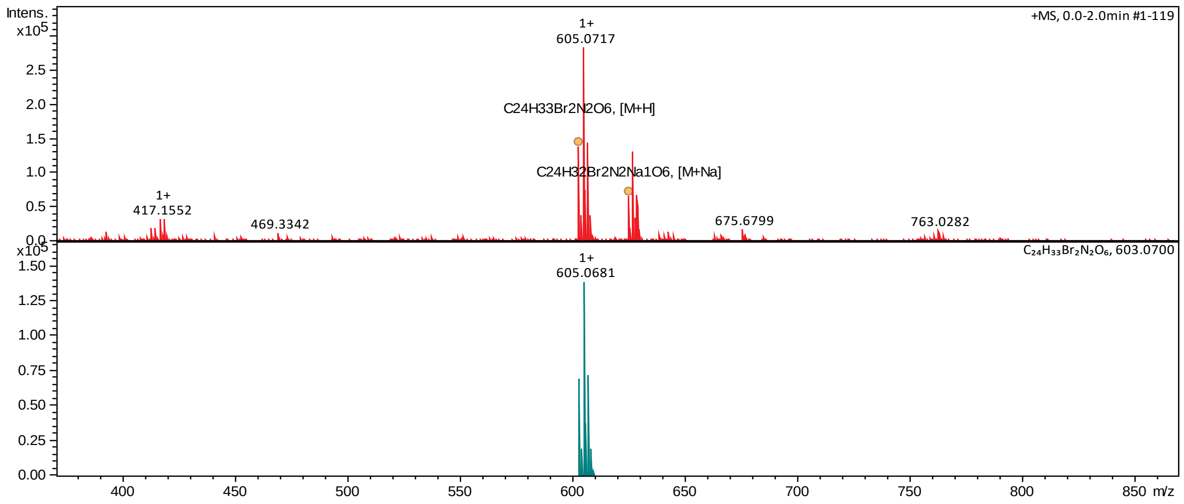


**Figure S60.** Deconvoluted ESI$^+$ HRMS (top) and calculated mass (bottom) spectra of carbamoylaldoxime mechanophore initiator **6**.

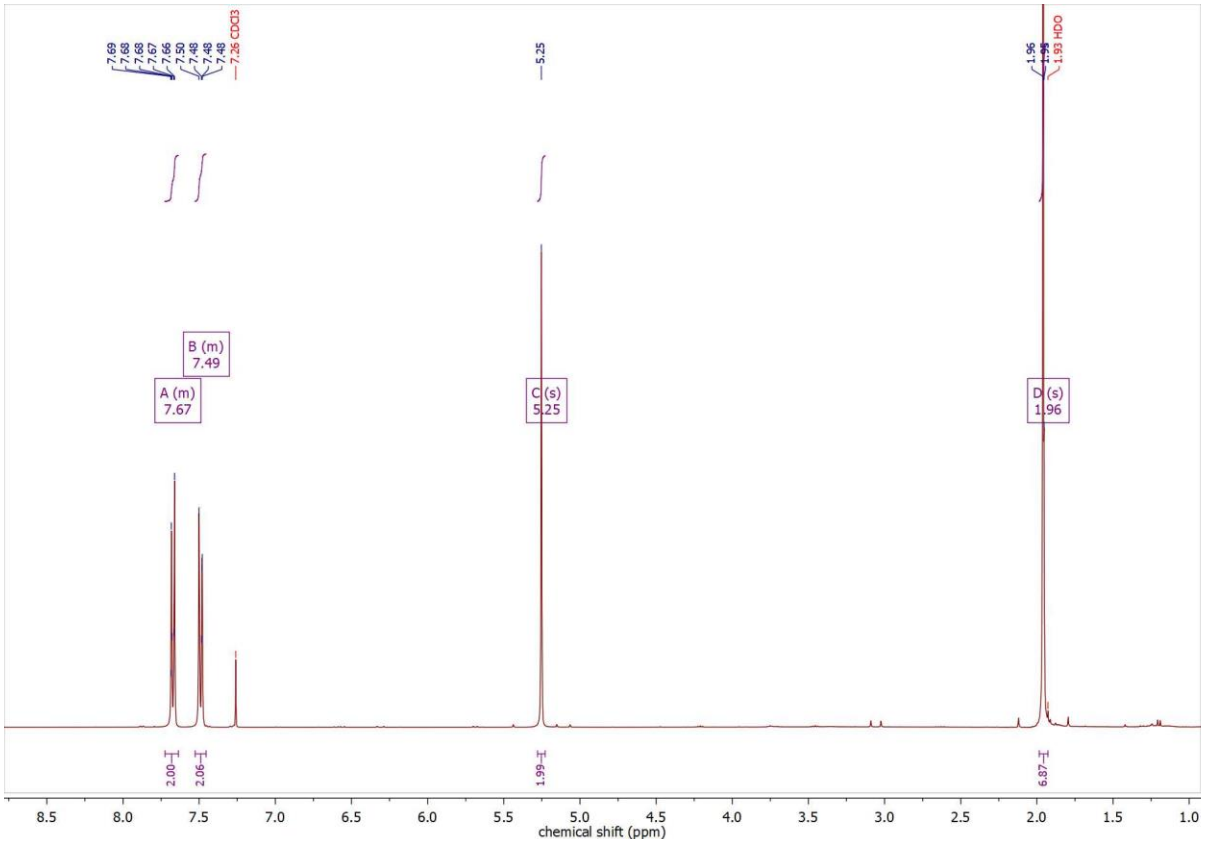


**Figure S61.** $^1$H-NMR spectrum of the nitrile reference initiator **9**.

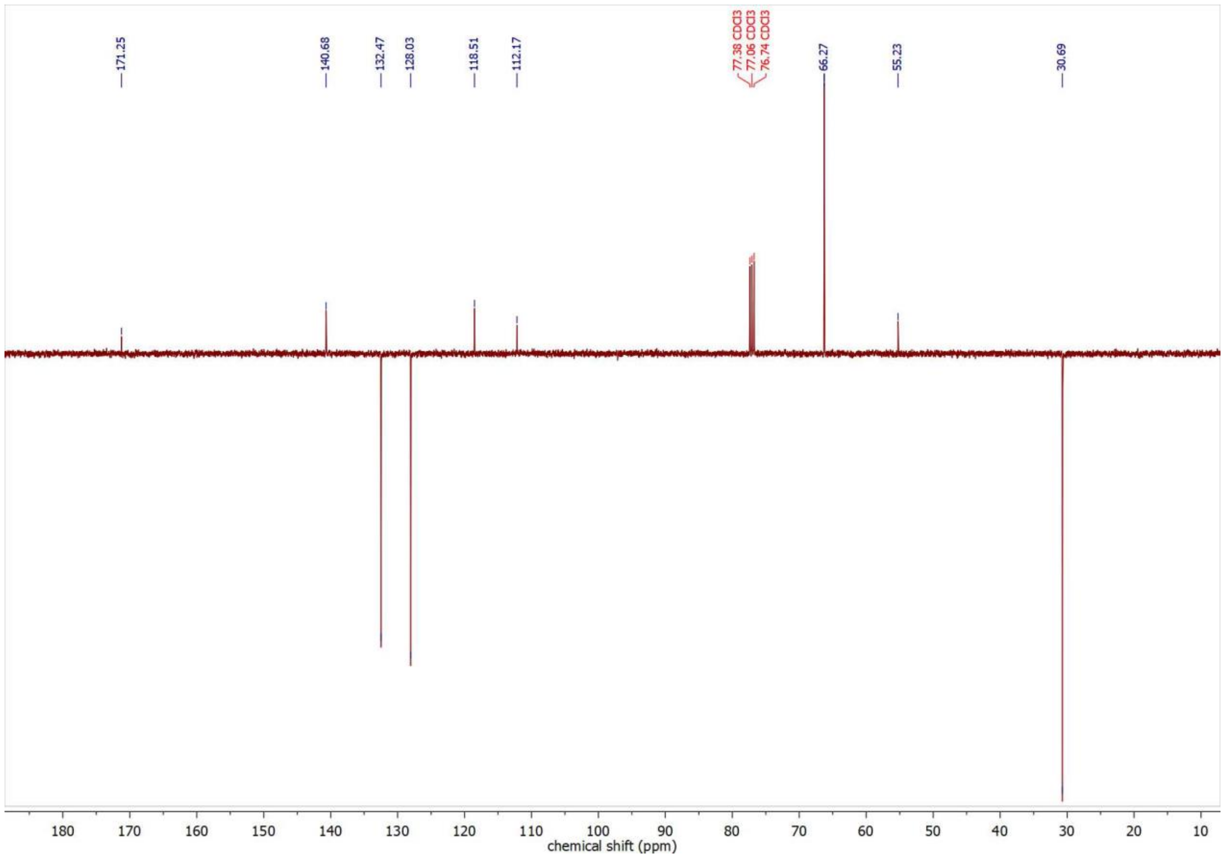


**Figure S62.** $^{13}C$-NMR spectra of the nitrile control initiator **9**.

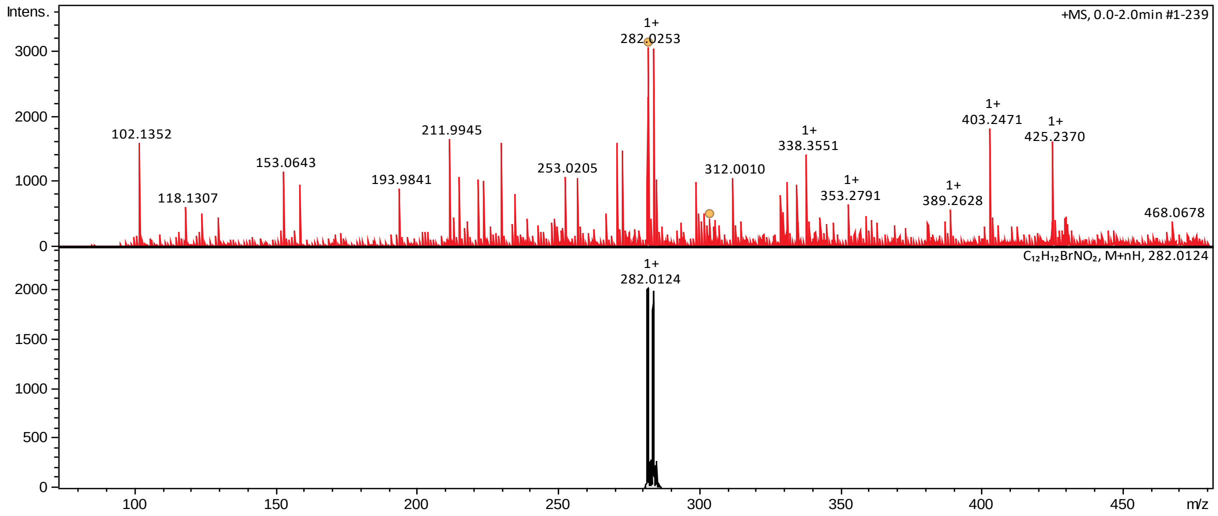


**Figure S63.** Deconvoluted ESI$^+$ HRMS (top) and calculated mass (bottom) spectra of nitrile control initiator **9**.

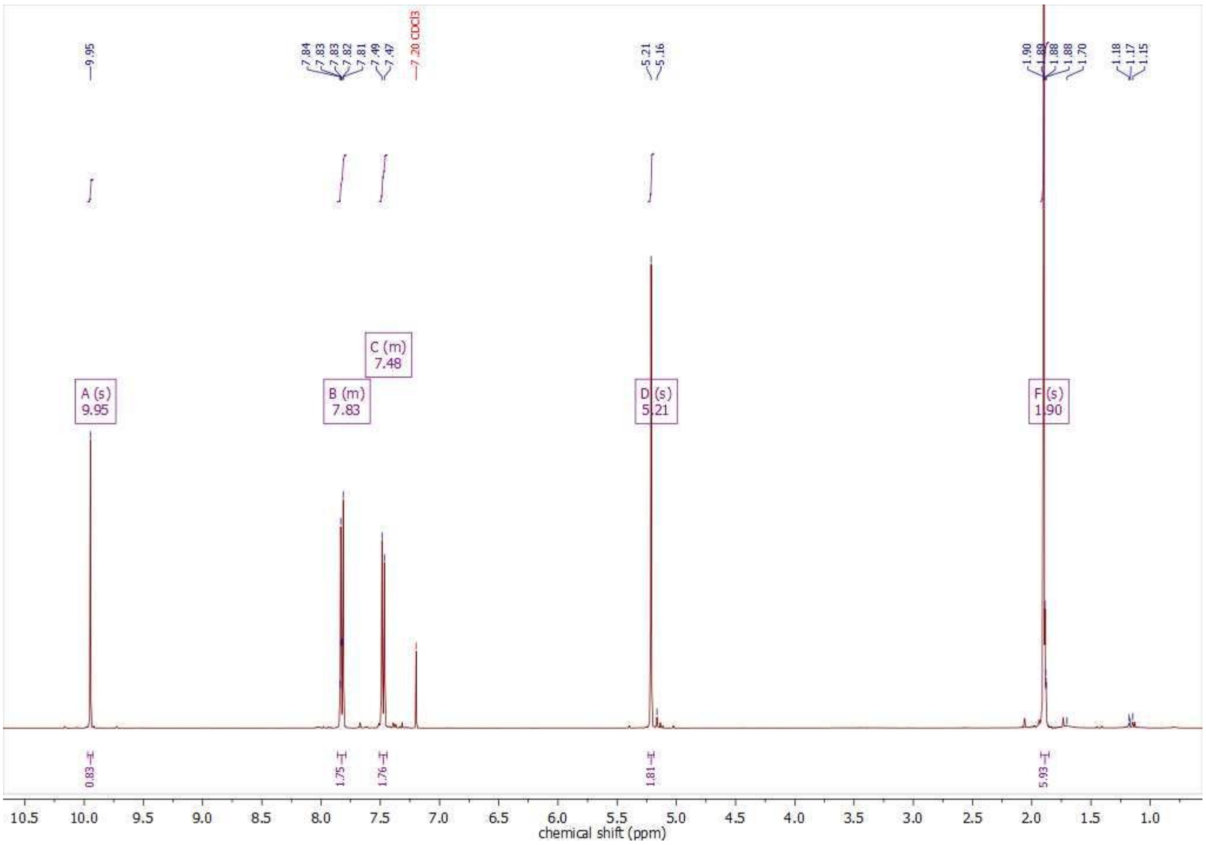


**Figure S64.** $^{1}$H-NMR spectrum of the aldehyde control initiator **10**.

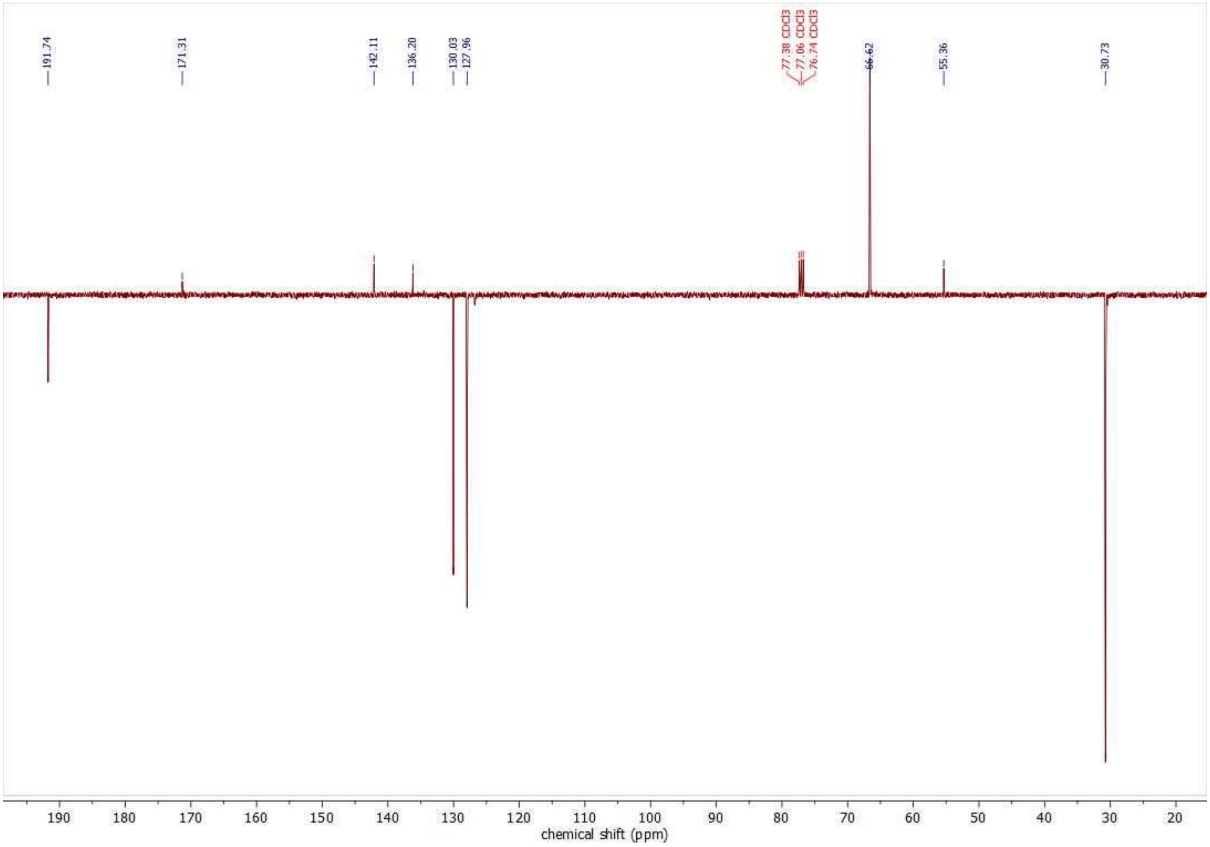


**Figure S65.** $^{13}$C-NMR spectrum of the aldehyde control initiator **10**.

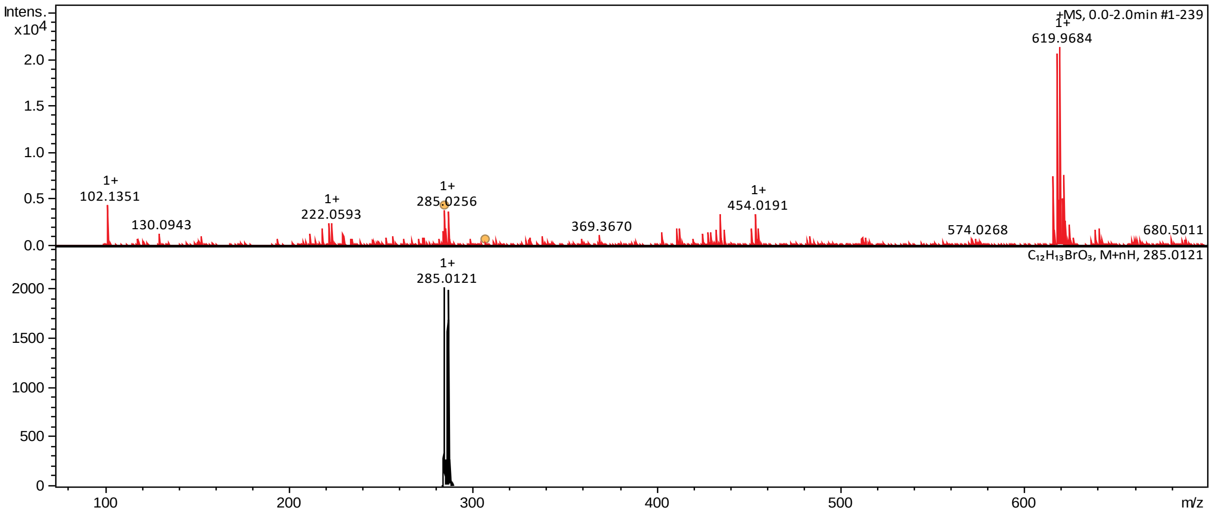


**Figure S66.** Deconvoluted ESI$^+$ HRMS (top) and calculated mass (bottom) spectra of aldehyde control initiator **10**.

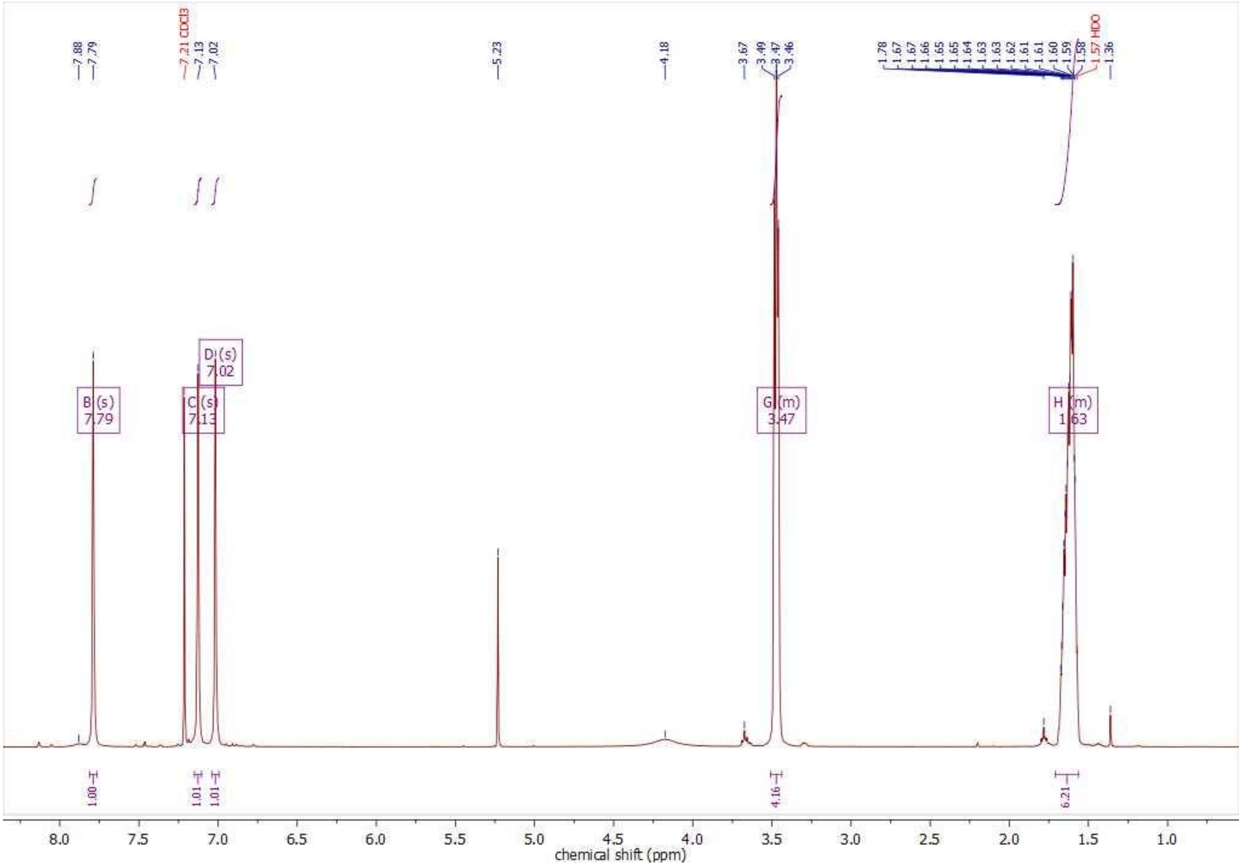


**Figure S67.** $^1$H-NMR spectrum of carbamoylimidazole **11**.

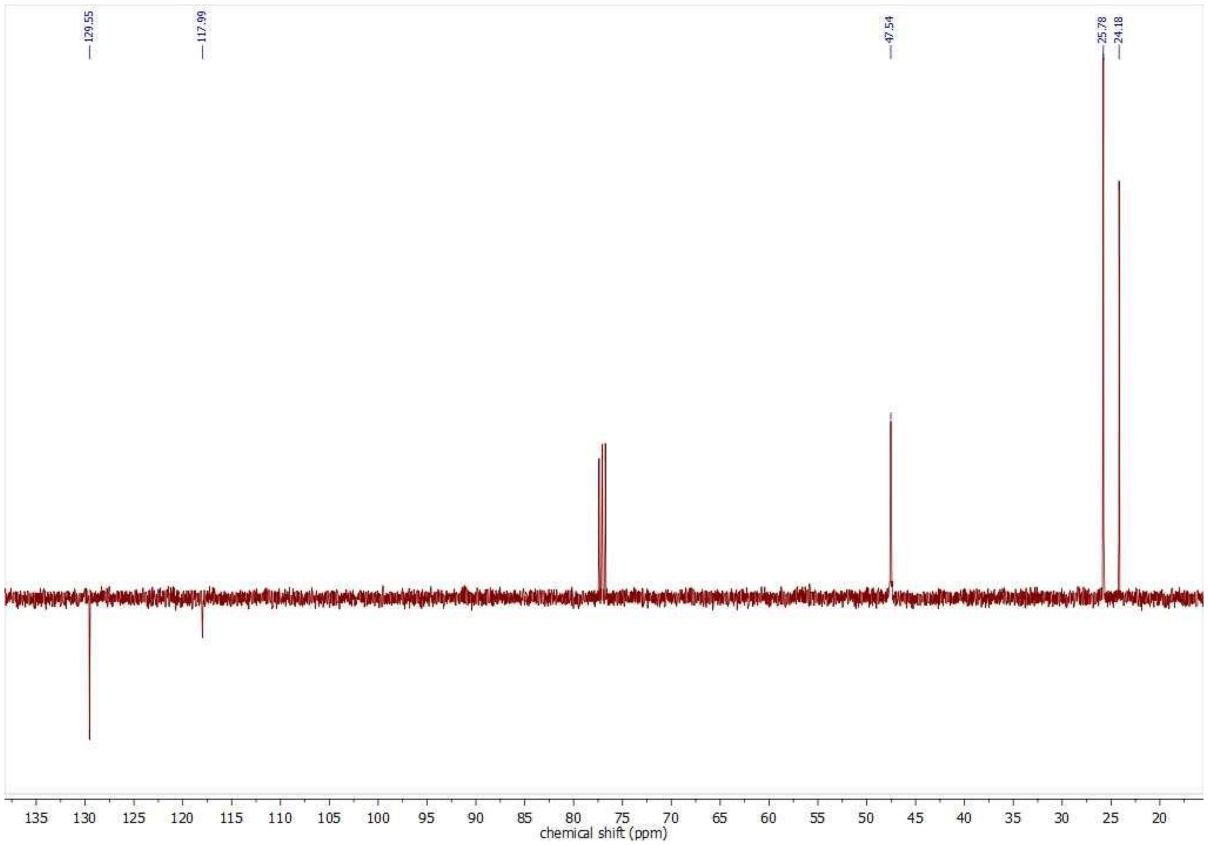


**Figure S68.** $^{13}C$-NMR spectrum of carbamoylimidazole **11**.

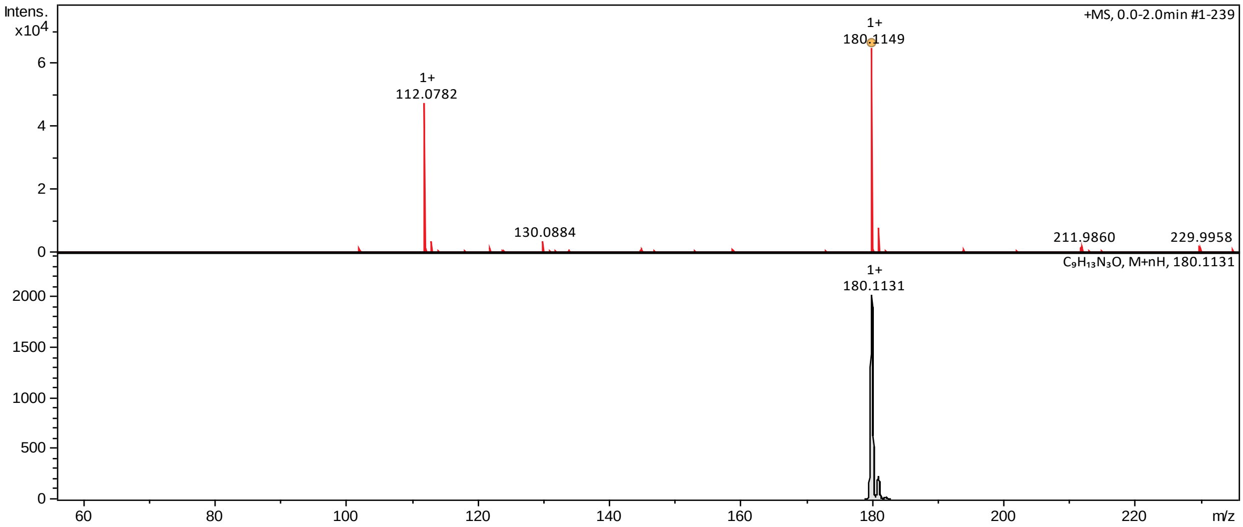


**Figure S69.** Deconvoluted ESI$^{+}$ HRMS (top) and calculated mass (bottom) spectra of carbamoylimidazole **11**.

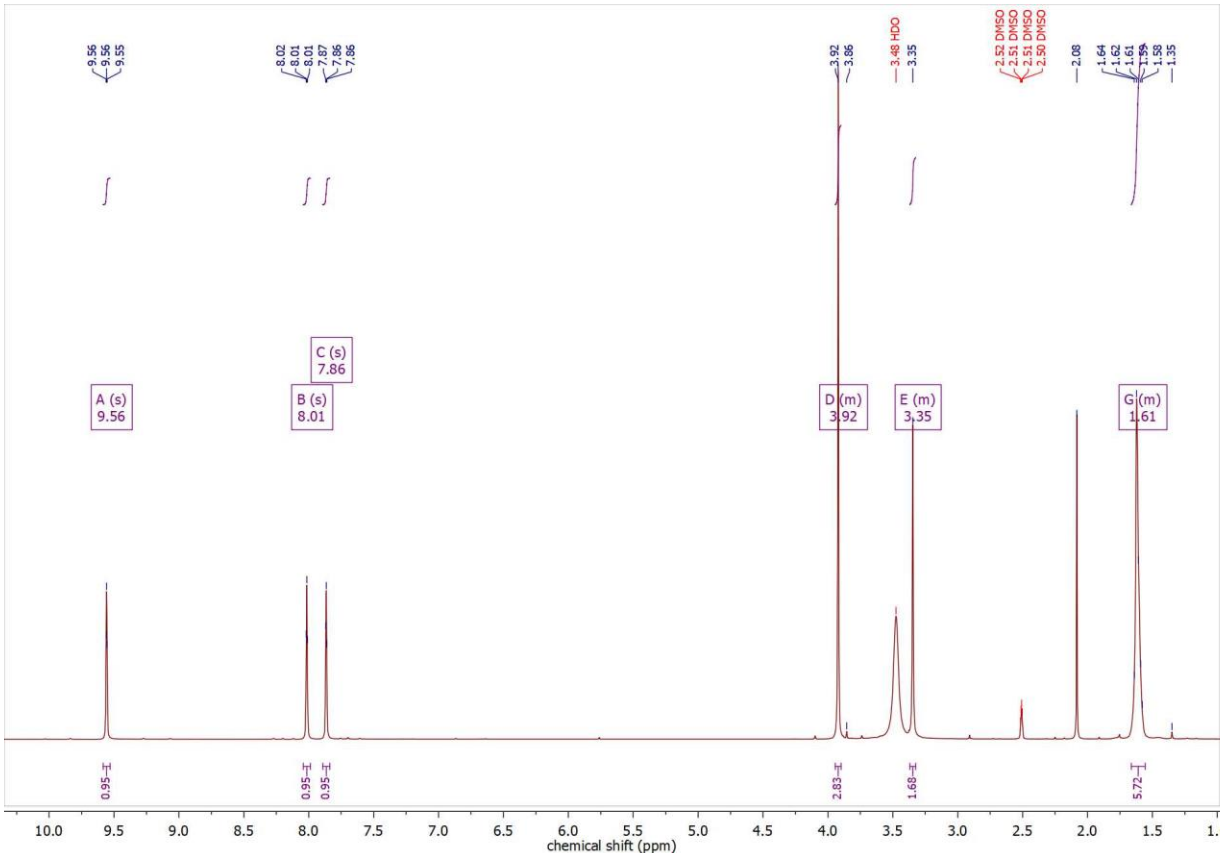


**Figure S70.** $^{1}$H-NMR spectrum of the carbamoylimidazolium salt **12**.

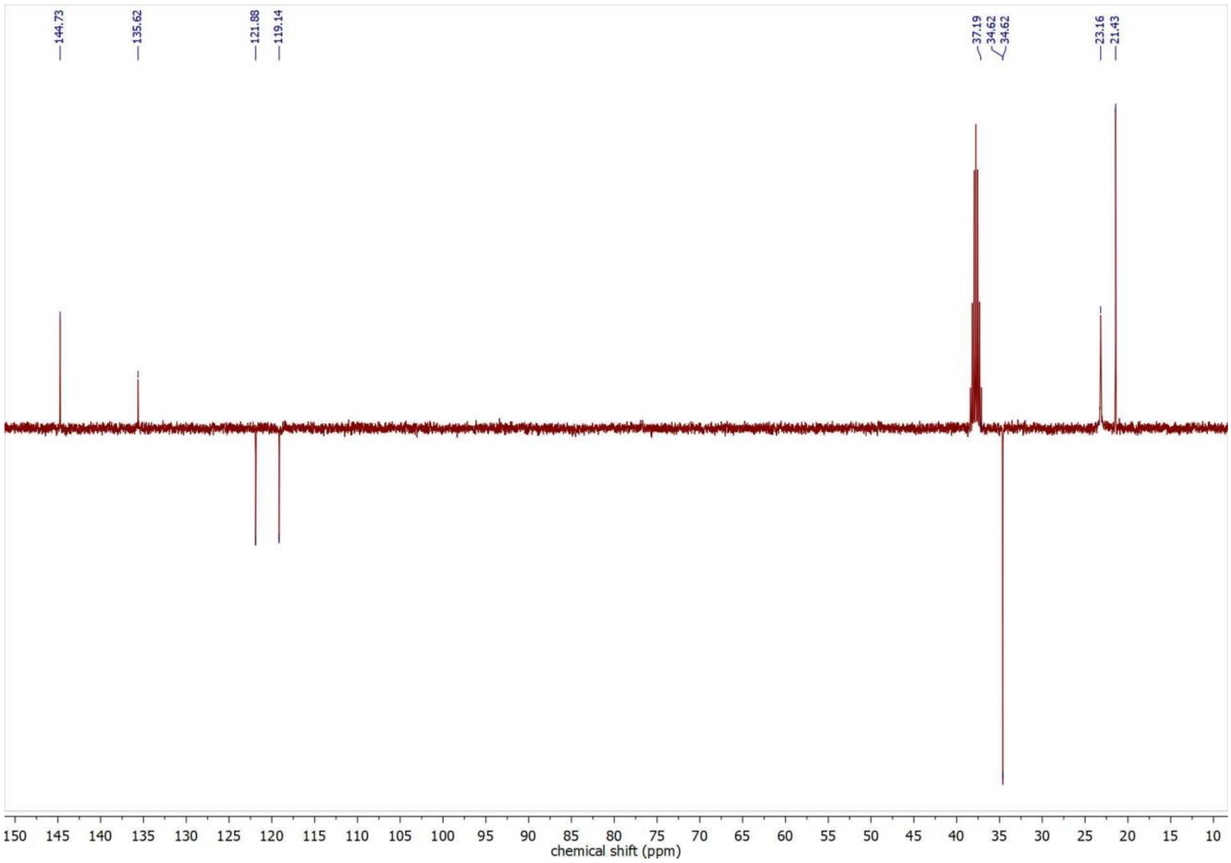


**Figure S71.** $^{13}$C-NMR spectrum of the carbmoyilimidazolium salt **12**.

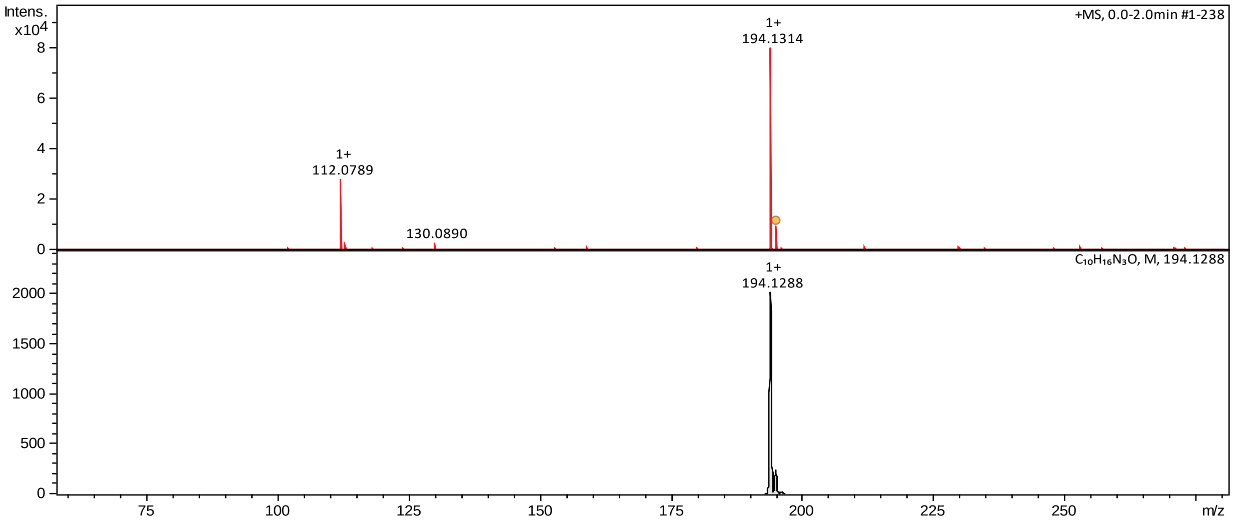


**Figure S72.** Deconvoluted ESI$^+$ HRMS (top) and calculated mass (bottom) spectra of carbamoylimidazolium **12**.

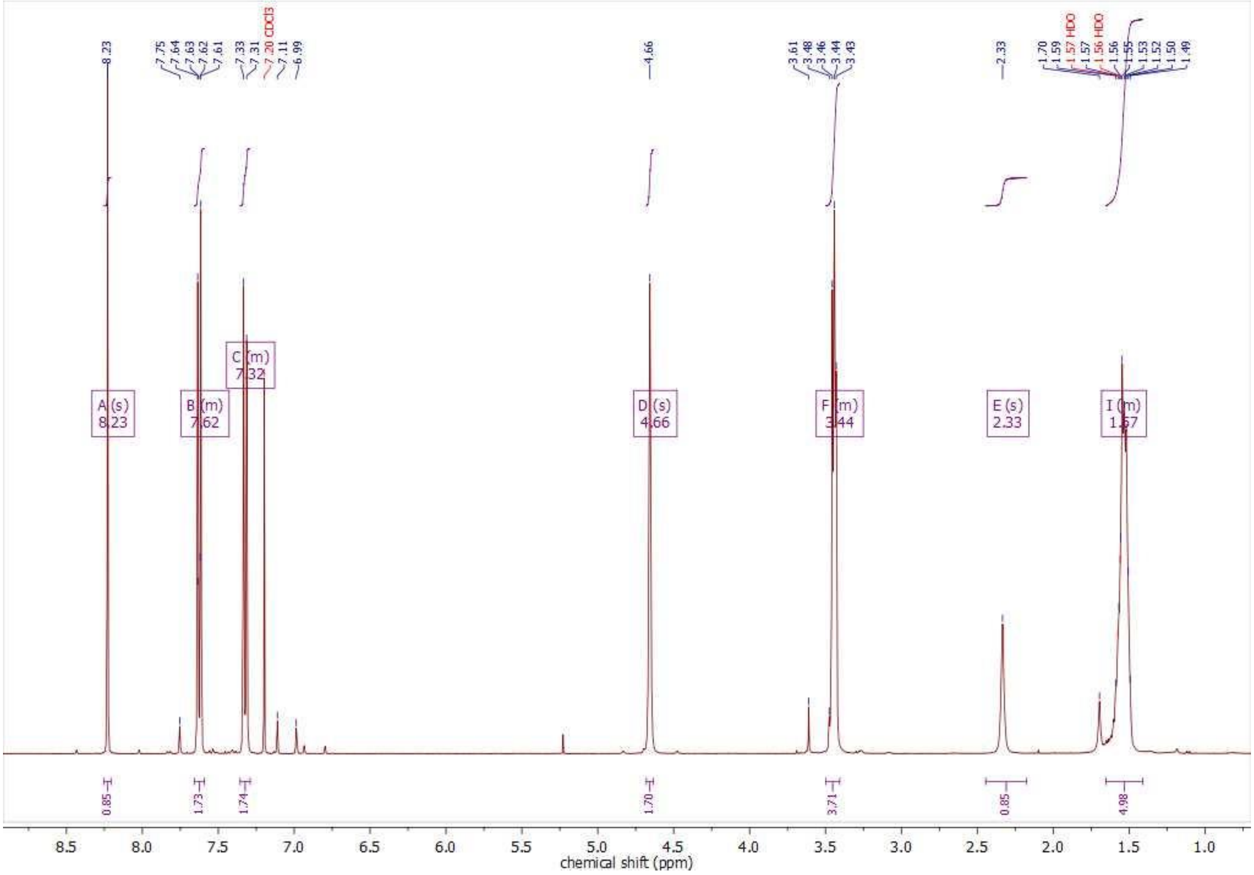


**Figure S73.** $^{1}$H-NMR spectrum of carbamoylaldoxime control **13**.

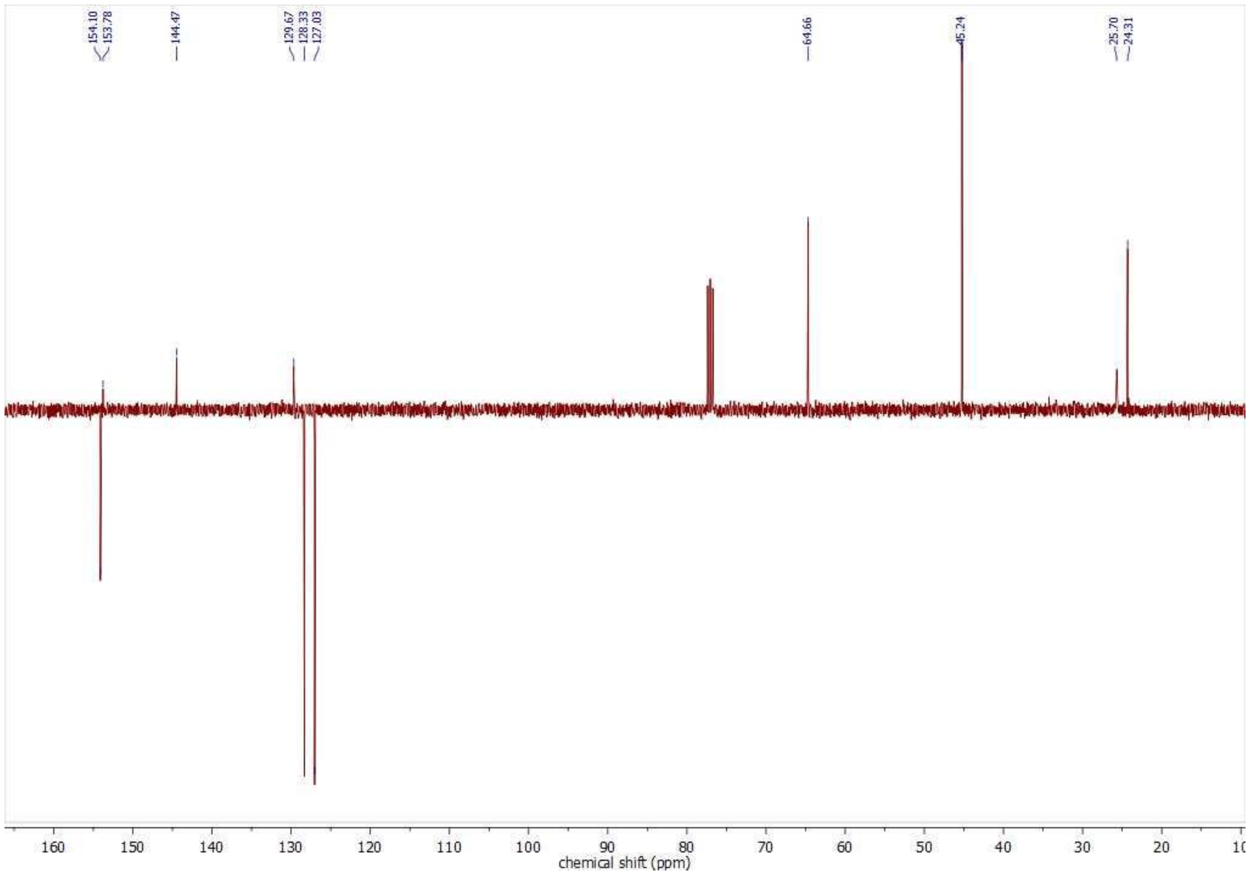


**Figure S74.** $^{13}$C-NMR spectrum of carbamoylaldoxime control **13**.

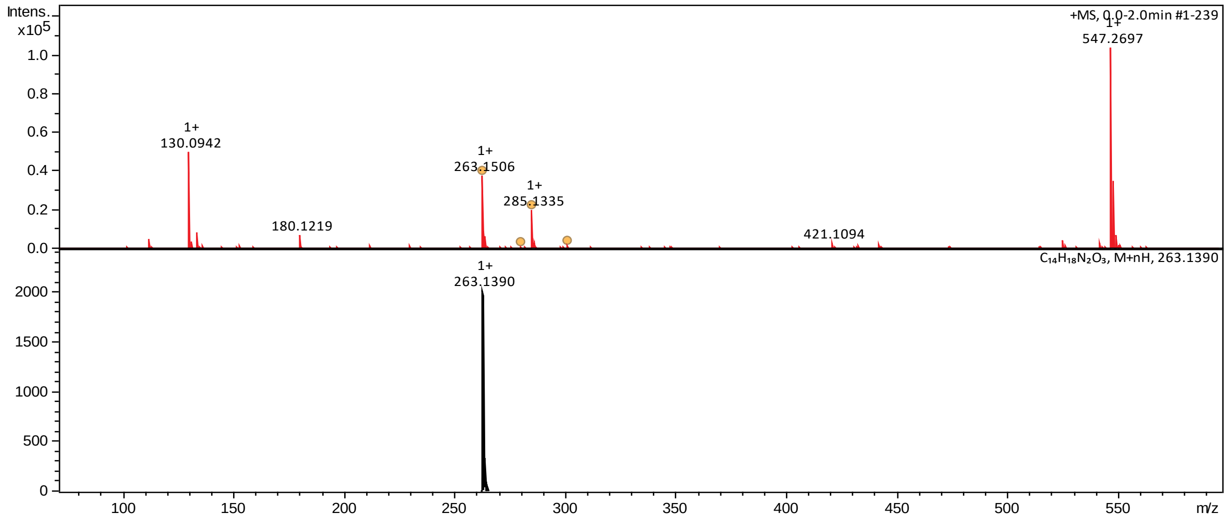


**Figure S75.** Deconvoluted ESI$^+$ HRMS (top) and calculated mass (bottom) spectra of carbamoylaldoxime control **13**.

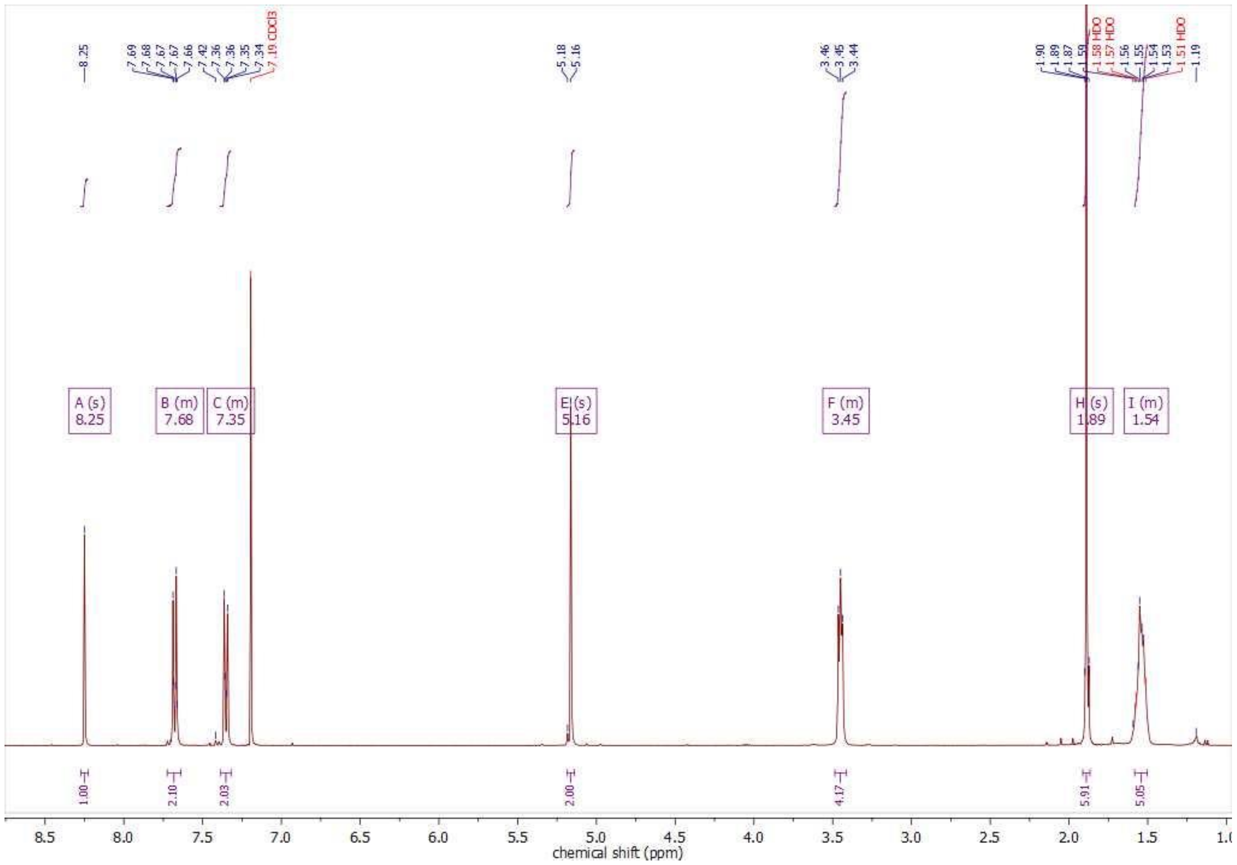

**Figure S76**. $^{1}$H-NMR spectrum of carbamoylaldoxime control initiator **14**.

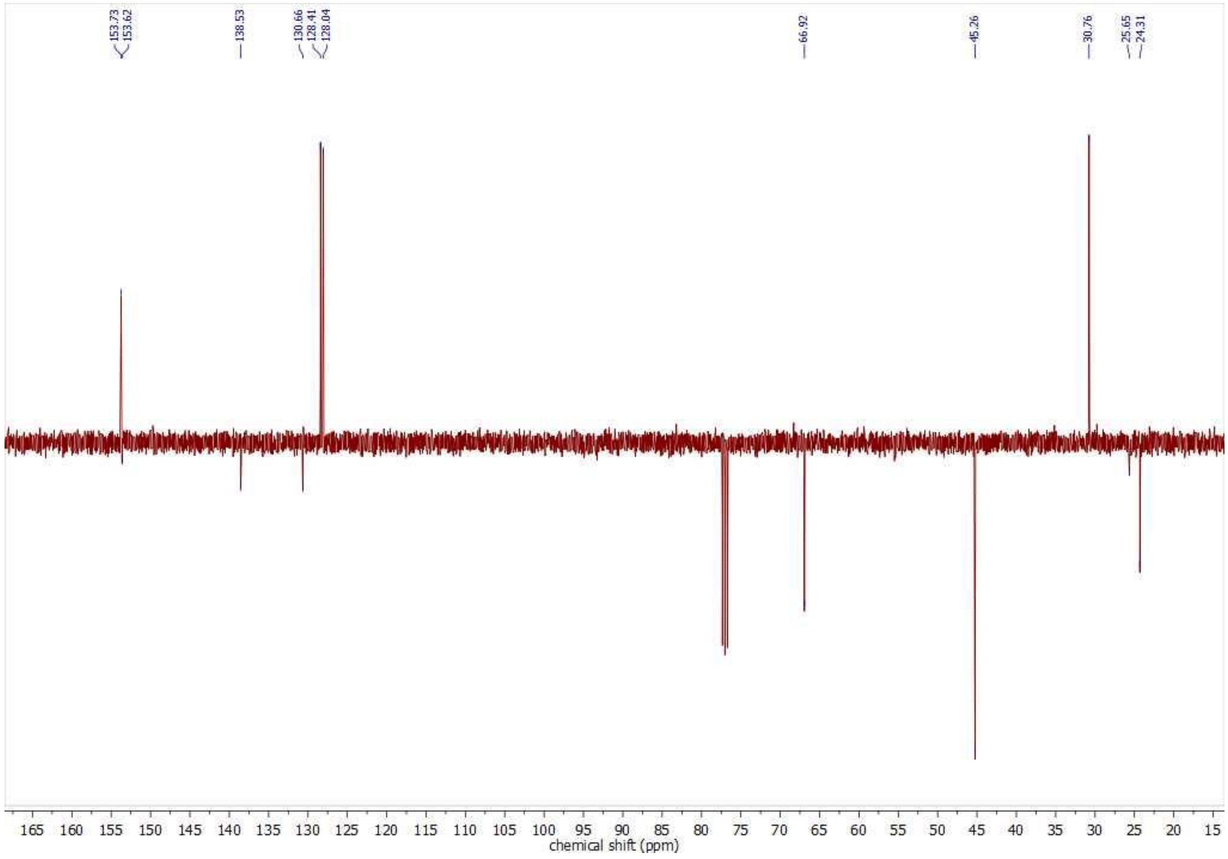

**Figure S77.** $^{13}$C-NMR spectrum of carbamoylaldoxime control initiator **14**.

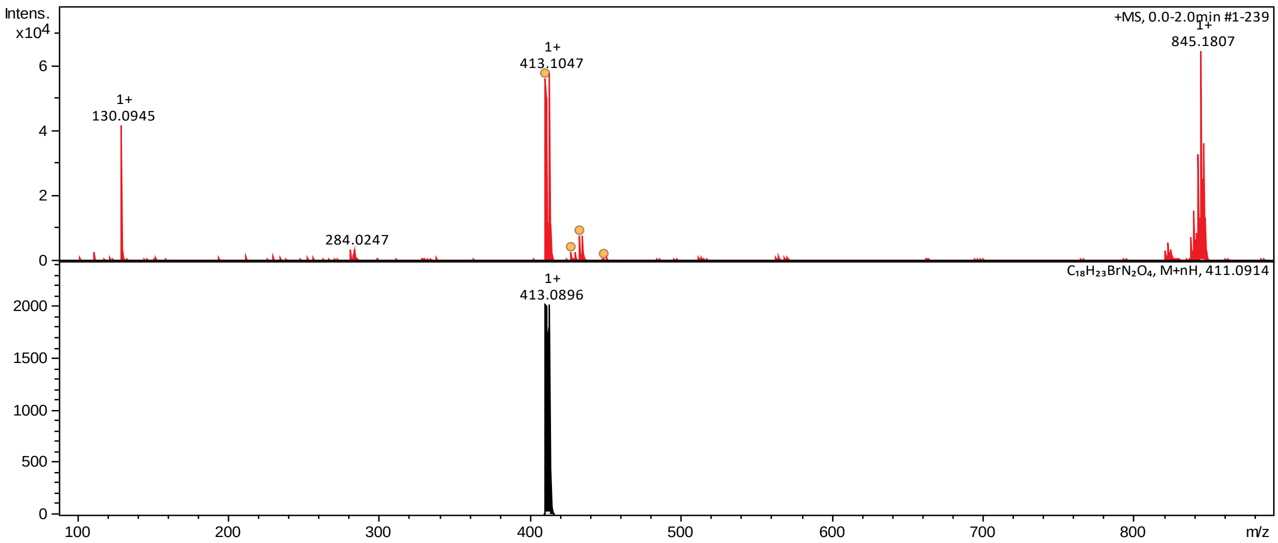


**Figure S78**. Deconvoluted ESI$^+$ HRMS (top) and calculated mass (bottom) spectra of carbamoylaldoxime control initiator **14**.

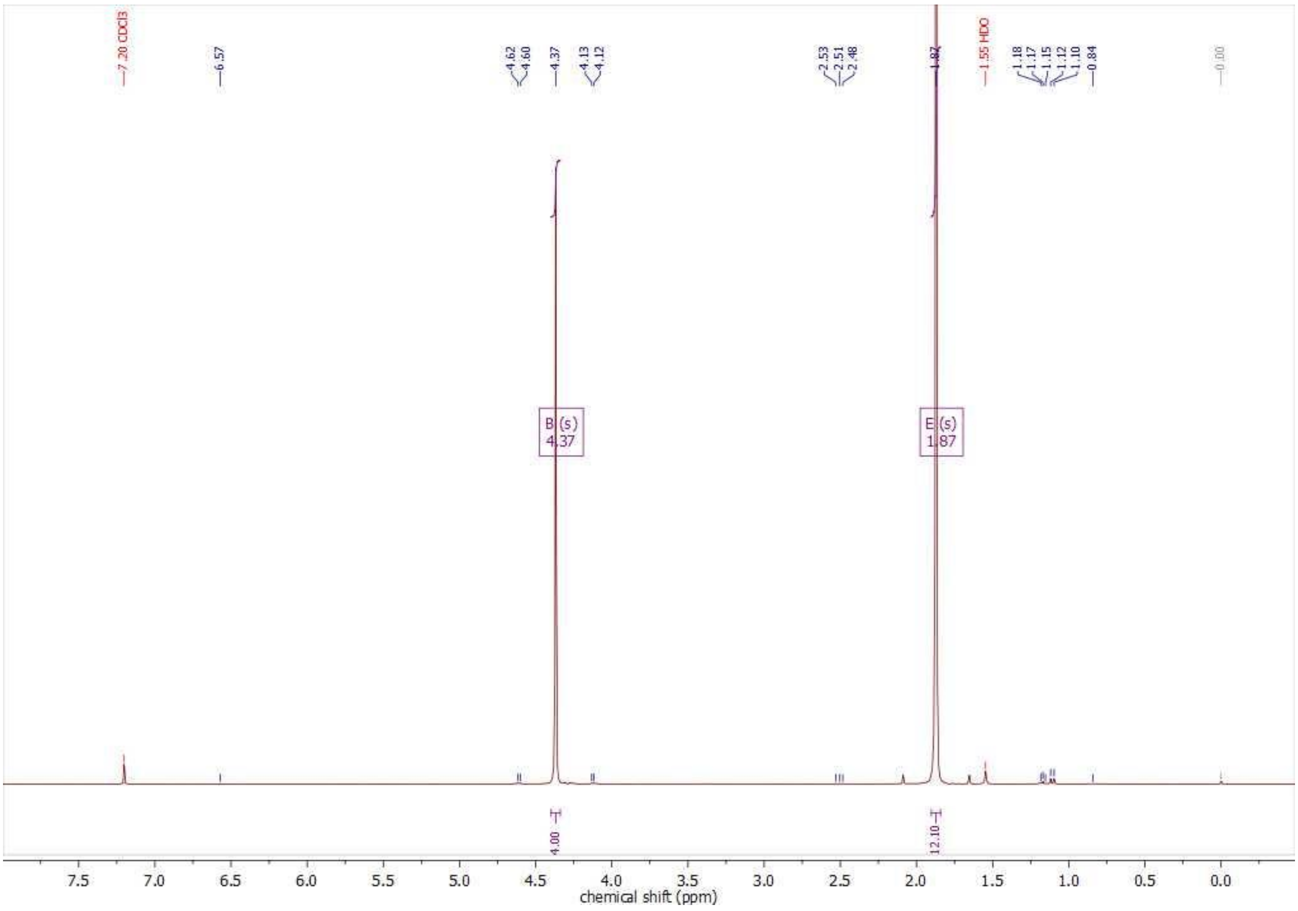


**Figure S79.** $^1$H-NMR spectrum of control initiator **15**.

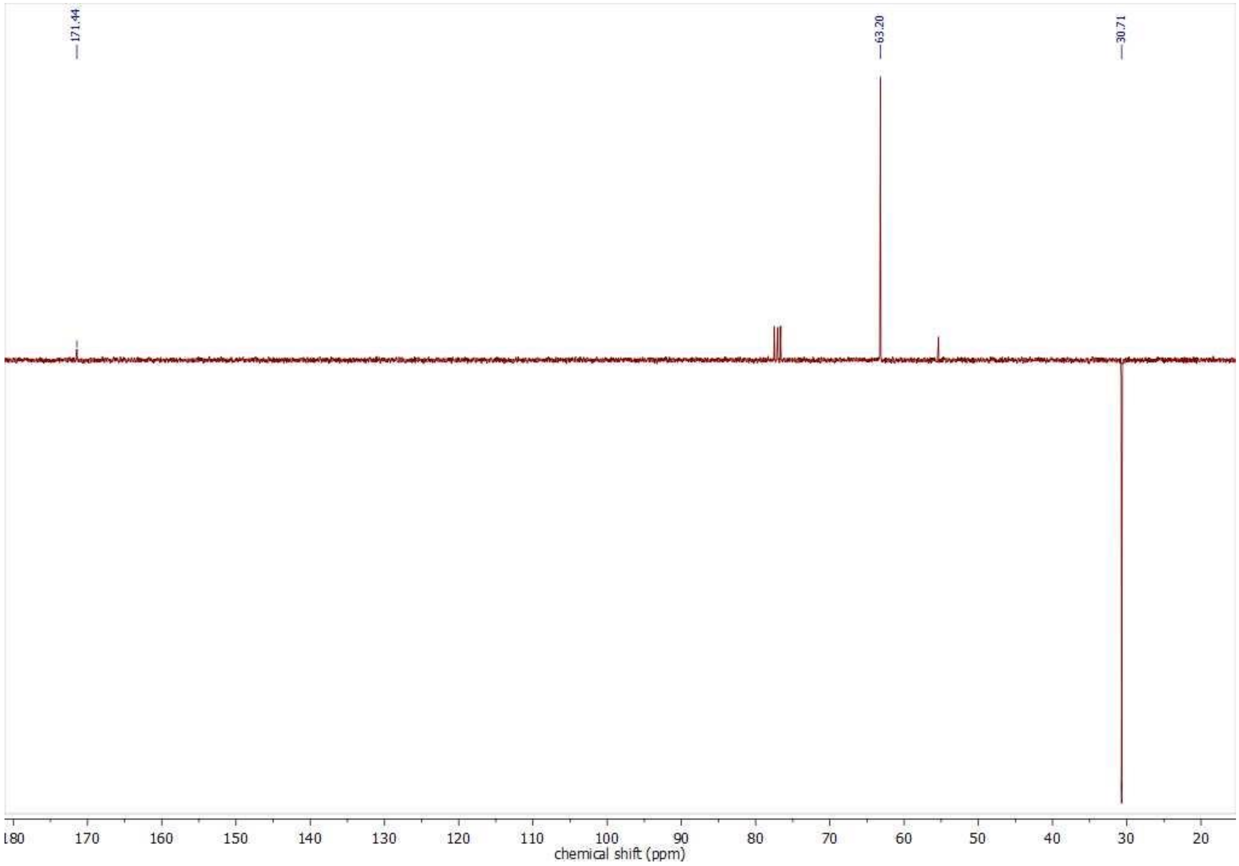


**Figure S80**. $^{13}$C-NMR spectrum of control initiator **15**.

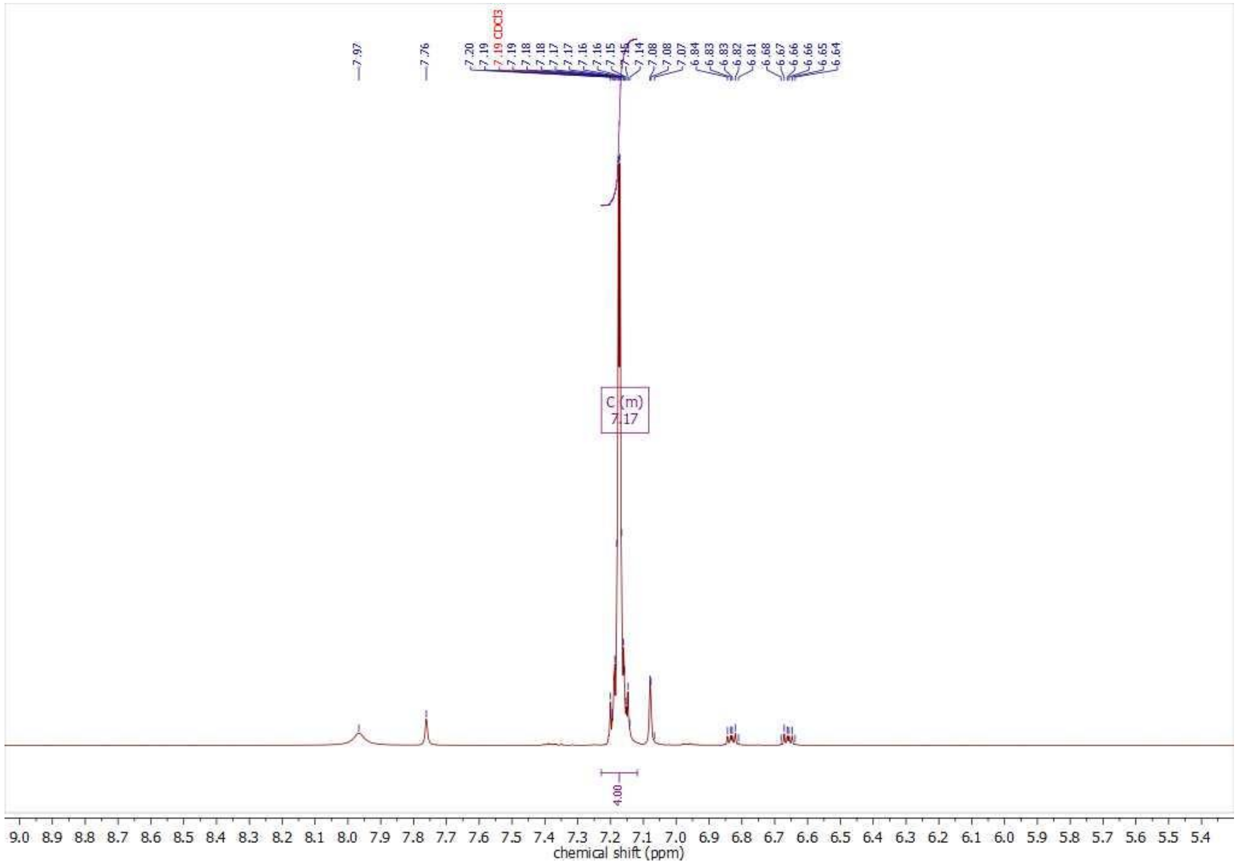


**Figure S81**. $^{1}$H-NMR spectrum of carbonate **16**.

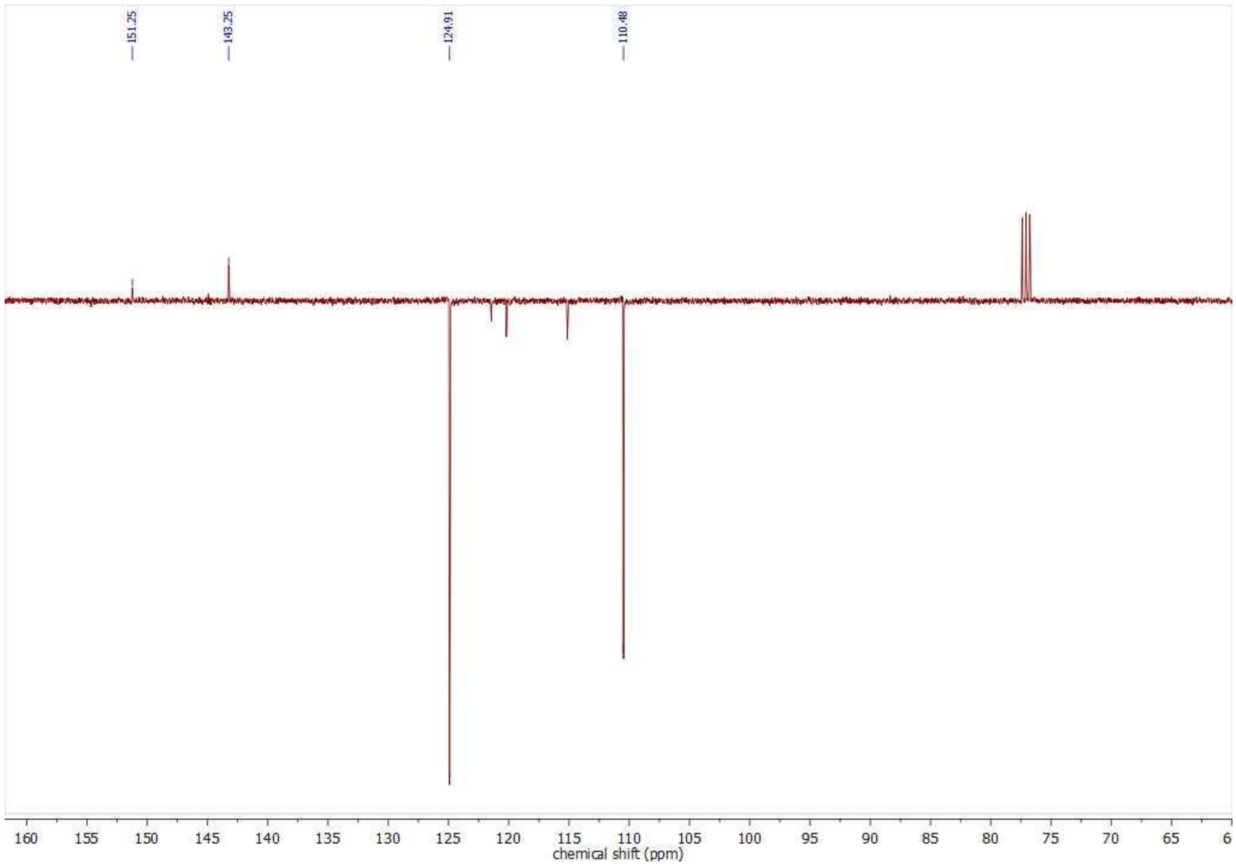

**Figure S82**. $^{13}$C-NMR spectrum of carbonate **16**.

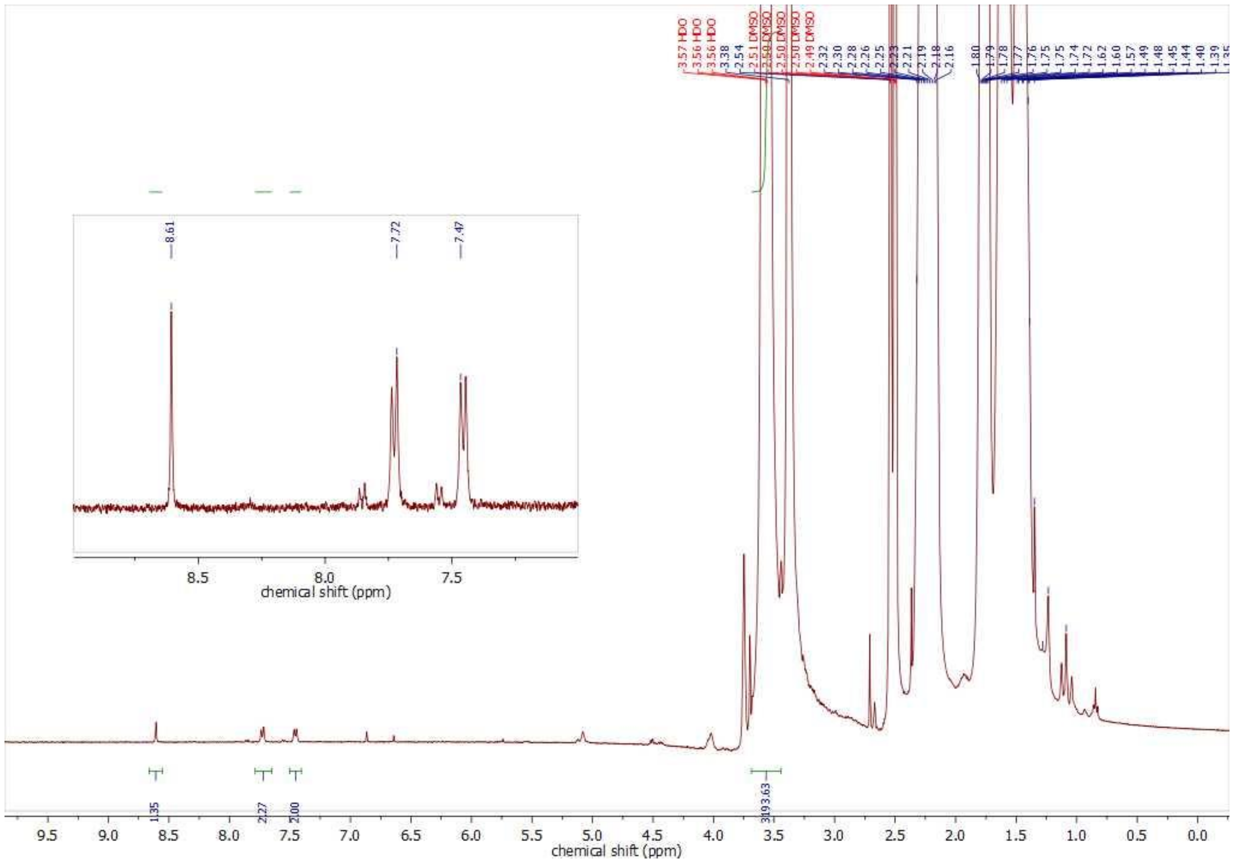

**Figure S83**. $^{1}$H-NMR spectrum of **PMA6$_{107}$**.

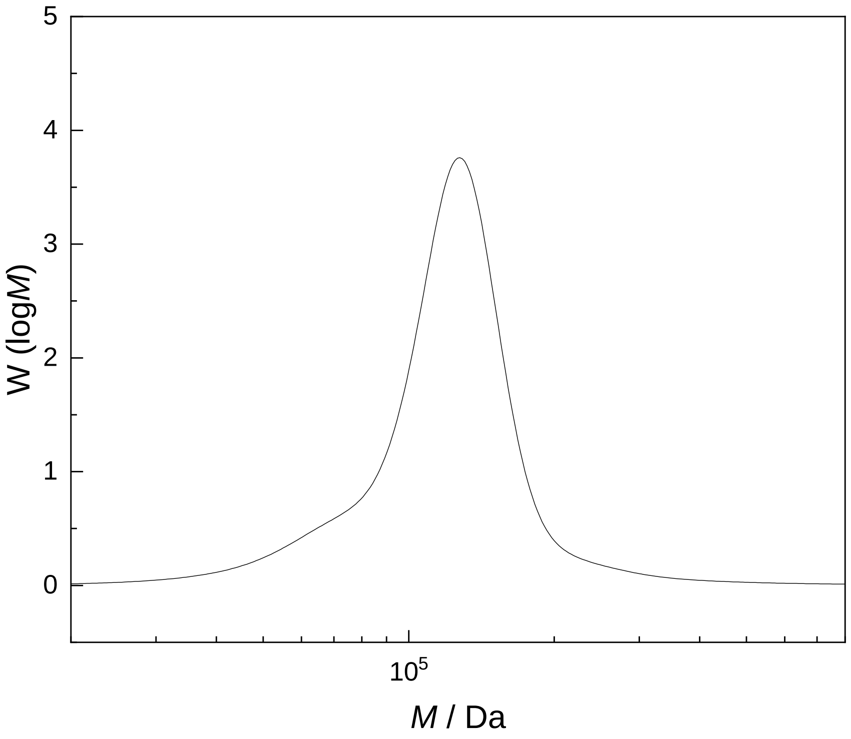


**Figure S84**. Molar mass distribution of GPC RI chromatogram (eluent: THF) of **PMA6$_{107}$**.

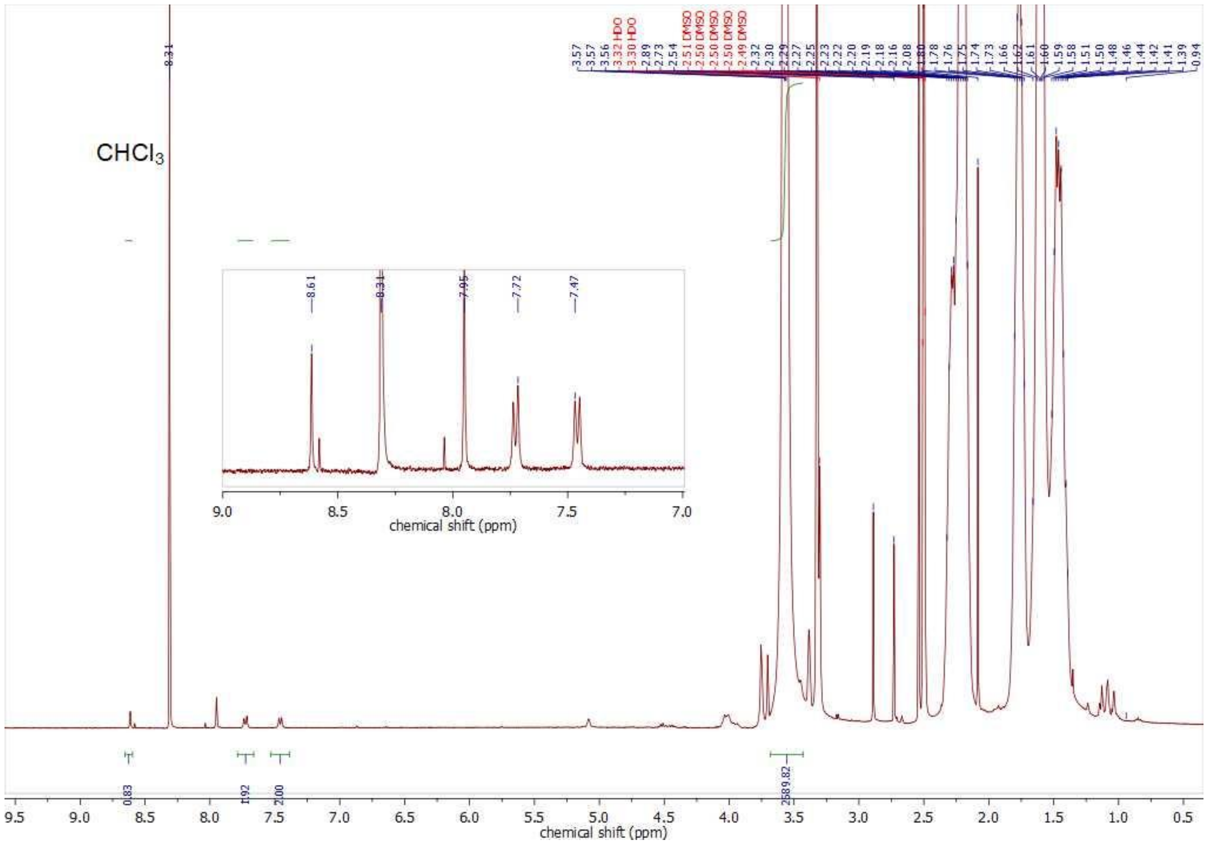


**Figure S85.** $^{1}$H-NMR spectrum of **PMA6$_{80}$**.

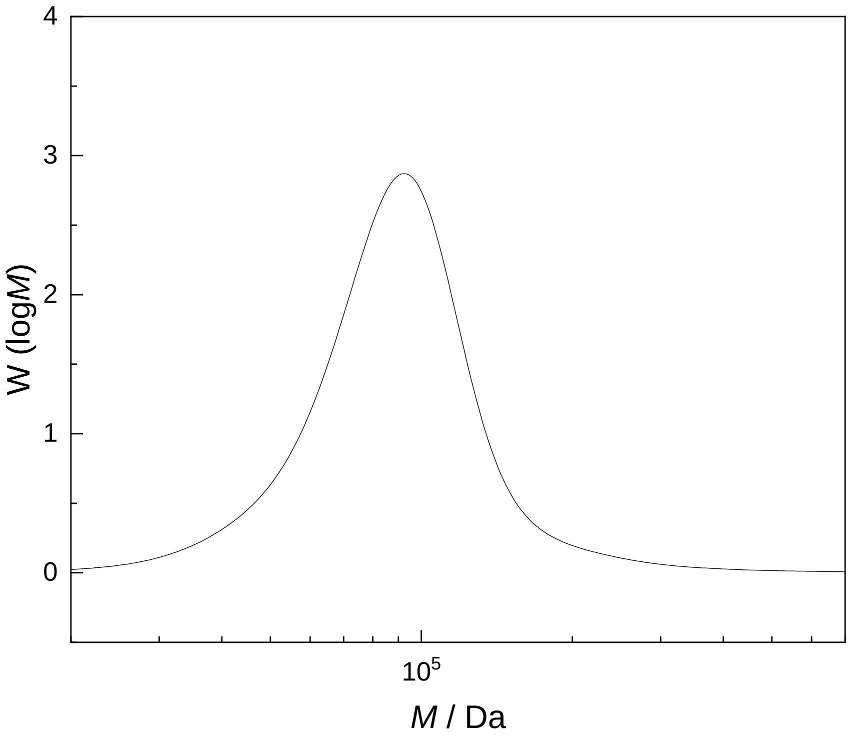


**Figure S86.** Molar mass distribution of GPC RI chromatogram (eluent: THF) of **PMA6$_{80}$**.

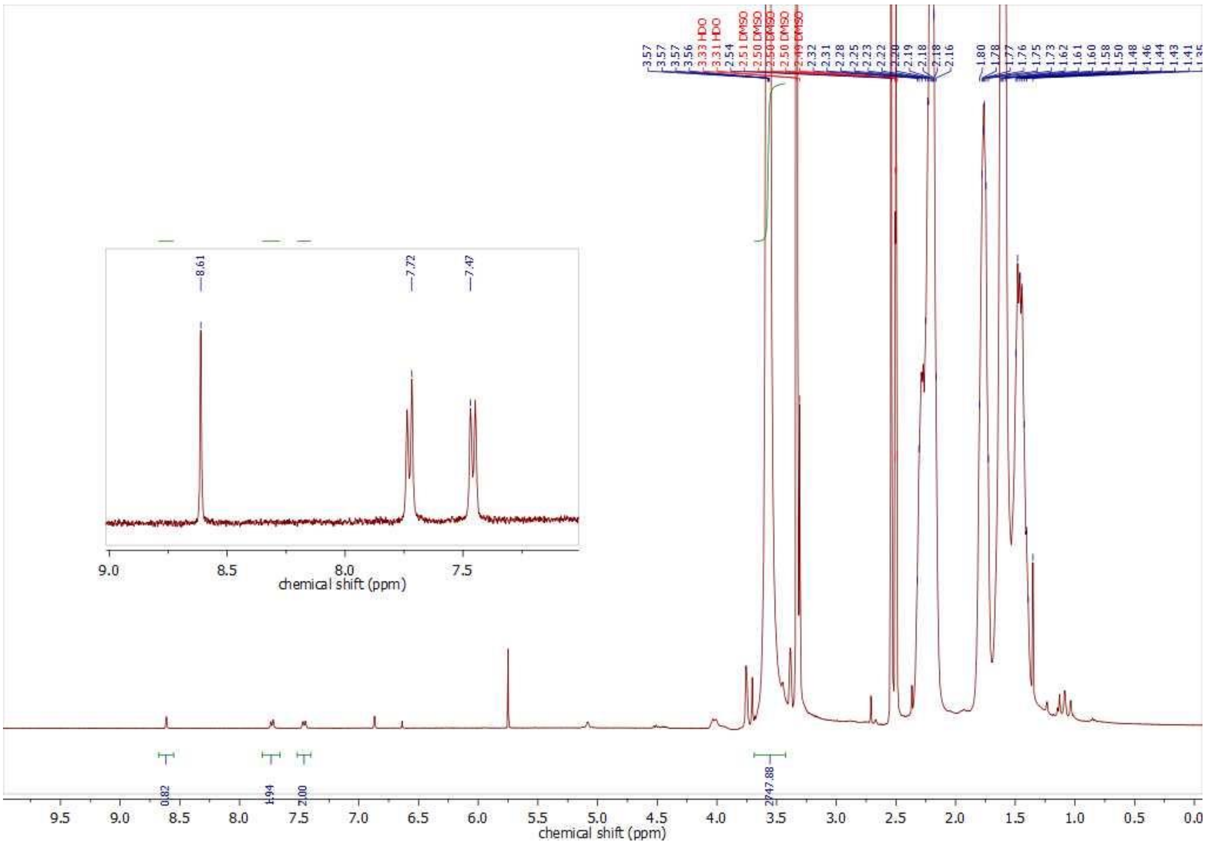


**Figure S87.** $^1$H-NMR spectrum of **PMA6$_{90}$**.

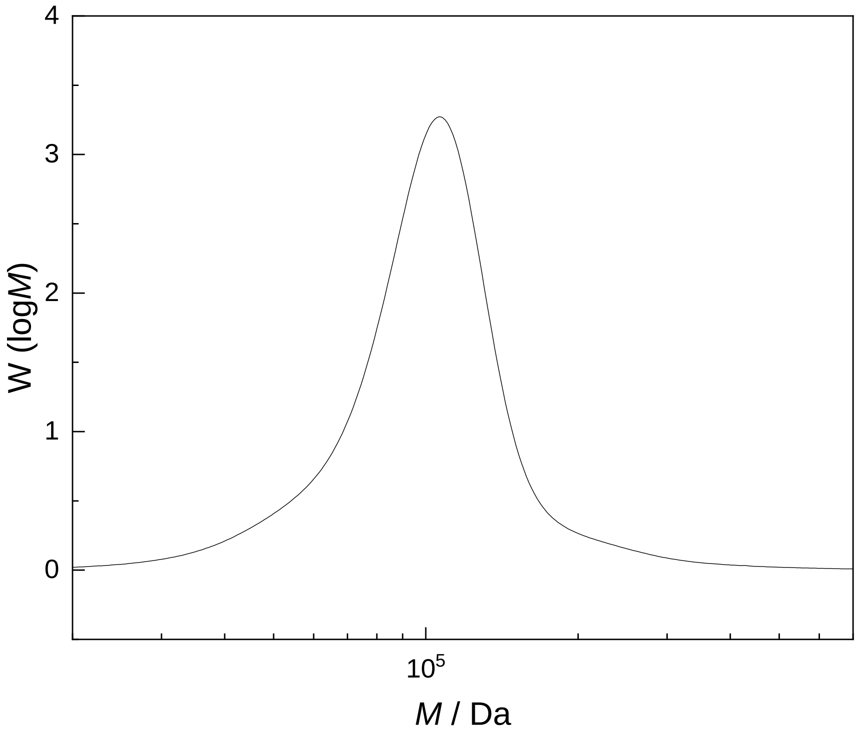


**Figure S88**. Molar mass distribution of GPC RI chromatogram (eluent: THF) of **PMA6$_{90}$**.

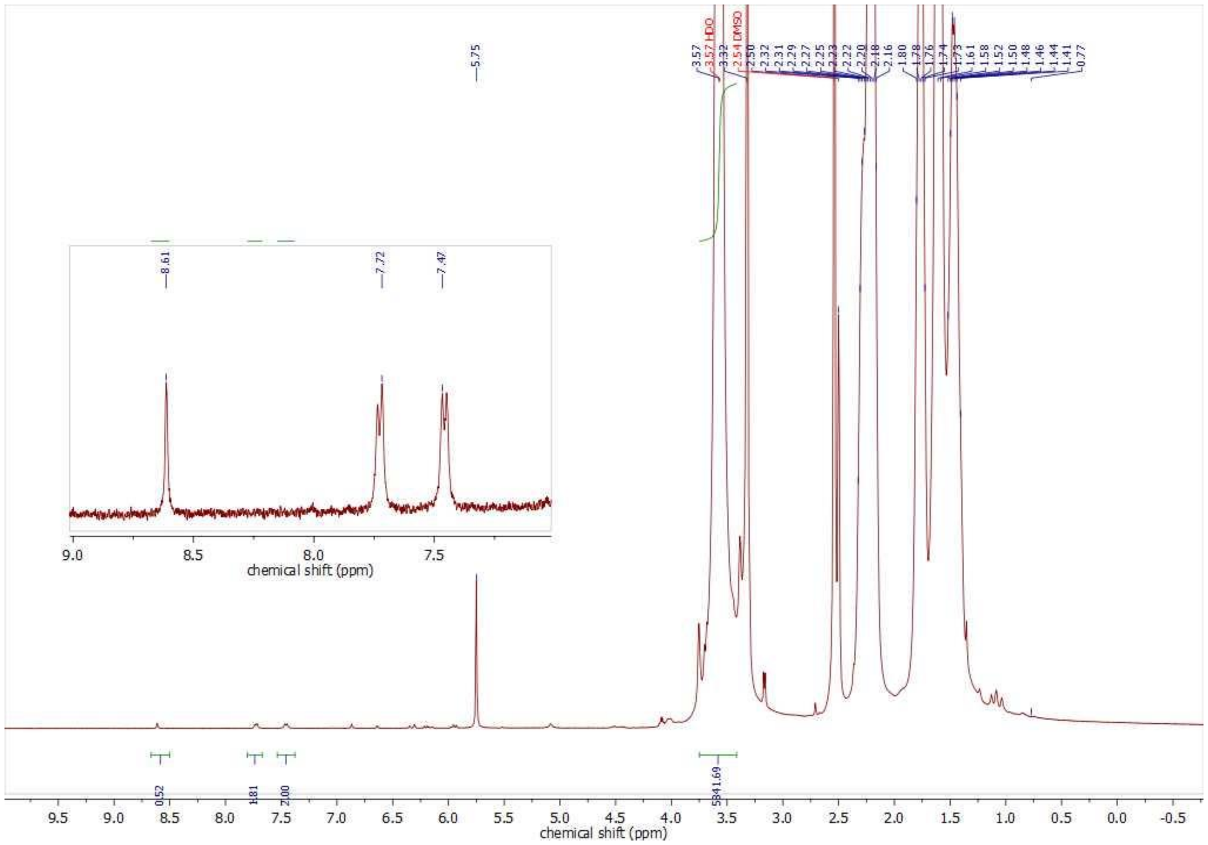


**Figure S89**. $^1$H-NMR spectrum of **PMA6$_{145}$**.

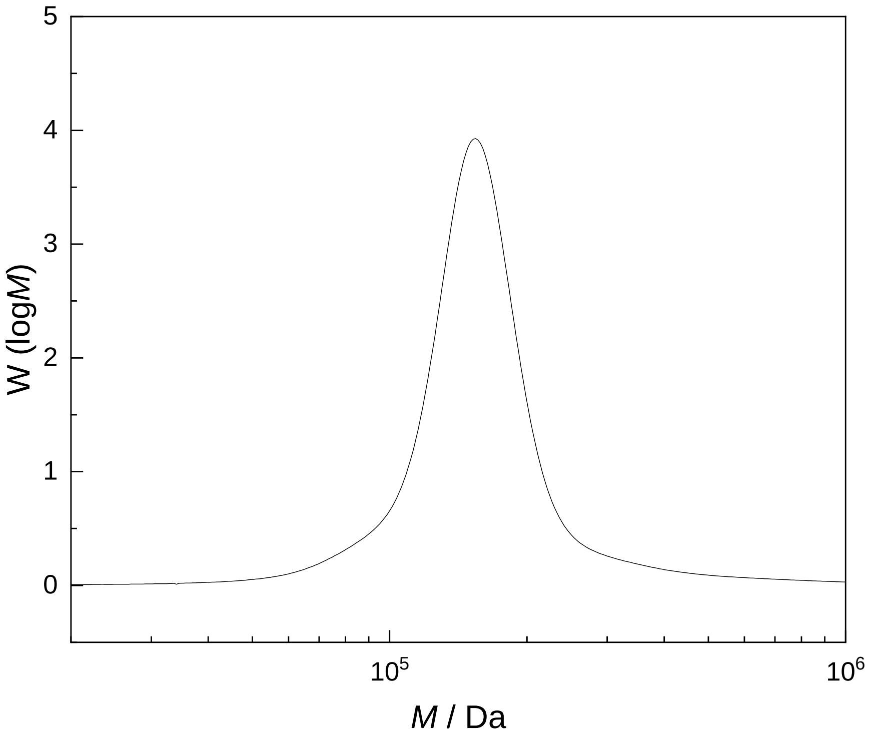


**Figure S90**. Molar mass distribution of GPC RI chromatogram (eluent: THF) of **PMA6$_{145}$**.

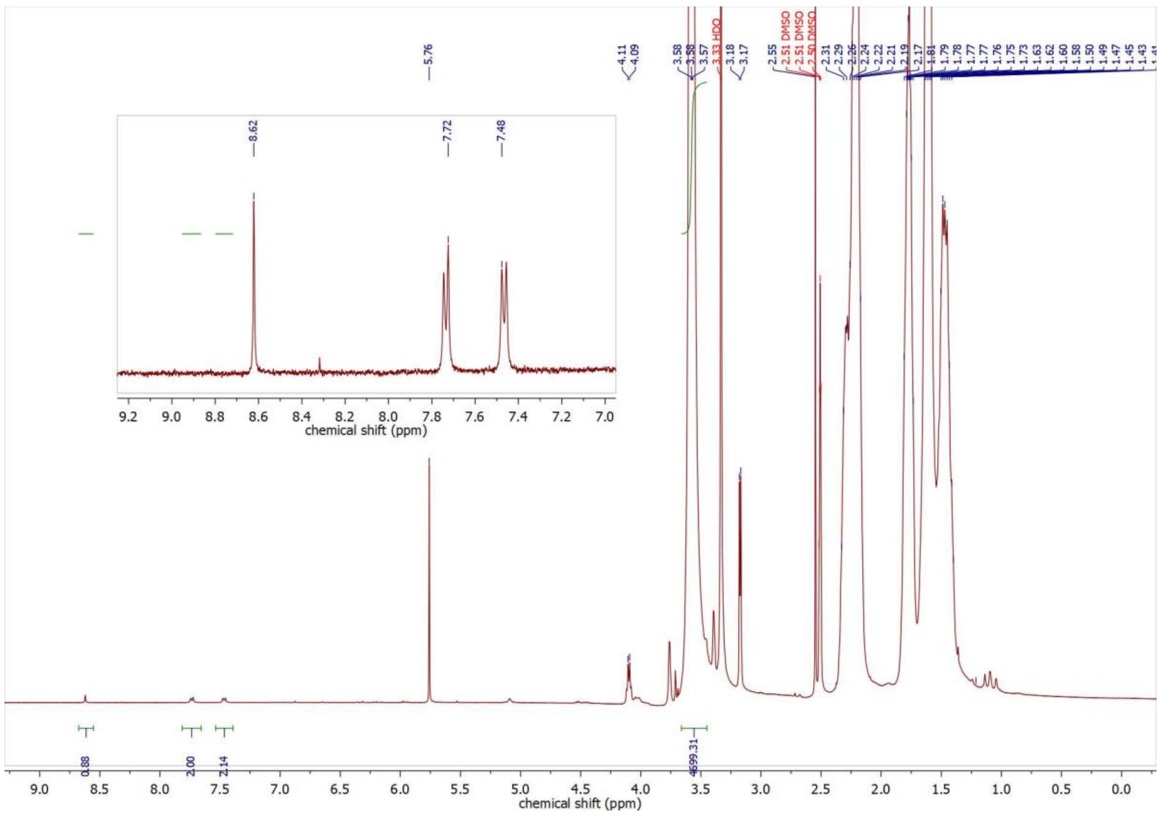


**Figure S91.** $^{1}$H-NMR spectrum of **PMA6$_{134}$**.

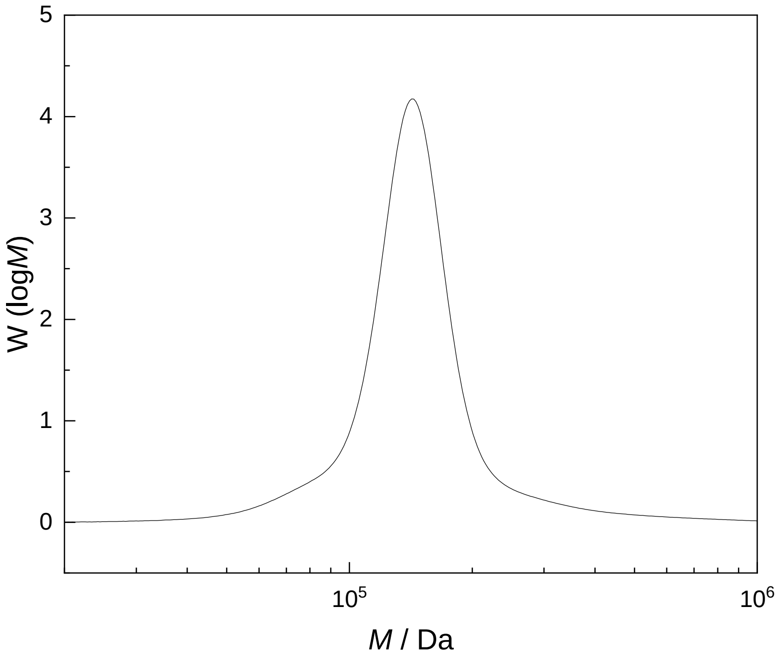


**Figure S92**. Molar mass distribution of GPC RI chromatogram (eluent: THF) of **PMA6$_{134}$**.

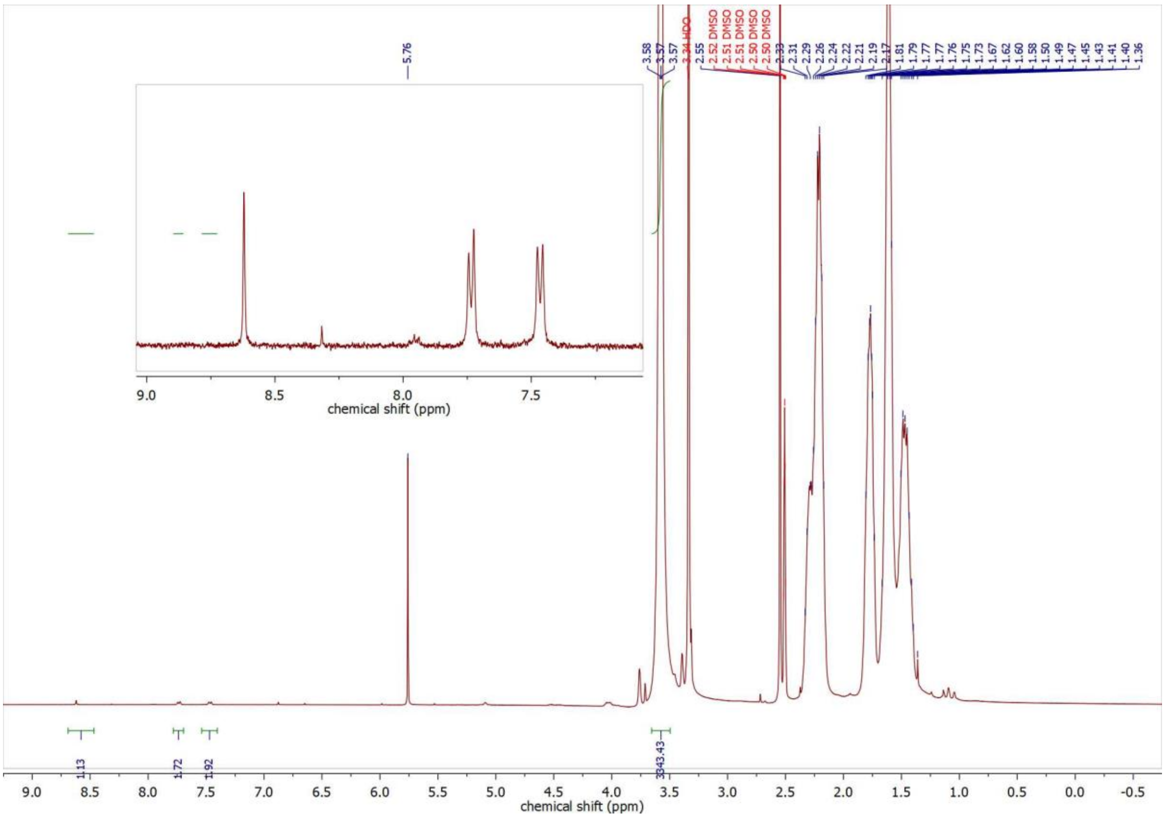


**Figure S93**. $^{1}$H-NMR spectrum of **PMA6$_{116}$**.

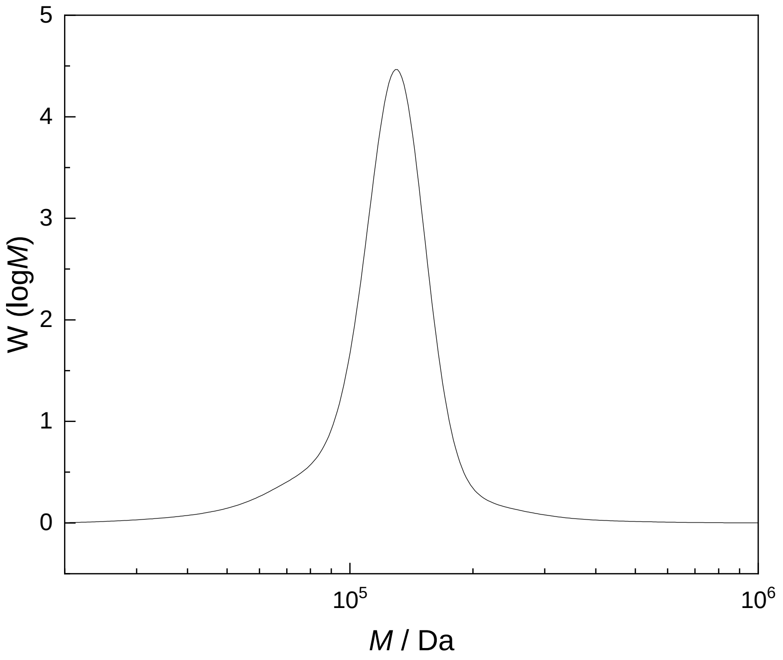


**Figure S94.** Molar mass distribution of GPC RI chromatogram (eluent: THF) of **PMA6$_{116}$**.

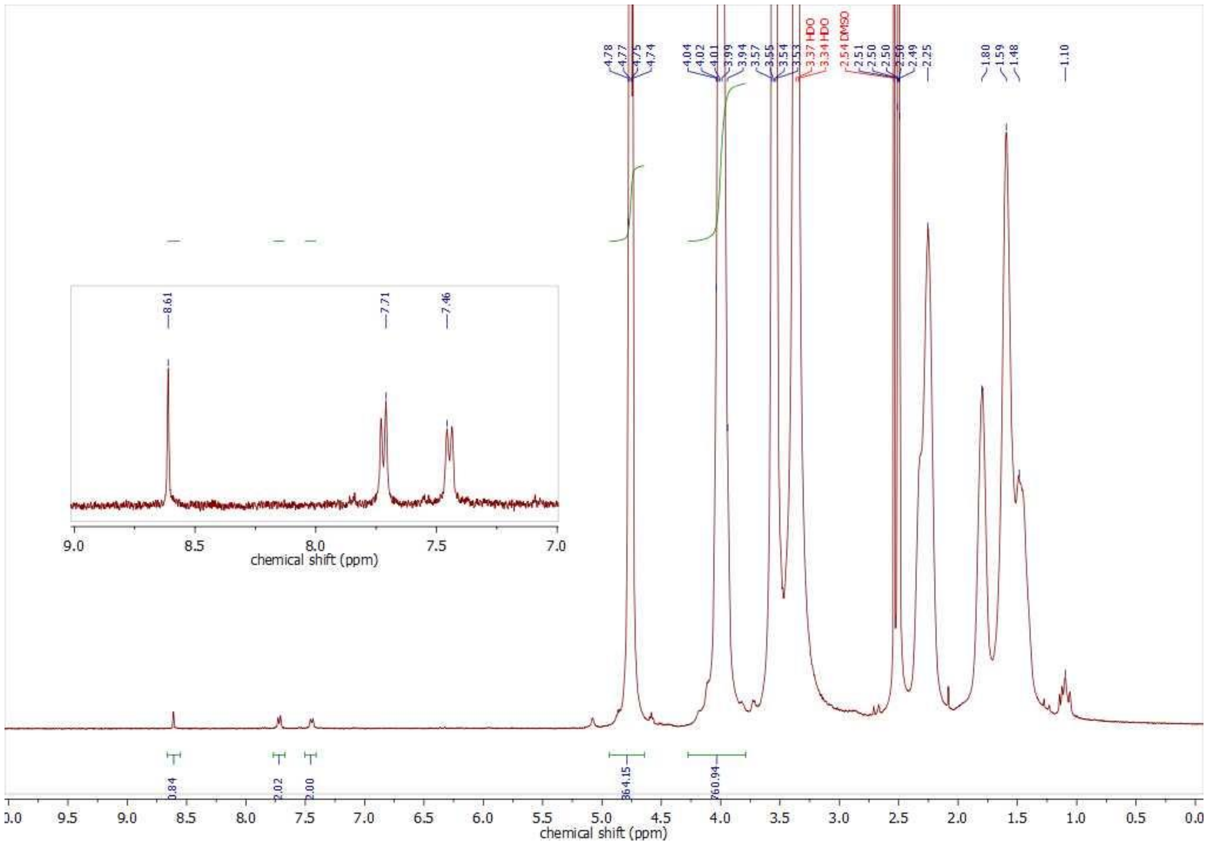


**Figure S95.** $^1$H-NMR spectrum of **PHEA6$_{44}$**.

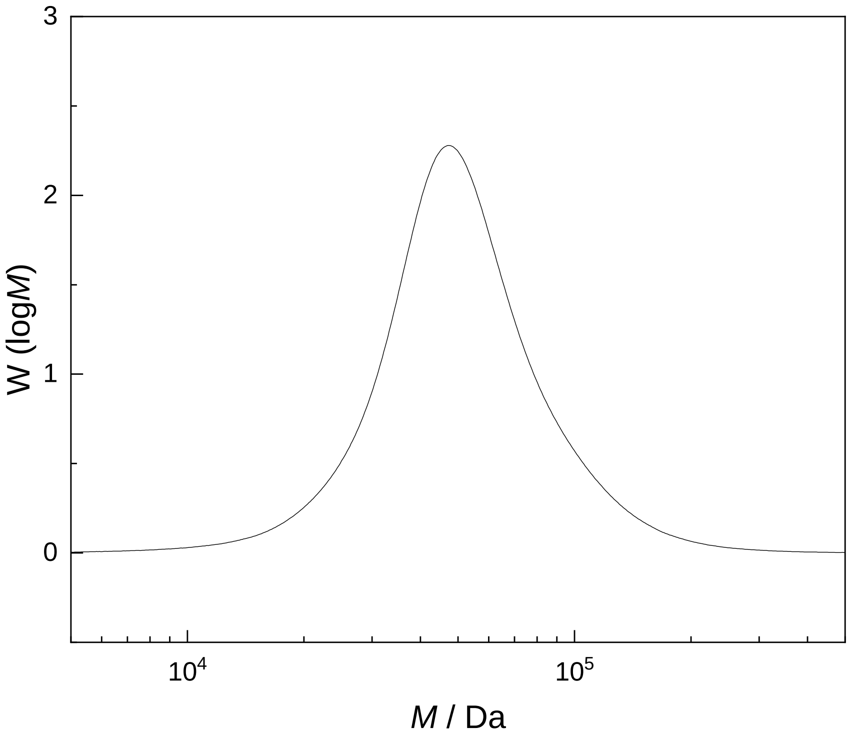


**Figure S96.** Molar mass distribution of GPC RI chromatogram (eluent: DMF) of **PHEA6$_{44}$**.

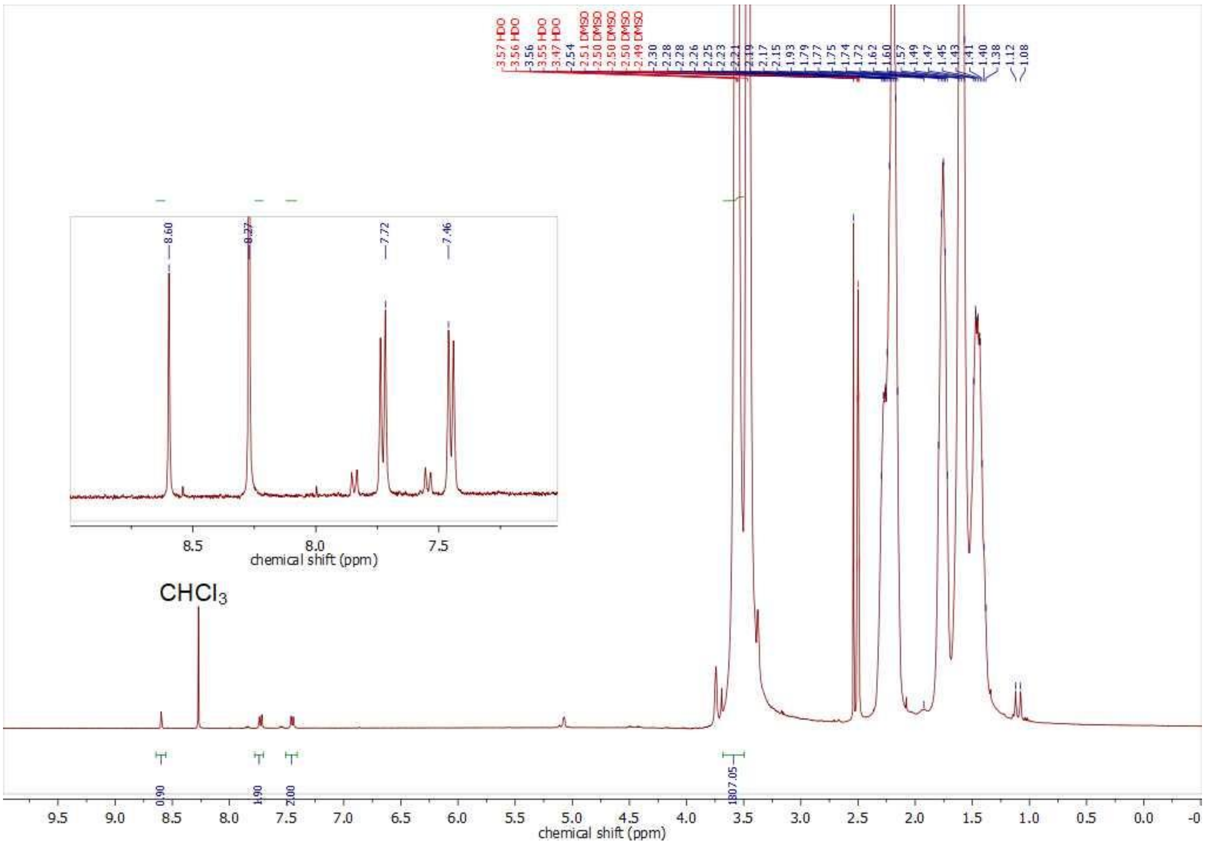


**Figure S97**. $^1$H-NMR spectrum of **PMA14**.

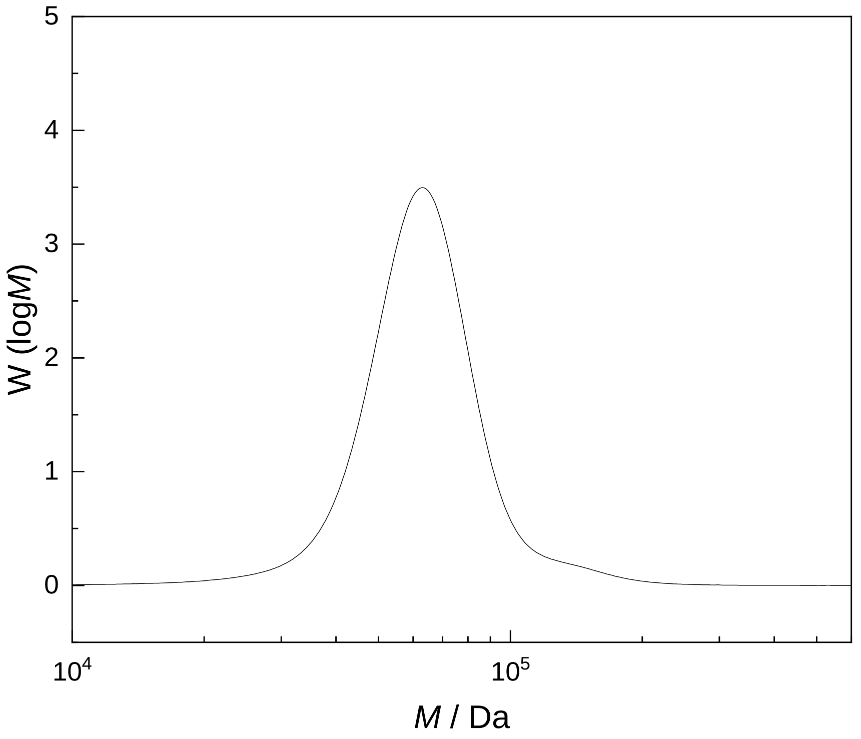


**Figure S98**. Molar mass distribution of GPC RI chromatogram (eluent: THF) of **PMA14.**

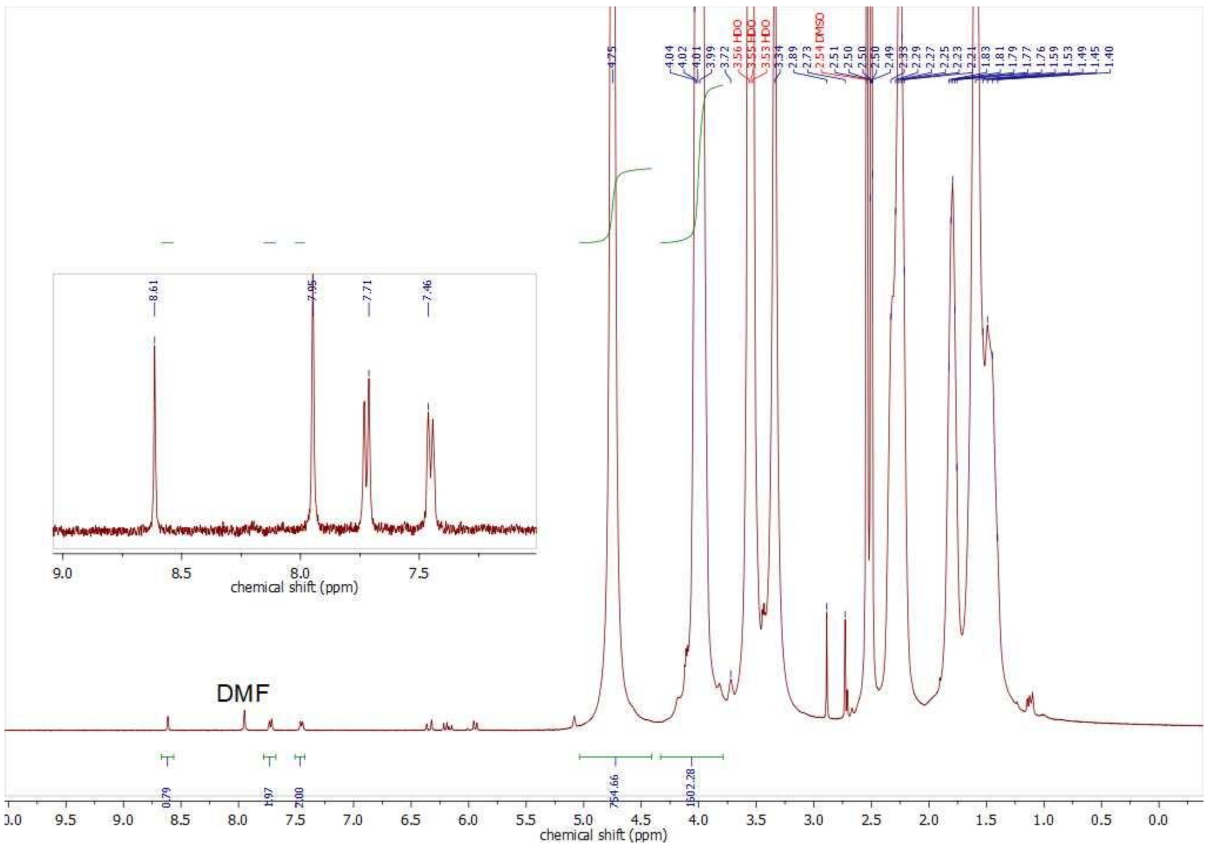


**Figure S99**. $^{1}$H-NMR spectrum of **PHEA14**.

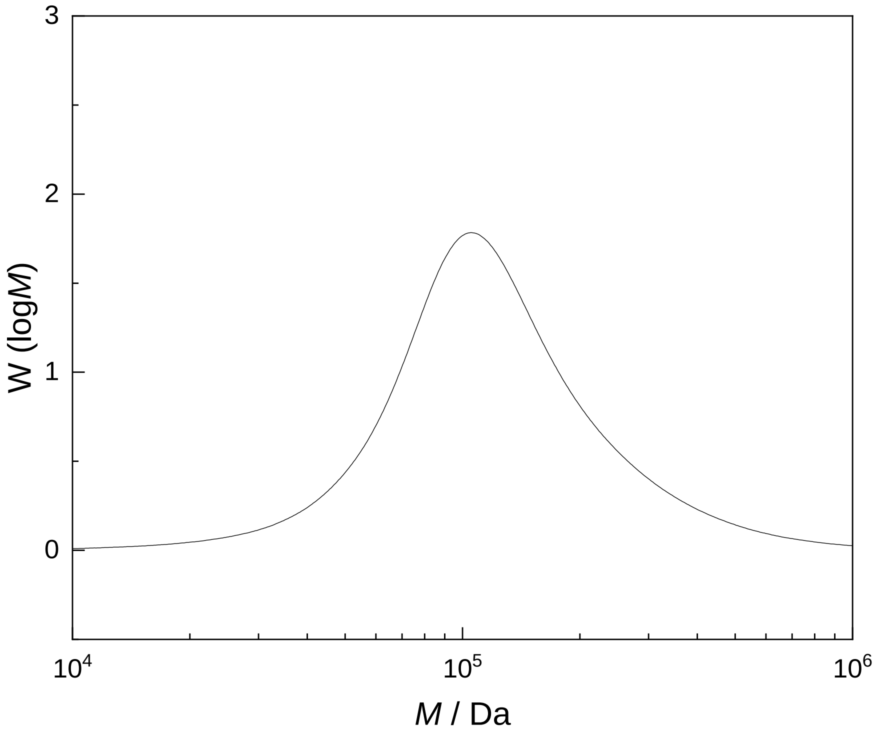


**Figure S100**. Molar mass distribution of GPC RI chromatogram (eluent: DMF) of **PHEA14**.

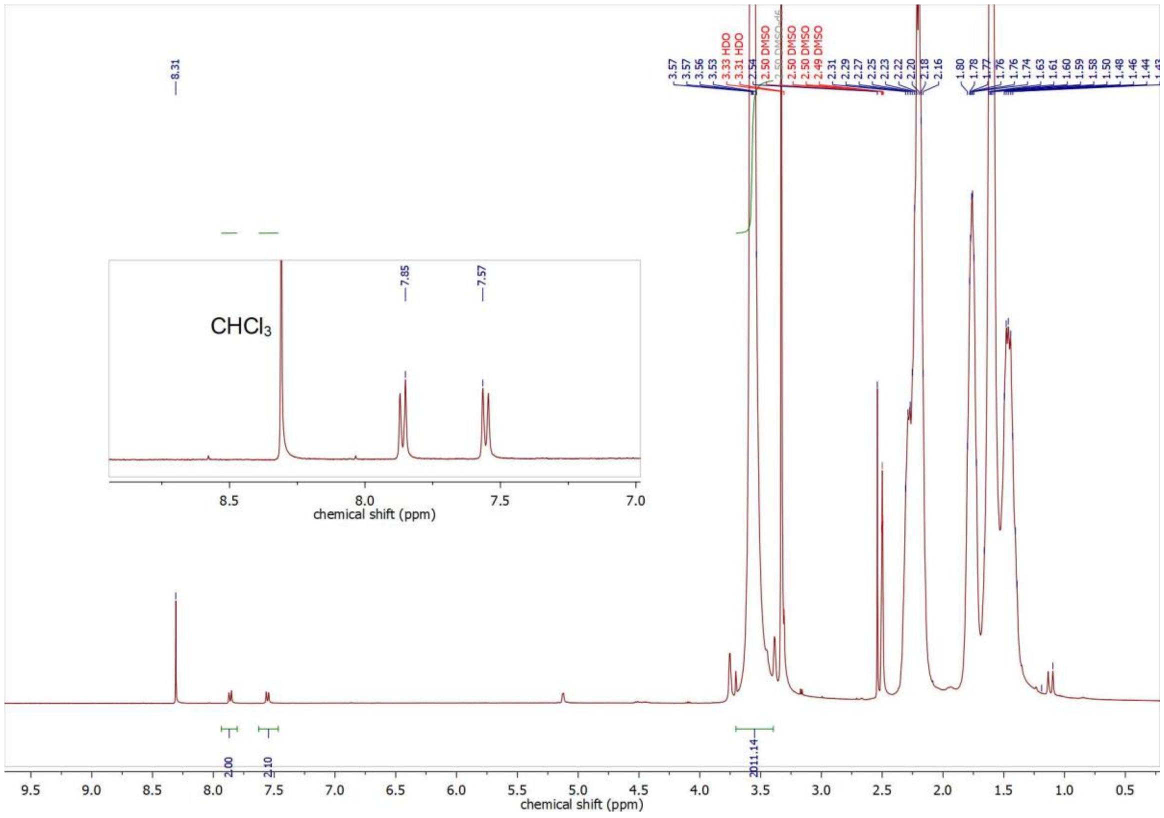


**Figure S101.** $^1$H-NMR spectrum of **PMA7**.

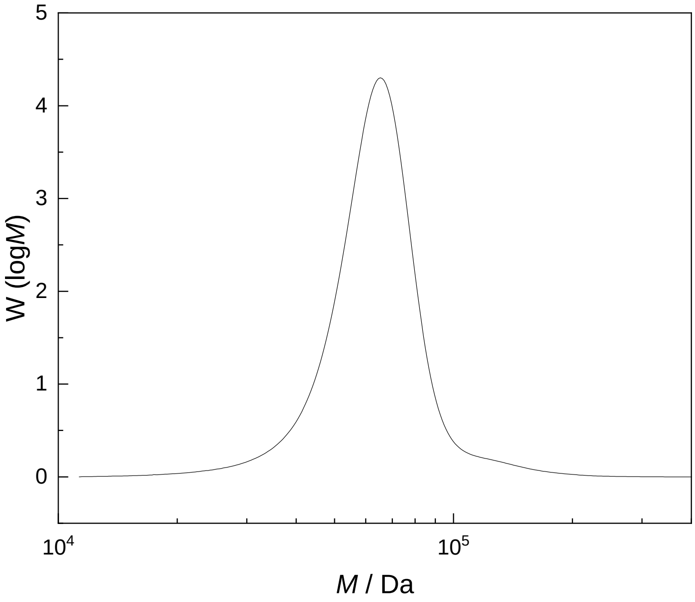


**Figure S102.** Molar mass distribution of GPC RI chromatogram (eluent: THF) of **PMA7**.

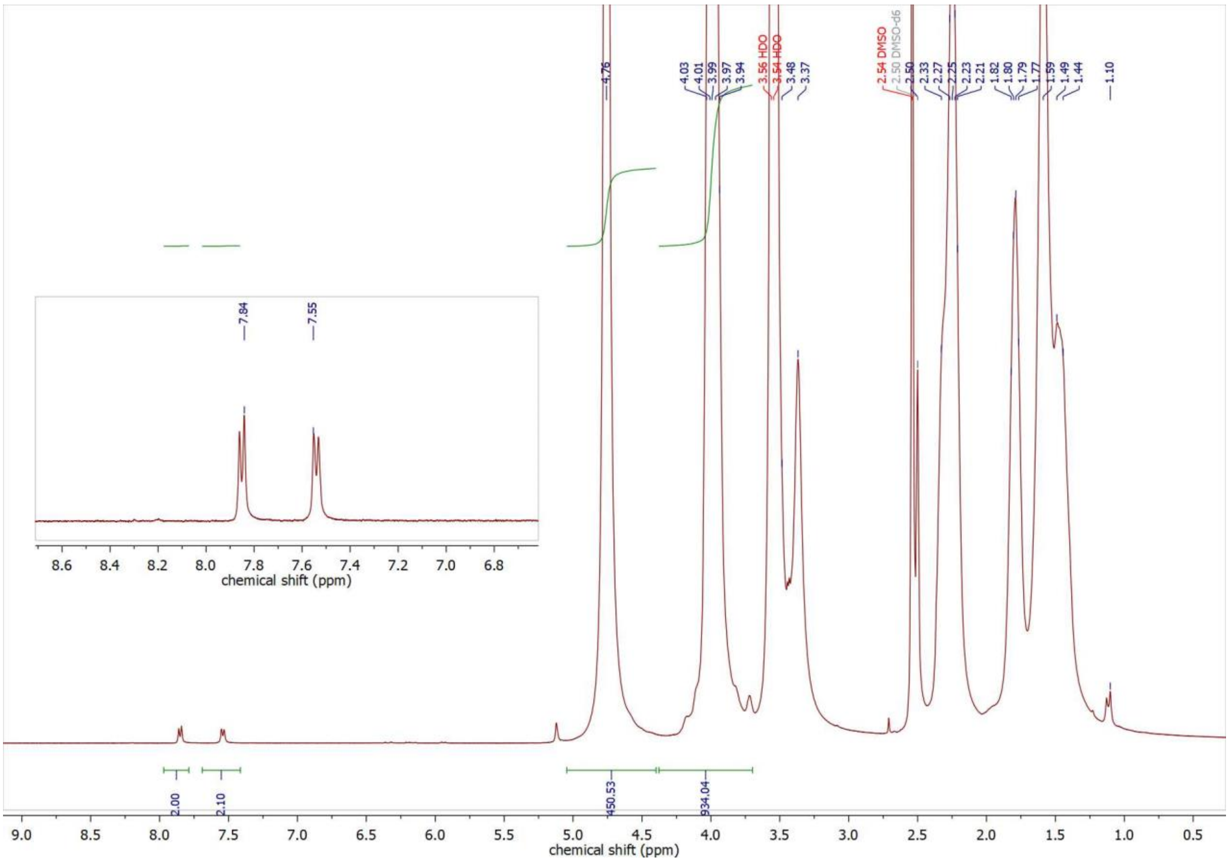


**Figure S103.** $^{1}$H-NMR spectrum of **PHEA7**.

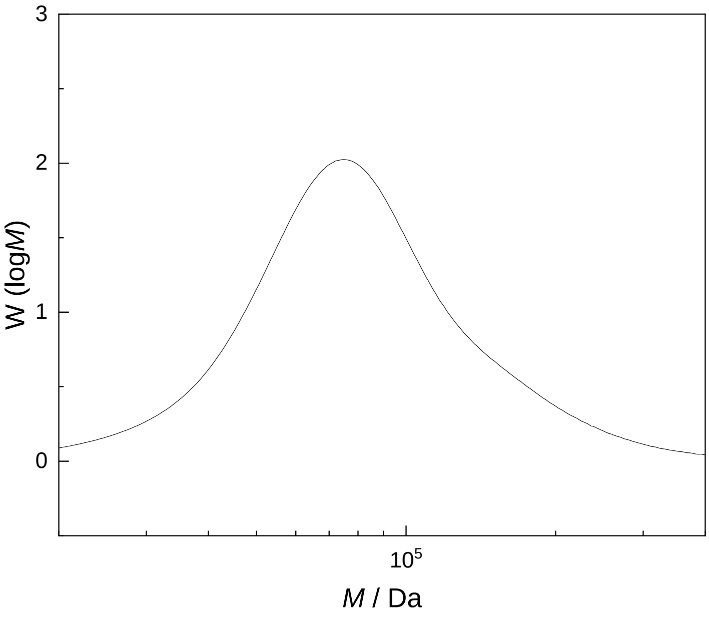


**Figure S104.** Molar mass distribution of GPC RI chromatogram (eluent: DMF) of **PHEA7**.

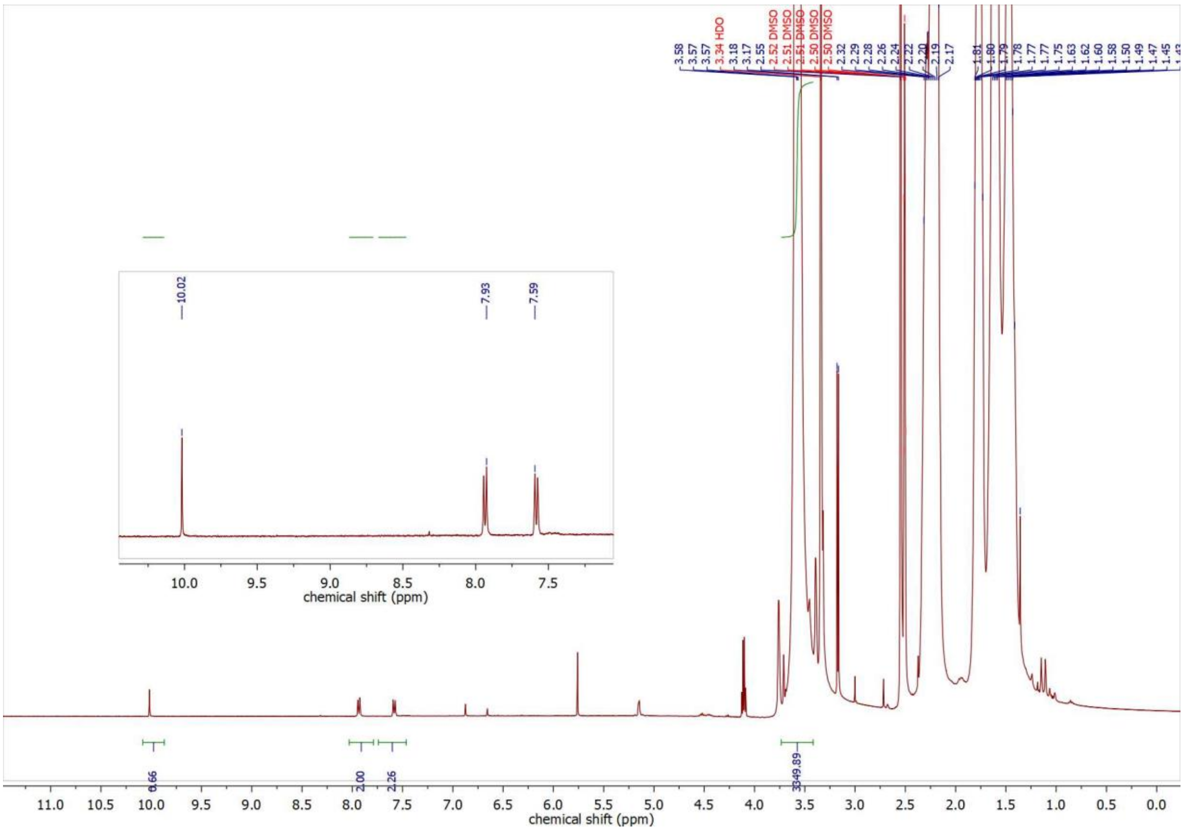


**Figure S105**. $^{1}$H-NMR spectrum of **PMA8**.

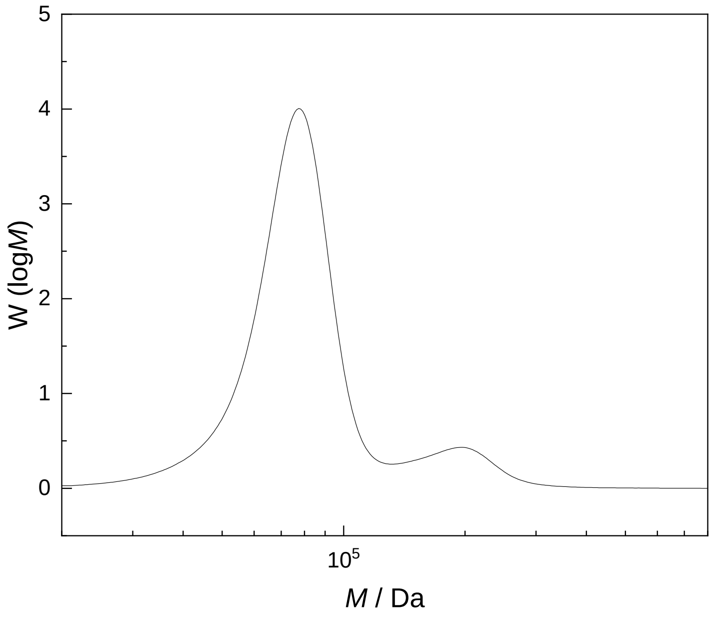


**Figure S106.** Molar mass distribution of GPC RI chromatogram (eluent: THF) of **PMA8**.

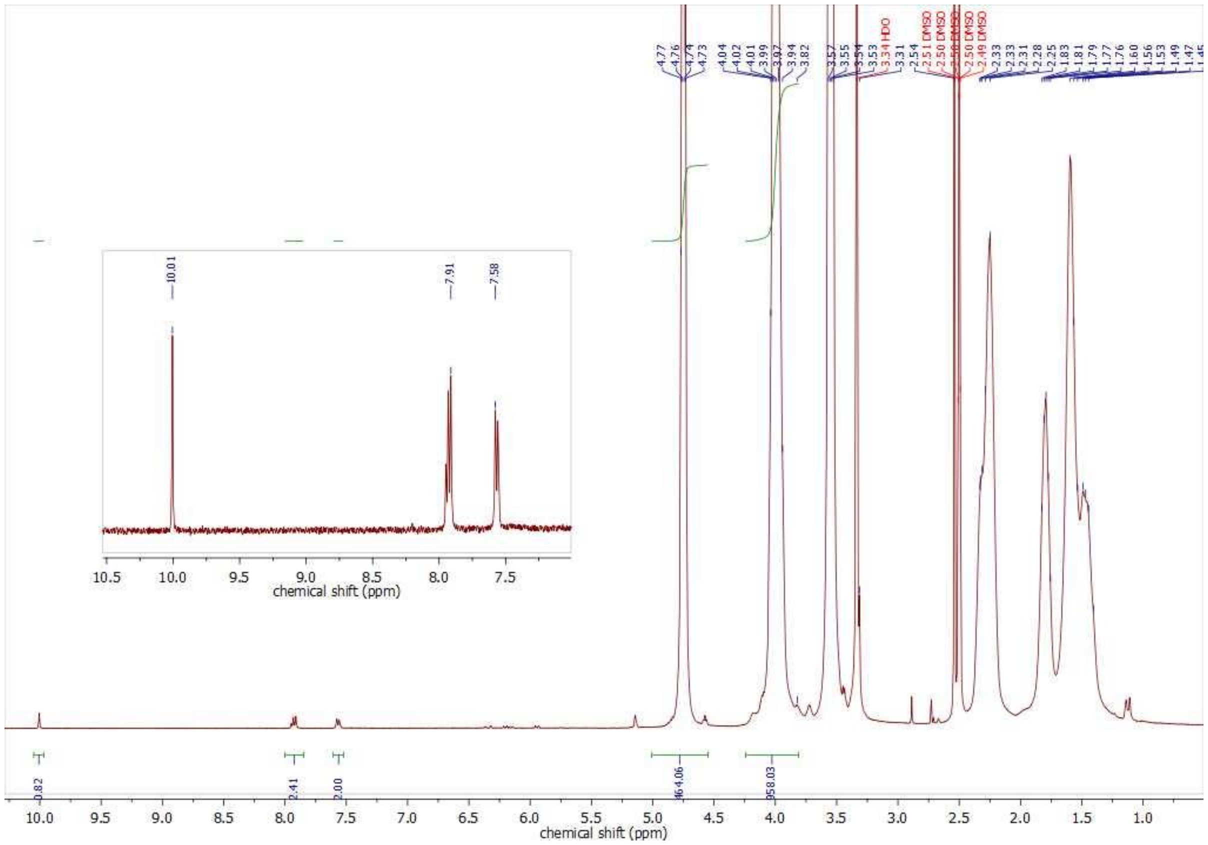


**Figure S107**. $^1$H-NMR spectrum of **PHEA8**.

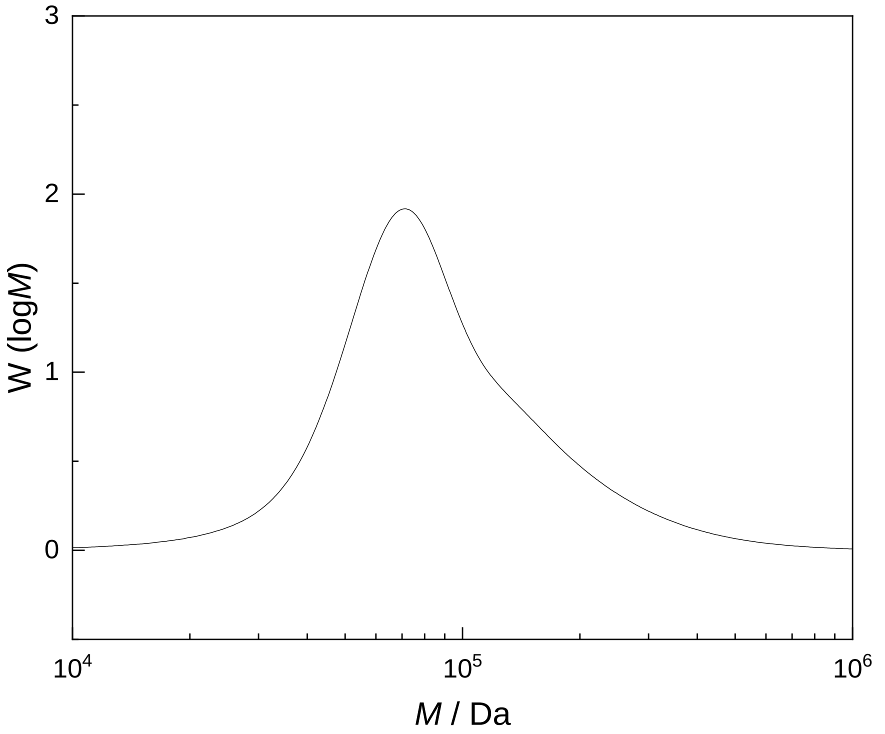


**Figure S108.** Molar mass distribution of GPC RI chromatogram (eluent: DMF) of **PHEA8**.

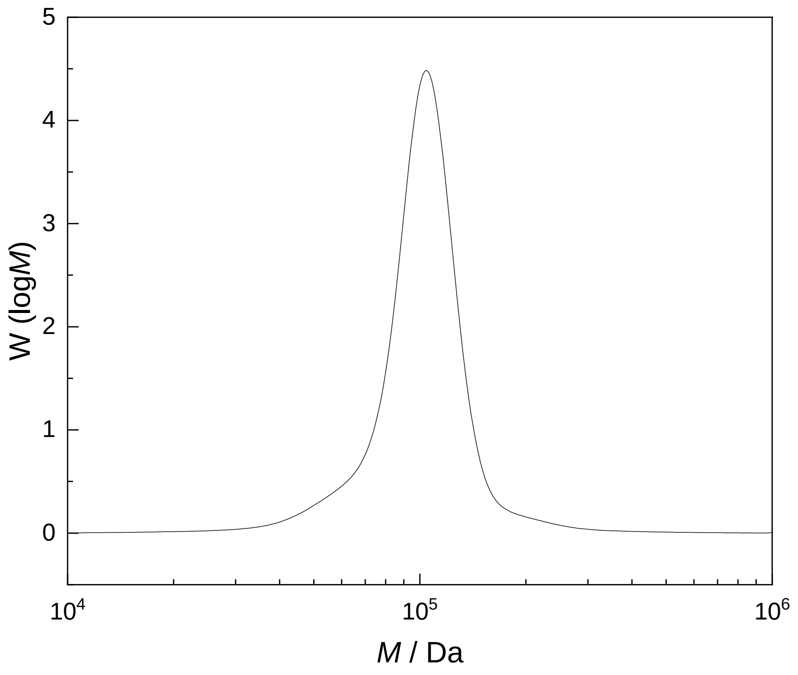


**Figure S109.** Molar mass distribution of GPC RI chromatogram (eluent: THF) of **$PMA_{94}$**.

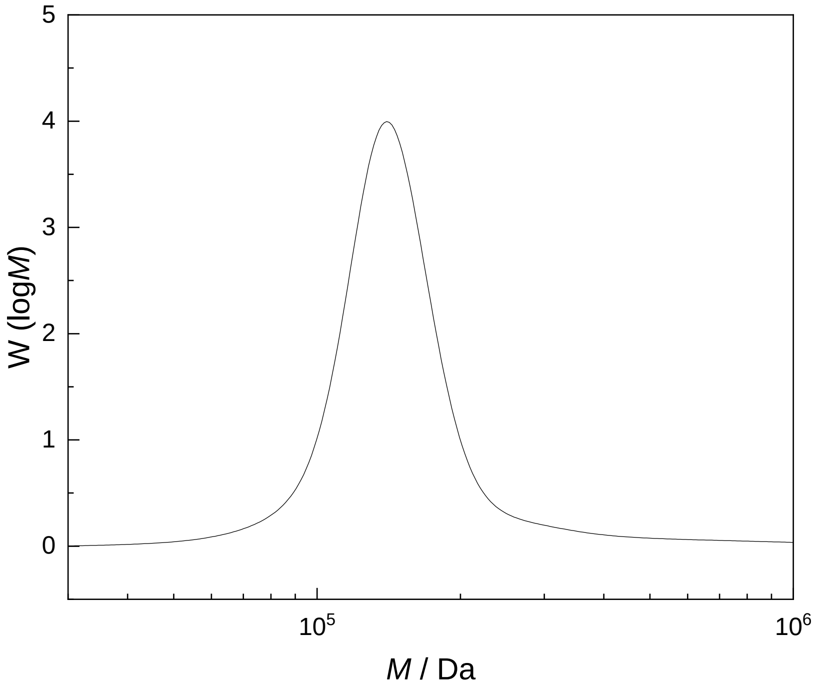


**Figure S110.** Molar mass distribution of GPC RI chromatogram (eluent: THF) of $\mathbf{PMA_{139}}$.

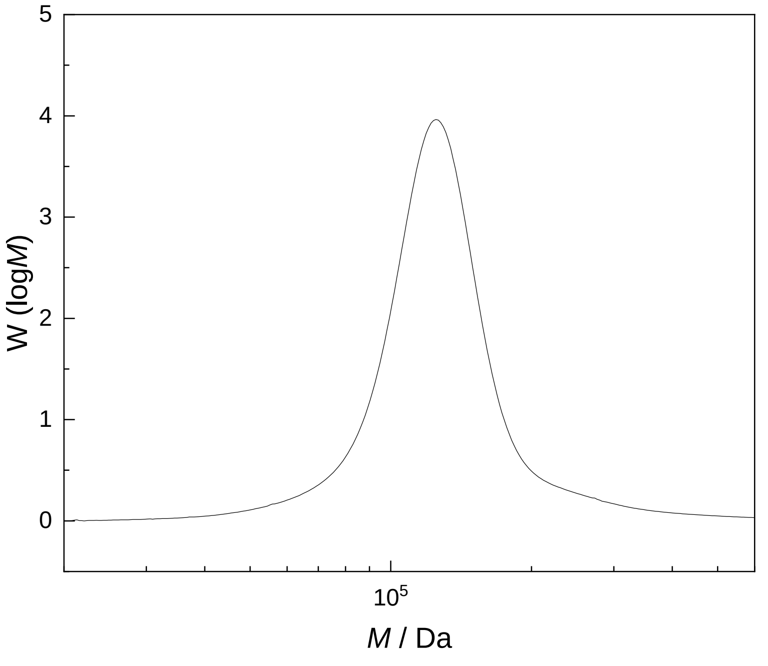


**Figure S111**. Molar mass distribution of GPC RI chromatogram (eluent: THF) of $\mathbf{PMA_{119}}$.

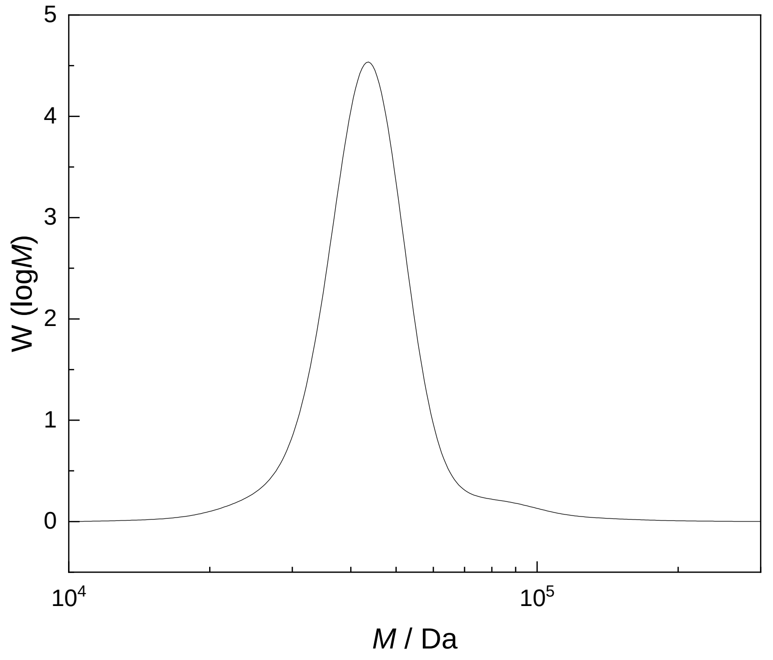


**Figure S112**. Molar mass distribution of GPC RI chromatogram (eluent: THF) of $\mathbf{PMA_{42}}$.

# 4. References


[1] S. L. Potisek, D. A. Davis, N. R. Sottos, S. R. White, J. S. Moore, *J. Am. Chem. Soc.* **2007**, *129*, 13808–13809.
[2] X. Leng, N. H. Nguyen, B. Van Beusekom, D. A. Wilson, V. Percec, *Polym. Chem.* **2013**, *4*, 2995–3004.
[3] J. Walton, *Molecules* **2016**, *21*, 63.
[4] J. A. Blake, K. U. Ingold, S. Lin, P. Mulder, D. A. Pratt, B. Sheeller, J. C. Walton, *Org. Biomol. Chem.* **2004**, *2*, 415–420.
[5] A. J. McCarroll, J. C. Walton, *J. Chem. Soc. Perkin Trans. 2* **2000**, 1868–1875.
[6] R. T. McBurney, J. C. Walton, *J. Am. Chem. Soc.* **2013**, *135*, 7349–7354.
[7] R. Sutcliffe, K. U. Ingold, *J. Am. Chem. Soc.* **1982**, *104*, 6071–6075.
[8] S. Il Hong, T. Kurosaki, M. Okawara, *J. Polym. Sci. Part A: Polym. Chem.* **1972**, *10*, 3405–3419.
[9] M. J. Kryger, A. M. Munaretto, J. S. Moore, *J. Am. Chem. Soc.* **2011**, *133*, 18992–18998.
[10] M. K. Beyer, *J. Chem. Phys.* **2000**, *112*, 7307–7312.
[11] M. Schmittel, S. De, S. Pramanik, *Angew. Chem. Int. Ed.* **2012**, *51*, 3832–3836.


# 5. Author contributions

D. C. synthesized all molecules, conducted the experiments, and performed the calculations. R. G. formulated the concept, acquired funding, administrated, and supervised the project. D. C. and R. G. conceived the experiments, curated the data, and wrote the manuscript.
